# Functional Investigation of *IGF1R* Mutations in Multiple Myeloma

**DOI:** 10.3390/cancers16112139

**Published:** 2024-06-04

**Authors:** Sofia Catalina Heredia-Guerrero, Marietheres Evers, Sarah Keppler, Marlene Schwarzfischer, Viktoria Fuhr, Hilka Rauert-Wunderlich, Anne Krügl, Theodora Nedeva, Tina Grieb, Julia Pickert, Hanna Koch, Torsten Steinbrunn, Otto-Jonas Bayrhof, Ralf Christian Bargou, Andreas Rosenwald, Thorsten Stühmer, Ellen Leich

**Affiliations:** 1 Institute of Pathology, University of Würzburg, 97080 Würzburg, Germanymarietheres.evers@uni-wuerzburg.de (M.E.); hilka.rauert-wunderlich@uni-wuerzburg.de (H.R.-W.); anne.kruegl@uni-wuerzburg.de (A.K.); theodora.nedeva@uni-wuerzburg.de (T.N.); tina.grieb@uni-wuerzburg.de (T.G.); rosenwald@uni-wuerzburg.de (A.R.); 2 Department of Internal Medicine II, University Hospital Würzburg, 97080 Würzburg, Germany; steinbrunn_t@ukw.de; 3 Department of Medical Oncology, Dana-Farber Cancer Institute, Harvard Medical School, Boston, MA 02115, USA; 4 Comprehensive Cancer Center Mainfranken, University Hospital Würzburg, 97080 Würzburg, Germanybargou_r@ukw.de (R.C.B.); stuehmer_t@ukw.de (T.S.)

**Keywords:** multiple myeloma, receptor tyrosine kinase signaling, *IGF1R* mutations, linsitinib, carfilzomib

## Abstract

**Simple Summary:**

Overexpression and/or mutations of the receptor tyrosine kinase IGF1R are associated with an adverse prognosis in MM but do not appear to have any impact on treatment response, and their functional role in MM is so far unknown. In the current study, we aim to understand the impact of *IGF1R* mutations on MM cell survival signaling, viability/proliferation and treatment response. We show that *IGF1R* mutations can impact IGF1R activation and/or downstream signaling and that the combination of the pIGF1R/pINSR inhibitor linsitinib with the second-generation proteasome inhibitor carfilzomib shows promising anti-myeloma activity, regardless of the *IGF1R* mutation status.

**Abstract:**

High expression of the receptor tyrosine kinase (RTK) insulin-like growth factor-1 receptor (*IGF1R*) and RTK mutations are associated with high-risk/worse prognosis in multiple myeloma (MM). Combining the pIGF1R/pINSR inhibitor linsitinib with the proteasome inhibitor (PI) bortezomib seemed promising in a clinical trial, but IGF1R expression was not associated with therapy response. Because the oncogenic impact of *IGF1R* mutations is so far unknown, we investigated the functional impact of *IGF1R* mutations on survival signaling, viability/proliferation and survival response to therapy. We transfected four human myeloma cell lines (HMCLs) with *IGF1R*^WT^, *IGF1R^D^*^1146N^ and *IGF1R*^N1129S^ (Sleeping Beauty), generated CRISPR-Cas9 *IGF1R* knockouts in the HMCLs U-266 (IGF1R^WT^) and L-363 (IGF1R^D1146N^) and tested the anti-MM activity of linsitinib alone and in combination with the second-generation PI carfilzomib in seven HMCLs. *IGF1R* knockout entailed reduced proliferation. Upon IGF1R overexpression, survival signaling was moderately increased in all HCMLs and slightly affected by *IGF1R^N1129S^* in one HMCL, whereby the viability remained unaffected. Expression of IGF1R^D1146N^ reduced pIGF1R-Y1135, especially under serum reduction, but did not impact downstream signaling. Linsitinib and carfilzomib showed enhanced anti-myeloma activity in six out of seven HMCL irrespective of the *IGF1R* mutation status. In conclusion, *IGF1R* mutations can impact IGF1R activation and/or downstream signaling, and a combination of linsitinib with carfilzomib might be a suitable therapeutic approach for MM patients potentially responsive to IGF1R blockade.

## 1. Introduction

Multiple myeloma (MM) is a malignant plasma cell neoplasm for which considerable treatment progress has been made in recent years [1,2,3]. Nevertheless, despite significant improvements in quality of life and length of survival for many patients, these treatments remain non-curative [4]. Except for a few individualized therapeutic concepts [5,6,7] (e.g., for MM patients with BRAF^V600E^ [5]), there are still no approved tumor genetics-based personalized therapies for MM. This may be mostly due to the marked genetic heterogeneity of this disease, which displays very few recurrent and druggable oncogenic lesions [8,9,10].

As in many oncologic diseases, the effectors of central growth and survival signals, such as RAS/MAPK and AKT signaling, are important in MM [11,12,13,14,15,16]. However, no clear correlation between the potential intrinsic activation of these effectors and the clinical course of the disease has been demonstrated so far, perhaps because of signal pathway redundancies. Receptor tyrosine kinases (RTKs), such as insulin-like growth factor 1 receptor (IGF1R), epidermal growth factor receptor (EGFR), fibroblast growth factor receptor 3 (FGFR3) and erb-b2 receptor tyrosine kinase 2 (ERBB2), which are intimately connected with growth, survival, differentiation and migration processes, may play an important role in this regard [17,18,19]. Genetic alterations of RTKs and of their effectors have been linked to tumor development in various cancers, and preclinical experiments as well as clinical studies have shown that patients with RTK mutations or aberrant RTK expression may benefit from treatment with RTK inhibitors [20,21,22,23,24,25].

We have previously described an accumulation of single nucleotide variants (SNVs) and patient-specific single nucleotide polymorphisms (SNPs) in RTK genes (including *IGF1R* as the most recurrently mutated RTK), detected in MM primary samples and human MM cell lines (HMCLs) [10,26]. Interestingly, the presence of these mutations was associated with a significantly worse prognosis [26,27].

Increased expression of IGF1R is mostly detectable in patients with high-risk MM and associated with a poor prognosis [28]. Additionally, increased expression of the IGF1R ligand IGF1 has been associated with progression from MGUS to MM, and IGF1 was shown to be an important autocrine and paracrine growth and survival factor for MM in vitro and in vivo [29,30].

Although IGF1R is supposed to be an important oncogene in MM and other malignant diseases [30,31,32] and promising findings were made in vitro [33,34,35,36], significant clinical responses to monotherapies with IGF1R inhibitors have not been achieved in patients [37,38]. This might be due to compensation mechanisms employing other growth factors, e.g., through the ability of IGF1R to form hybrid receptors [37,38,39]. On the other hand, the small-molecule dual pIGF1R/pINSR inhibitor linsitinib has recently been employed in combination with the first-generation proteasome inhibitor (PI) bortezomib and dexamethasone to achieve responses in PI-refractory MM patients [31]. However, because these effects did not correlate with IGF1R expression or the CD45 phenotype of MM cells [31,33,34], it remains unclear what characterizes cells sensitive to IGF1R inhibition, highlighting the lack of biological or biomarker-based information [31]. Moreover, it was shown that IGF1R expression and its activation were contributing to bortezomib resistance [40]. Understanding the potential functional impact of *IGF1R* mutations detected in MM on survival signaling, viability/proliferation and therapy is therefore desirable.

We initially used siRNA-mediated IGF1R downregulation and IGF1 stimulation in HMCLs (n = 7) to underline published findings [29,32,33,34,36,41,42,43] and studied the impact of IGF1R overexpression and mutant IGF1R on the RTK effectors and growth/survival markers AKT, MEK and ERK using four different stably transfected HMCLs grown under normal culturing conditions or in serum-starved medium. Moreover, we investigated the impact of CRISPR-Cas9-mediated *IGF1R* knockout on the proliferation in the *IGF1R*-mutant HMCL L-363 and the *IGF1R* wild-type (WT) HMCL U-266 and analyzed the response to IGF1R inhibition in seven HMCLs using linsitinib in combination with the second-generation PI carfilzomib, commonly used in relapsed refractory MM [44].

## 2. Materials and Methods

### 2.1. Cell Culture

The HMCLs L-363 (IGF1R^D1146N^) (RRID:CVCL_1357) [10,26], JJN-3 (IGF1R^WT^) (RRID:CVCL_2078), KMS-12-BM (IGF1R^WT^) (RRID:CVCL_1334), U-266 (IGF1R^WT^) (RRID:CVCL_J235) and AMO-1 (IGF1R^WT^) (RRID:CVCL_1806) were purchased from the “Deutsche Sammlung von Mikroorganismen und Zellkulturen GmbH” (DSMZ, Braunschweig, Germany). MM.1S (IGF1R^WT^) (RRID:CVCL_8792) was acquired from LGC Biolabs (Wesel, Germany) and KMS-11 (IGF1R^WT^) (RRID:CVCL_2989) from the Japanese Collection of Research Bioresources (JCRB, Osaka, Japan). The cells were cultured in RPMI-1640 supplemented with 10% FBS, 2mM L-glutamine and 1mM sodium pyruvate at 37 °C and 5% CO_2_ for a maximum of 3 months and regularly tested for mycoplasma using the VenorGEM One-Step kit (Minerva Biolabs, Berlin, Germany).

### 2.2. IGF1 Stimulation

Per condition, 3 × 10^6^ cells were cultured in serum-reduced medium (culturing medium with 0.5% FBS) for 18 h, stimulated with 20 ng/mL IGF1 (Immunotools, Friesoythe, Germany) for 10 min, immediately cooled on ice and then pelleted for the extraction of lysates. Unstimulated cells served as the controls.

### 2.3. Pharmacologic Inhibitors

The dual pIGF1R/pINSR inhibitor linsitinib (OSI-906) (#S1091) (Selleck Chemicals, Houston, TX, USA) and the PI carfilzomib (#S2853) (Selleck Chemicals, Houston, TX, USA) were dissolved in H_2_O-free DMSO and stored as 10 mM stock solutions at −80 °C. The working solutions were always freshly prepared in cell culture medium.

### 2.4. siRNA Knockdown

siRNA-mediated knockdown was performed using *IGF1R*-specific stealth siRNA (HSS105253; Invitrogen, Darmstadt, Germany). AllStar scrambled RNA (scrRNA) (Qiagen, Hilden, Germany) served as the control. Prior to electroporation with a Gene Pulser II (Bio-Rad, Munich, Germany), the cells were grown in culturing medium with 15% FBS overnight. The next day, 6 × 10^6^ cells per condition were washed with 1x PBS and resuspended in 200 µL fresh, unsupplemented RPMI-1640 medium containing either 2 µM *IGF1R* stealth siRNA or 2 µM scrRNA. Electroporations were performed with 2 mm cuvettes at 180 V (AMO-1, JJN-3 and L-363), 200 V (KMS-11, KMS-12-BM and MM.1S) or 230 V (U-266). Subsequently, the cell suspensions were immediately transferred to 500 µL unsupplemented RPMI-1640, kept at RT for 5 min and then transferred to 6-well plates with electroporation medium ((EP-medium) (culturing medium containing 15% FBS, 1x PenStrep)) and cultured at 37 °C. After 24 h, the living cells were separated from the debris by centrifugation at 800 rcf for 5 min and resuspension of the pellet in 2.5 mL EP-medium mixed with 750 µL OptiPrep (Progen, Heidelberg, Germany). The suspension was overlaid with 200 µL 1x PBS and centrifuged for 7 min at 2122 rcf. The layer of living cells was transferred from the medium/PBS interface to the EP-medium, spun down and plated in 6-well plates. The following day, the cells were pelleted and frozen for Western analyses.

### 2.5. CRISPR-Cas9 Screen

CRISPR-Cas9 experiments were performed using the Alt-R CRISPR-Cas9 System (IDT (Integrated DNA Technologies, Leuven, Belgium)) and guide RNAs targeting IGF1R (target-sequence exon 18 (RTK domain): GGACGAACTTATTGGCGTTG AGG; target-sequence exon 2: CCTGAGGAACATTACTC GGG) using the crRNA:tracrRNA duplex format [45]. First, the ribonucleoprotein (RNP) complex consisting of the crRNA:tracrRNA duplex and the Cas9 nuclease (IDT) was assembled and the RNP complex transfected into HMCL by electroporation (2 mm cuvettes, 190 V (L-363), 230 V (U-266)) or nucleofection (program X-005 for U-266, Amaxa nucleofector 2b (Lonza, Switzerland)). Single-cell clones were seeded and grown in 96-well plates in culturing medium supplemented with 2 ng/mL IL-6, 2 ng/mL VEGF-A and 100 U/mL TNF-α. Successful genome editing was confirmed by PCR and Sanger sequencing (primers listed in Table 1). Upon reaching sufficient cell numbers, the knockout clones were cultured in normal culturing medium no longer containing IL-6, VEGF-A and TNF-α.

### 2.6. Generation of IGF1R^WT^ and IGF1R^mut^-Overexpressing HMCL Sublines

#### 2.6.1. Mutagenesis PCR

An MM patient-derived *IGF1R* mutation (N1129S) [26] and the mutation intrinsic to L-363 cells (D1146N) [10,26] (Table S1) were introduced into IGF1R-cDNA by PCR using specific mutagenesis primers (Table S2), the Q5 DNA polymerase (New England Biolabs, Frankfurt, Germany (NEB)) and the plasmid pCR-XL-TOPO-IGF1R (Bioscience, Berlin, Germany) as the template [45]. Notably, both mutations are located within the conserved RTK domain and predicted to be “damaging” according to the polyphen score revealed by SeattleSeq annotation (Table S1). DpnI digestion was performed to destroy the template. Finally, *E. coli* NEB10β cells were transformed with the newly amplified plasmids. The plasmid minipreps were sequenced to confirm the presence of the desired mutations.

#### 2.6.2. Expression Cloning and Stable Transfection of HMCLs Using the Sleeping Beauty Transposon System

The mutant and WT IGF1R-cDNA were amplified from pCR-XL-TOPO-IGF1R-WT and pCR-XL-TOPO-IGF1R-mut plasmids and NotI and EcoRI restriction digestion sites introduced by PCR with Phusion High-Fidelity DNA polymerase (NEB, Frankfurt, Germany) (primers: Table S3). Subsequently, the PCR products and the expression vector pSF-CMV-Puro-COOH-GST were digested with NotI-HF and EcoRI-HF (NEB, Frankfurt, Germany) for 1 h at 37 °C and heat inactivated at 60 °C for 20 min. The vector and insert were ligated using the T4 ligase in a 1:3 (vector:insert) ratio and the ligated plasmids transformed into *E. coli* NEB10β. To confirm the successful integration of the insert, the isolated plasmids were digested using NotI-HF and EcoRI-HF for 1 h at 37 °C. The plasmids containing an insert were sequenced at Eurofins Scientific (Table S4) [45,46].

Subsequently, pSF-IGF1R-WT, pSF-IGF1R-N1129S and pSF-IGF1R-D1146N were amplified using specifically designed flanking primers (Table S3). The size-selected and gel-purified DNA was ligated with the NheI/NotI-digested vectors pT2-SVPuroCMV and pT2-SVPuroCAG (1:3). The *E. coli* NEB10β was then transformed with the ligated plasmids and plated on ampicillin^+^ LB-agar plates for the clonal selection. The plasmid minipreparations were sequenced to verify the correct insertion and the absence of undesired mutations within the IGF1R variants (Table S4) [45].

AMO-1, U-266, JJN-3 and the CRISPR-Cas9 IGF1R-knockout cell line L-363-B4 were transfected in 4 mm cuvettes by electroporation as previously published [47]. A total of 10 µg of IGF1R-expression vectors (i.e., pT2-SVPuroCAG-IGF1R-WT, pT2-SVPuroCAG-IGF1R-D1146N, pT2-SVPuroCAG-IGF1R-N1129S (AMO-1), pT2-SVPuroCMV-IGF1R-WT, pT2-SVPuroCMV-IGF1R-D1146N and pT2-SVPuroCMV-IGF1R-N1129S (L-363-B4, U-266 and JJN-3)), 2.5 µg expression vector for GFP (pmax-GFP) and 15 µg transposase expression vector (pCMV(CAT)T7-SB100-Transposase) were included in the respective electroporation mixtures. The transfection efficiency was assessed by flow cytometry using a BD FACSCanto II (BD Biosciences, Heidelberg, Germany). The electroporated cells were subjected to puromycin selection for 10 days (1 µg/mL (JJN-3, L-363 and U-266) and 1.5 µg/mL (AMO-1)). Successful overexpression was verified by comparing the IGF1R expression levels of the overexpression sublines with the EV-transfected sublines prior to any further analysis (Figure S1).

### 2.7. SDS-PAGE and Immunoblotting

Preparation of the whole cell lysates, SDS-PAGE and immunoblotting were performed as previously described [46] (for antibodies see Table 2). The Western blot images were evaluated by visual inspection and, where technically possible, the intensities calculated using Fiji (“Gels” tool). The intensities calculated for each marker were always normalized to those of the corresponding GAPDH bands. For details, see the figure legends. See the Supplementary Methods for the uncropped blot figures.

### 2.8. Viability and Proliferation Assays

*AlamarBlue assays:* Were performed to analyze the viability of the HMCLs overexpressing IGF1R^WT^, IGF1R^D1146N^ or IGF1R^N1129S^ under normal culturing conditions.

15 × 10^3^ cells/well were seeded in 96-well plates in quadruplicates and cultured overnight in 200 µL culture medium (for standard conditions). The following day, 20 µL AlamarBlue solution (and 20 ng/mL IGF1 for the stimulation experiments) was added to each well and the absorbance was measured at 570 nm and 600 nm after 24 h, 48 h and 72 h with a microtiter plate reader (FLUOstar Omega, BMG Labtech, Ortenberg, Germany). Quadruplicates of the medium alone and medium with AlamarBlue were used as the controls.

*MTT assays:* For the measurement of the metabolic activity/viability after treatment with linsitinib at distinct time points (24 h, 48 h, 72 h and 96 h), 8000 cells/100 µL (MM.1S, AMO-1, JJN-3, KMS-11 and L-363), 15,000 cells/100 µL (U-266) or 25,000 cells/100 µL (KMS-12-BM) were seeded in culture medium and 0.4 µM inhibitor or the adequate amount of DMSO (negative control) added stepwise at 0 h or after 24 h, 48 h and 72 h. Subsequently, 10 μL thiazolyl blue tetrazolium bromide was added to 100 µL cell suspension in the 96-well plates. After 4 h, 90 μL solubilization solution was added. Following overnight incubation, the absorbance of the reduced solubilized formazan was measured at 570 nm.

*IGF1R-knockout clone proliferation assays:* The L-363 (6 × 10^5^) and U-266 cells (1 × 10^6^) were seeded in T-25 cell culture flasks in 5 mL and 8 mL culture medium, respectively. Regarding L-363, the living cells were counted using a Neubauer counting chamber on days 3, 6, 8, 10 and 13. On day 3, 3 mL medium was added to the cell culture flask. The cells were split in a 1:4 ratio on day 6, 4 mL medium was added on day 8 and the culture was split again in a 1:3 ratio on day 10. The U-266 cells were counted on days 4, 7, 11, 14 and 17. On day 7, the culture was split in a 3:4 ratio and 2 mL medium was added on day 9, followed by a 1:2 split on day 11 and the addition of 2 mL medium on day 14.

### 2.9. Apoptosis and Survival Assay (Annexin-V/PI)

A total of 10,000–20,000 cells per well were seeded in 96-well plates and treated with either linsitinib, carfilzomib or both drugs for 3 days. Subsequently, the cells were collected, stained with annexin V-FITC and propidium iodide (PI) solution and measured according to a previously described flow cytometry protocol [47]. After an initial estimation of single-drug effects, the concentrations that would produce only minor effects were chosen for the complete combination experiments shown.

## 3. Results

### 3.1. Stimulation as Well as Attenuation of IGF1R Signaling Influence the Activation of IGF1R Effectors and the Proliferation Rate in HMCLs

All the HMCLs used in the current study expressed and activated IGF1R, with KMS-11 showing the highest and AMO-1 the lowest IGF1R levels (Figure S2), and IGF1R deprivation affected the AKT and/or MEK-ERK signaling in all the HMCLs (Figure S3), underlining previous findings [29,32,33,34,36,41,42,43].

**Figure 1:**
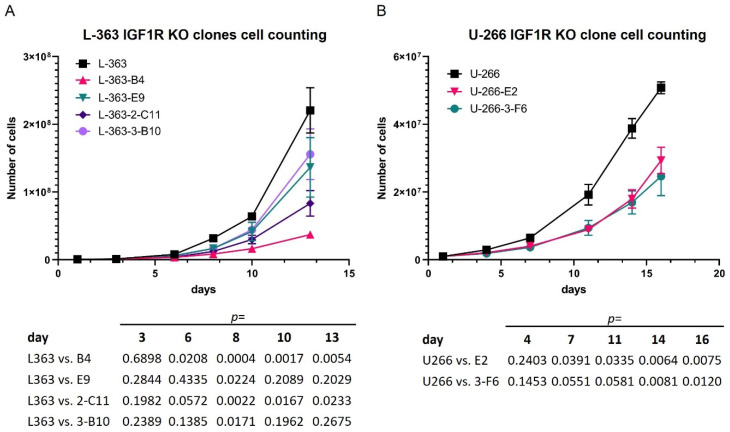
Proliferation curves of different IGF1R-knockout clones of the HMCL (**A**) L-363 (clones B4, and E9 (exon 18-targeted), 2-C11, 3-B10 (exon 2-targeted)) and (**B**) U-266 (clone 3-F6 (exon 2-targeted) and E2 (exon 18-targeted)) in comparison to the respective wild-type cells. All the results were revealed by at least three independent experiments. The statistical test was a two-tailed unpaired *t*-test. The *p* values are summarized below the curves.

Moreover, the proliferation was considerably reduced in the different CRISPR-Cas9 *IGF1R*-knockout clones (Figures S4 and S5) of both the L-363 (IGF1R^mut^) and U-266 (IGF1R^WT^) cells (Figure 1A,B), confirming the role of IGF1R as an important survival and proliferation factor in MM.

### 3.2. IGF1R Overexpression Impacts the Activation of MEK/ERK and AKT

**Figure 2:**
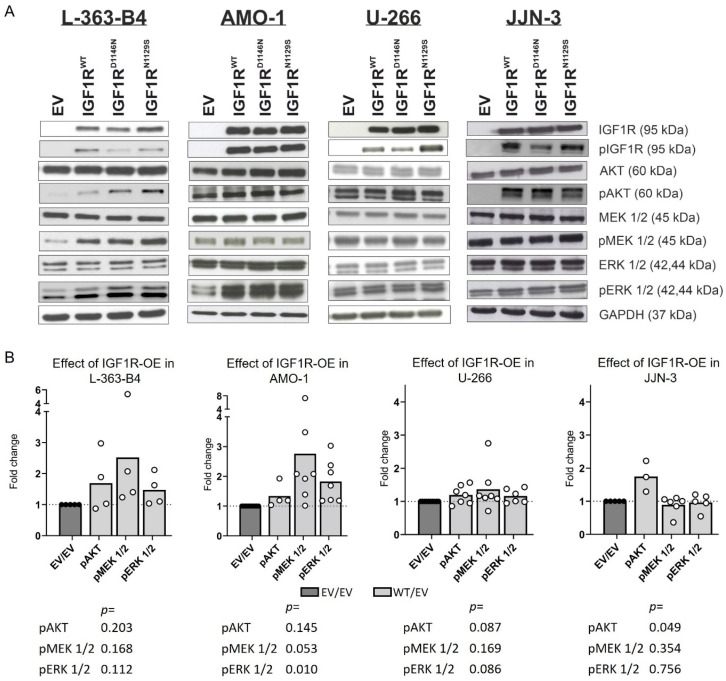
Investigation of the expression and activation status of potential IGF1R effectors by Western blot analysis in the *IGF1R* KO subline L-363-B4 and regular AMO-1, U-266 and JJN-3 cells transfected with either empty vector (EV), IGF1R^WT^, IGF1R^D1146N^ or IGF1R^N1129S^. (**A**) The blots shown are representative for at least three independent rounds, and part of the blots for L363-B4 are also depicted in [45]. Different effectors were assessed on separate blots or parts of the blots. (**B**) The signal intensities were calculated by Fiji to assess the effect of IGF1R^WT^ overexpression (OE) compared to the EV. It was not assessable for IGF1R due to strong differences in the expression levels between the EV and overexpression lines (see Figure S1). The intensities were normalized to the corresponding GAPDH signal detected on the same membrane and, subsequently, the signals for the WT were normalized to the EV (WT/EV). The statistical test was a two-tailed unpaired *t*-test.

Because IGF1R depletion affected the RTK signaling (especially AKT activation) and proliferation in all the HMCLs investigated, we next studied the effects of IGF1R overexpression in three IGF1R^WT^ HMCLs (AMO-1, U-266 and JJN-3) and one IGF1R^mut^ HMCL (L-363) by Western blotting (Figure 2A). Due to the intrinsic occurrence of an *IGF1R* mutation, we used the *IGF1R*-knockout cell line L-363-B4 instead of the parental cell line L-363.

Overexpression of IGF1R^WT^ resulted in the increased activation of AKT and/or MEK/ERK signaling in normal cell culture conditions in all the HMCLs (slight effects, especially in U-266) (Figure 2A,B) but had no effect on viability (Figure S6).

### 3.3. Mutations Can Impact IGF1R Activation and/or Downstream Signaling

RTK mutations can be a decisive factor in tumor progression and targeted treatment approaches [20,23,48,49,50] and are associated with inferior survival in MM. Therefore, we investigated whether the L-363 mutation IGF1R^D1146N^ or the MM patient-derived mutation IGF1R^N1129S^ (for details, see Section 2 and Table S1) alter the activation of the classical RTK effectors.

**Figure 3:**
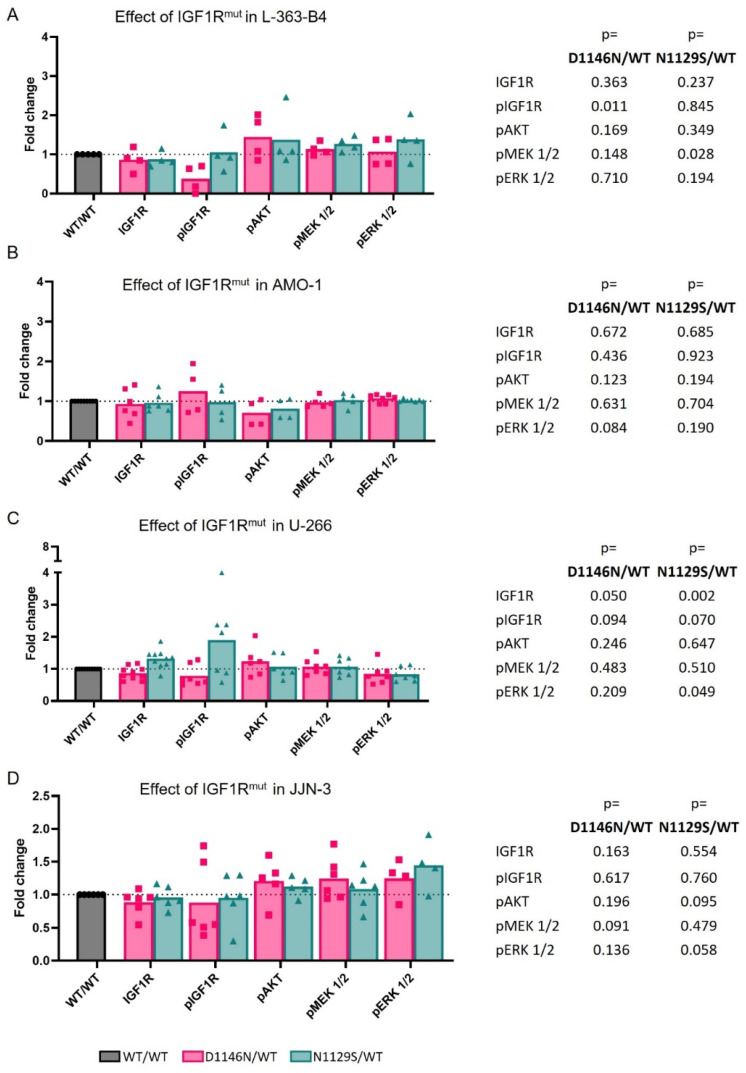
The effect of the two IGF1R mutations D1146N and N1129S on the expression and activation of IGF1R and on the activation status of the different classical RTK effectors compared to IGF1R^WT^ under normal culturing conditions in L-363-B4 (**A**), AMO-1 (**B**), U-266 (**C**) and JJN-3 (**D**). For a representative blot, see Figure 2A. For the calculation of the intensity values of each single marker, the GAPDH intensity, detected on the same membrane, served as the reference. To depict the effect of the mutation, the GAPDH normalized values of the mutants D1146N and N1129S were divided by the values of the WT for each respective marker. The statistical test was a two-tailed unpaired *t*-test.

**Figure 4:**
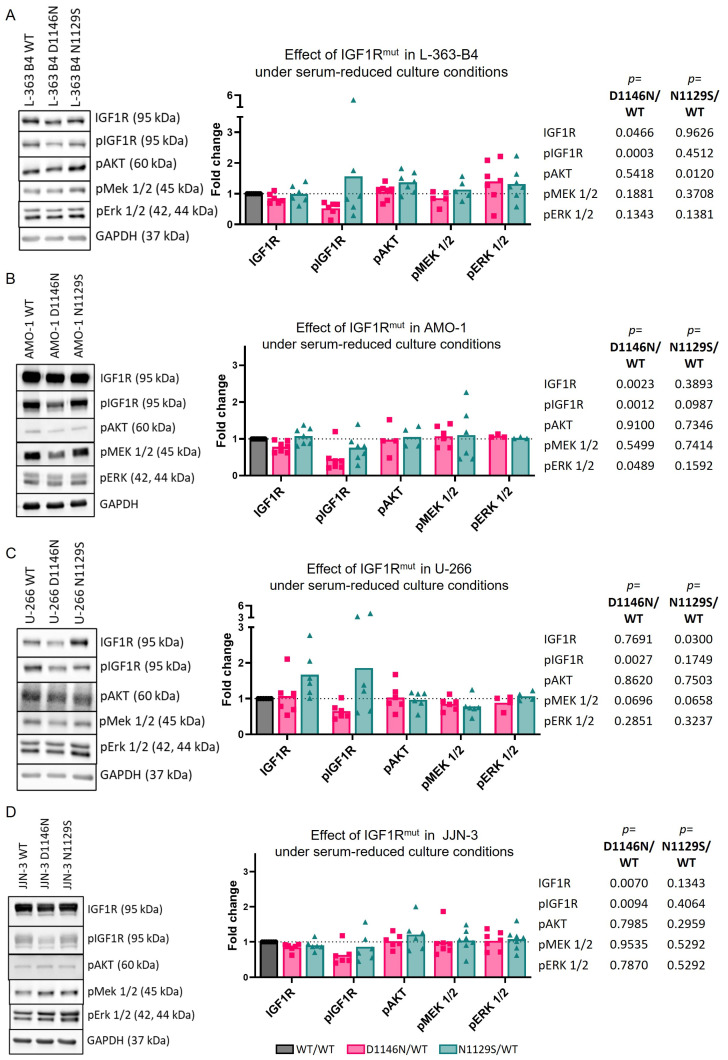
The effect of mutations under serum-reduced conditions (starved) in L-363-B4 (**A**), AMO-1 (**B**), U-266 (**C**) and JJN-3 (**D**). A representative Western blot from a minimum of three independent experiments is shown. The intensities calculated for each marker were normalized to those of the corresponding GAPDH bands followed by the normalization of the intensities calculated for the cell lines overexpressing mutant IGF1R to those of the respective cell lines overexpressing IGF1R^WT^. The statistical test was a two-tailed unpaired *t*-test.

Comparing the HMCLs overexpressing IGF1R^D1146N^ with IGF1R^WT^, a significant reduction in pIGF1R Y1135 was observed in L-363-B4 under normal culturing conditions and in all four IGF1R overexpression HMCL models under serum reduction (Figure 3A and Figure 4A–D). However, this inactivation did not translate to changes in the AKT and MEK/ERK signaling. Comparing IGF1R^N1129S^ with IGF1R^WT^ under standard culturing conditions, a slight but significantly higher MEK activation (*p* = 0.028) and a higher activation of ERK was observed in L-363-B4-overexpressing IGF1R^N1129S^ in three out of four independent experiments (Figure 3A). Moreover, there also seemed to be a tendency for higher AKT activation upon the expression of mutant IGF1R (Figure 3A).

Notably, similar observations were made when L363-B4 was kept in low-FBS (0.5%) culture (Figure 4A). However, the expression of mutant IGF1R did not impact the viability (Figure S6). The effect of overexpression of mutant IGF1R on downstream effectors in the other HMCLs under normal culturing conditions and in serum-reduced medium was inconclusive (Figure 3B–D and Figure 4B–D).

### 3.4. Linsitinib Affects AKT Signaling, Viability and Survival in All HMCLs

**Figure 5:**
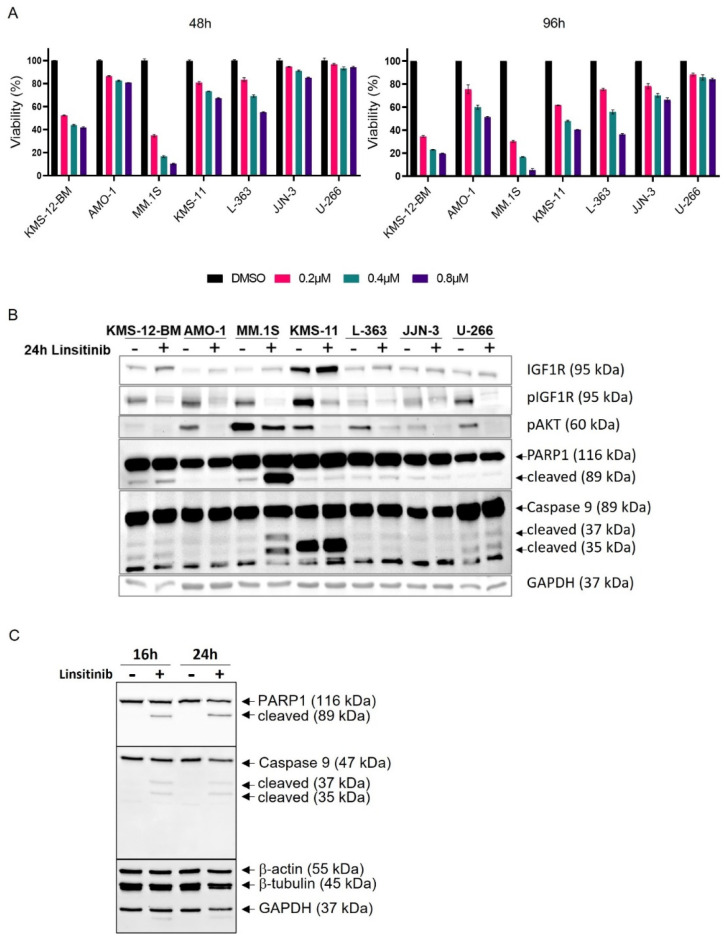
Treatment of HMCLs with linsitinib (Lin). Effect on metabolism and signaling was assessed using (**A**) MTT-assay and (**B**,**C**) Western blot analysis. MTT assays after 48, 72 and 96 h were performed in technical triplicates (mean ± SEM) (for 72 h values, see Figure S7). Viability is relative to DMSO control. (**C**) PARP-1 and caspase 9 cleavage in MM.1S after 16 h and 24 h. Different proteins were assessed on separate parts of the same blot or on different blots.

Treatment with linsitinib affected the viability of all the HMCLs except for U-266 in a dose- and time-dependent manner within a concentration range of 0.2–0.8 µM. Notably, the U-266 cells remained virtually unaffected after 48 h and their viability was only minimally reduced after 72 h and 96 h of treatment (Figure 5A and Figure S7). Because 0.2 µM linsitinib only had a moderate impact on the viability of the AMO-1, JJN-3 and L-363 cells, while the MM.1S cells appeared to be particularly sensitive with maximal drug effects already visible at 48 h, we chose a concentration of 0.4 µM for the subsequent experiments.

**Figure 6:**
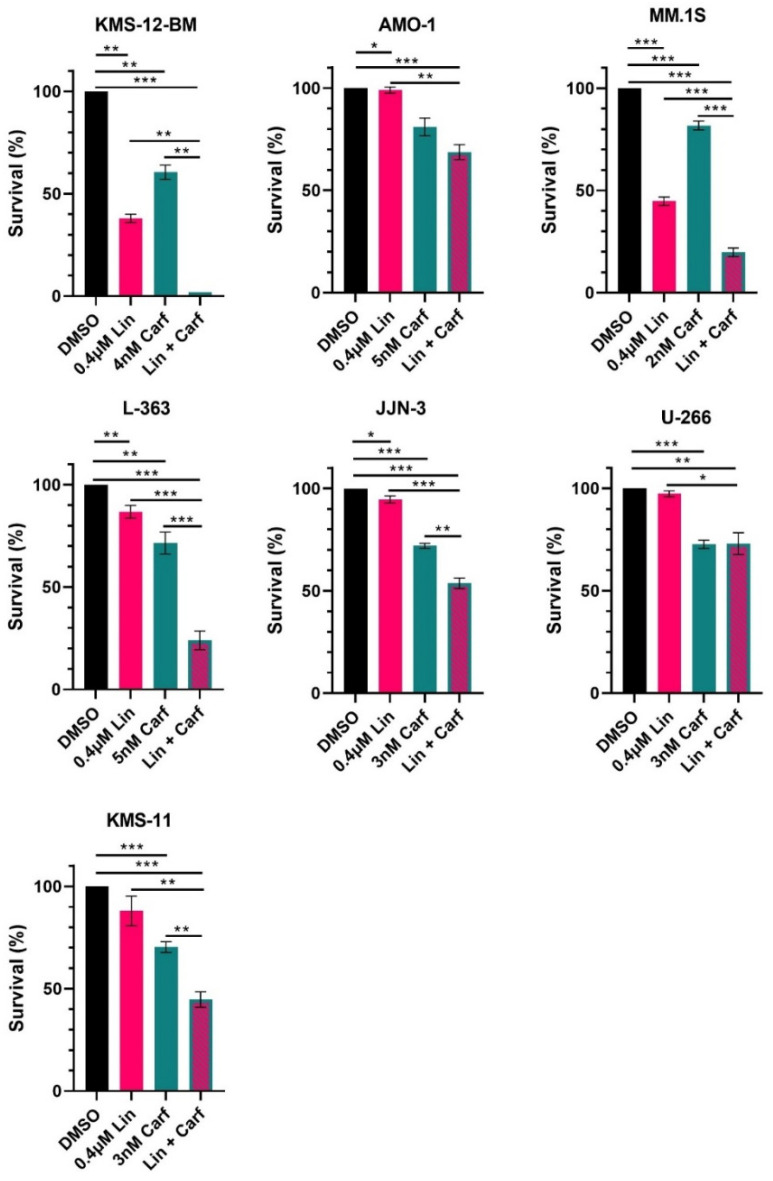
Treatment of 7 HMCLs with linsitinib (Lin) alone and in combination with carfilzomib (Carf) for 72 h. Effect on survival was determined by annexin-V/PI staining in three independent experiments except for KMS-12-BM (two experiments). Data shown is survival relative to DMSO control (mean ± SEM). Statistical test was a two-tailed unpaired *t*-test. * for *p* < 0.05; ** for *p* < 0.01; and *** for *p* < 0.001.

At 0.4 µM linsitinib, a clear reduction, although not complete extinction, of the pIGF1R and pAKT signals was observed for all seven HMCLs tested (Figure 5B). The annexin-V/PI staining (Figure 6) showed that incubation with 0.4 µM linsitinib for 72 h significantly reduced the survival of the MM.1S (IGF1R^WT^) and KMS-12-BM (IGF1R^WT^) cells, while the survival of the other five HMCLs was clearly less affected (L-363 (IGF1R^mut^), JJN-3 (IGF1R^WT^) and KMS-11 (IGF1R^WT^)) or even unaffected (AMO-1 (IGF1R^WT^) and U-266 (IGF1R^WT^)).

Increased PARP1 and caspase 9 cleavage following linsitinib treatment was only observed for MM.1S in the independent experiments (Figure 5B,C).

### 3.5. Combined Treatment with Linsitinib and Carfilzomib Is Effective in HMCL with and without IGF1R Mutations

Previous studies have concluded that single-agent treatments with IGF1R inhibitors (e.g., linsitinib) are not effective [37,38,39] and the intrinsic *IGF1R* mutation in L-363 did not correlate with an improved response of the L-363 cells to linsitinib alone compared to the IGF1R^WT^ HMCLs (Figure 6). A combination of linsitinib with the first-generation PI bortezomib has shown promising results in patients [31]; however, bortezomib was reported to be inhibited by IGF1R expression [40]. For this reason and given that HMCLs rather represent relapsed and refractory MM, we chose the second-generation PI carfilzomib, commonly used for relapsed and refractory MM [44], for our investigations.

Initially, the carfilzomib sensitivity of each HMCL was determined in order to identify concentrations with little efficacy (i.e., 10–40% cell death after 3 days) to allow for the assessment of the combination effects with linsitinib. As expected from the single-agent inhibition experiment, linsitinib did not add to the effect of carfilzomib in U-266 and AMO-1 (Figure 6). Linsitinib in combination with carfilzomib had, however, a significantly stronger effect on survival in all the other HMCLs (KMS-12-BM, MM.1S, L-363, JJN-3 and KMS-11), which neither seemed to correlate with the level of IGF1R expression (see Figure S2) nor the presence of an IGF1R mutation (Figure 6).

## 4. Discussion

Many cancers depend at least to some extent on oncogenic growth and survival signaling from RTKs. These are frequently affected by genetic alterations such as SNVs, although with greatly variable significance and prevalence between and within specific tumor types [21,22,23,24,25,42,48,51]. However, even under circumstances where only a rather small subgroup of patients might benefit from a particular anti-RTK therapy, such an approach can be warranted, if the responsive patient population can be confidently identified.

Our previous analysis of SNVs and rare patient-specific single nucleotide polymorphisms in MM patients demonstrated that IGF1R is one of the most frequently affected RTK genes and that RTK mutations are associated with inferior survival [26,27], supporting the role of IGF1R as a bad prognostic marker in MM [28]. Although previous publications have investigated the effects of IGF1 and IGF1R blockade on a molecular level in MM [29,32,33,34,36,41,42,43], this study is the first to investigate the functional consequences of IGF1R overexpression and the impact of *IGF1R* mutations. Given the high genetic heterogeneity of MM [8,10,52] and the potential of IGF1R for oncogenic activity through the formation of hybrid receptors as well as through activating mutations, we chose HMCLs that represent different molecular aspects with relevance to IGF1R signaling, such as intrinsic AKT activity, AKT dependence [16,53] and the mutation pattern [10]. Notably, we included the HMCL L-363, which carries an intrinsic *IGF1R* missense mutation (D1146N) [10].

Consistent with previous findings [29,32,33,34,36,41,42,43], the HMCLs investigated in the current study all responded to IGF1 stimulation, IGF1R-specific knockdown and pIGF1R/pINSR inhibition by linsitinib, with either increased or decreased levels of the survival marker pAKT. Of note, survival was hardly affected by the selective blockade of pIGF1R/pINSR using linsitinib in CD45^+^ HMCLs (e.g., U-266), confirming previous findings [33,34]. However, decreased proliferation was observed upon the CRISPR-Cas9 knockout of *IGF1R* in several single-cell clones of both the *IGF1R*-WT and CD45+ HMCL U-266 and the *IGF1R*-mutant HMCL L-363, supporting the observation that MM depends on IGF1R and that IGF1R is preferentially essential for MM compared to other neoplasias [42]. Although the reduction in proliferation did not seem to be dependent on the mutation status, it might still be interesting to test inhibitors that not “only” target pIGF1R but also IGF1R expression, especially in pIGF1R inhibition-resistant CD45^+^ MM [33,34].

In agreement with previous results [35], IGF1R expression did, however, not seem to be a suitable biomarker to discriminate between HMCLs responding to IGF1R inhibition in the current study. The sensitivity to linsitinib might thus be influenced by other parameters such as AKT activation levels or mutant KRAS, which were shown to be associated with the response to, for example, AKT inhibition [16,53]. This theory fits well with the behavior of MM.1S cells (KRAS^mut^, AKT-dependent) [16,46], which showed the strongest response with respect to metabolism and survival. However, there were notable exceptions, e.g., the fairly strong responses of the KMS-12-BM cells (KRAS^WT^, very low pAKT) and very little effect on the survival of AMO-1 cells (KRAS^mut^, low-to-moderate pAKT levels) [35].

Overexpression of IGF1R had the opposite effect on RTK signaling as IGF1R knockdown, though the effect seemed to be more moderate and did not result in increased viability. In comparison to the overexpression of IGF1R^WT^, overexpression of IGF1R^N1129S^ led to a slightly increased activation of potential IGF1R effectors (MEK, ERK and/or AKT) in L-363-B4 cells under normal culturing conditions but also in serum-reduced medium. This suggests that IGF1R^N1129S^ may be activated independently from external stimuli in this HMCL. However, the observed effects for mutant IGF1R were subtle in the L-363-B4 cells and rather inconclusive in the other three IGF1R-overexpressing HMCL models. Expression of mutant IGF1R in four different HMCL models also did not translate into increased viability. The slight and inconclusive effect of *IGF1R* mutations on survival signaling and the missing impact on viability might be due to the high number of mutations in important oncogenes within the HMCLs, including other RTKs and RTK effectors, which might compensate the effect of the *IGF1R* mutation (e.g., NRAS mutation in L-363 and BRAF mutation in U-266) [10]. Notably, the mutation IGF1R^D1146N^ displayed a significant decrease in the phosphorylation of the residue Y1135—located in the activation loop of IGF1R—in all four IGF1R overexpression HMCL models under serum-reduced conditions. However, this inactivation did not translate to changes in AKT and MEK/ERK signaling. Interestingly, previous investigations demonstrated that phosphorylation of the IGF1R is necessary for its ubiquitination and proteasomal degradation [54]. At this point, it remains unclear if the IGF1R mutation D1146N in L363 has an impact on this mechanism or if the loss of IGF1R activation is compensated for by other upstream regulators.

Moreover, this study showed that a combination of linsitinib and the second-generation PI carfilzomib, which is commonly used in relapsed/refractory MM and which, to our knowledge, has not been investigated in functional or clinical studies in the context of IGF1R so far, was effective in six out of seven HMCLs. However, no clear correlation between the level of response and the *IGF1R* mutation status was found. This might indicate that, for MM patients potentially responsive to IGF1R blockade, a combination with carfilzomib might be suitable to achieve longer and/or deeper remissions.

Of note, it has been previously shown that increased IGF1R expression and the activation of its signaling system contribute to acquired bortezomib resistance in MM cell lines, which were progressively adapted to withstand high bortezomib concentrations [40]. IGF1R suppression re-sensitized such cells to bortezomib [40]. Although the mechanisms and temporal patterns of PI resistance may vary, not just for different chemical compounds but also between preclinical and clinical settings, it is still worth mentioning that we did not observe that the comparatively high IGF1R expression or activation levels of the MM.1S and KMS-12-BM cells implicated any resilience against carfilzomib. These cells were fully sensitive to treatment with carfilzomib, indicating its potential suitability for combination with linsitinib in MM patients, irrespective of the IGF1R expression level and the *IGF1R* mutation status.

## 5. Conclusions

Our studies in four HMCL models overexpressing WT and mutant IGF1R showed that *IGF1R* mutations can affect the phosphorylation status of IGF1R (D1146N) and slightly increase survival signaling (N1129S), although they did not impact viability of the affected cells. However, the combination treatment of linsitinib and carfilzomib effectively enhanced MM cell death in six out of seven HMCLs. Even though this effect was not correlated with the strength of IGF1R expression or with the *IGF1R* mutation status, it warrants further clinical testing.

## Figures and Tables

**Table 1:** Primers used for Sanger sequencing of IGF1R KO clones.

Primer	Sequence
*IGF1R* Exon 18 F	5′ CATAAACAACCCACGGTGCC 3′
*IGF1R* Exon 18 R	5′ AAGGAGTCCGTGCACTCAAG 3′
*IGF1R* Exon 2 F	5′ GACATCCGCAACGACTATCA 3′
*IGF1R* Exon 2 R	5′ TTCTCACACATCGGCTTCTC 3′

**Table 2:** Antibodies used in Western blotting.

Table	Catalogue Numbers#	Dilution	Company
IGF1R	#9750	1:1000	Cell Signaling Technology (CST) (Danvers, MA, USA)
pIGF1R (Y1135)	#3918	1:1000	CST
pPYK2 (Y402)	#3291	1:2000	CST
AKT	#4691	1:2000	CST
pAKT (S473)	#4058	1:2000	CST
MEK1/2	#9122	1:8000	CST
pMEK1/2 (S217/221)	#9154	1:4000	CST
ERK1/2	#9102	1:4000	CST
pERK1/2 (T202/Y204)	#9101	1:4000	CST
CASP9	#9508	1:1000	CST
PARP	#9532	1:1000	CST
beta-tubulin	#2146	1:7500	CST
beta-actin	#4970	1:16,000	CST
GAPDH	#5174	1:100,000	CST
HRP-linked anti-rabbit	#7074	1:1000–1:3000	CST

## Data Availability

The raw data supporting the conclusions of this article will be made available by the authors on request.

## Supplementary Materials

The following supporting information can be downloaded at: https://www.mdpi.com/article/10.3390/cancers16112139/s1, Figure S1: Verification of IGF1RWT and IGF1Rmut (D1146N and N1129S) overexpression in stably transfected AMO-1 (A), U-266 (B), JJN-3 (C) and L-363 B4 (D). GAPDH was detected on the same blot as IGF1R. EV: empty vector-transfected control; Figure S2: (A) Investigation of the expression and activation status of IGF1R under normal culturing conditions. Intensities revealed with the Fiji tool were calculated for each marker by normalizing it to those of the corresponding GAPDH bands. Data shown are mean of three independent experiments. (B) Expression and activation status of IGF1R and activation status of AKT, MEK and ERK before (serum-reduced (0.5% FBS)) and after stimulation with IGF1. Western blot shown is representative for three independent experiments; Figure S3: Investigation of the expression and activation status of potential IGF1R effectors by Western analysis in 7 HMCLs after siRNA-mediated IGF1R knockdown. (A) The blot shown is representative of three independent experiments. (B) In addition, the signal intensities as revealed by Western blot analyses in the siRNA knockdown experiments were calculated for all repetitions using Fiji (“Gels” tool) and depicted as a bar diagram. Intensities revealed with the Fiji tool were calculated for each marker by normalizing it to those of the corresponding GAPDH bands and the intensities of the siRNA-treated samples were subsequently normalized to those of the corresponding scrRNA-treated samples. Cells transfected with scrambled siRNA (scrRNA) served as negative controls. Knockdowns were performed in at least three independent experiments. Each data point represents one independent experiment. The signal intensities for pERK in KMS-12-BM were too low for calculation with Fiji (n.e.); Figure S4: Overview of L-363 CRISPR-Cas9 IGF1R-knockout clones. (A) Comparison of IGF1R sequence in IGF1R KO clones at the sgRNA target site (Exon 18 KO). Figure adapted from Ref. [45]. (B) Overview of IGF1R expression in all KO clones used for experiments (Exon 18 and Exon 2 KO clones). (C) Indirect immunofluorescence (IF). For IF, cells were stained with DAPI (CST (#4412); 1:1000). IGF1R [alphaR3] (Genetex mouse; 1:50) and goat/anti-mouse-Alexa-Fluor 647 (ThermoFisher (A32728), 1:400). Images were scanned using either a Zeiss Elyra S.1- structural imaging microscope (63× oil) and a S-CMOS-Camera: PCO Edge 5.5 or using a Leica SP2 confocal laser scanning microscope (40× oil). Analysis was performed using ZEN lite and Fiji; Figure S5: Overview of U-266 CRISPR-Cas9 IGF1R-knockout clones. (A) Comparison of IGF1R sequence in IGF1R KO clones at the sgRNA target site (Exon 18 KO). Figure adapted from Ref. [40]. (B) Overview of IGF1R expression in all KO clones used for experiments (Exon 18 and Exon 2 KO clones). B3 is a WT single-cell clone. (C) Indirect immunofluorescence (IF). For IF, cells were stained with DAPI (CST (#4412); 1:1000). IGF1R [alphaR3] (Genetex mouse; 1:50) and goat/anti-mouse-Alexa-Fluor 647 (ThermoFisher (A32728), 1:400). Images were scanned using a Zeiss Elyra S.1- structural imaging microscope (63× oil). Analysis was performed using ZEN lite and Fiji; Figure S6: Viability assay (AlamarBlue) of IGF1R-overexpressing cells under normal culturing conditions. Results were derived from three independent experiments (mean ± SEM); Figure S7: Treatment of MM cell lines with different concentrations of linsitinib and subsequent measurement of the effects on metabolism using an MTT assay after 72h incubation (mean ± SEM); Table S1: SeattleSeq annotation was used to annotate the IGF1R mutations D1146N and N1129S to the reference genome hg19. The bioinformatic predictors PhastCons and GERP predict the level of conservation and PolyPhen2 predicts structural changes; Table S2: Primers used for mutagenesis PCR; Table S3: Primers used for cloning of expression and donor vectors; Table S4: Primer used for Sanger sequencing of ligated plasmids.

## Abbreviations

Insulin-like growth factor 1 receptor	IGF1R
Multiple myeloma	MM
Human multiple myeloma cell lines	HMCLs
Receptor tyrosine kinases	RTKs
Epidermal growth factor receptor	EGFR
Fibroblast growth factor receptor 3	FGFR3
erb-b2 receptor tyrosine kinase 2	ERBB2
Single nucleotide variants	SNVs
Single nucleotide polymorphisms	SNPs
