# Supplementary material for: Functional Investigation of IGF1R Mutations in Multiple Myeloma
Source: Cancers (Basel). 2024 Jun 4;16(11):2139. doi: 10.3390/cancers16112139 (PMC11171363; doi:10.3390/cancers16112139)

Figure S1

Verification of $IGF1R^{WT}$ and $IGF1R^{mut}$ (D1146N and N1129S) overexpression in stably transfected AMO-1 (A), U-266 (B), JJN-3 (C) and L-363 B4 (D). GAPDH was detected on the same blot as IGF1R. EV: empty vector transfected control.

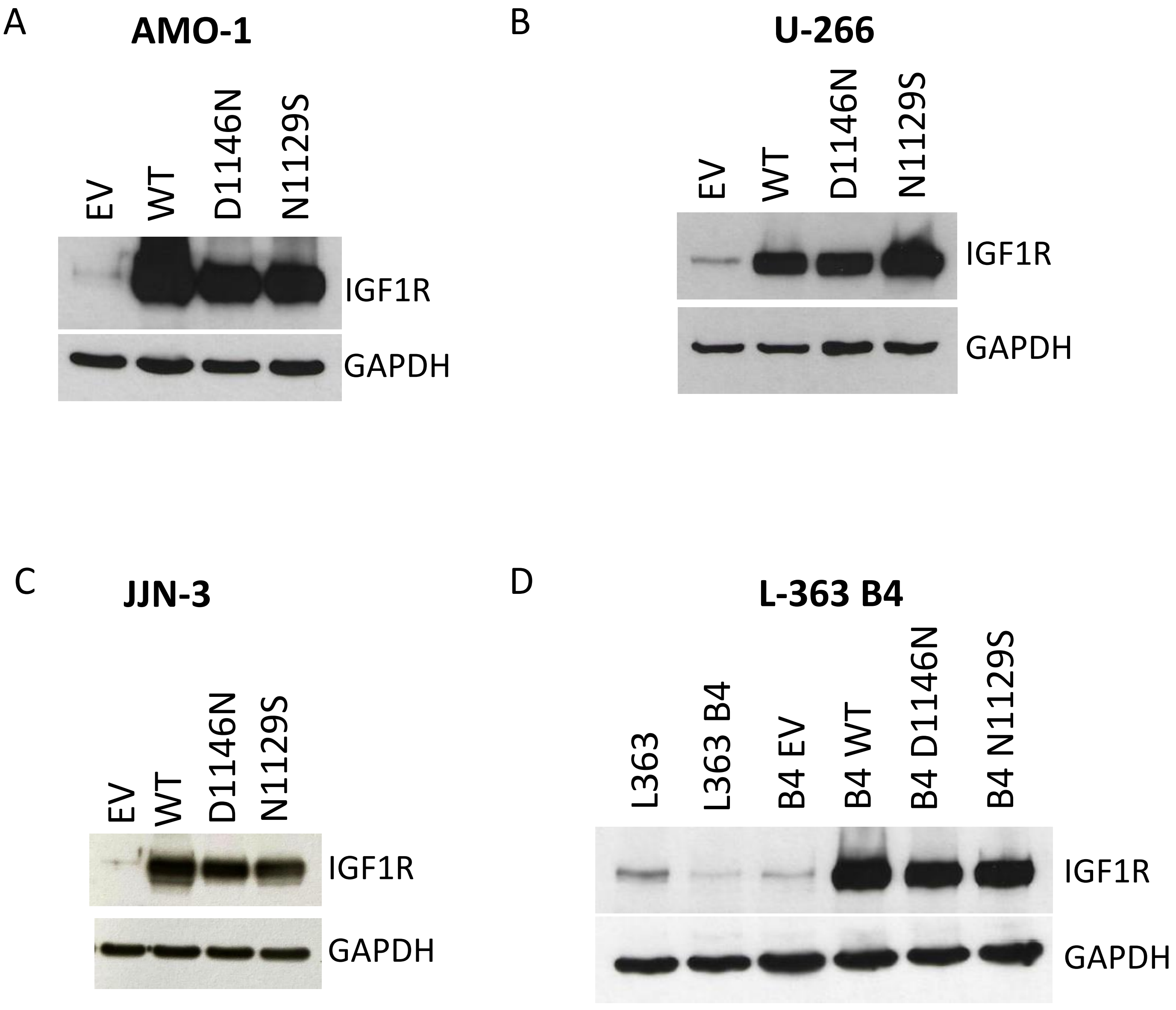

Figure S2

(**A**) Investigation of the expression and activation status of IGF1R under normal culturing conditions. Intensities revealed with the Fiji tool were calculated for each marker by normalizing it to those of the corresponding GAPDH bands. Data shown is mean of three independent experiments. (**B**) Expression and activation status of IGF1R and activation status of AKT, MEK and ERK before (serum-reduced (0.5% FBS)) and after stimulation with IGF1. Western blot shown is representative for three independent experiments.

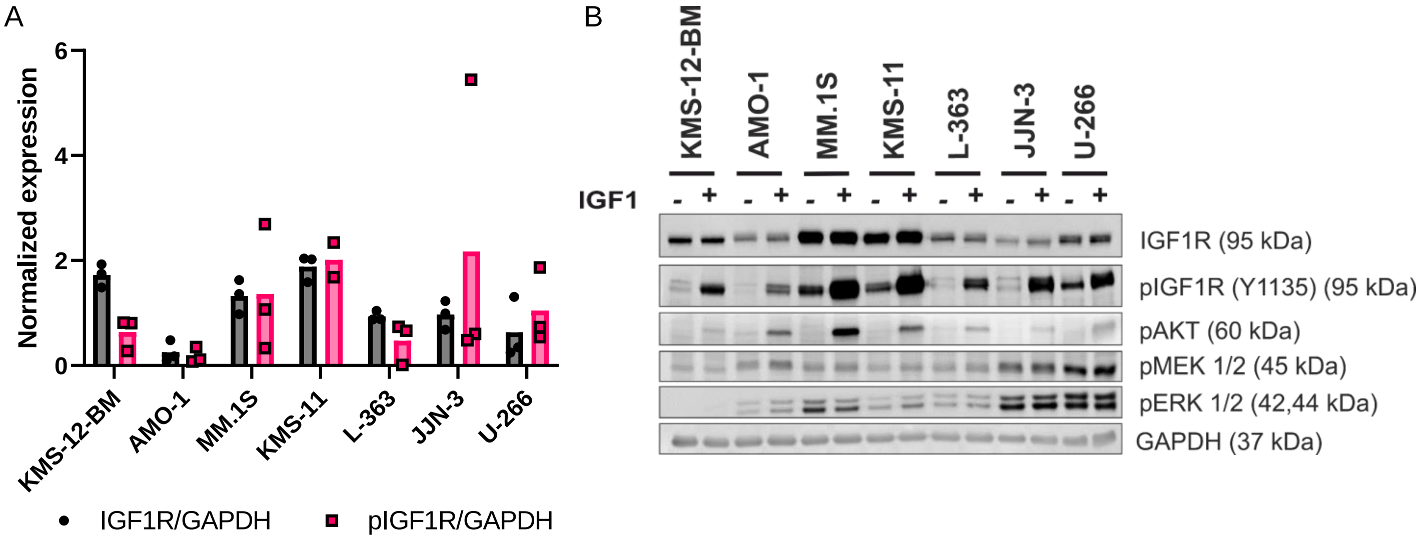

Figure S3

Investigation of the expression and activation status of potential IGF1R-effectors by Western analysis in 7 HMCLs after siRNA-mediated IGF1R-knockdown. (**A**) The blot shown is representative for three independent experiments. (**B**) In addition, the signal intensities as revealed by Western blot analyses in the siRNA knockdown experiments, were calculated for all repetitions using Fiji (“Gels” tool) and depicted as a bar diagram. Intensities revealed with the Fiji tool were calculated for each marker by normalizing it to those of the corresponding GAPDH bands and the intensities of the siRNA-treated samples were subsequently normalized to those of the corresponding scrRNA-treated samples. Cells transfected with scrambled siRNA (scrRNA) served as negative controls. Knockdowns were performed in at least three independent experiments. Each data point represents one independent experiment. The signal intensities for pERK in KMS-12-BM were too low for calculation with Fiji (n.e.).

A

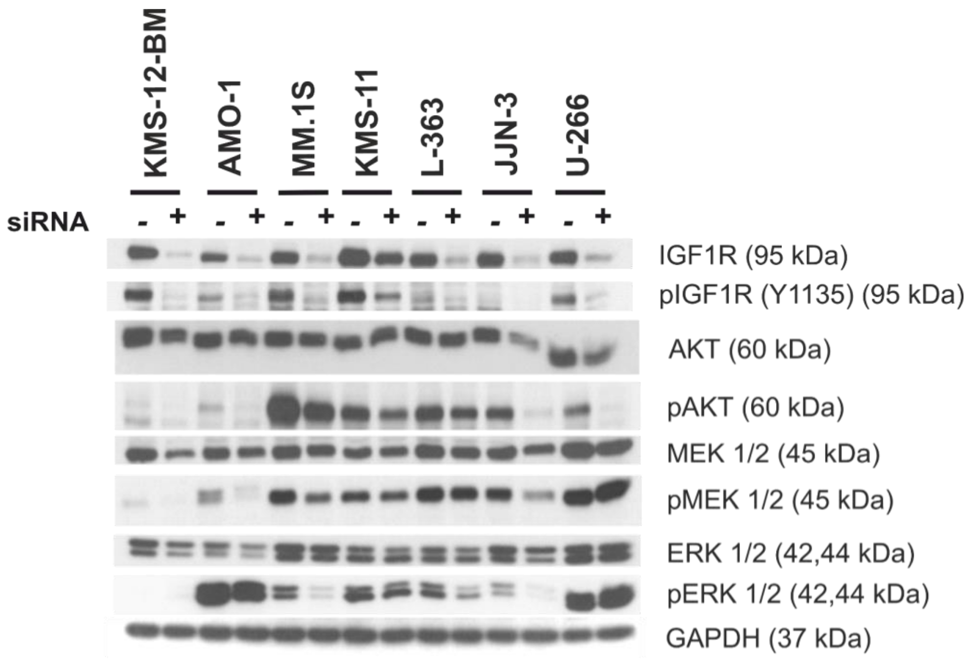

B

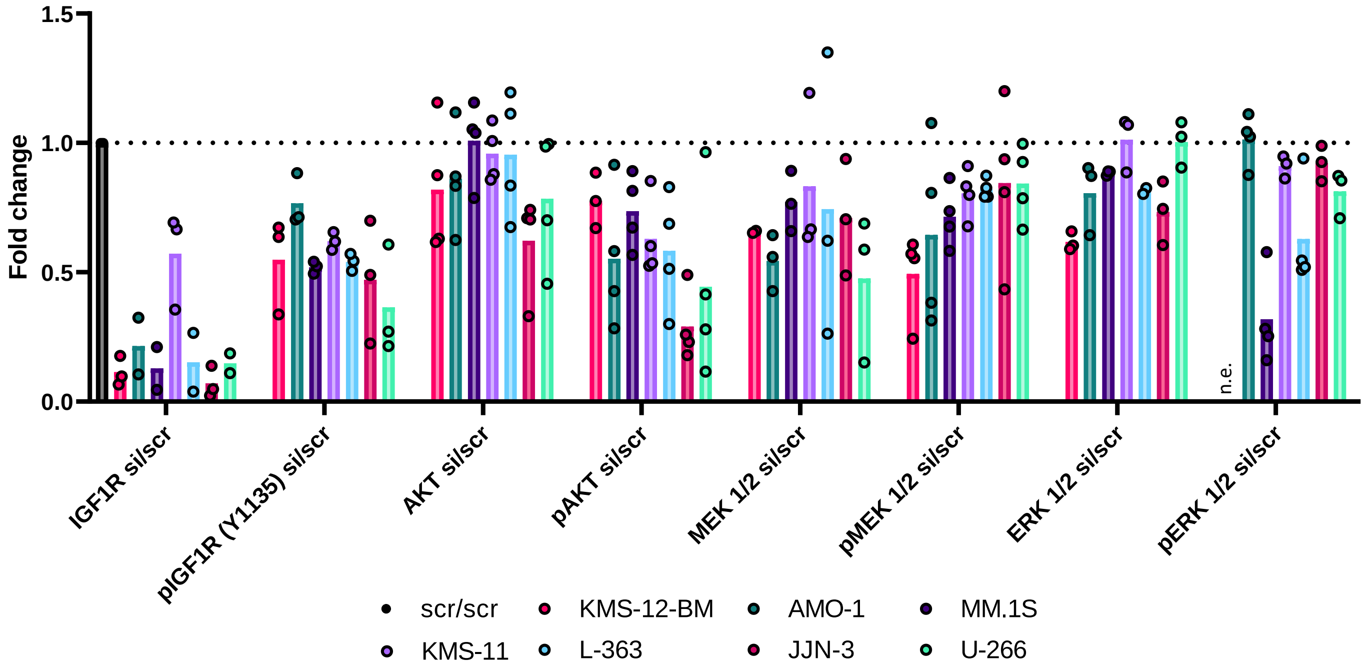

Figure S4 Part I Overview of L-363 CRISPR-Cas9 IGF1R knockout clones. (**A**) Comparison of IGF1R sequence in IGF1R KO clones at the sgRNA target site (Exon 18 KO). Figure adapted from Ref[45].

A

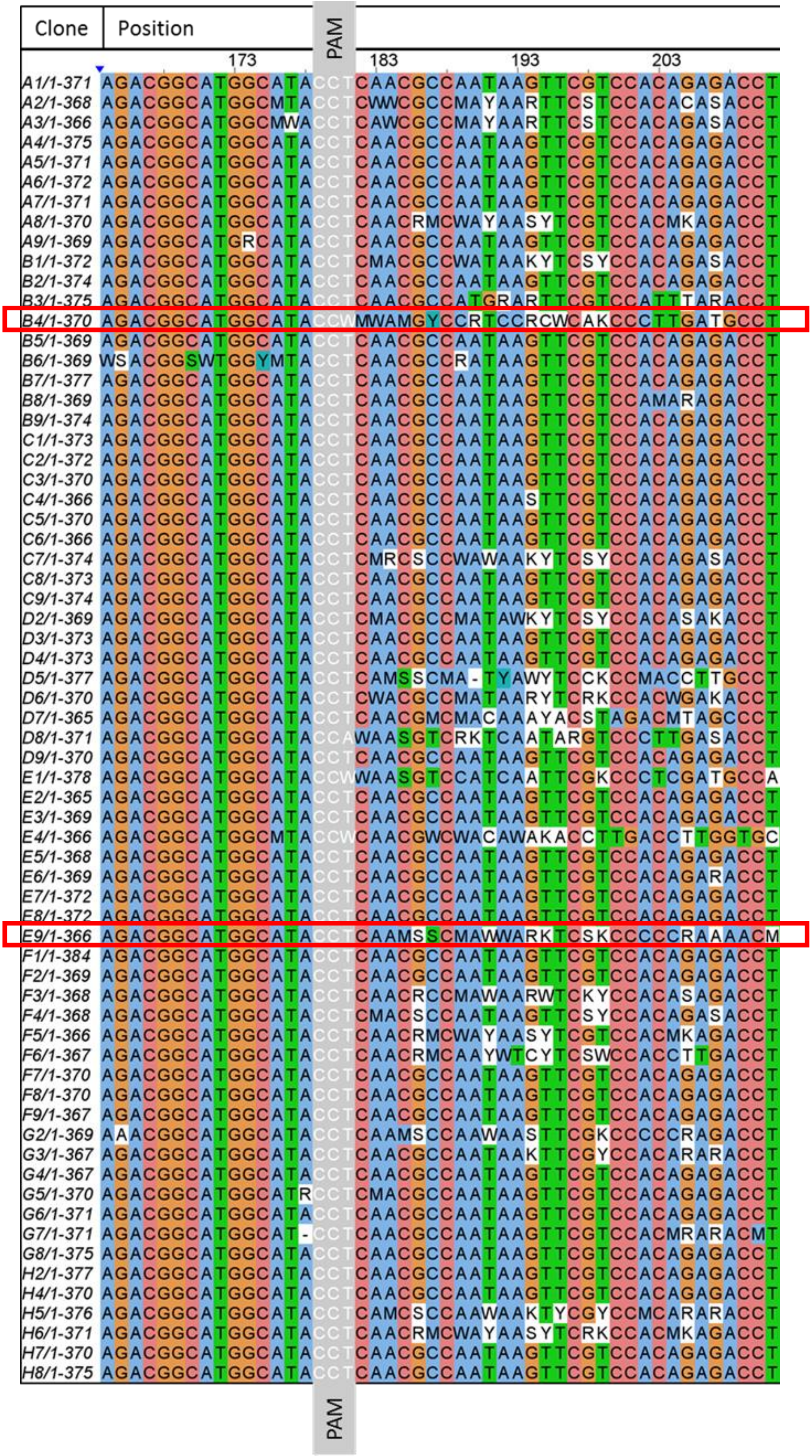

Figure S4 Part II Overview of L-363 CRISPR-Cas9 IGF1R knockout clones. (**B**) Overview of IGF1R expression in all KO clones used for experiments (Exon 18 and Exon 2 KO clones). (**C**) Indirect immunofluorescence (IF) (not shown for B3). For IF, cells were stained with DAPI (CST (#4412); 1:1000). IGF1R [alphaR3] (Genetex mouse; 1:50) and goat/anti-mouse-Alexa-Fluor 647, (ThermoFisher (A32728), 1:400). Images were scanned using either a Zeiss Elyra S.1- structural imaging microscope (63x oil) and a S-CMOS-Camera: PCO Edge 5.5 or using a Leica SP2 confocal laser scanning microscope (40x oil). Analysis was performed using ZEN lite and Fiji.

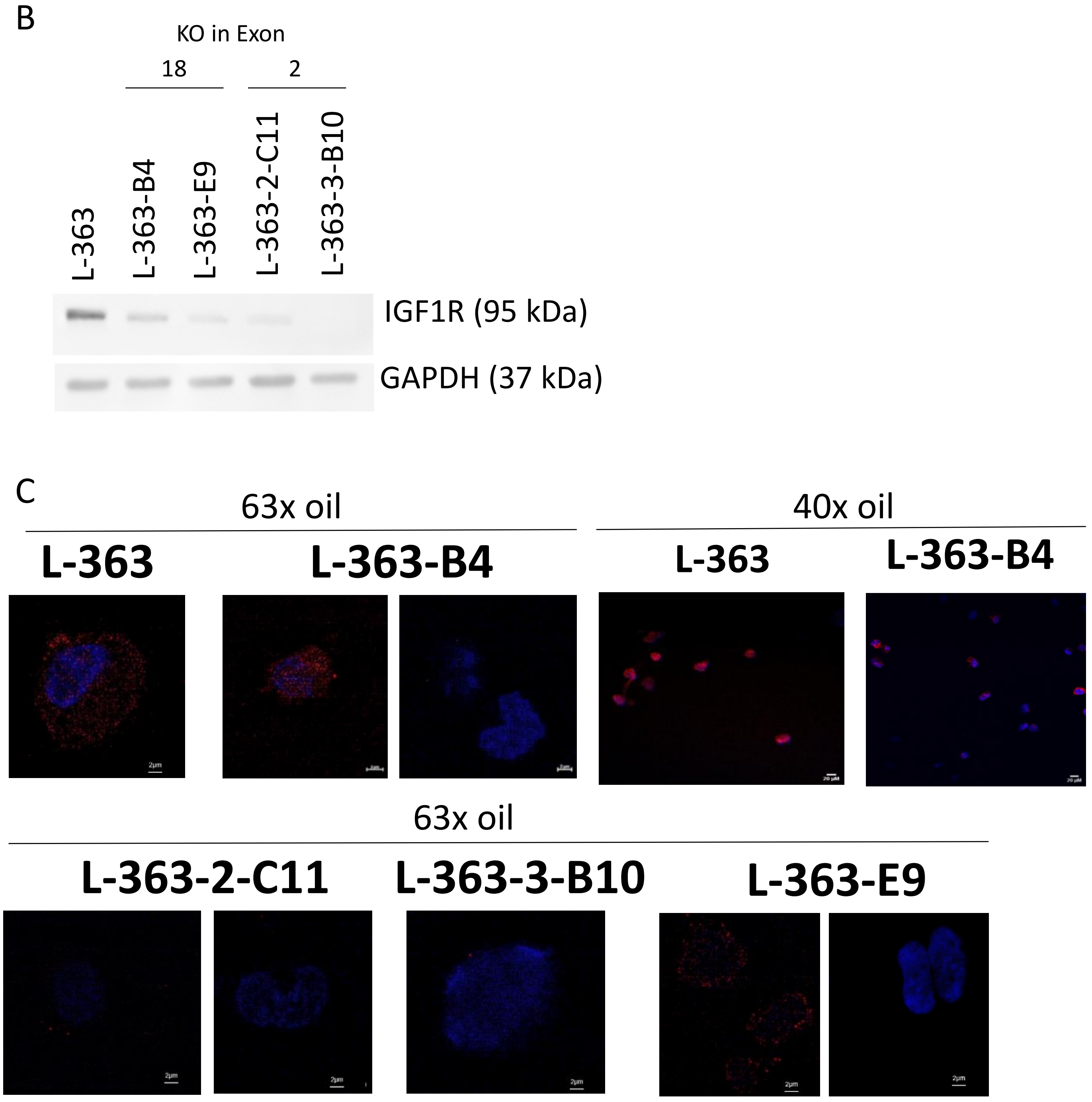

Figure S5 Overview of U-266 CRISPR-Cas9 IGF1R knockout clones. (**A**) Comparison of IGF1R sequence in IGF1R KO clones at the sgRNA target site (Exon 18 KO). Figure adapted from Ref[40]. (**B**) Overview of IGF1R expression in all KO clones used for experiments (Exon 18 and Exon 2 KO clones). B3 is a WT single cell clone. (**C**) Indirect immunofluorescence (IF) (not shown for B3). For IF, cells were stained with DAPI (CST (#4412); 1:1000). IGF1R [alphaR3] (Genetex mouse; 1:50) and goat/anti-mouse-Alexa-Fluor 647, (ThermoFisher (A32728), 1:400). Images were scanned using a Zeiss Elyra S.1- structural imaging microscope (63x oil). Analysis was performed using ZEN lite and Fiji.

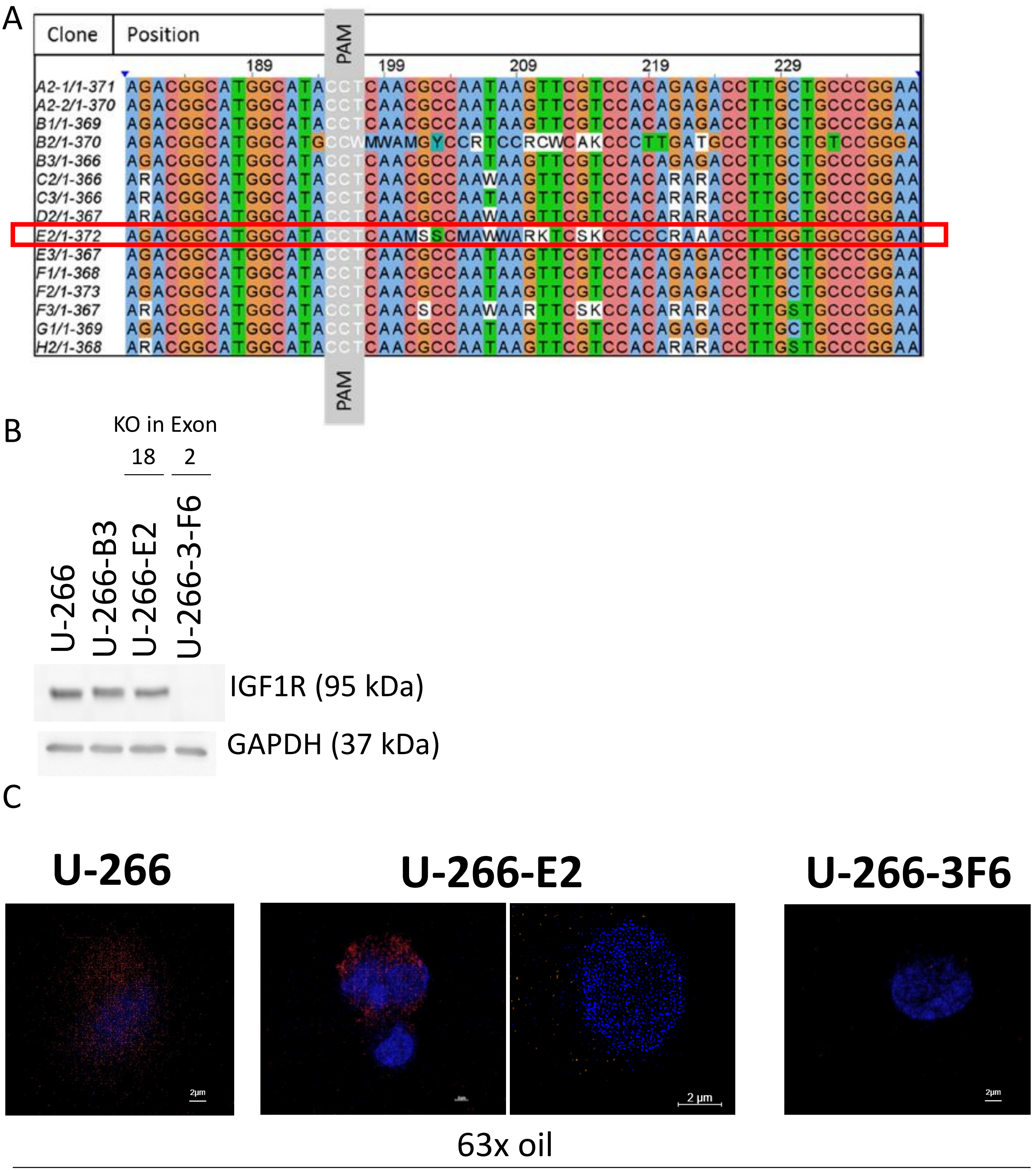

Figure S6 Viability assay (AlamarBlue) of IGF1R overexpressing cells under normal culturing conditions. Results were derived from three independent experiments (mean ± SEM).

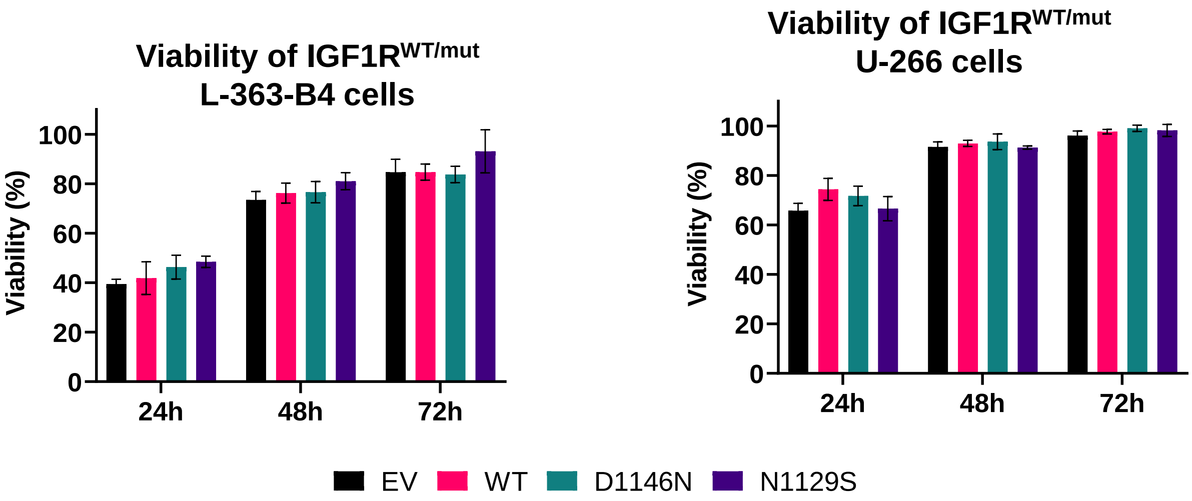

Figure S7 Treatment of MM cell lines with different concentrations of linsitinib and subsequent measurement of the effects on metabolism using an MTT-assay after 72h incubation (mean ± SEM). For 48 and 96h see **Figure 5A**.

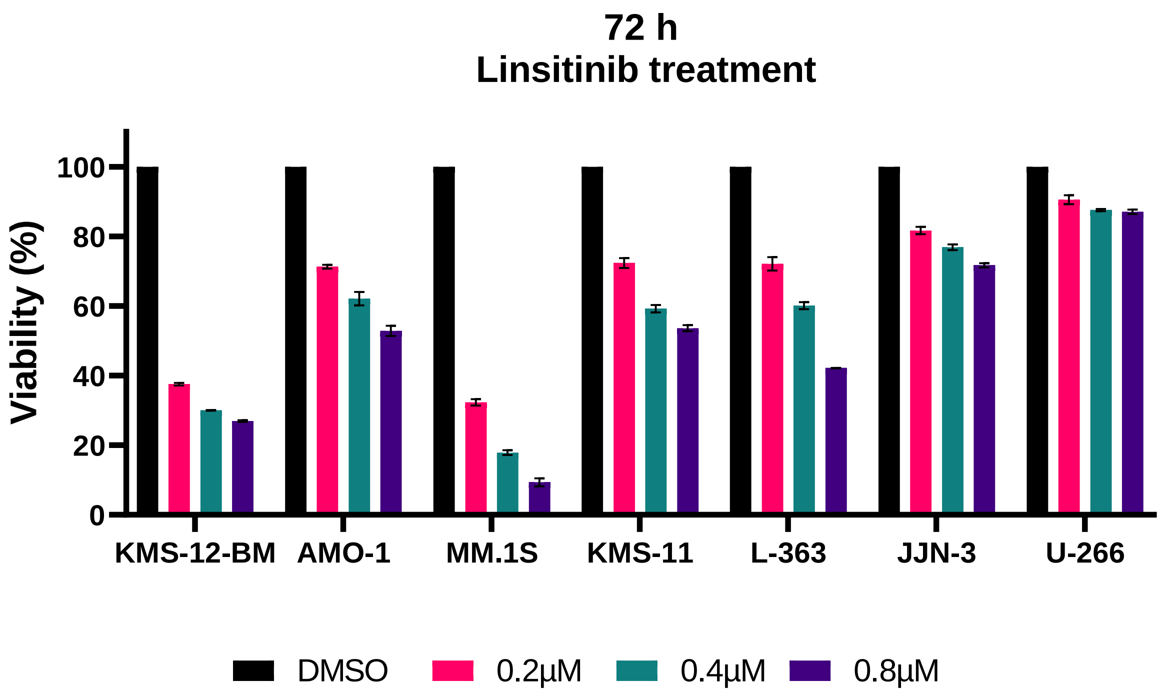

Supplement: Supplementary file 1 [file cancers-16-02139-s001.zip › Supplementary figures.pdf]
