# Supplementary material for: Functional Investigation of IGF1R Mutations in Multiple Myeloma
Source: Cancers (Basel). 2024 Jun 4;16(11):2139. doi: 10.3390/cancers16112139 (PMC11171363; doi:10.3390/cancers16112139)

Uncropped Western Blots of Figures

Figure 2A

Ladder

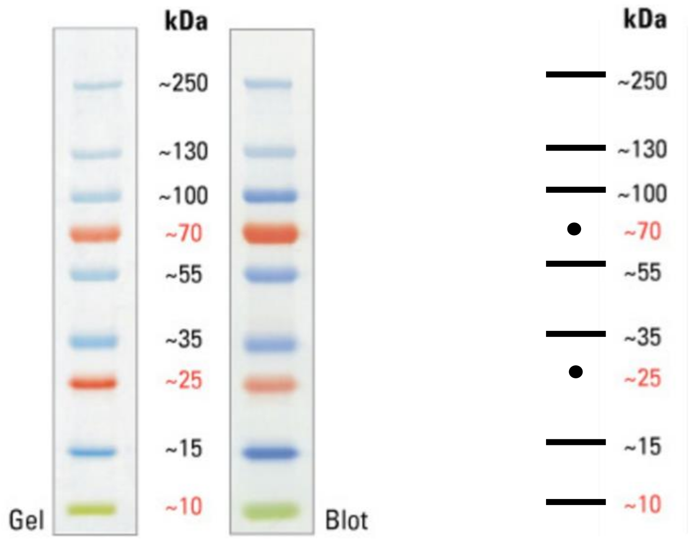

PageRuler™ Plus
Prestained Protein
Ladder, 10 to 250 kDa
Katalognummer: #26
619 Thermo Fischer

Markers:

IGF1R
pIGF1R(Y1135)
AKT
pAKT
MEK1/2
pMEK1/2
ERK1/2
pERK1/2

House keepers (HKEs):
GAPDH
b-actin
b-tubulin

Round depicted
in WB figure

Round not depicted
in WB figure
(used for bar graphs)

Figure 2A (L-363-B4)

Round 1,2:

Loading: L-363-B4 | EV | IGF1R^{WT} | IGF1R^{D1146N} | IGF1R^{N1129S}

Round 3:

Loading: L-363 | L-363-B4 | EV | IGF1R^{WT} | IGF1R^{D1146N} | IGF1R^{N1129S}

Round 4,5:

Loading: L-363 | L-363-B4 | EV | IGF1R^{WT} | IGF1R^{D1146N} | IGF1R^{N1129S} — culturing medium

L-363 | L-363-B4 | EV | IGF1R^{WT} | IGF1R^{D1146N} | IGF1R^{N1129S} — starving medium (crossed out)

concerns only Figure 4, therefore crossed out

****ERK (Round 4,5):**

L-363 | L-363-B4 | EV | IGF1R^{WT} | IGF1R^{D1146N} | IGF1R^{N1129S} — Round 4 culturing medium

L-363 | L-363-B4 | EV | IGF1R^{WT} | IGF1R^{D1146N} | IGF1R^{N1129S} — Round 5 culturing medium

IGF1R (95kDa) // GAPDH (37kDa)

Round 1

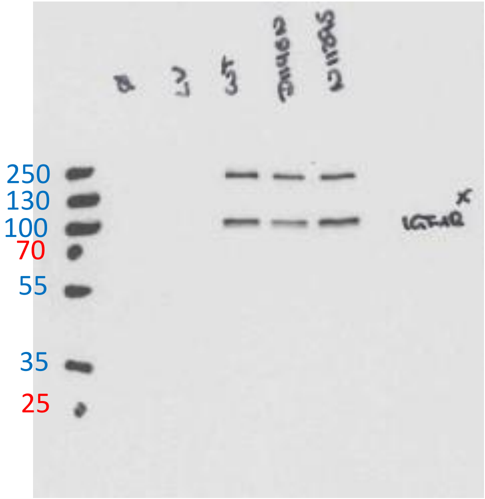

HKE:

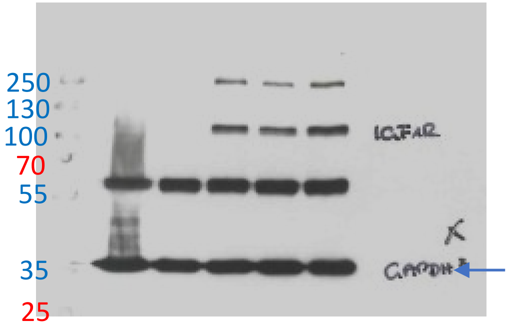

Round 2

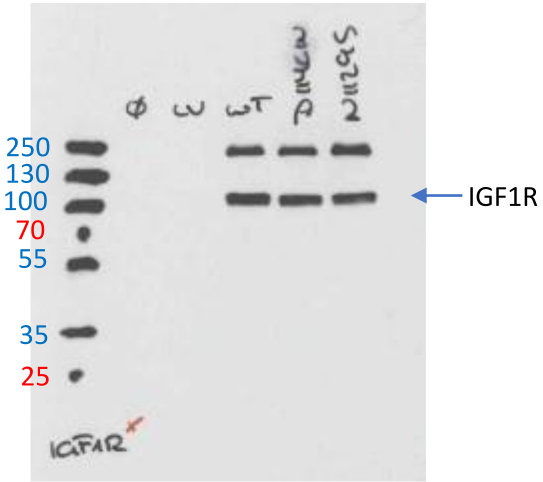

HKE:

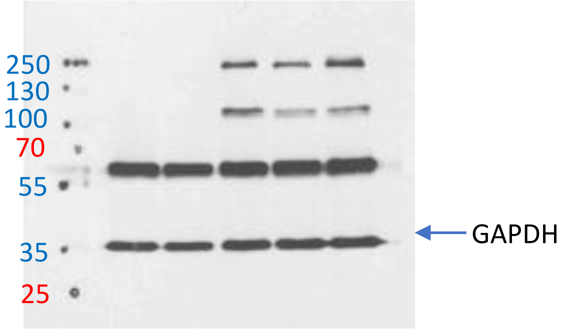

Round 3

Round 4

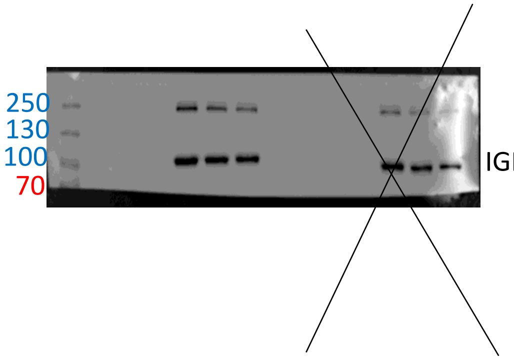

HKE:

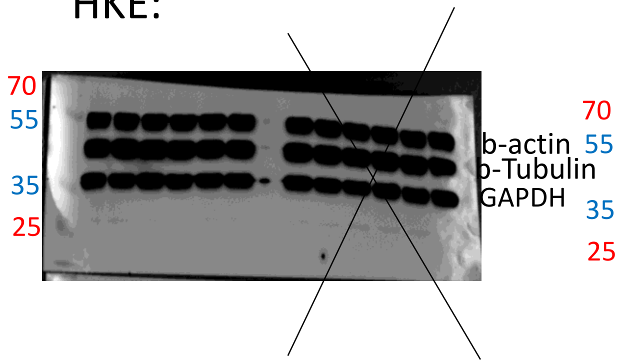

Round 5

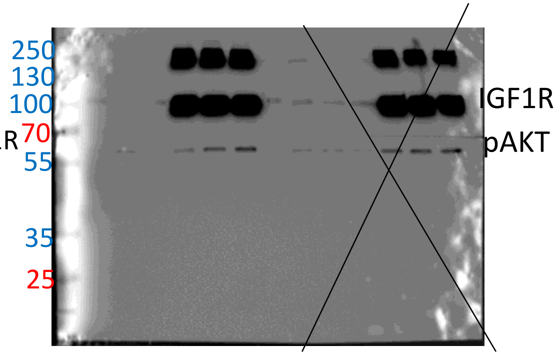

HKE:

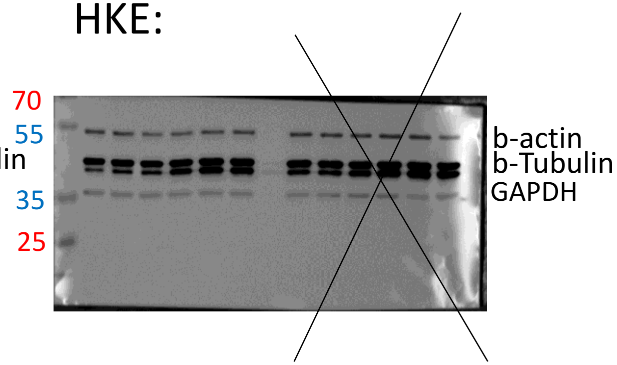

only concerns figure 4

pIGF1R (Y1135) (95kDa) // GAPDH (37kDa)

Round 1

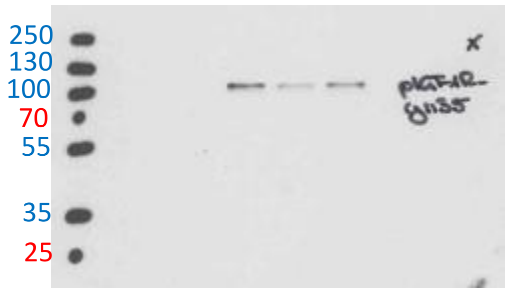

HKE:

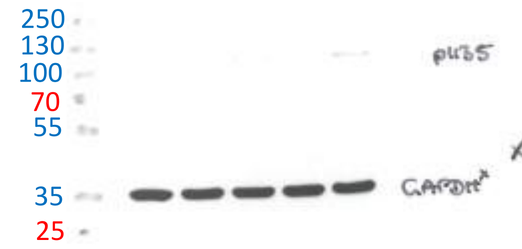

Round 2

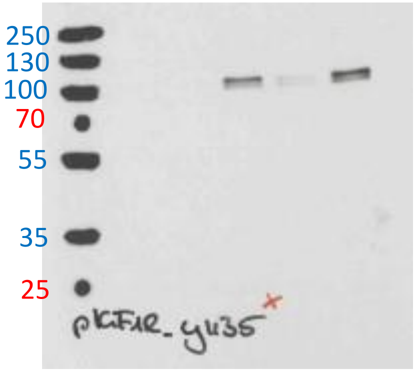

HKE:

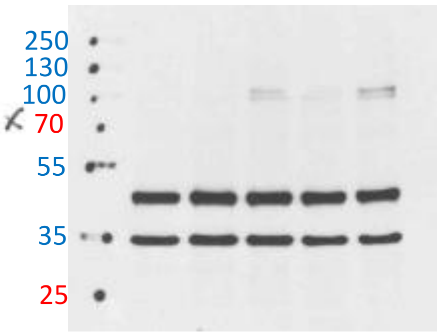

Round 3

Round 4

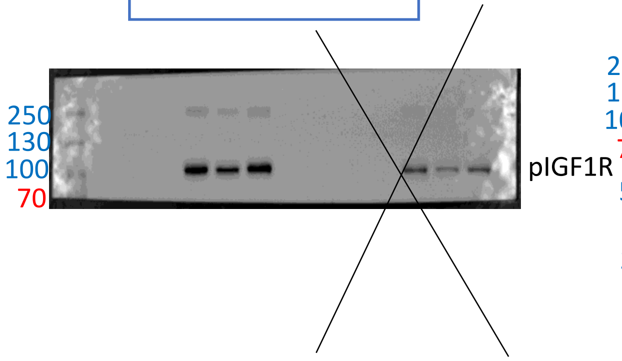

HKE:

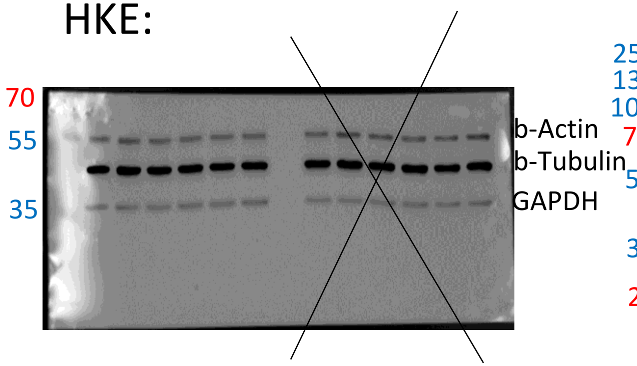

Round 5

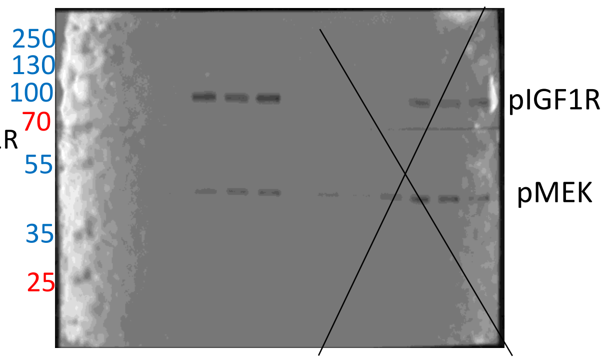

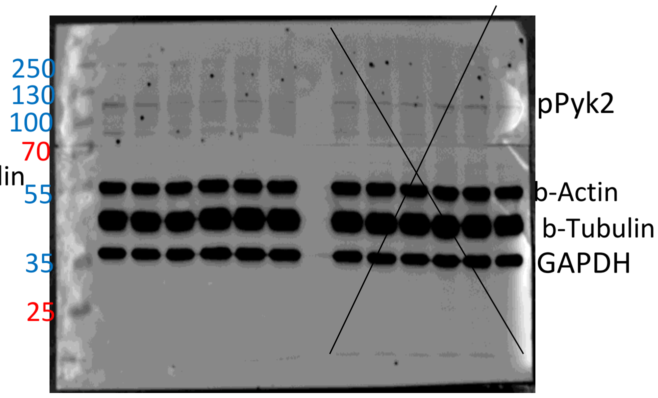

only concerns figure 4

AKT (60kDa) // GAPDH (37kDa)

Round 1

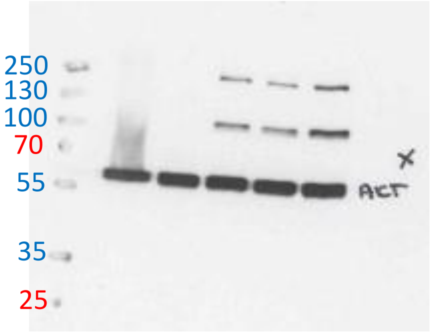

HKE:

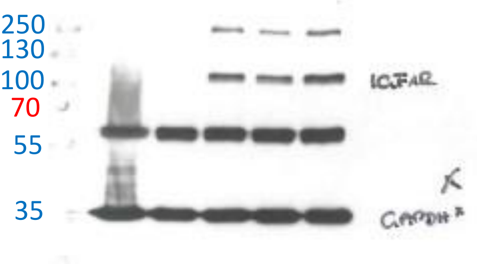

Round 2

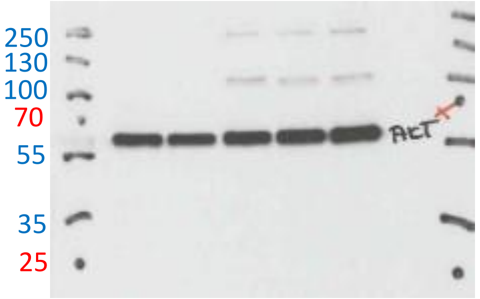

HKE:

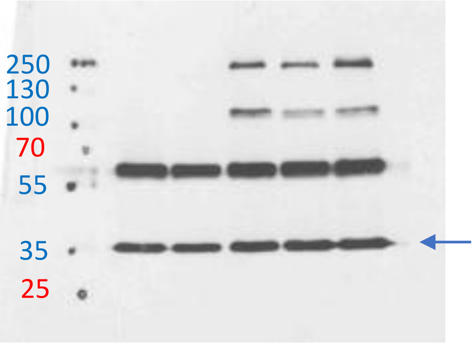

Round 3

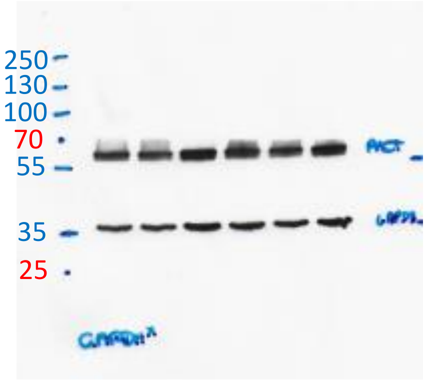

HKE:

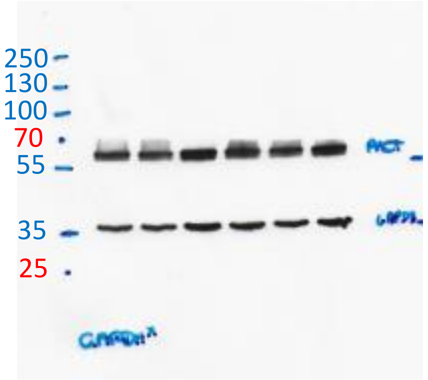

Round 4

Round 5

pAKT (60kDa) // GAPDH (37kDa)

Round 1

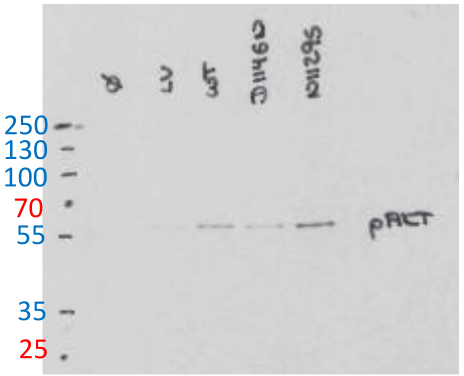

HKE:

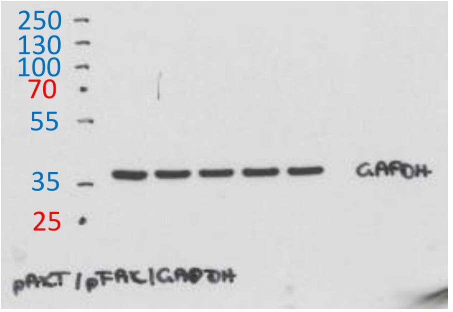

Round 2

Round 3

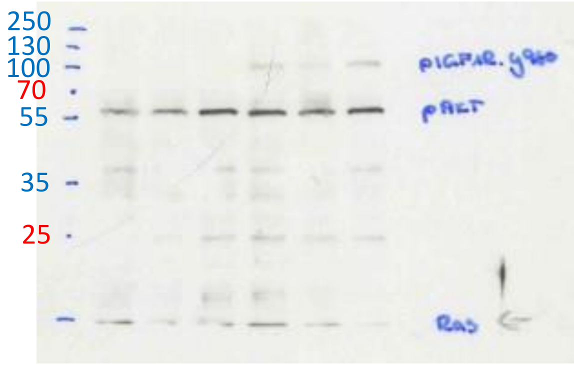

HKE:

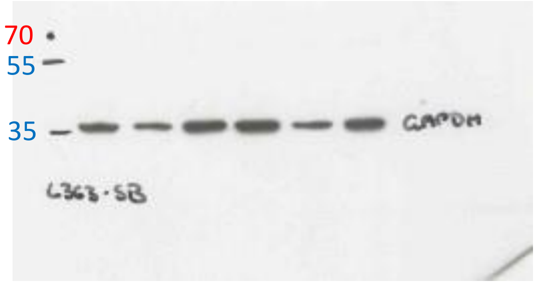

Round 4

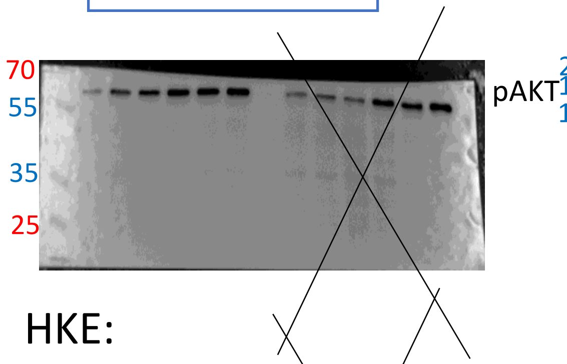

HKE:

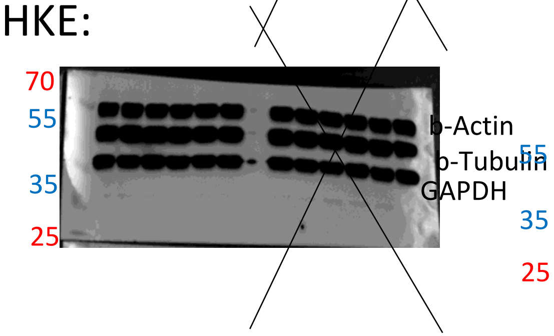

Round 5

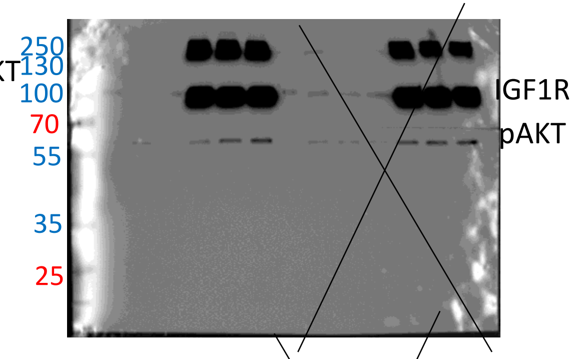

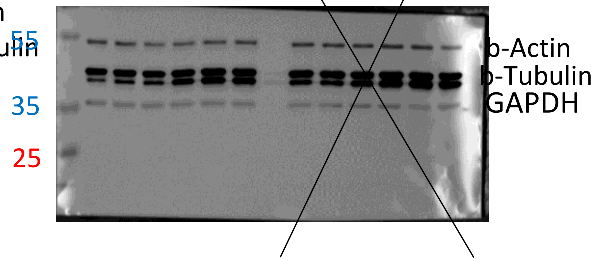

only concerns figure 4

MEK 1/2 (45kDa) // GAPDH (37kDa)

Round 1

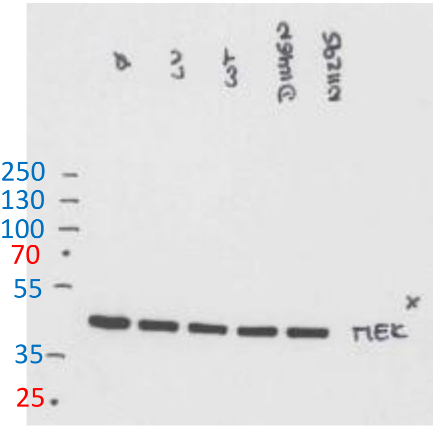

HKE:

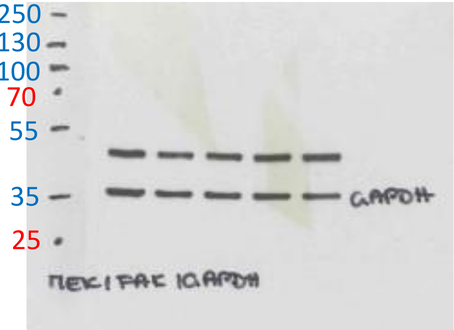

Round 2

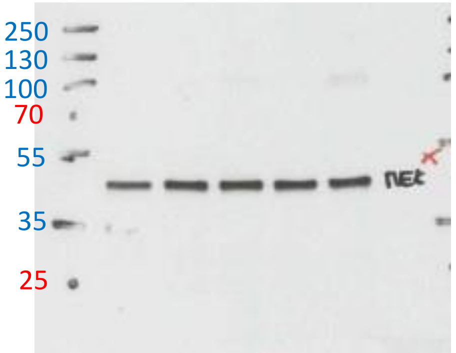

HKE:

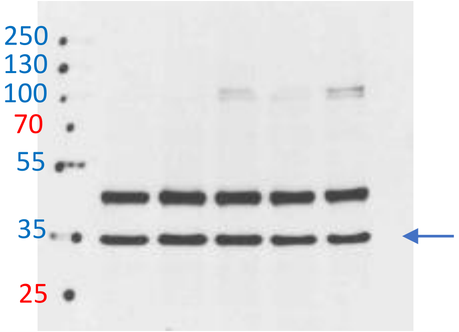

Round 3

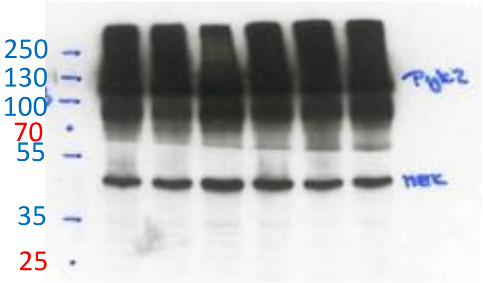

HKE:

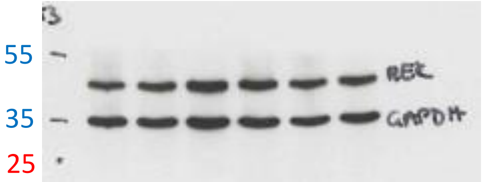

Round 4

Round 5

pMEK 1/2 (45kDa) // GAPDH (37kDa)

Round 1

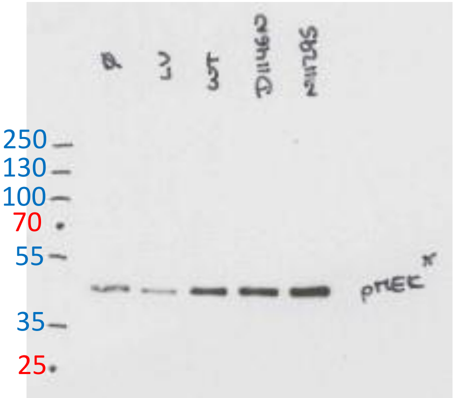

HKE:

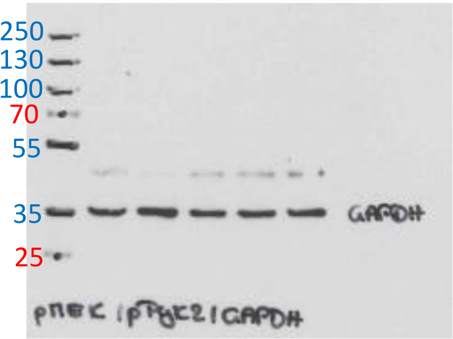

Round 2

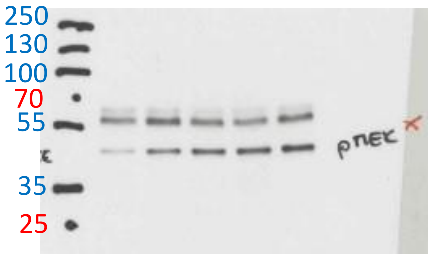

HKE:

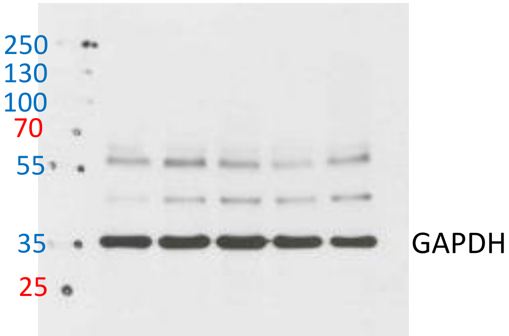

Round 3

Round 4

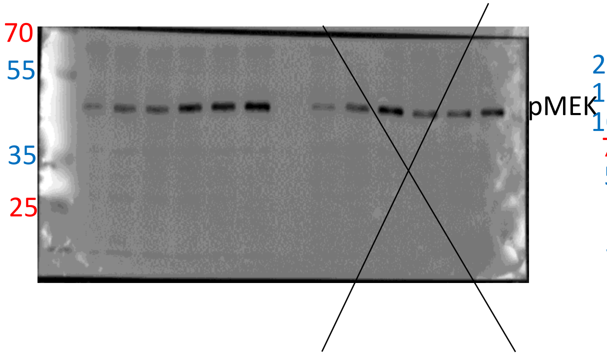

HKE:

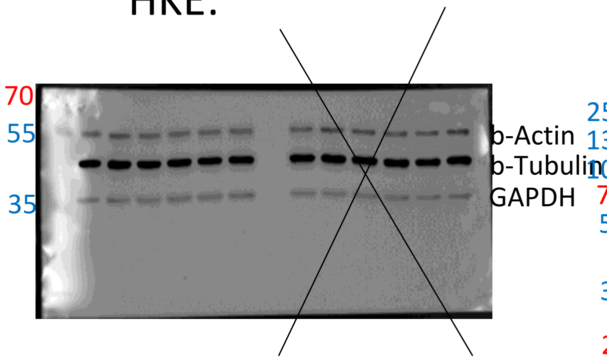

Round 5

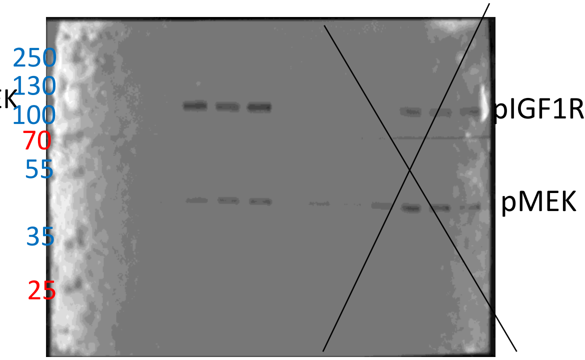

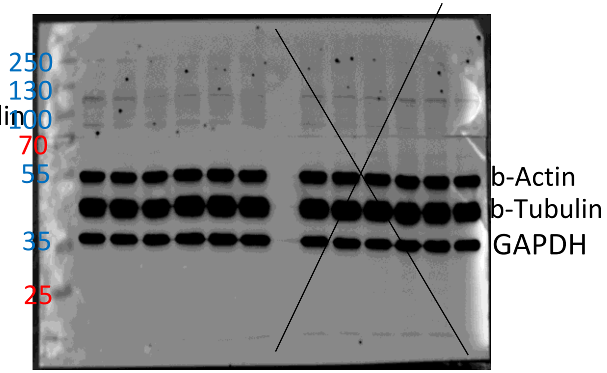

only concerns figure 4

ERK 1/2 (45kDa) // GAPDH (37kDa)

Round 1

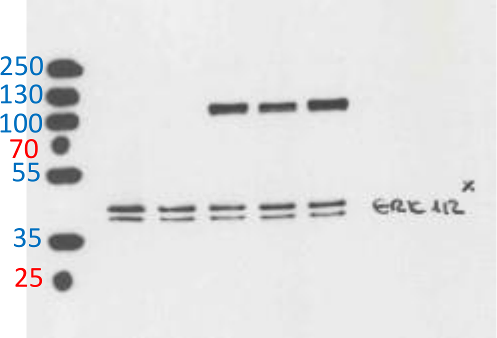

HKE:

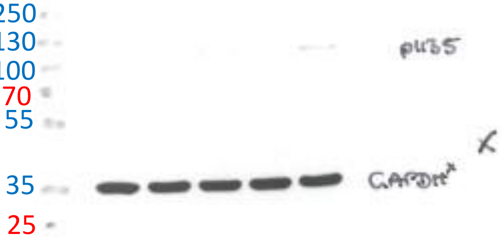

Round 2

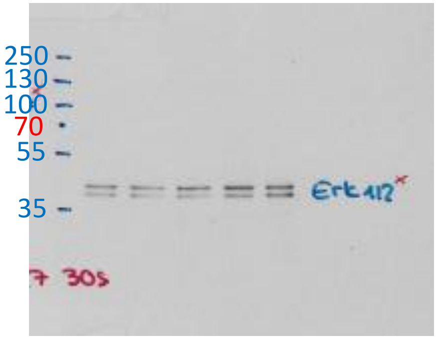

HKE:

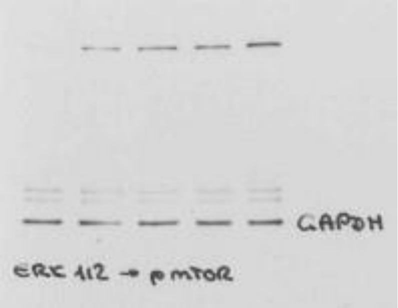

Round 3

Round 4

Round 5

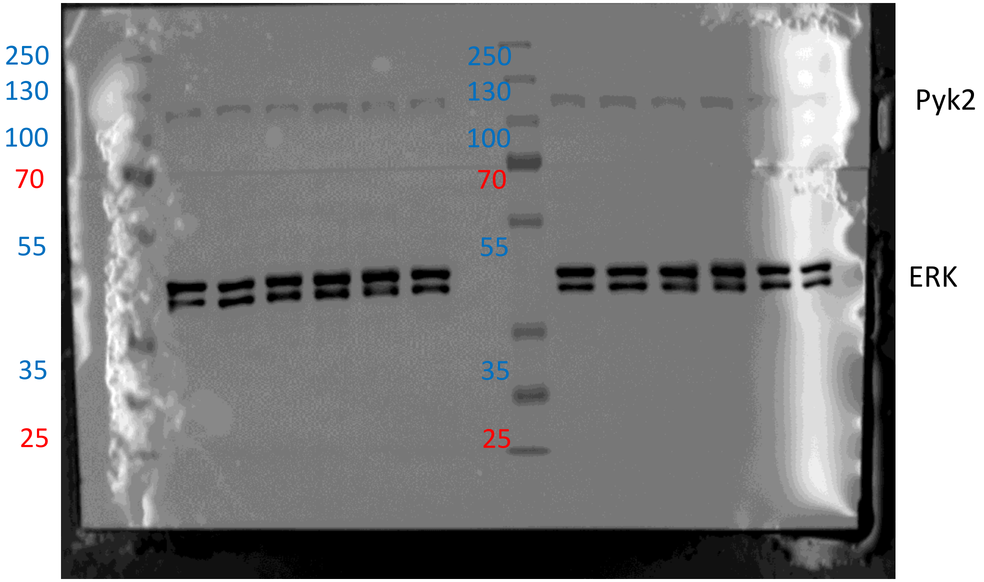

HKE:

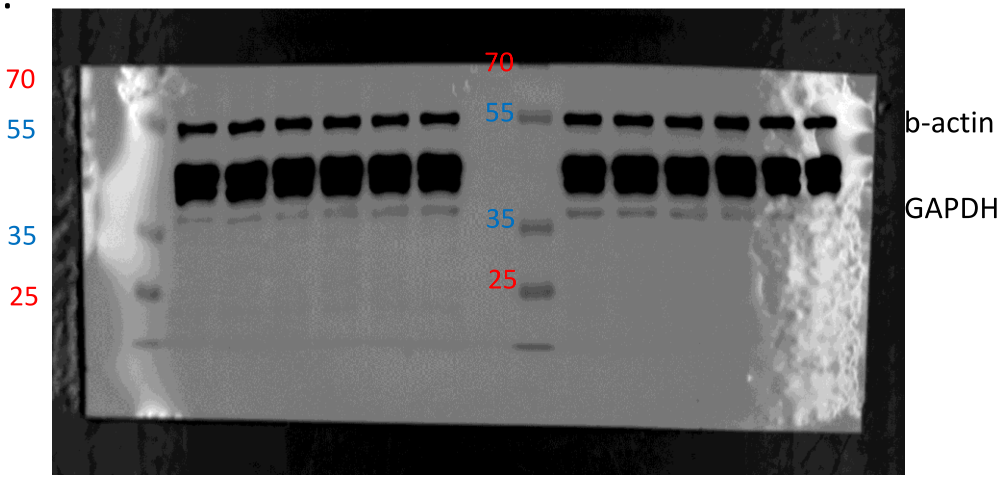

pERK 1/2 (45kDa) // GAPDH (37kDa)

Round 1	Round 2	Round 3	Round 4	Round 5

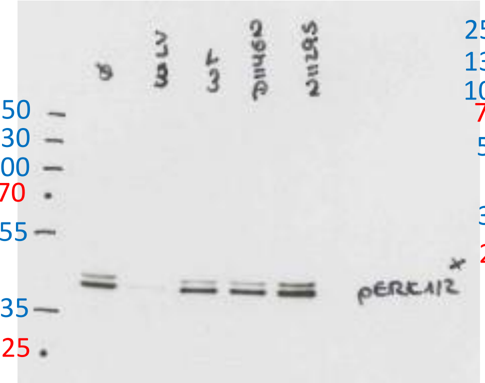

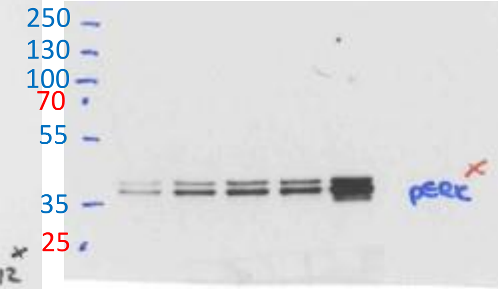

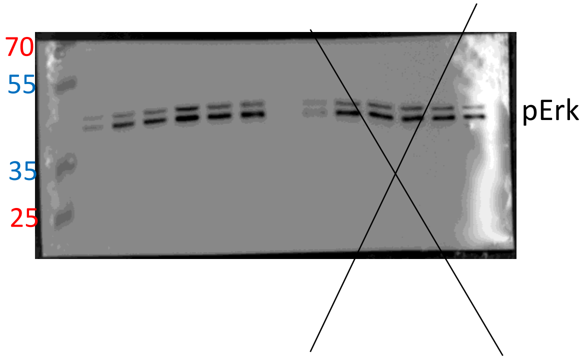

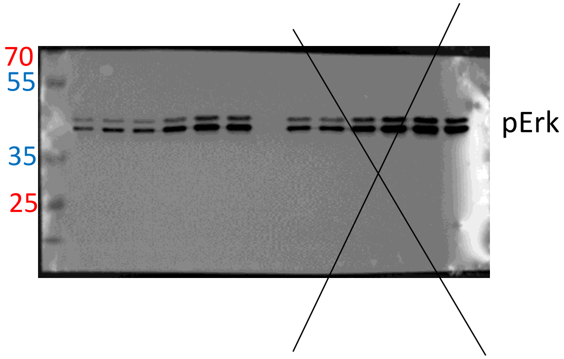

HKE:

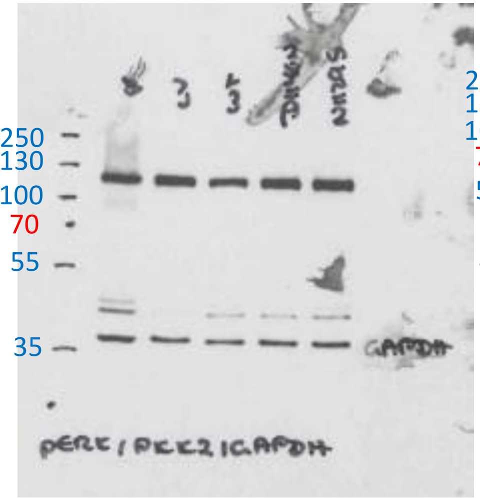

HKE:

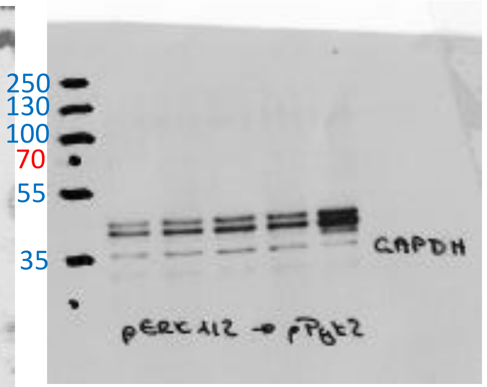

HKE:

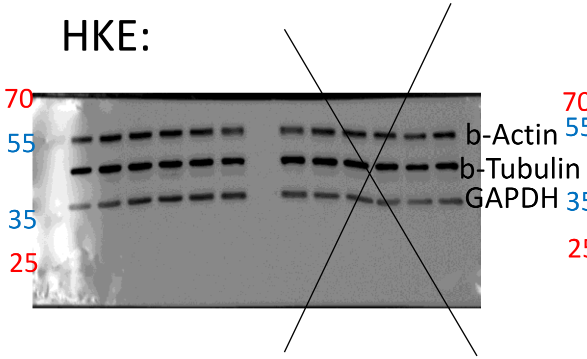

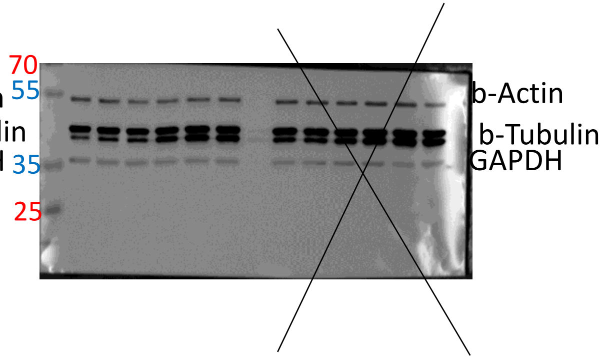

only concerns figure 4

Figure 2A (AMO-1)

Round 1,2,3,6:

Loading: EV | IGF1R^{WT} | IGF1R^{D1146N} | IGF1R^{N1129S}

Round 4,5,7:

Loading: AMO-1 | EV | IGF1R^{WT} | IGF1R^{D1146N} | IGF1R^{N1129S}

Round 8,9:

Loading: AMO-1 | EV | IGF1R^{WT} | IGF1R^{D1146N} | IGF1R^{N1129S} (culturing medium) | ~~AMO-1 | EV | IGF1R^{WT} | IGF1R^{D1146N} | IGF1R^{N1129S} (starving medium)~~

culturing medium

starving medium

Concerns only Figure 4, therefore crossed out

IGF1R (95kDa) // GAPDH (37kDa)

Round 1

Round 2

Round 3

Round 4

Round 5

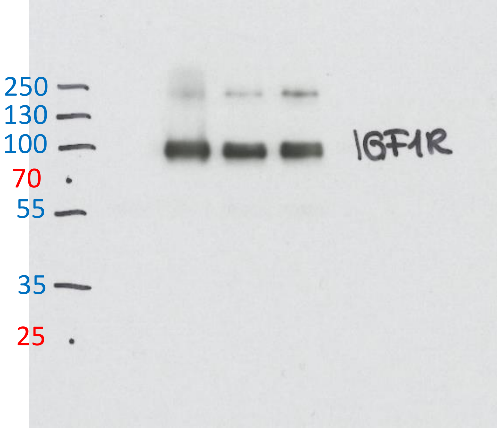

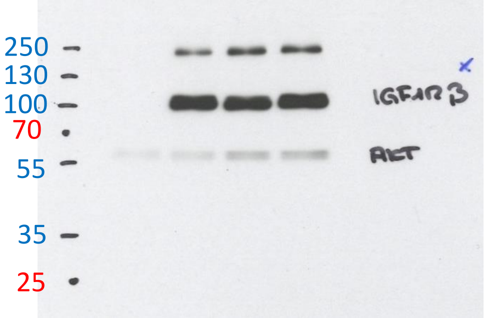

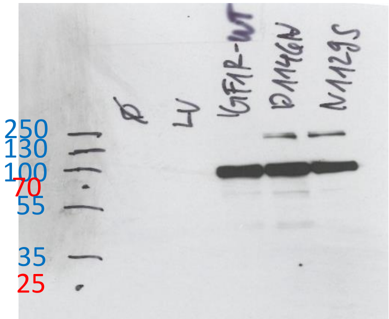

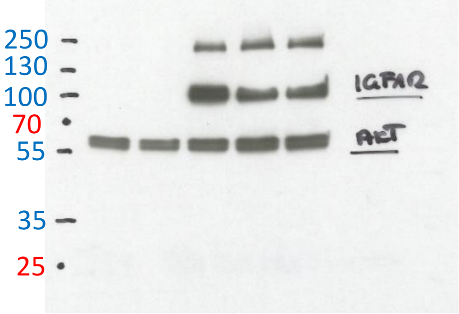

HKE:

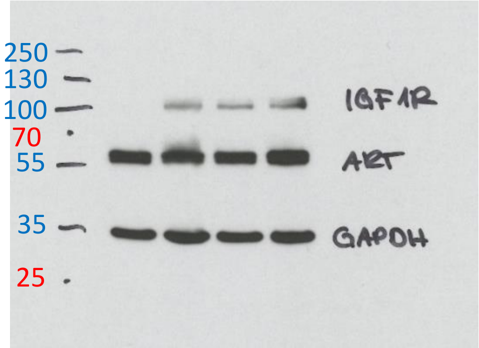

HKE:

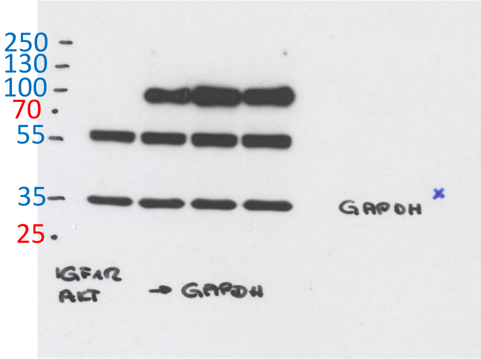

HKE:

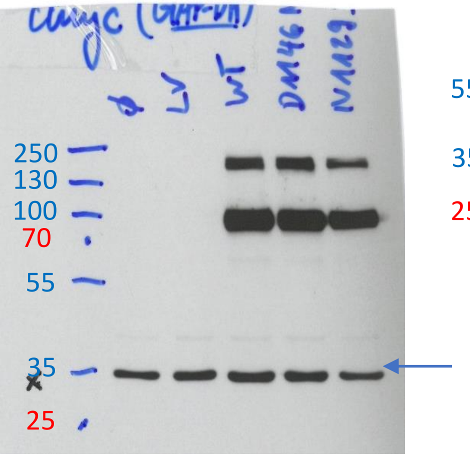

HKE:

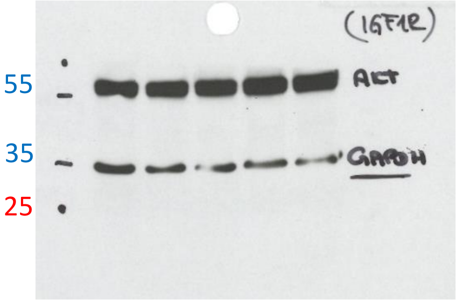

IGF1R (95kDa) // GAPDH (37kDa)

Round 6

Round 7

Round 8

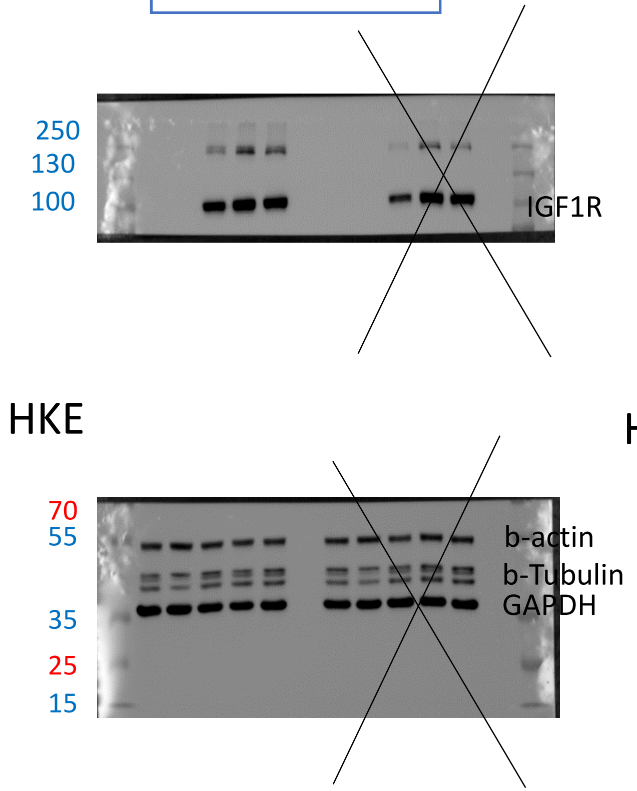

Round 9

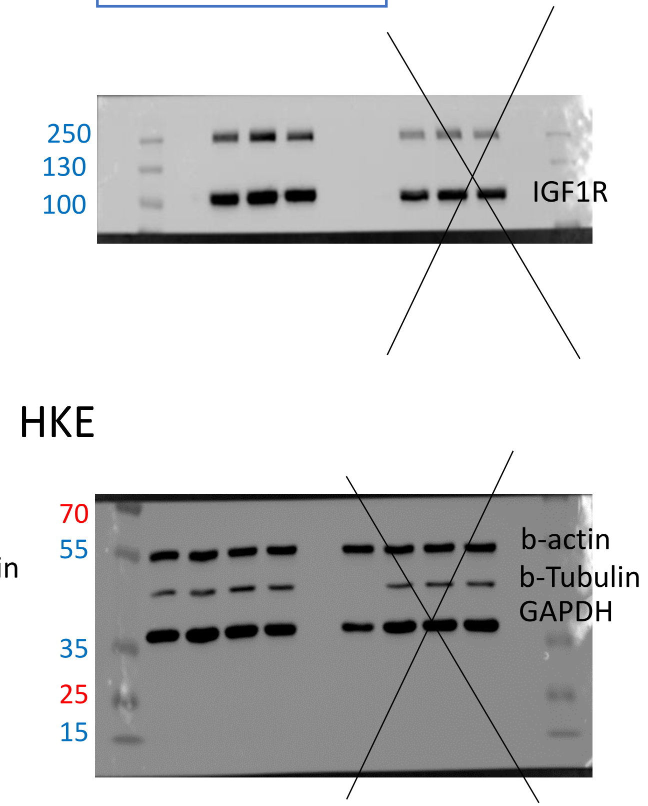

only concerns figure 4

pIGF1R (Y1135) (95kDa) // GAPDH (37kDa)

Round 1

Round 2

Round 3

Round 4

Round 5

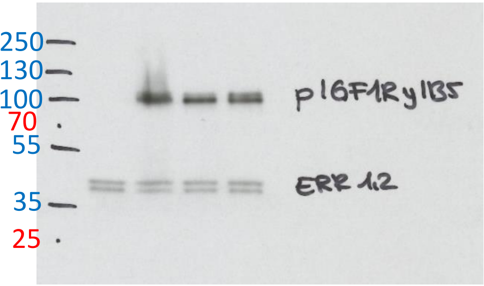

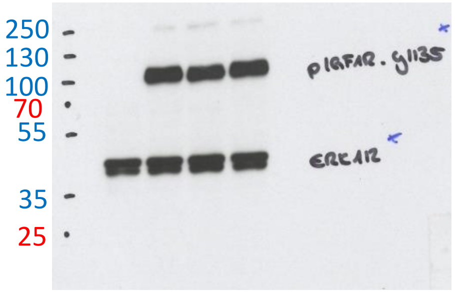

HKE:

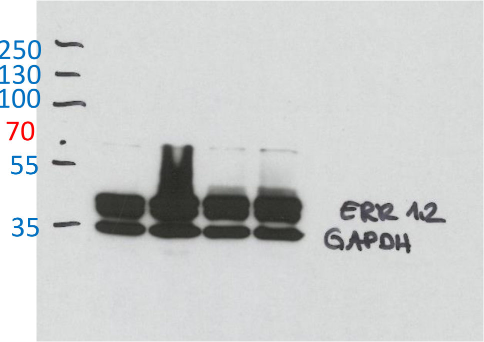

HKE:

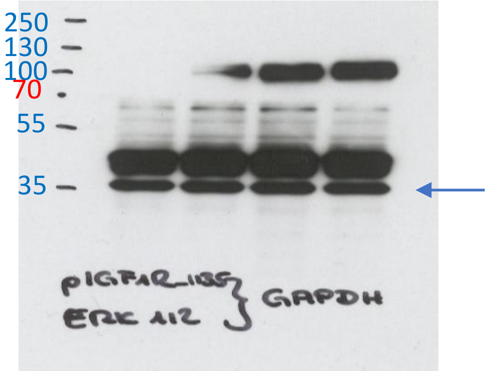

pIGF1R (Y1135) (95kDa) // GAPDH (37kDa)

Round 6

Round 7

Round 8

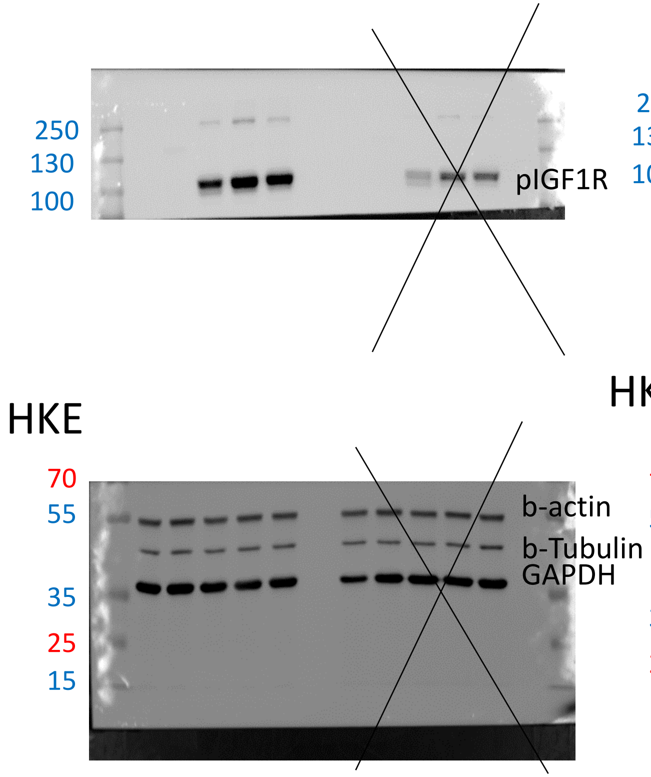

Round 9

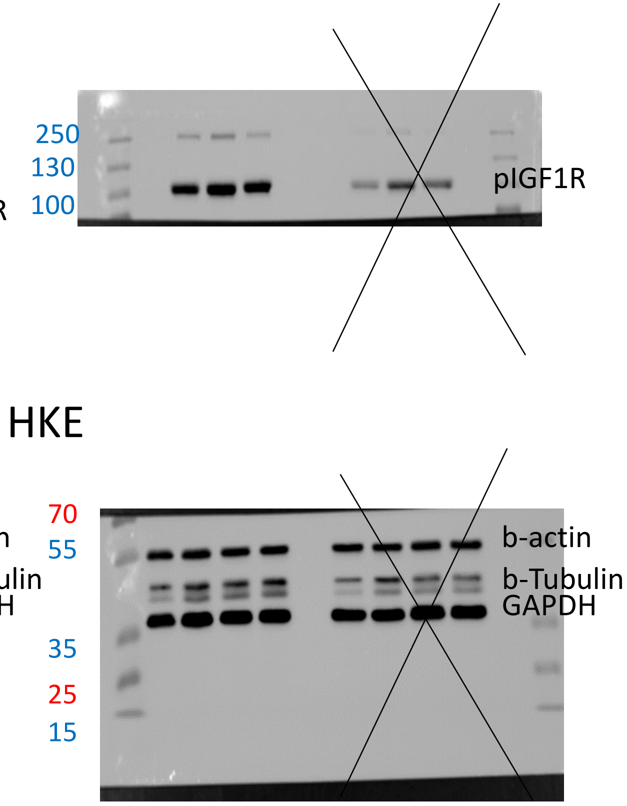

only concerns figure 4

AKT (60kDa) // GAPDH (37kDa)

Round 1

Round 2

Round 3

Round 4

Round 5

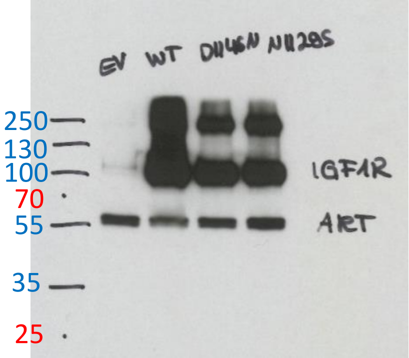

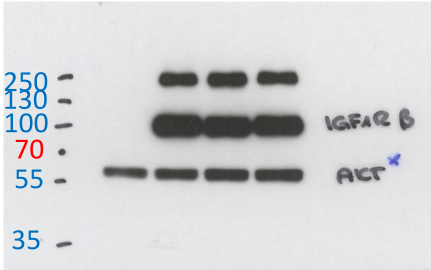

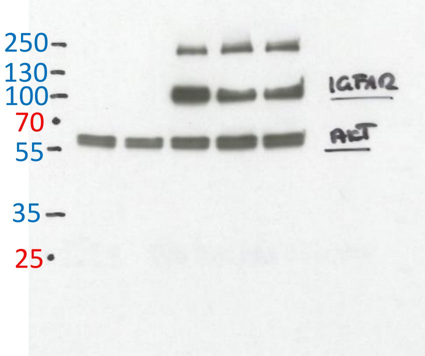

HKE:

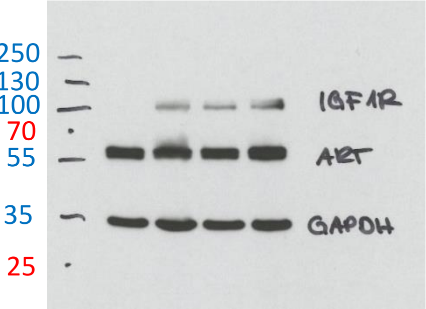

HKE:

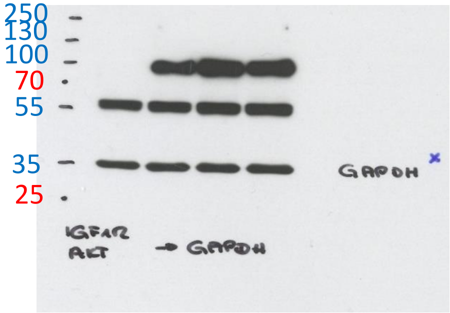

HKE:

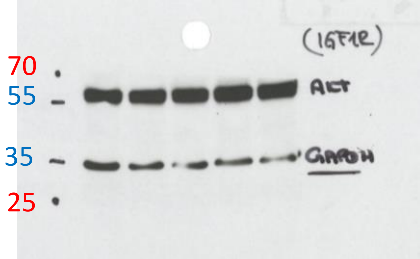

AKT (60kDa) // GAPDH (37kDa)

Round 6

Round 7

Round 8

Round 9

pAKT (60kDa) // GAPDH (37kDa)

Round 1

Round 2

Round 3

Round 4

Round 5

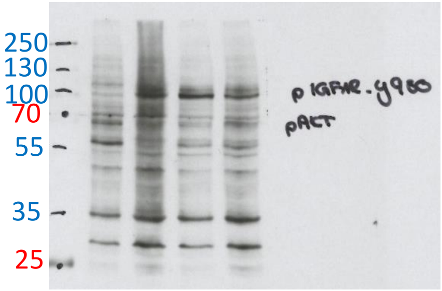

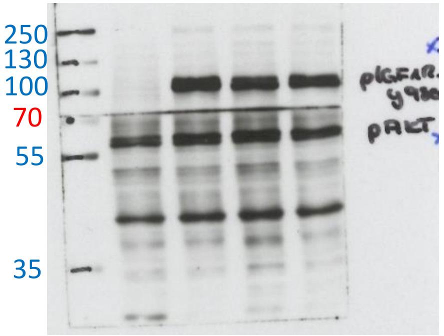

HKE:

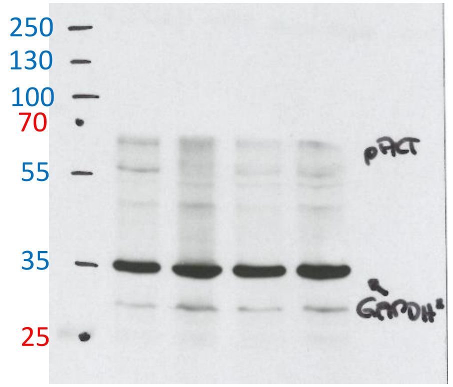

HKE:

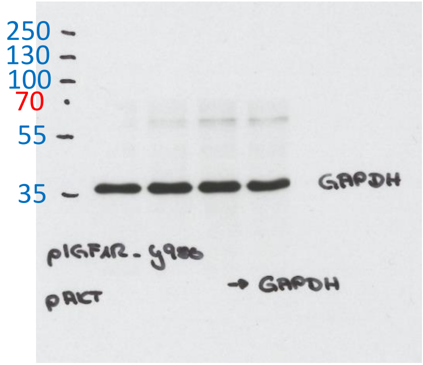

pAKT (60kDa) // GAPDH (37kDa)

Round 6

Round 7

Round 8

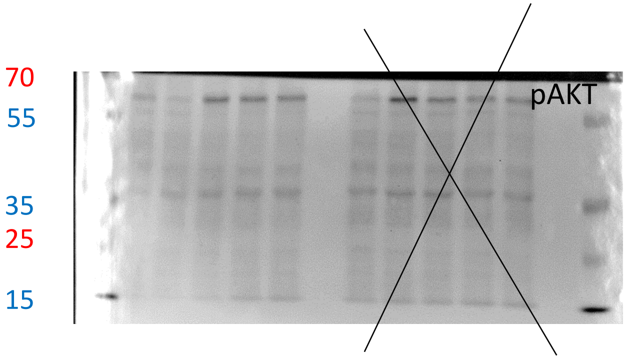

HKE:

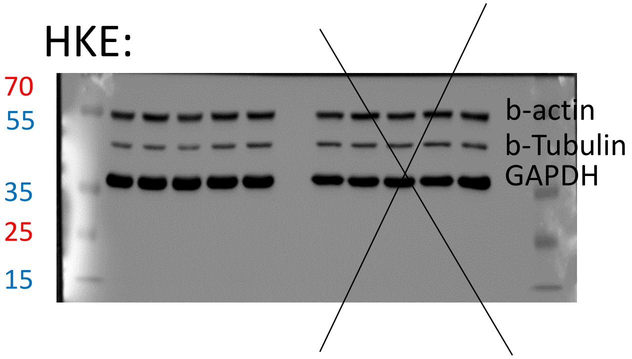

Round 9

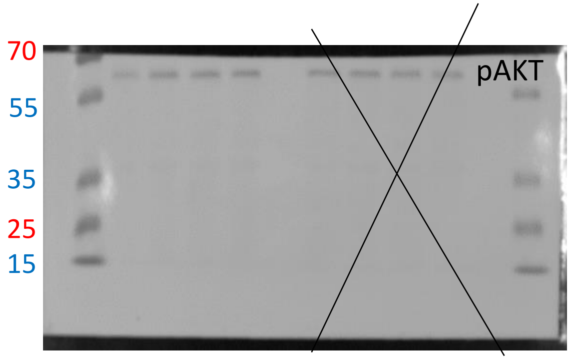

HKE:

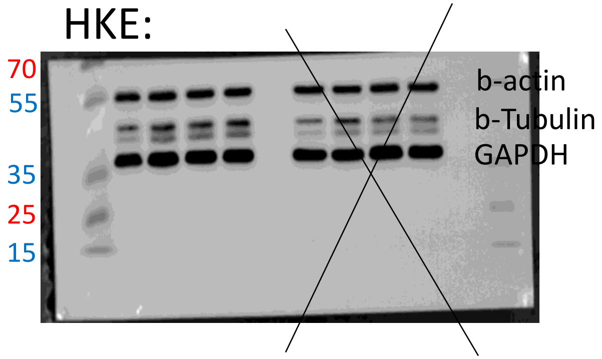

X only concerns figure 4

MEK 1/2 (45kDa) // GAPDH (37kDa)

Round 1

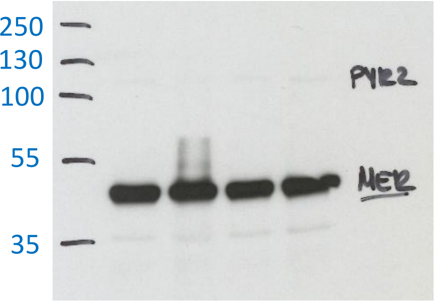

HKE:

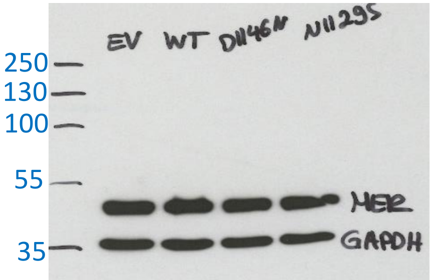

Round 2

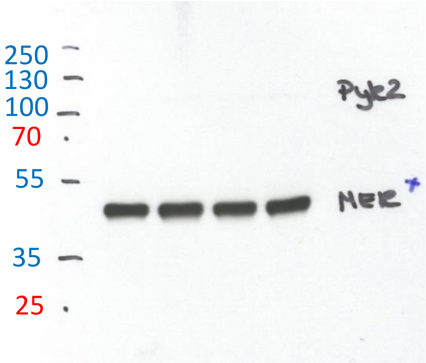

HKE:

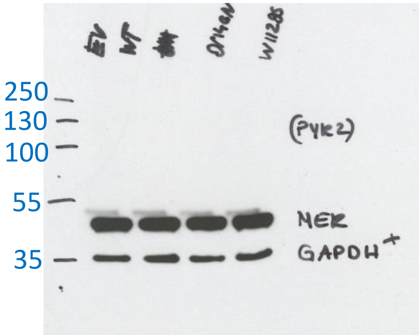

Round 3

Round 4

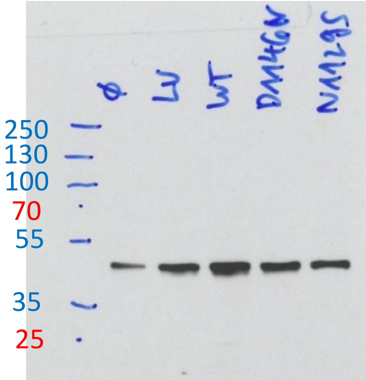

HKE:

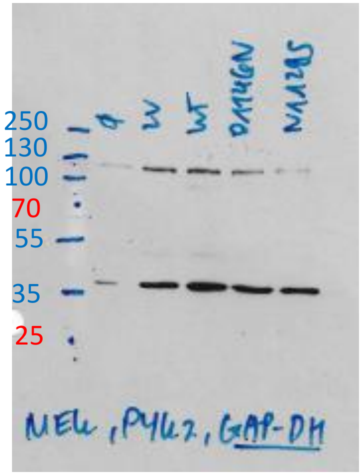

Round 5

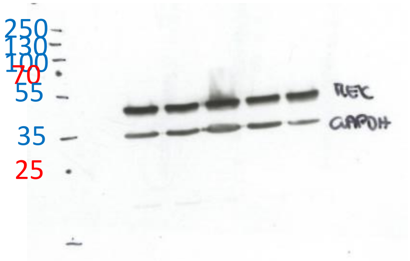

HKE:

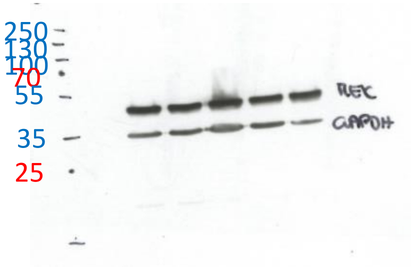

MEK 1/2 (45kDa) // GAPDH (37kDa)

Round 6

Round 7

Round 8

Round 9

pMEK 1/2 (45kDa) // GAPDH (37kDa)

Round 1

Round 2

Round 3

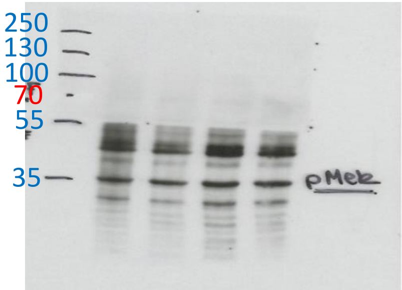

HKE:

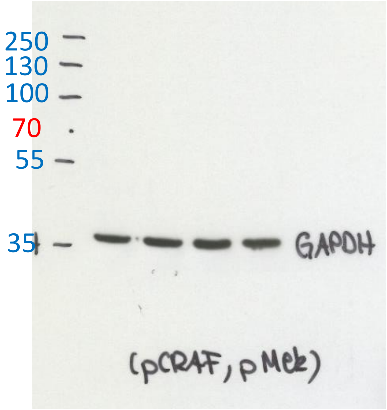

Round 4

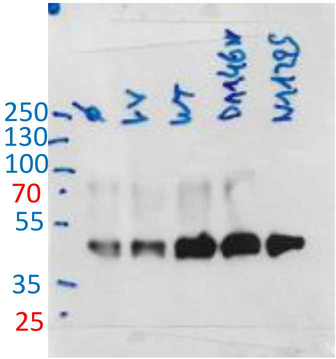

HKE:

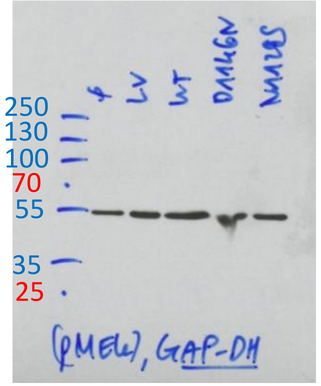

Round 5

pMEK 1/2 (45kDa) // GAPDH (37kDa)

Round 6

Round 7

Round 8

Round 9

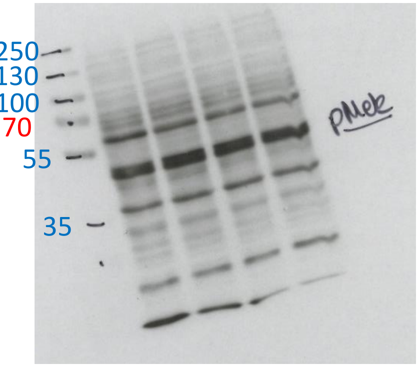

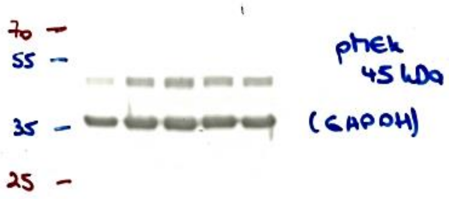

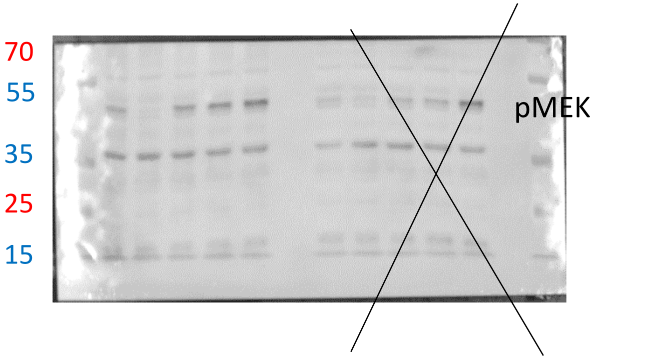

HKE:

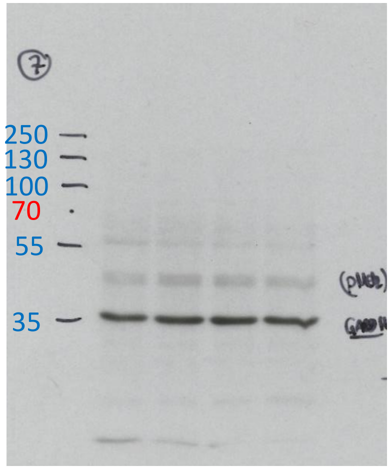

HKE:

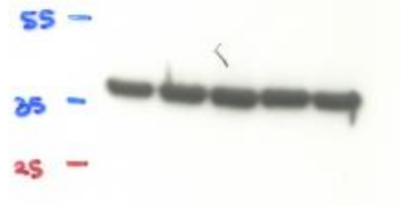

HKE:

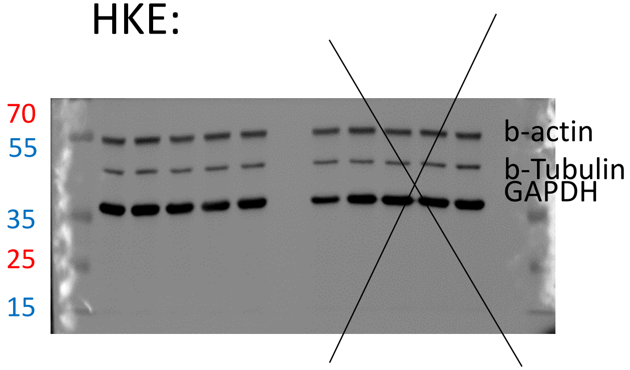

only concerns figure 4

ERK 1/2 (45kDa) // GAPDH (37kDa)

Round 1

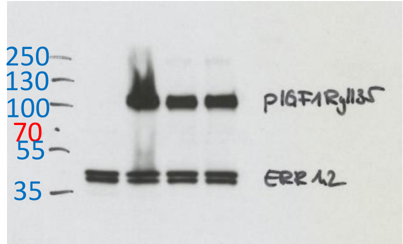

HKE:

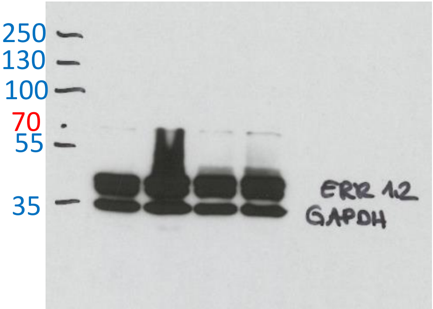

Round 2

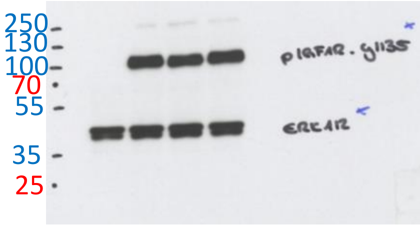

HKE:

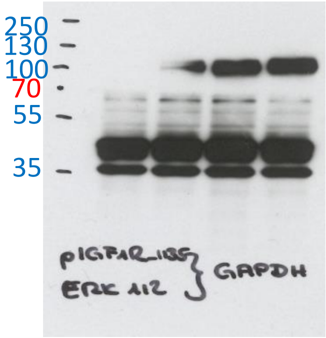

Round 3

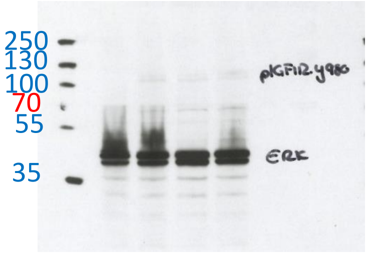

HKE:

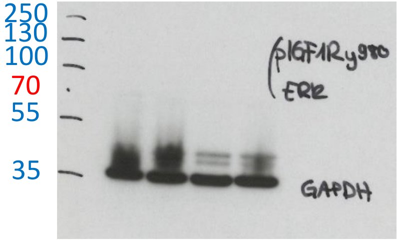

Round 4

Round 5

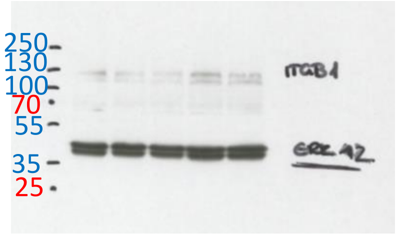

HKE:

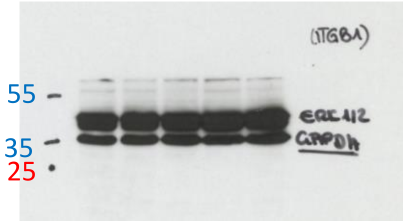

ERK 1/2 (45kDa) // GAPDH (37kDa)

Round 6

Round 7

Round 8

Round 9

pERK 1/2 (45kDa) // GAPDH (37kDa)

Round 1

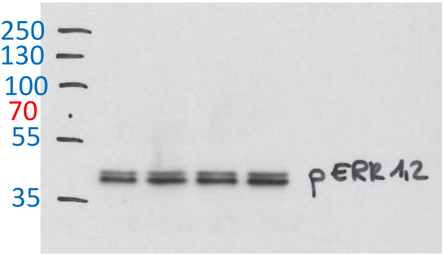

HKE:

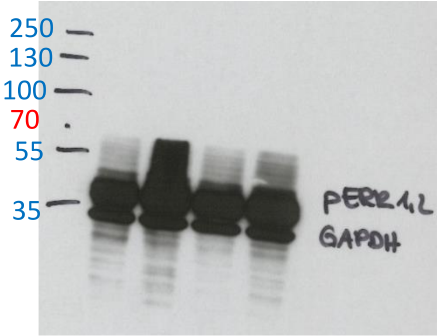

Round 2

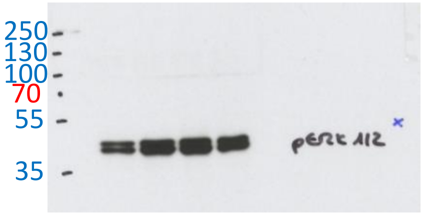

HKE:

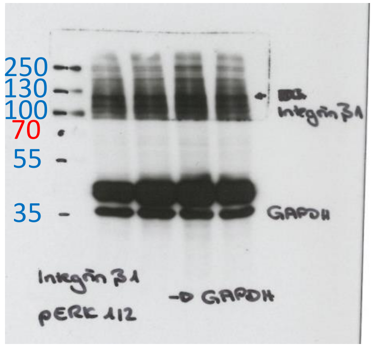

Round 3

Round 4

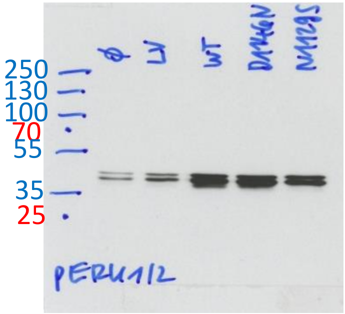

HKE:

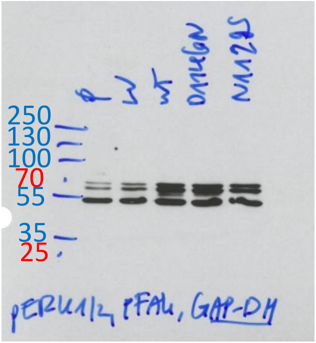

Round 5

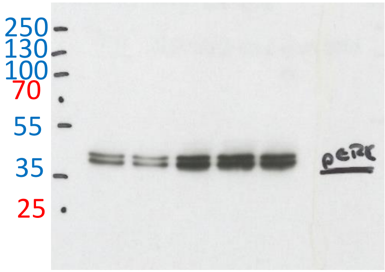

HKE:

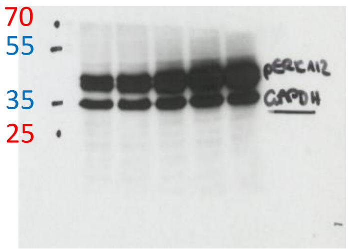

pERK 1/2 (45kDa) // GAPDH (37kDa)

Round 6

Round 7

Round 8

Round 9

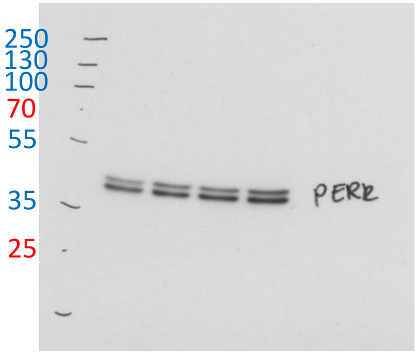

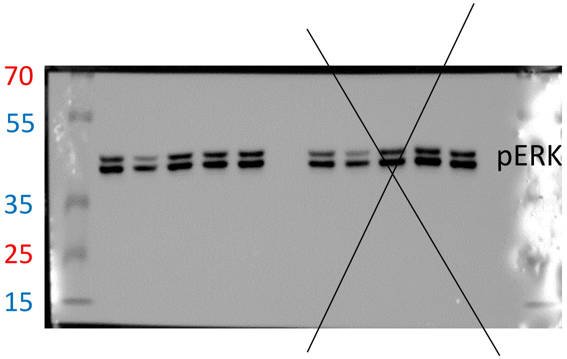

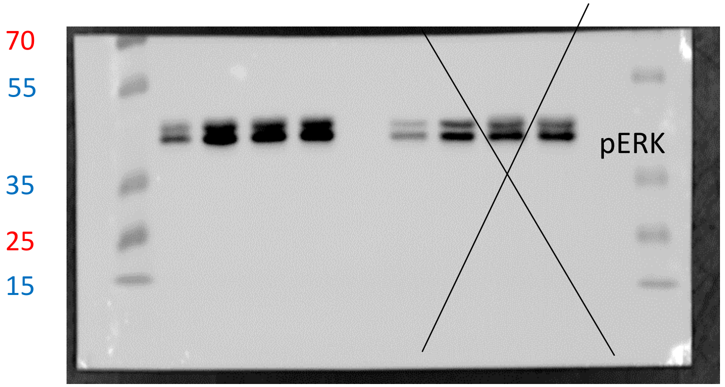

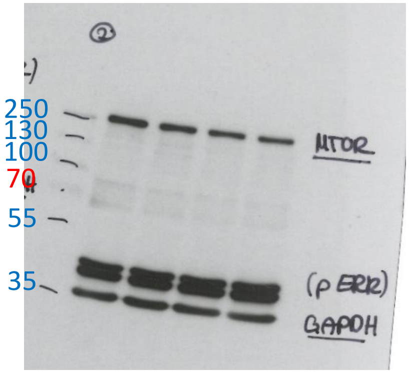

HKE:

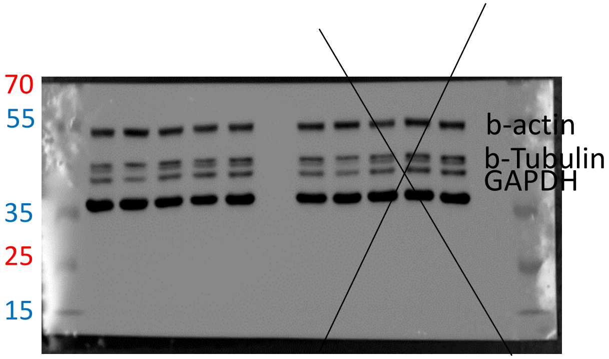

HKE:

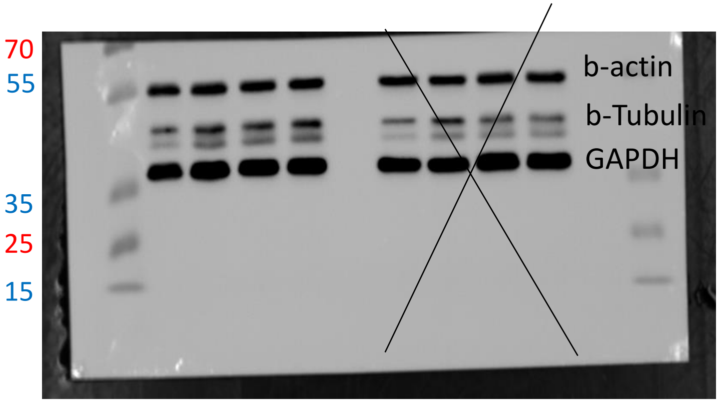

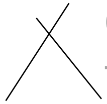 only concerns figure 4

Figure 2A (U-266)

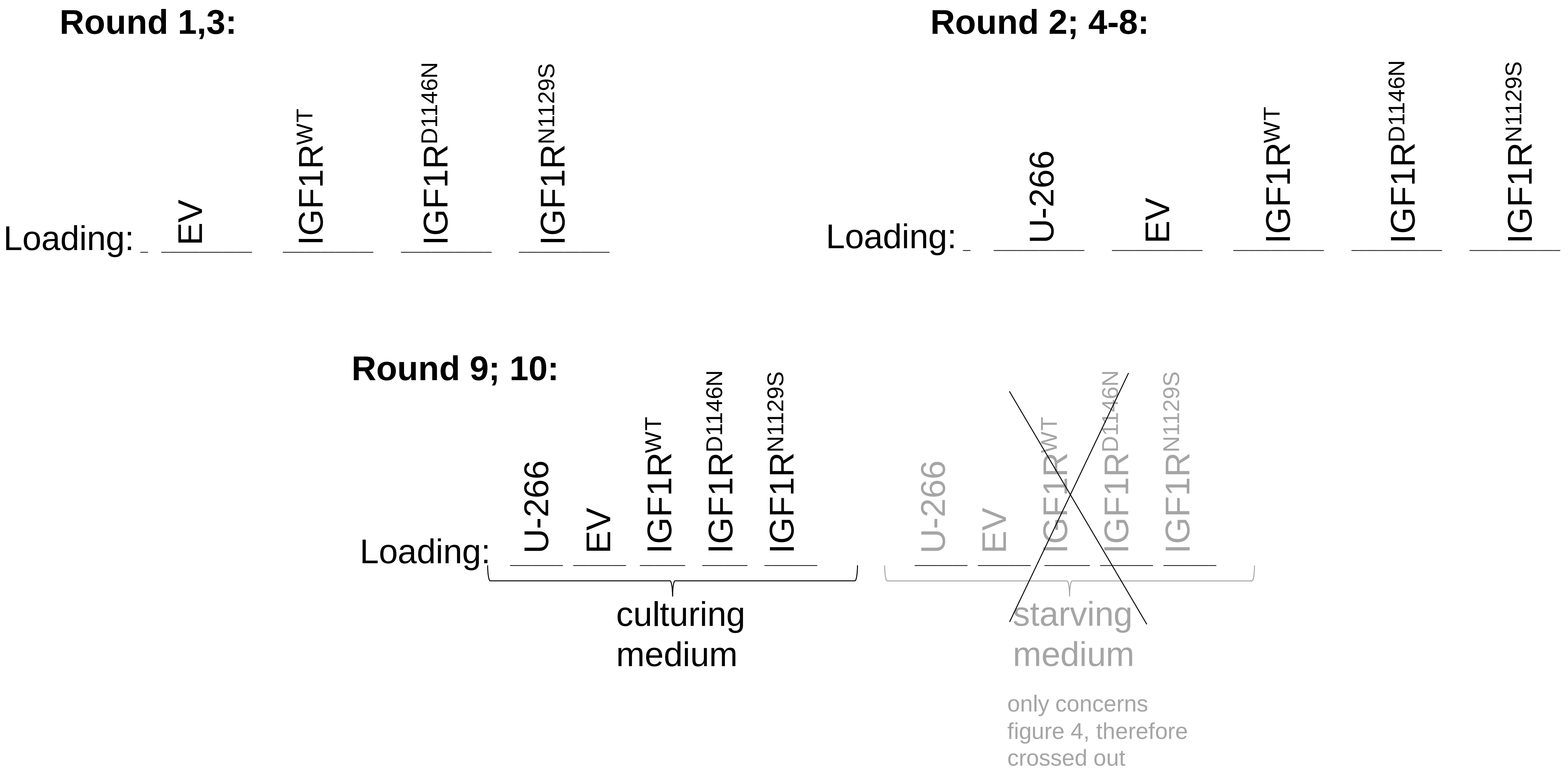

IGF1R (95kDa) // GAPDH (37kDa)

Round 1

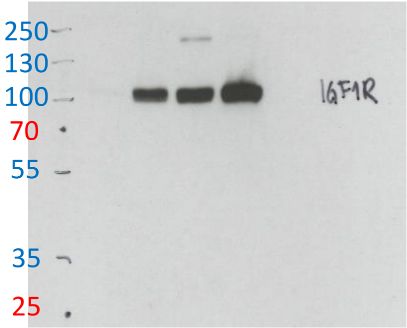

HKE:

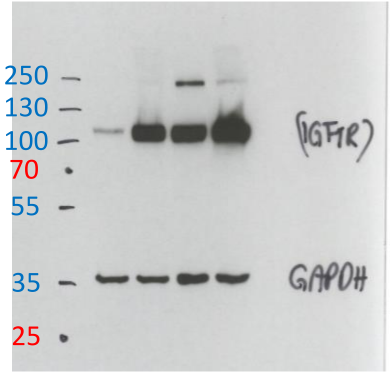

Round 2

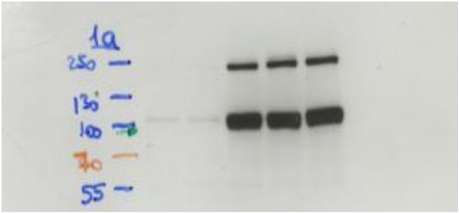

HKE:

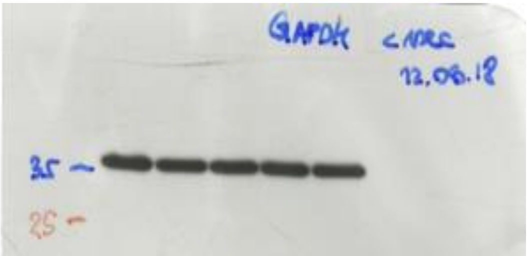

Round 3

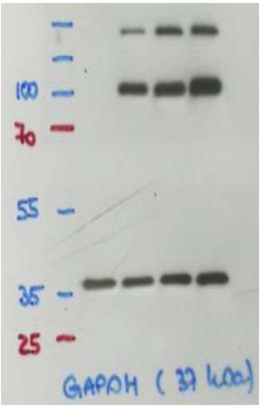

HKE:

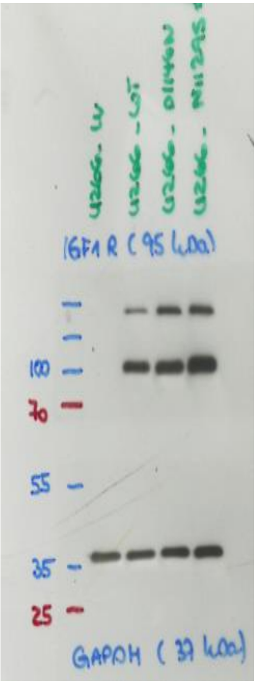

Round 4

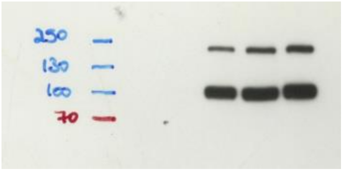

HKE:

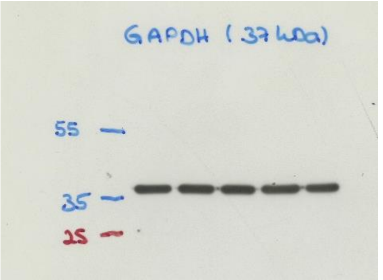

Round 5

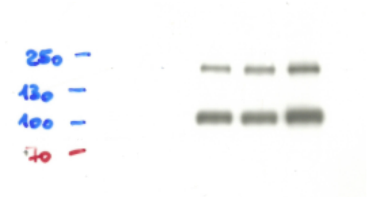

HKE:

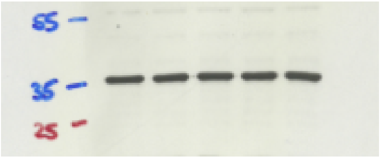

IGF1R (95kDa) // GAPDH (37kDa)

Round 6

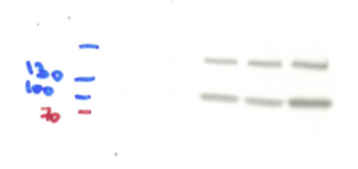

HKE:

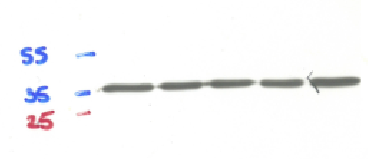

Round 7

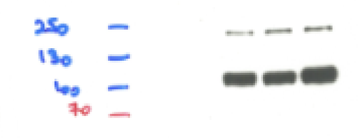

HKE:

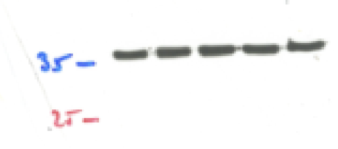

Round 8

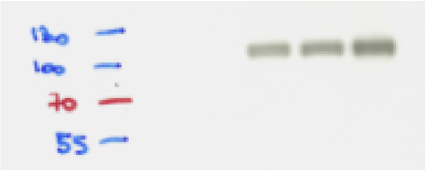

HKE:

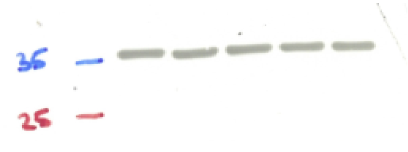

Round 9

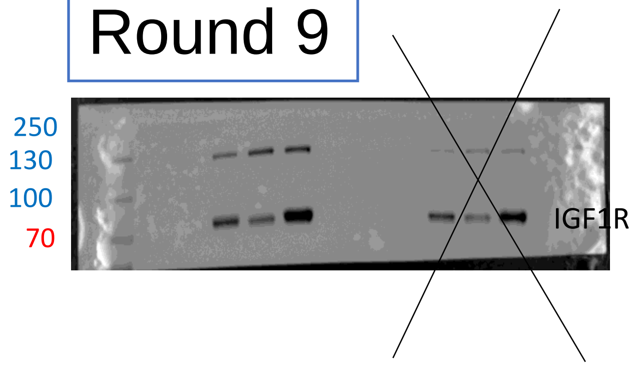

HKE

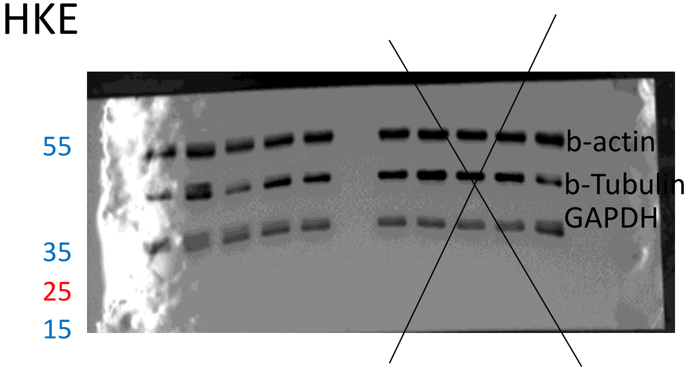

Round 10

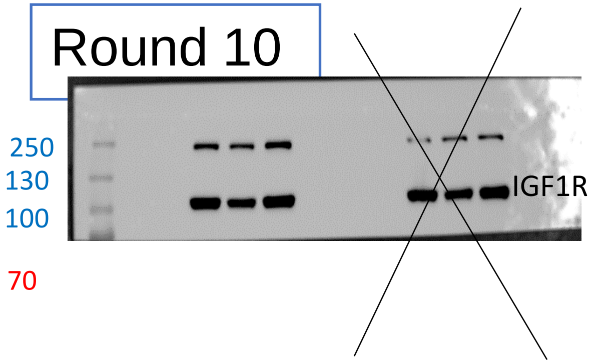

HKE

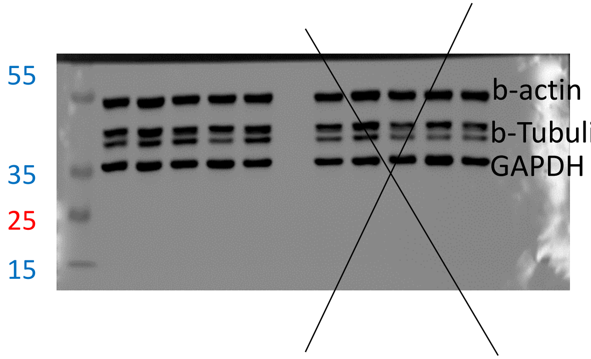

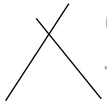

only concerns figure 4

pIGF1R (Y1135) (95kDa) // GAPDH (37kDa)

Round 1

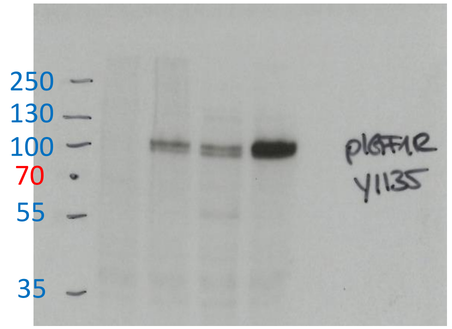

HKE:

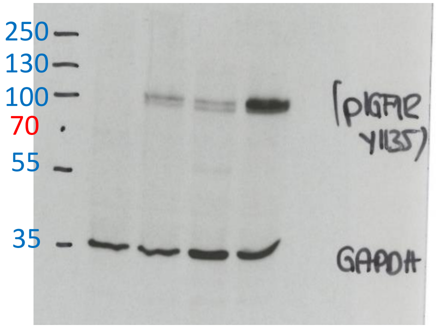

Round 2

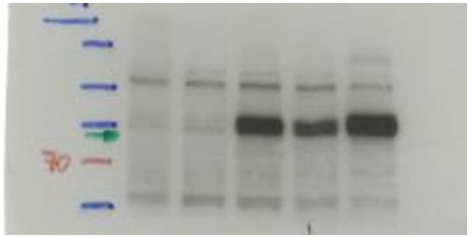

HKE:

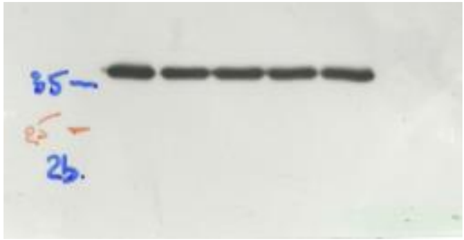

Round 3

Round 4

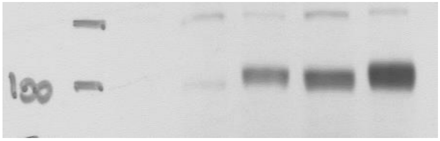

HKE:

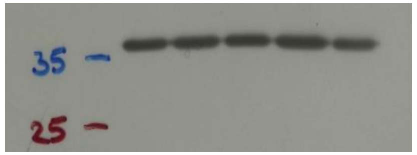

Round 5

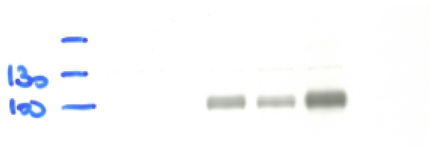

HKE:

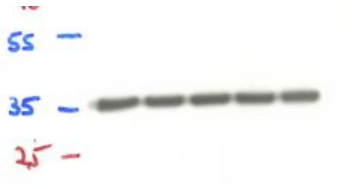

pIGF1R (Y1135) (95kDa) // GAPDH (37kDa)

Round 6

Round 7

Round 8

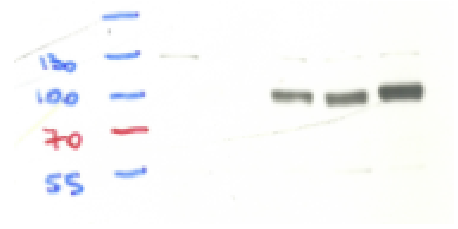

HKE:

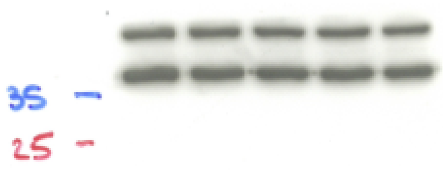

Round 9

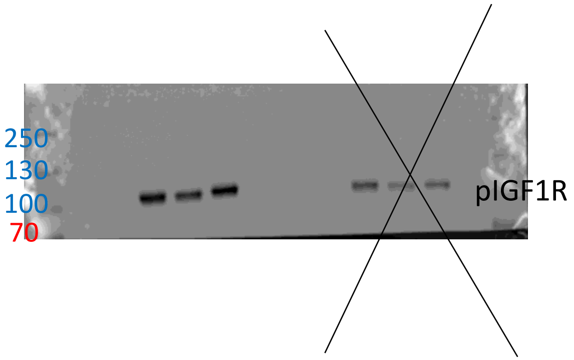

HKE:

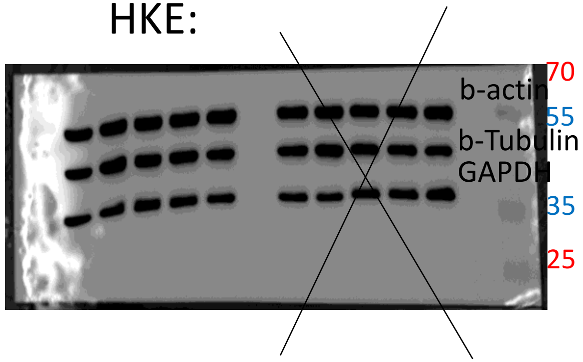

Round 10

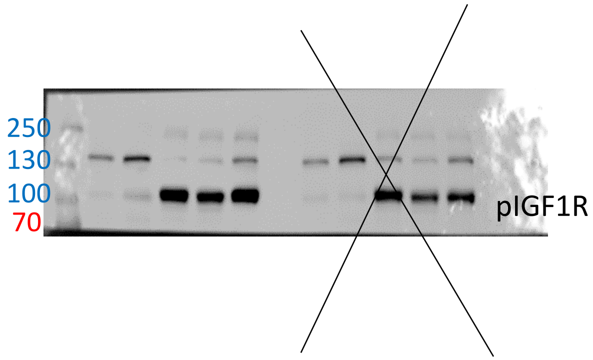

HKE:

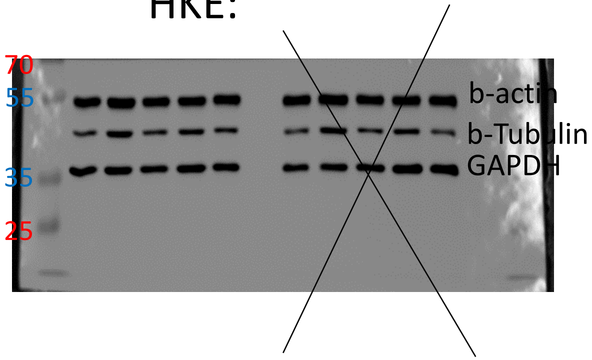

only concerns figure 4

AKT (60kDa) // GAPDH (37kDa)

Round 1

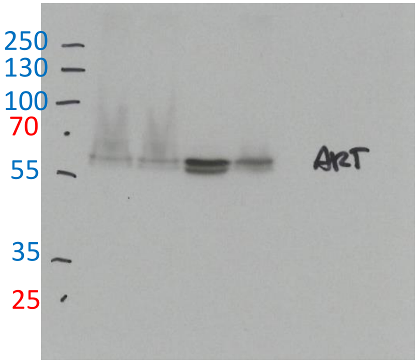

HKE:

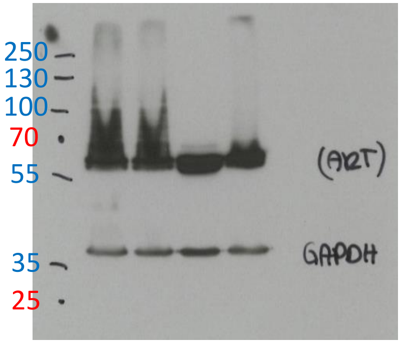

Round 2

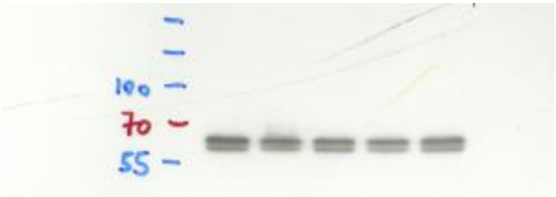

HKE:

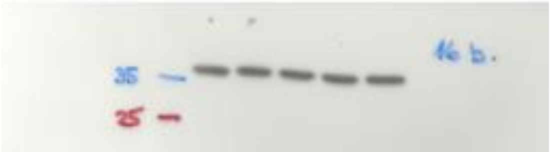

Round 3

Round 4

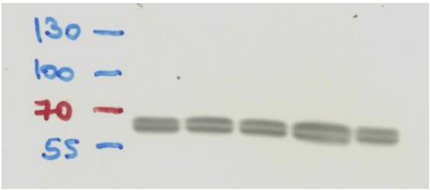

HKE:

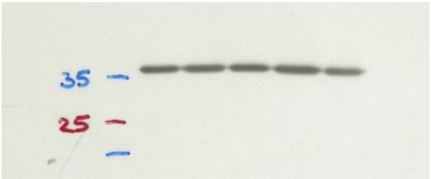

Round 5

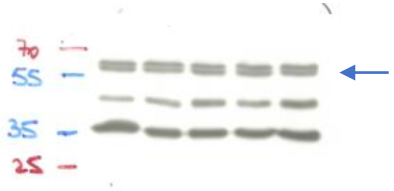

HKE:

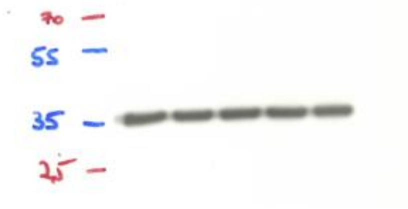

AKT (60kDa) // GAPDH (37kDa)

Round 6

Round 7

Round 8

Round 9

Round 10

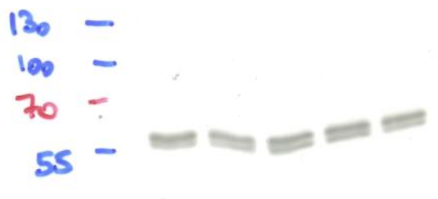

HKE:

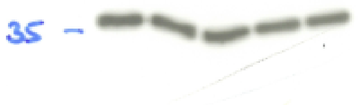

pAKT (60kDa) // GAPDH (37kDa)

Round 1

Round 2

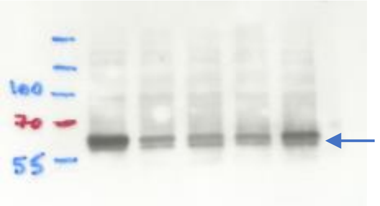

HKE:

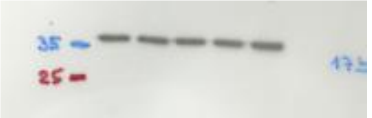

Round 3

HKE:

Round 4

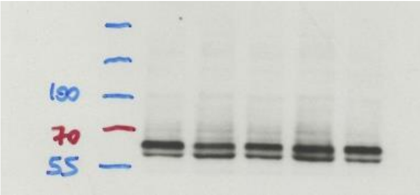

HKE:

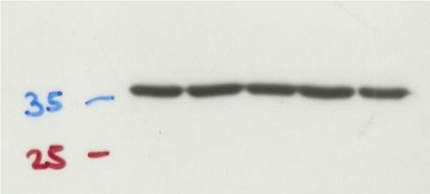

Round 5

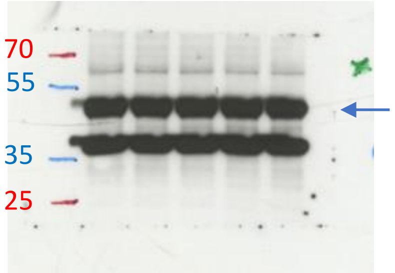

HKE:

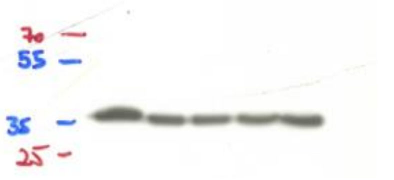

pAKT (60kDa) // GAPDH (37kDa)

Round 6

Round 7

Round 8

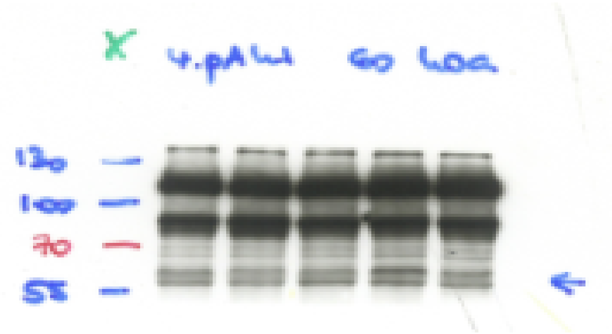

HKE:

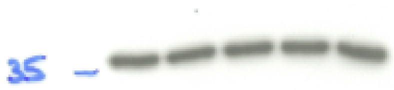

Round 9

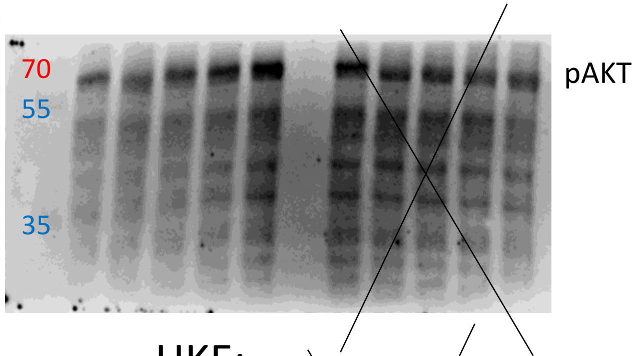

HKE:

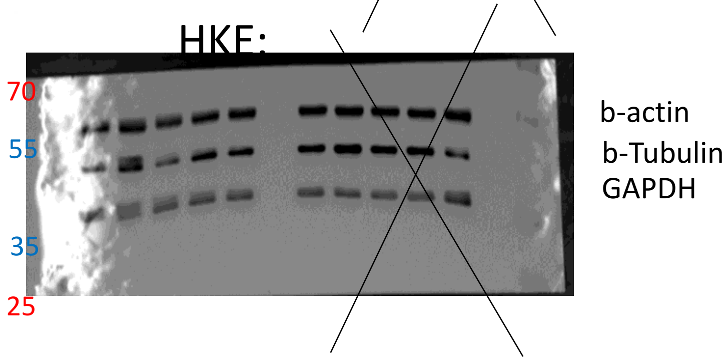

Round 10

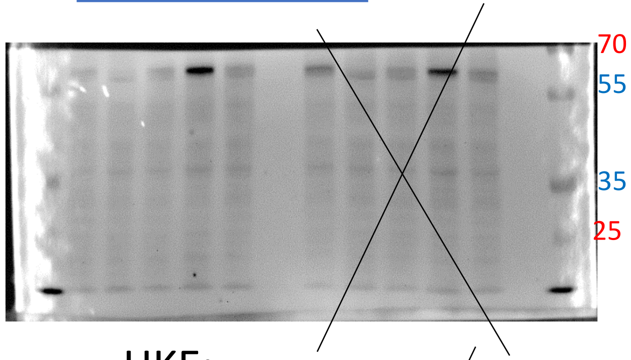

HKE:

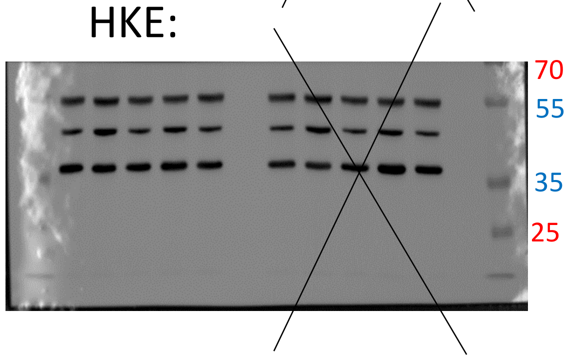

only concerns figure 4

MEK 1/2 (45kDa) // GAPDH (37kDa)

Round 1

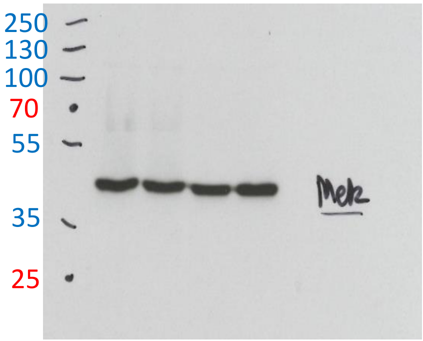

HKE:

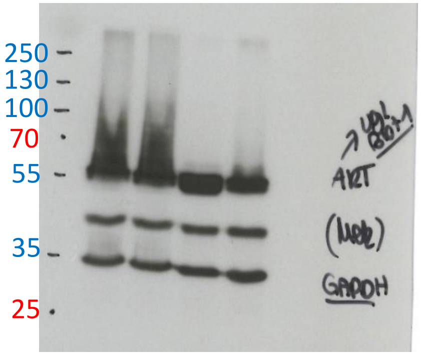

Round 2

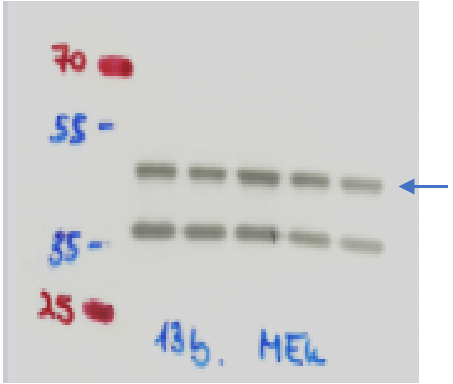

HKE:

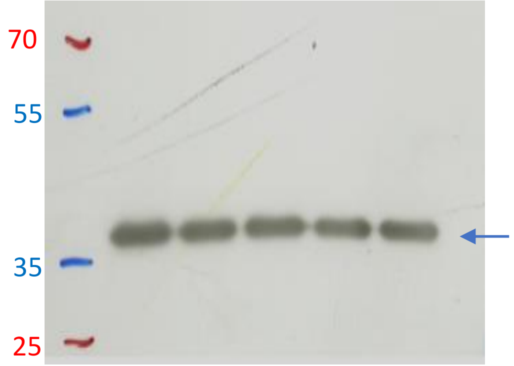

Round 3

Round 4

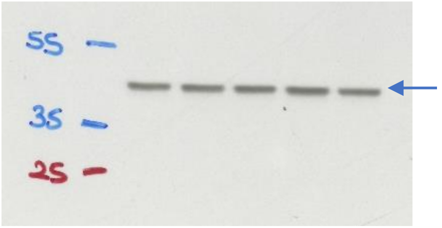

HKE:

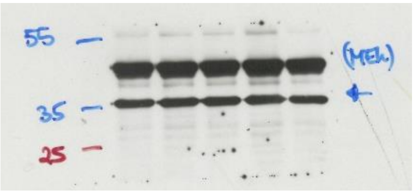

Round 5

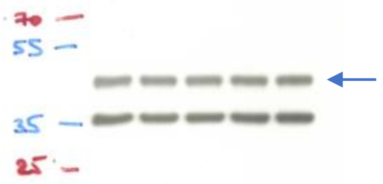

HKE:

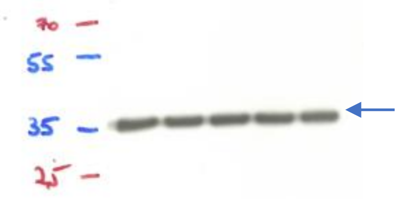

MEK 1/2 (45kDa) // GAPDH (37kDa)

Round 6

Round 7

Round 8

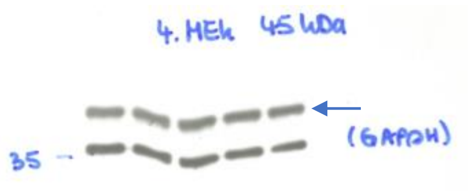

HKE:

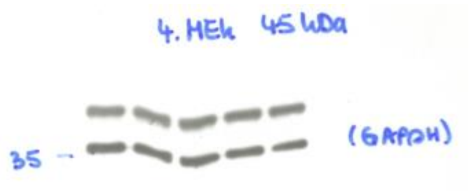

Round 9

Round 10

pMEK 1/2 (45kDa) // GAPDH (37kDa)

Round 1

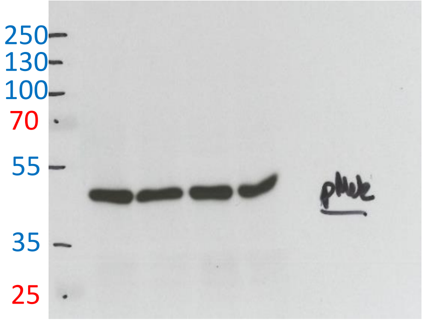

HKE:

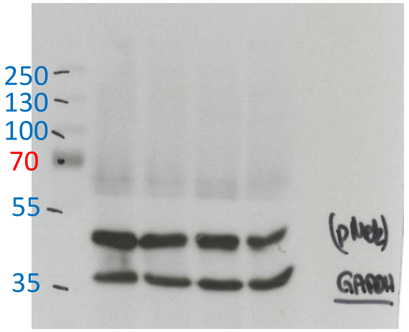

Round 2

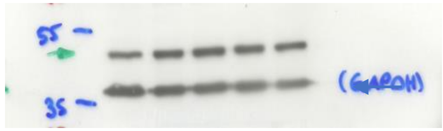

HKE:

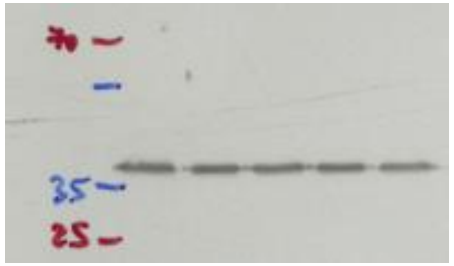

Round 3

Round 4

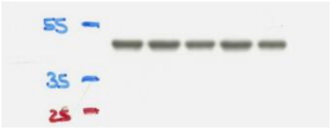

HKE:

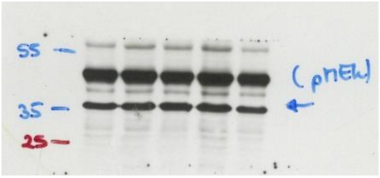

Round 5

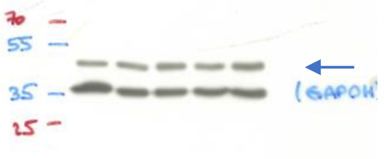

HKE:

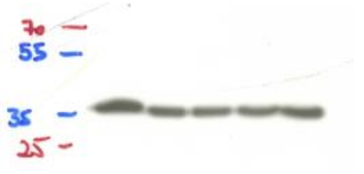

pMEK 1/2 (45kDa) // GAPDH (37kDa)

Round 6

Round 7

Round 8

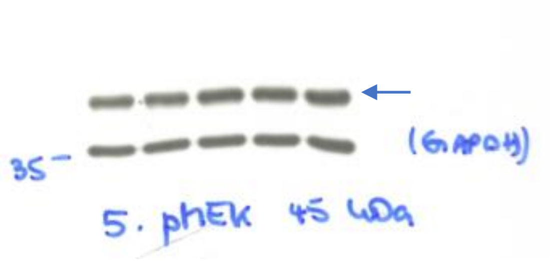

HKE:

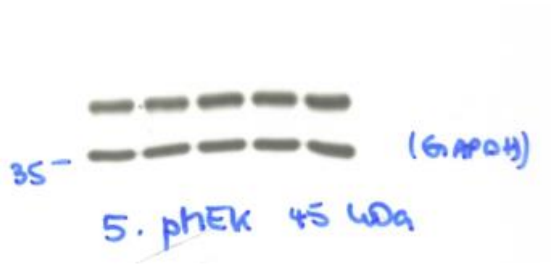

Round 9

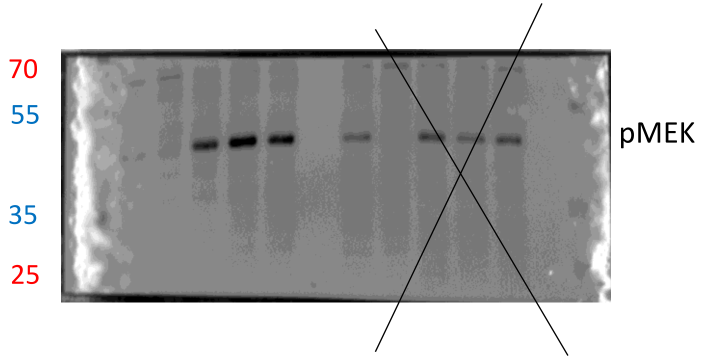

HKE:

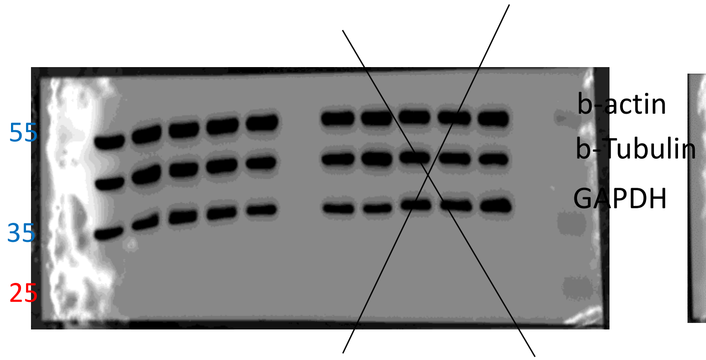

Round 10

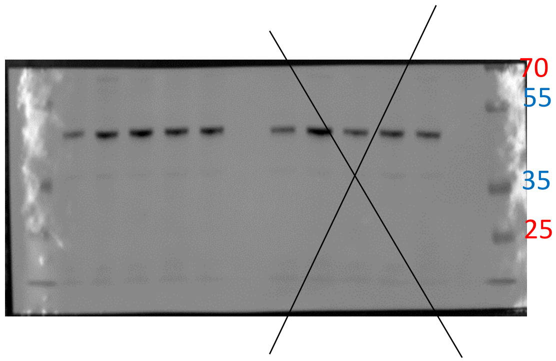

HKE:

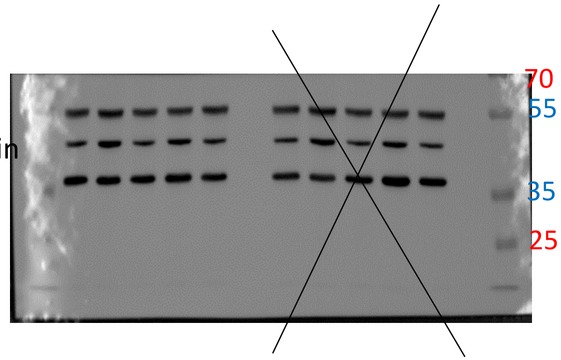

only concerns figure 4

ERK 1/2 (45kDa) // GAPDH (37kDa)

Round 1

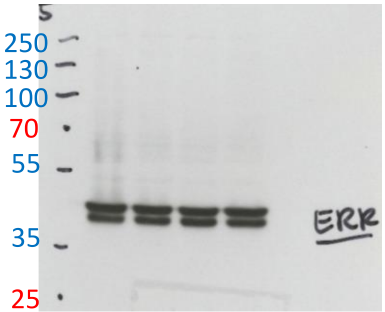

HKE:

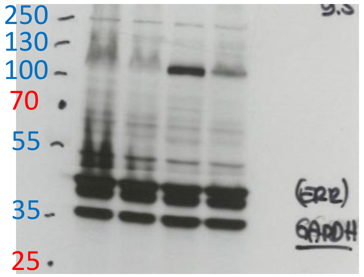

Round 2

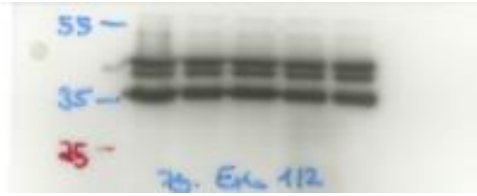

HKE:

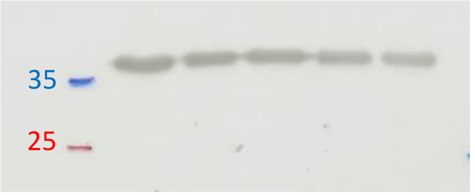

Round 3

Round 4

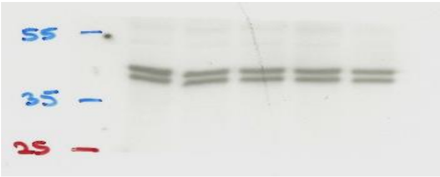

HKE:

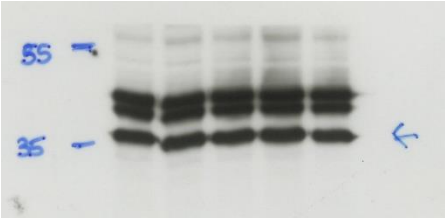

Round 5

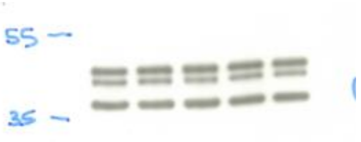

HKE:

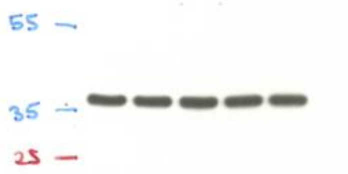

ERK 1/2 (45kDa) // GAPDH (37kDa)

Round 6

Round 7

Round 8

Round 9

Round 10

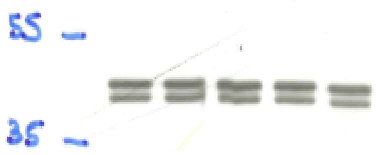

HKE:

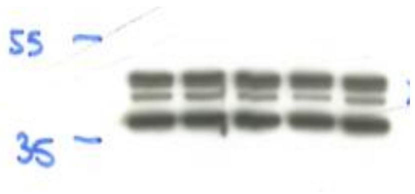

pERK 1/2 (45kDa) // GAPDH (37kDa)

Round 1

Round 2

Round 3

Round 4

Round 5

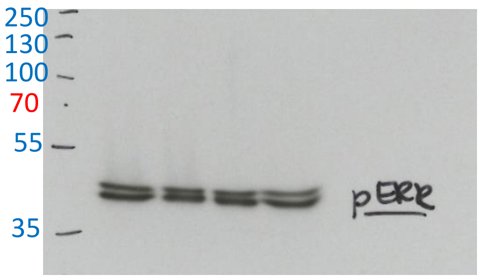

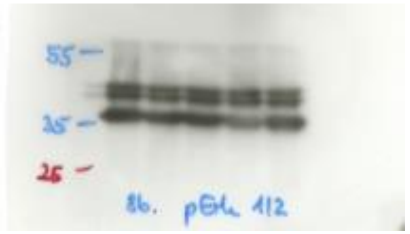

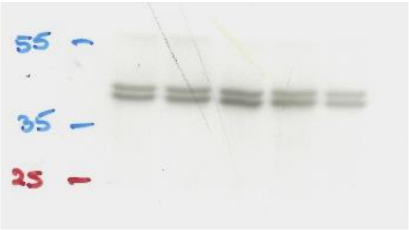

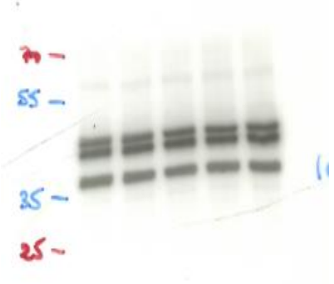

HKE:

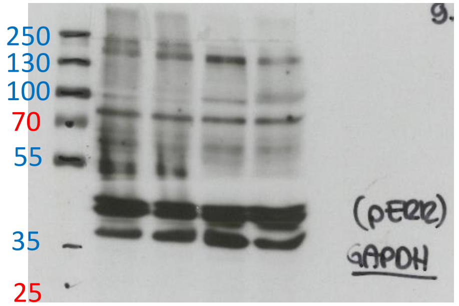

HKE:

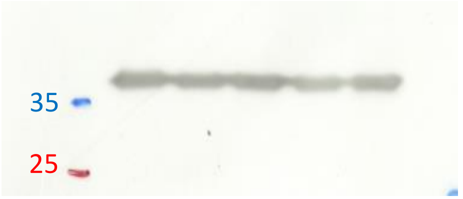

HKE:

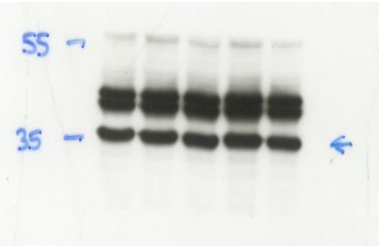

HKE:

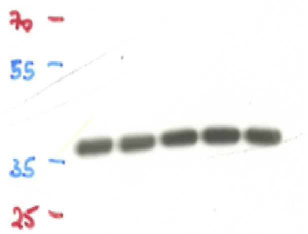

pERK 1/2 (45kDa) // GAPDH (37kDa)

Round 6

Round 7

Round 8

Round 9

Round 10

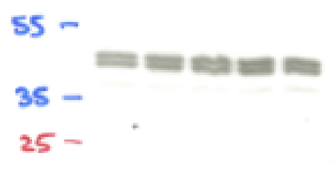

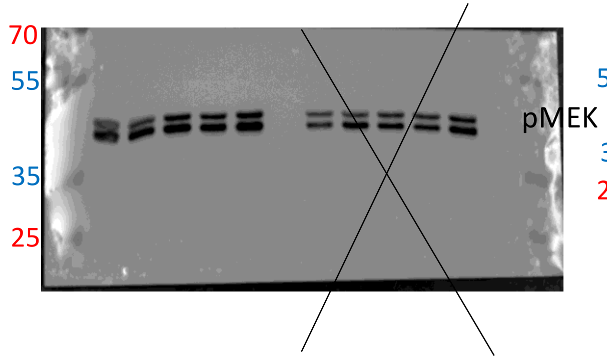

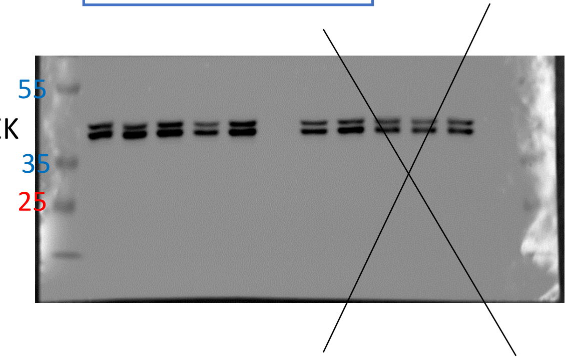

HKE:

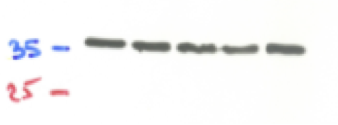

HKE:

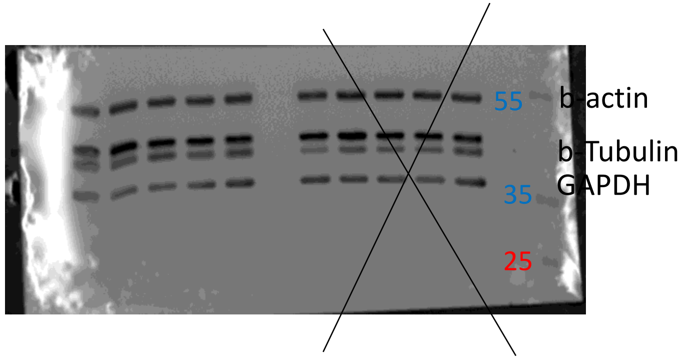

HKE:

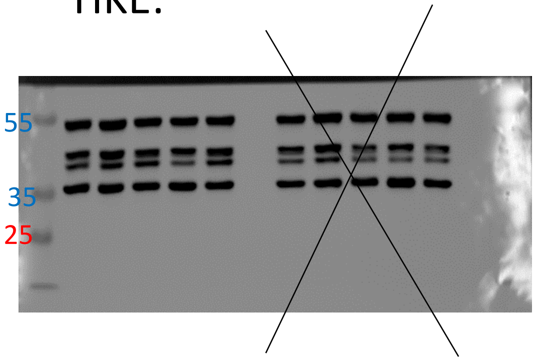

only concerns
figure 4

Figure 2A (JJN-3)

Round 1-3:

Loading: JJN-3 | EV | IGF1R^{WT} | IGF1R^{D1146N} | IGF1R^{N1129S}

Round 4-6:

Loading: JJN-3 | EV | IGF1R^{WT} | IGF1R^{D1146N} | IGF1R^{N1129S}

culturing medium

~~JJN-3 | EV | IGF1R^{WT} | IGF1R^{D1146N} | IGF1R^{N1129S}~~

~~starving medium~~

only concerns Figure 4

for AKT, MEK & ERK (Round 5,6): normal loading

Loading: JJN-3 | EV | IGF1R^{WT} | IGF1R^{D1146N} | IGF1R^{N1129S}

IGF1R (95kDa) // GAPDH (37kDa)

Round 1

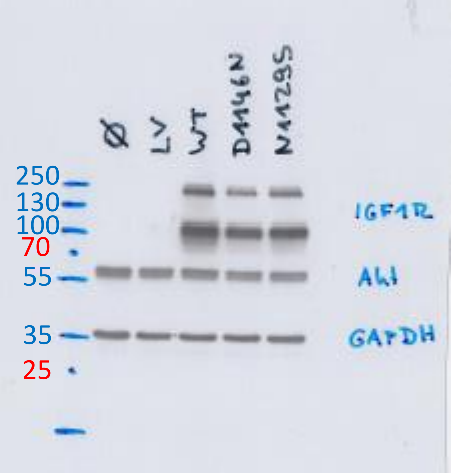

HKE:

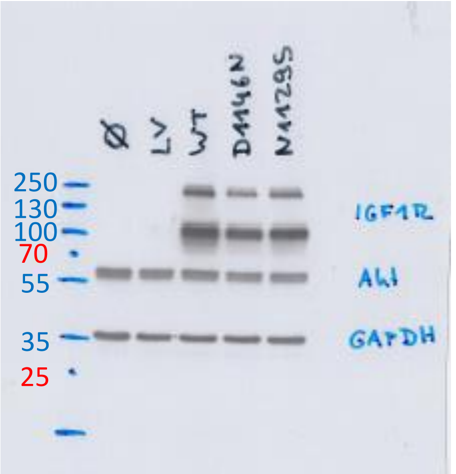

Round 2

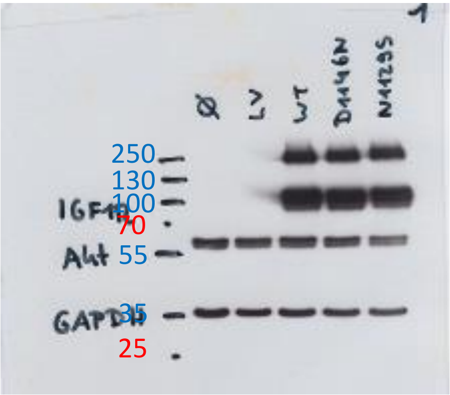

HKE:

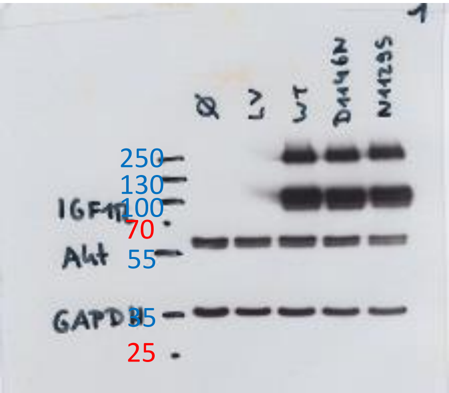

Round 3

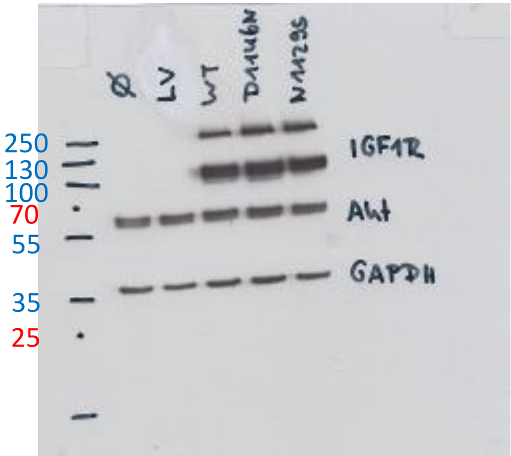

HKE:

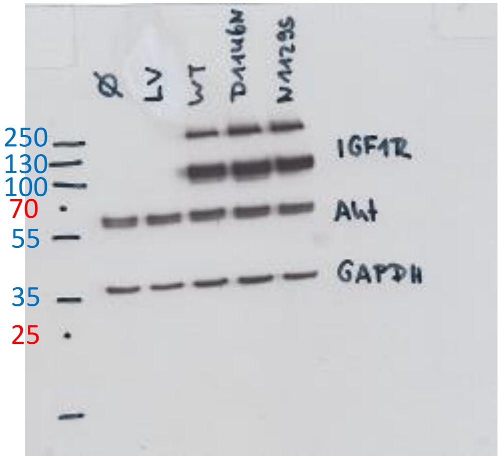

IGF1R (95kDa) // GAPDH (37kDa)

Round 4

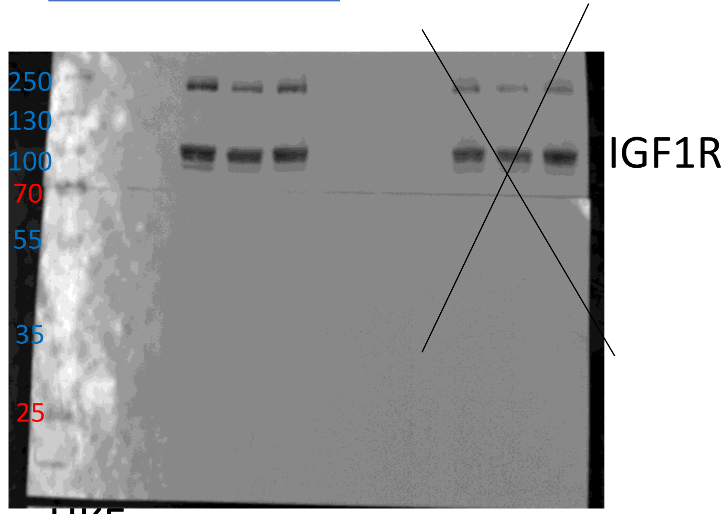

HKE:

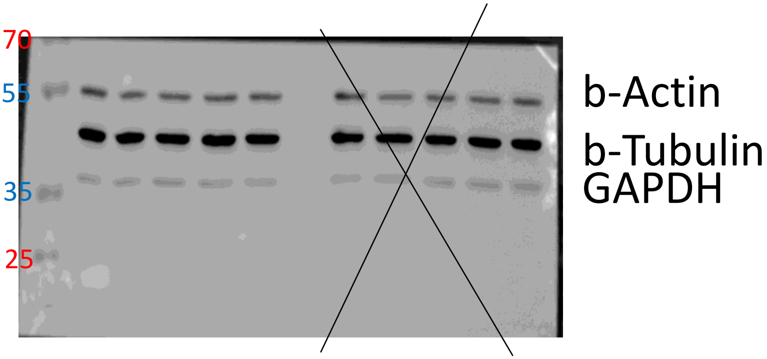

Round 5

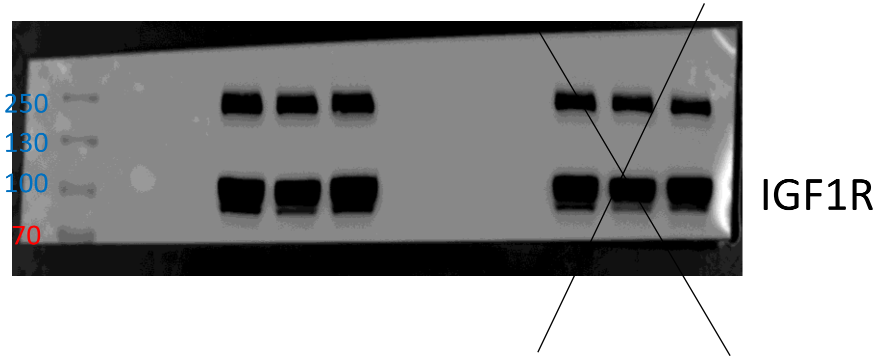

HKE:

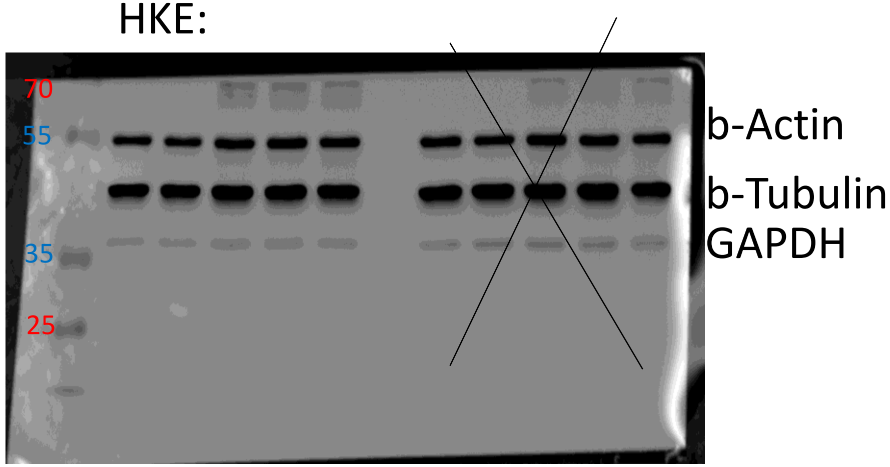

Round 6

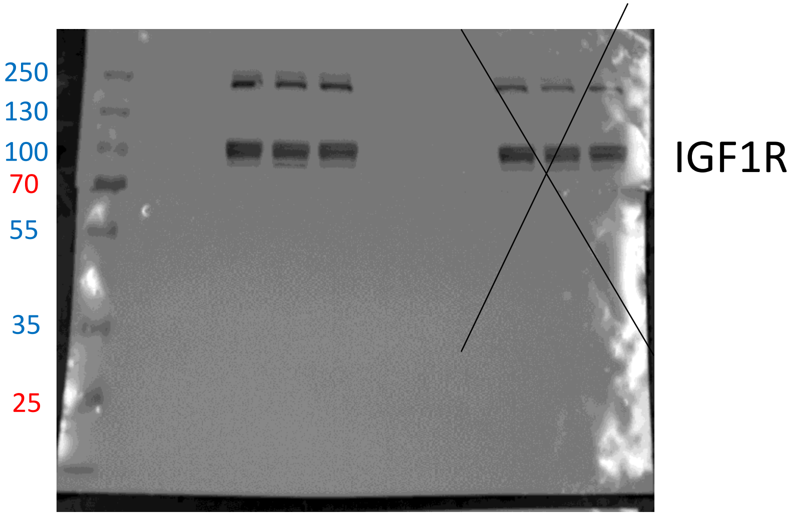

HKE:

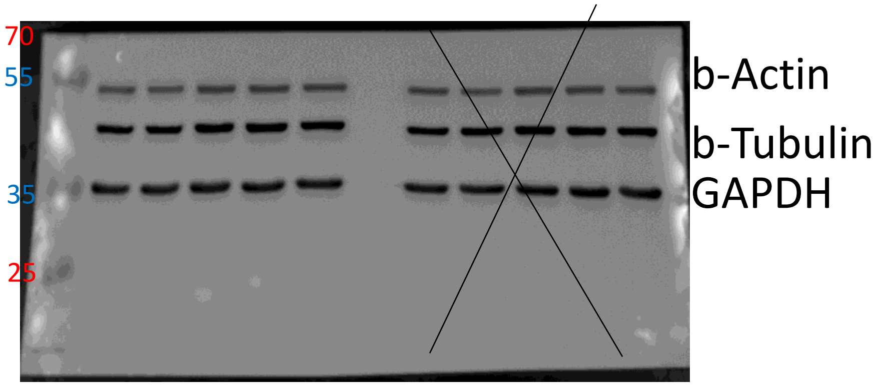

only concerns
figure 4

pIGF1R (Y1135) (95kDa) // GAPDH (37kDa)

Round 1

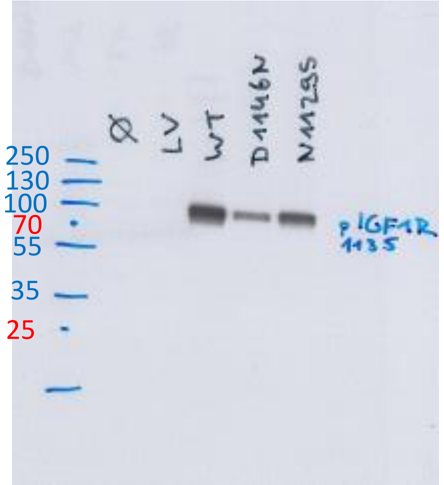

HKE:

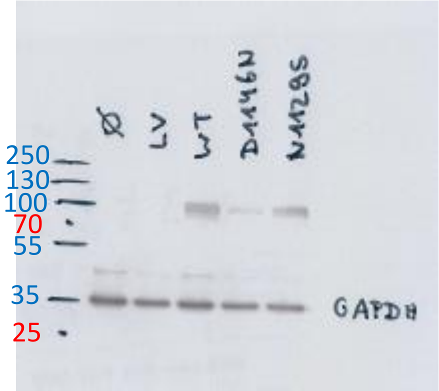

Round 2

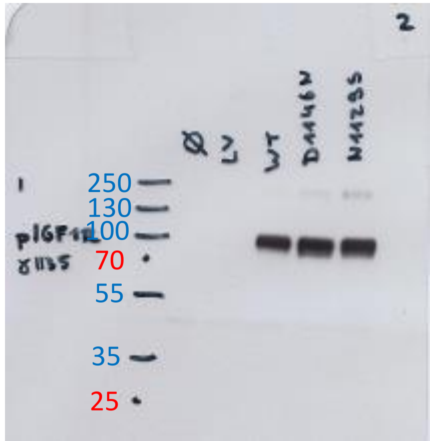

HKE:

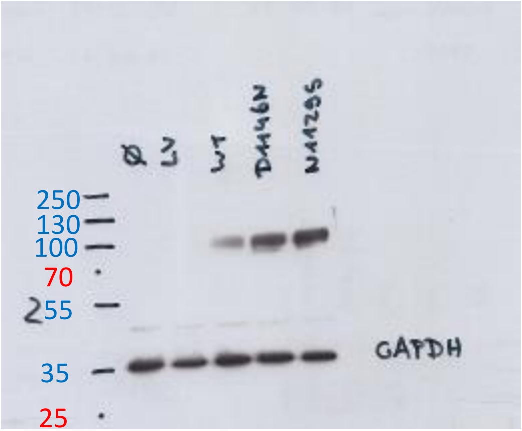

Round 3

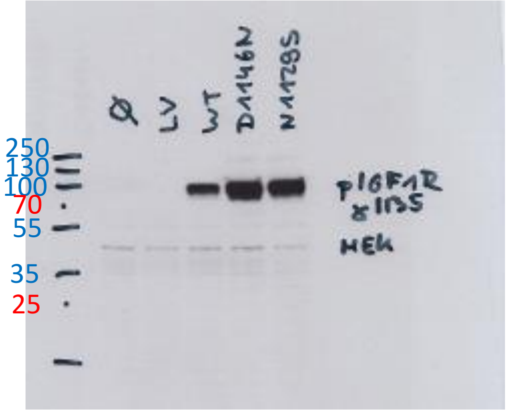

HKE:

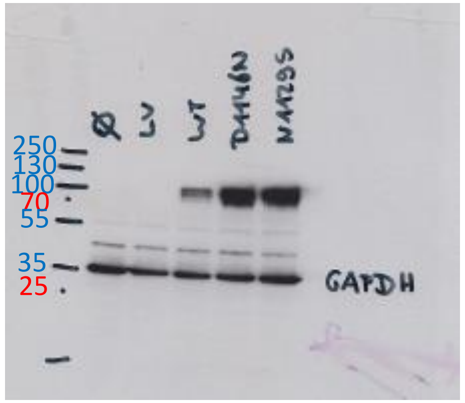

pIGF1R (Y1135) (95kDa) // GAPDH (37kDa)

Round 4 | Round 5 | Round 6

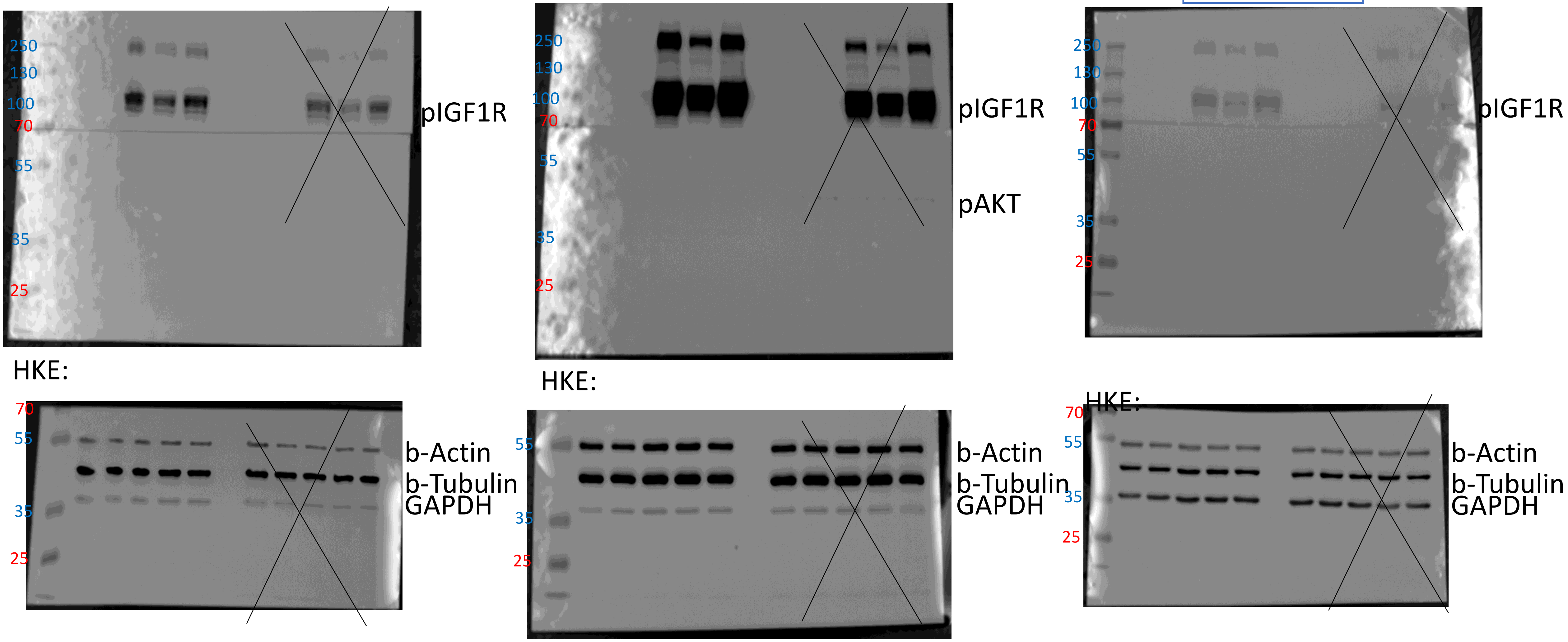

X only concerns figure 4

AKT (60kDa) // GAPDH (37kDa)

Round 1

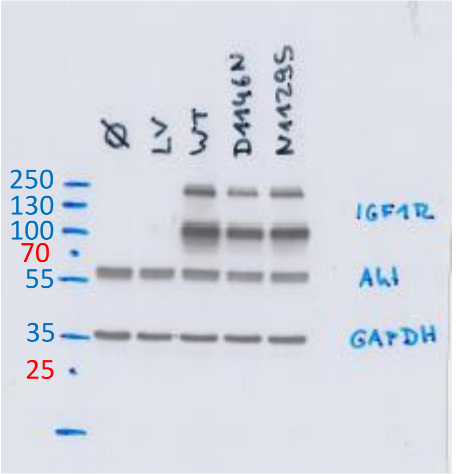

Round 2

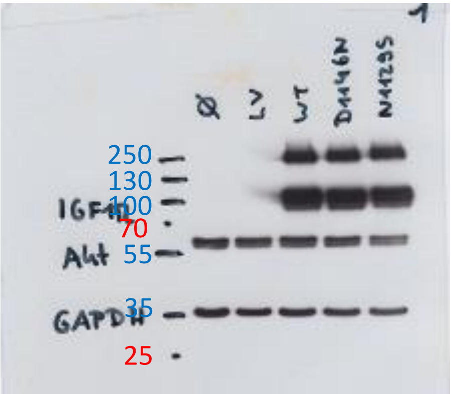

Round 3

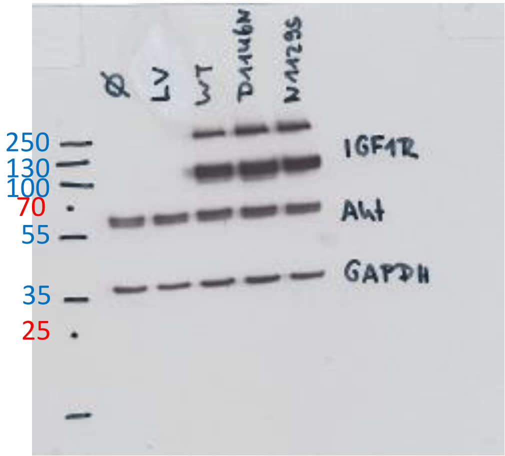

HKE:

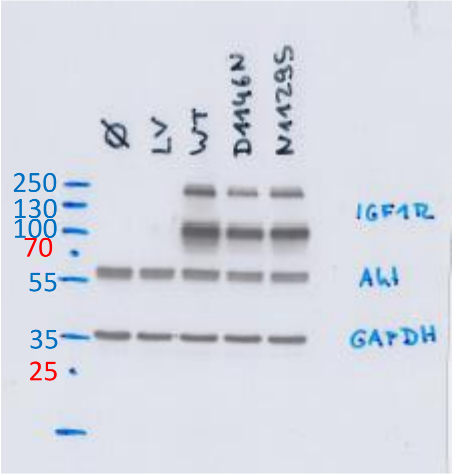

HKE:

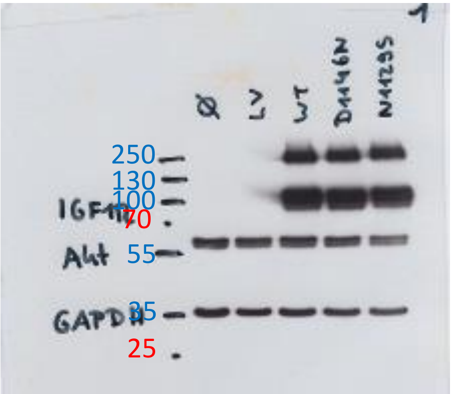

HKE:

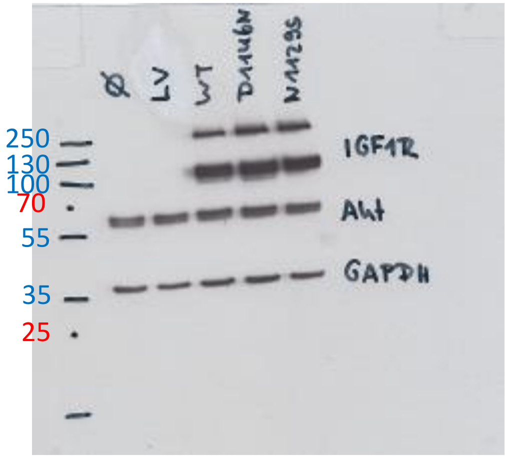

AKT (60kDa) // GAPDH (37kDa)

Round 4

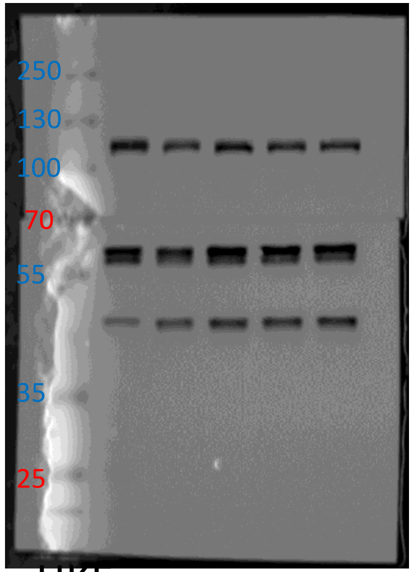

Pyk2
AKT
MEK

HKE:

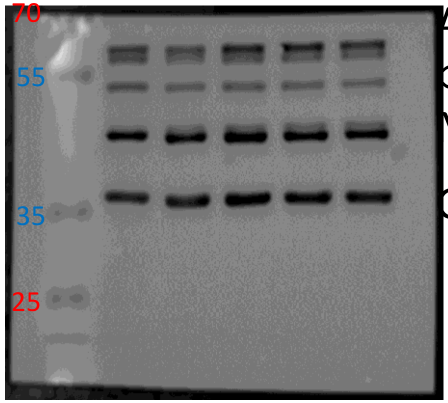

AKT
b-Tubulin
MEK
GAPDH

Round 5

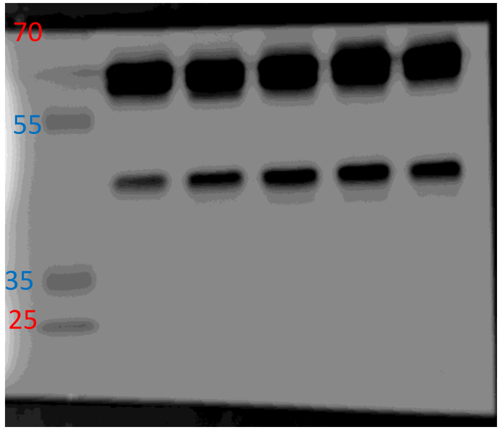

AKT
MEK

HKE:

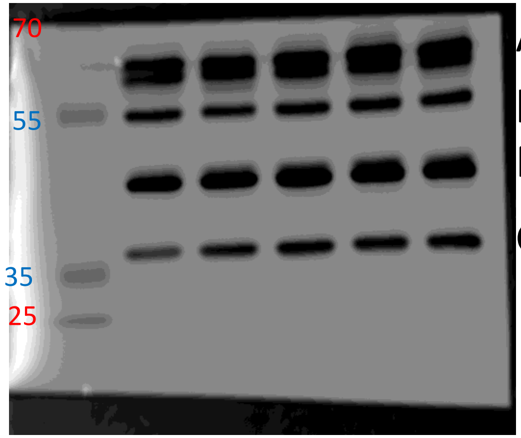

AKT
b-Tubulin
MEK
GAPDH

Round 6

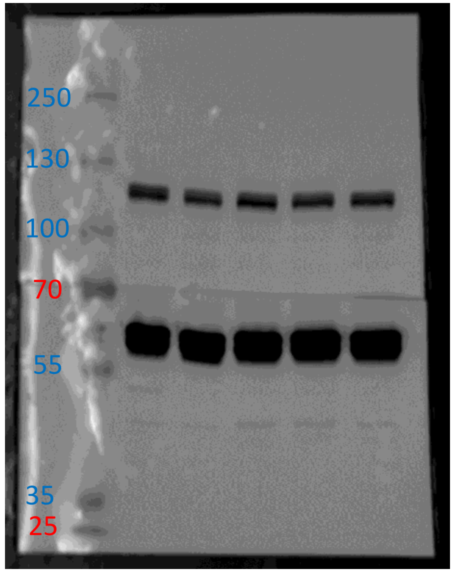

Pyk2
AKT

HKE:

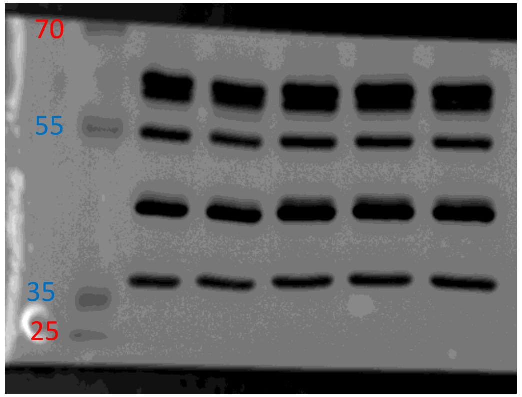

AKT
b-Tubulin
MEK
GAPDH

pAKT (60kDa) // GAPDH (37kDa)

Round 1

Round 2

Round 3

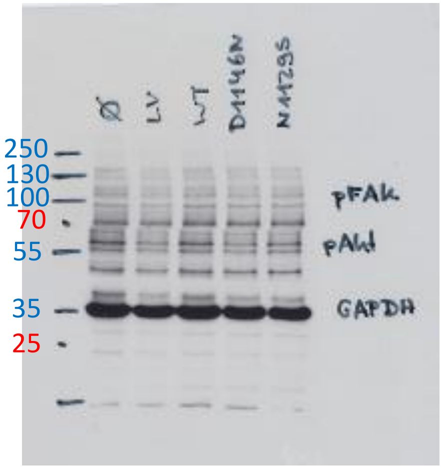

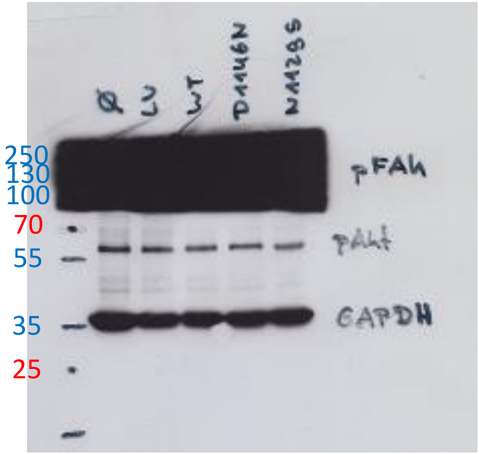

HKE:

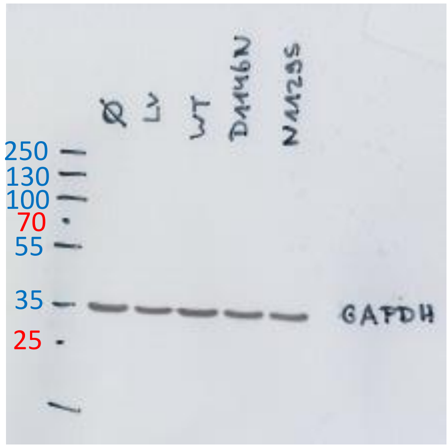

HKE:

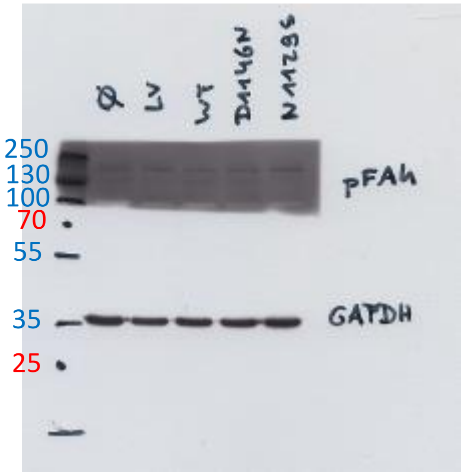

pAKT (60kDa) // GAPDH (37kDa)

Round 4

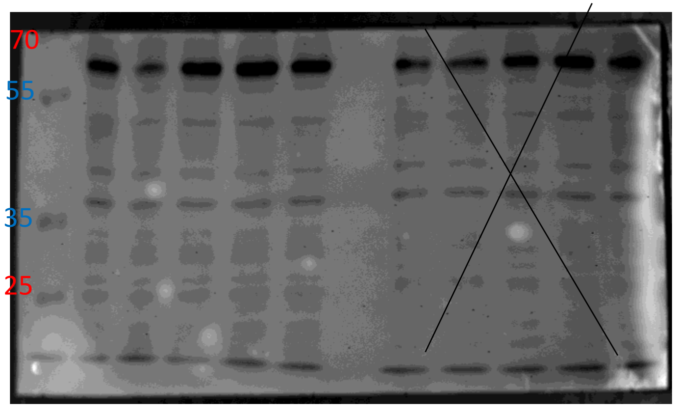

pAKT

HKE:

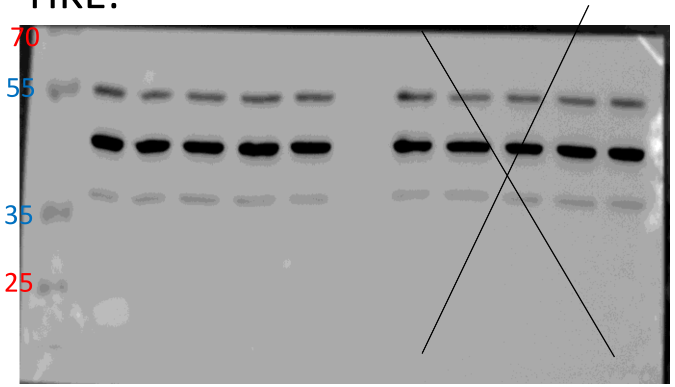

b-Actin
b-Tubulin
GAPDH

Round 5

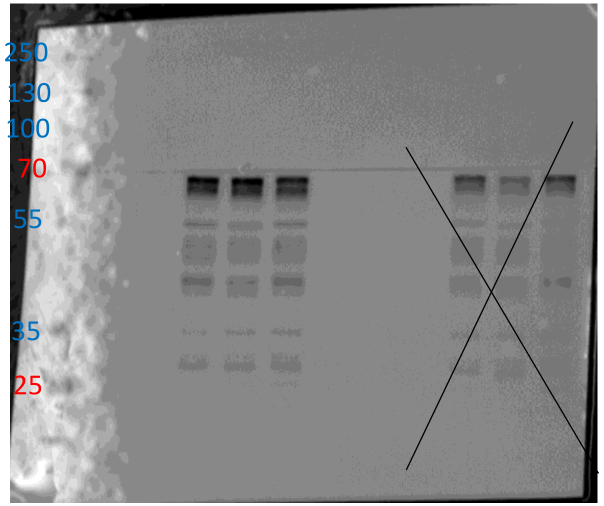

pAKT

HKE:

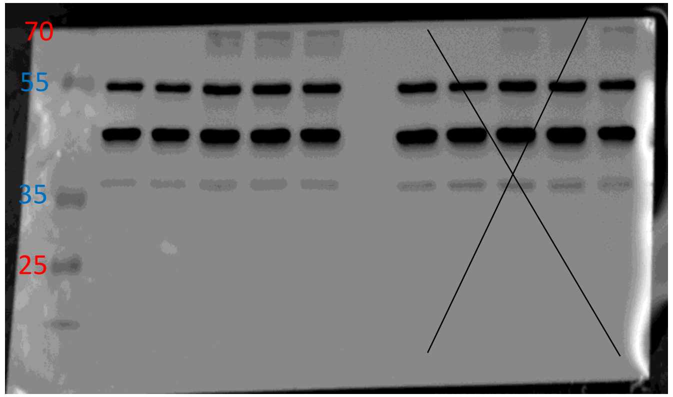

b-Actin
b-Tubulin
GAPDH

Round 6

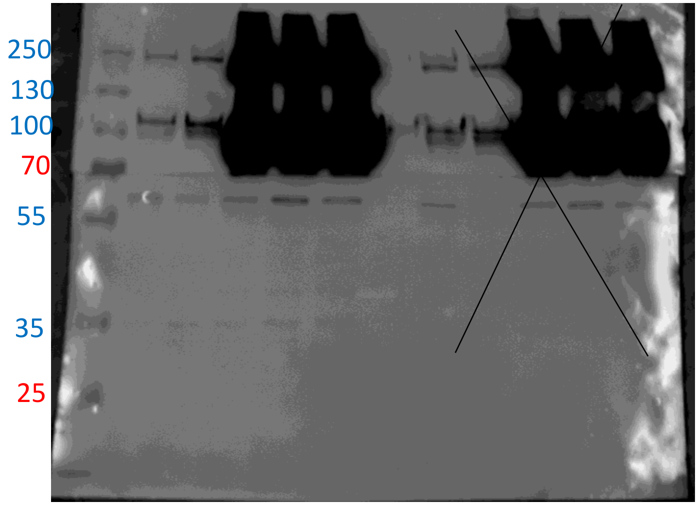

IGF1R
pAKT

HKE:

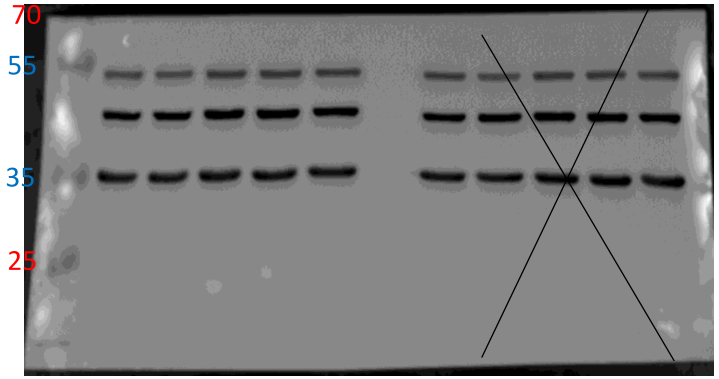

only concerns
figure 4

MEK 1/2 (45kDa) // GAPDH (37kDa)

Round 1

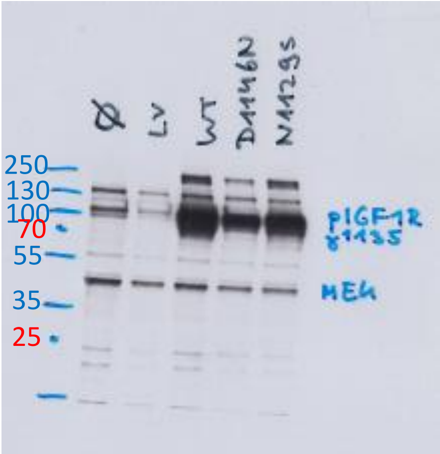

Round 2

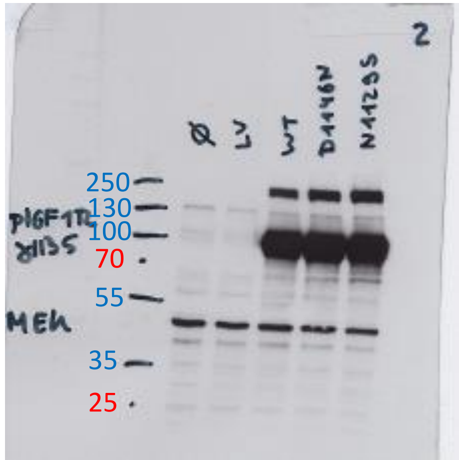

Round 3

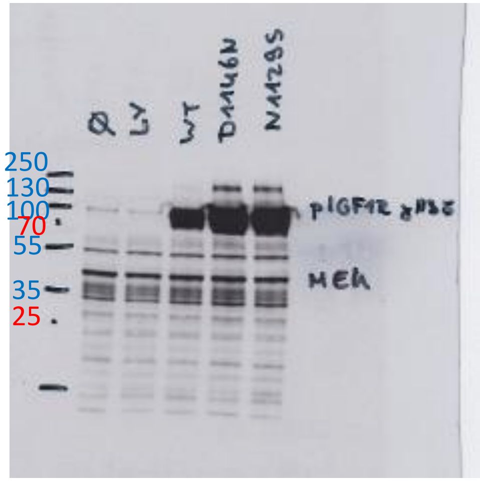

HKE:

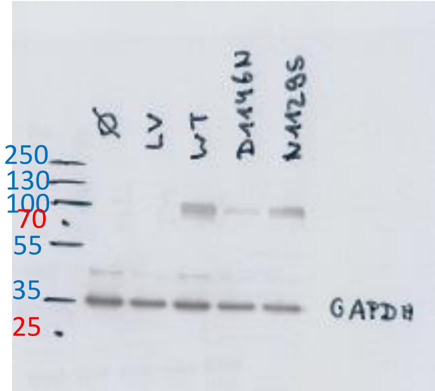

HKE:

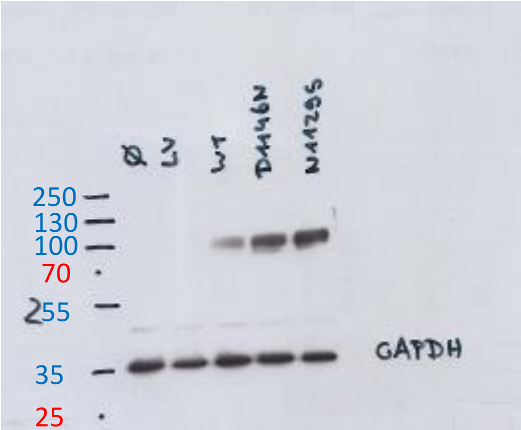

HKE:

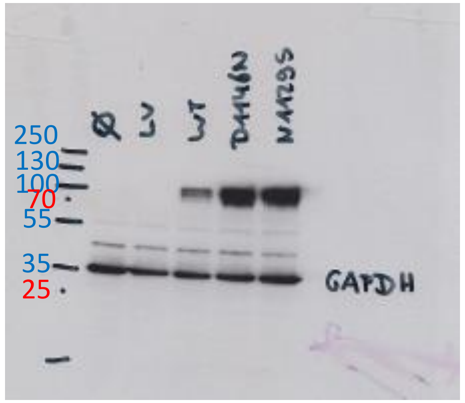

MEK 1/2 (45kDa) // GAPDH (37kDa)

Round 4

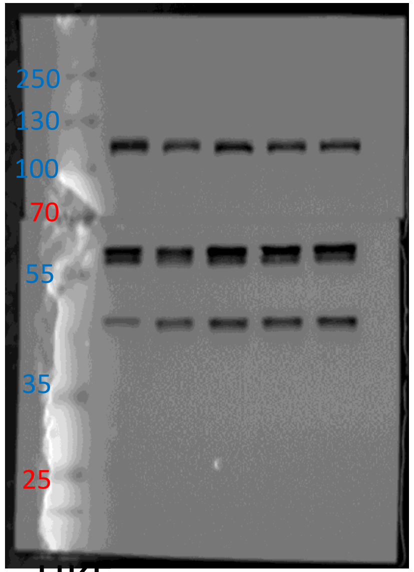

Pyk2

AKT

MEK

HKE:

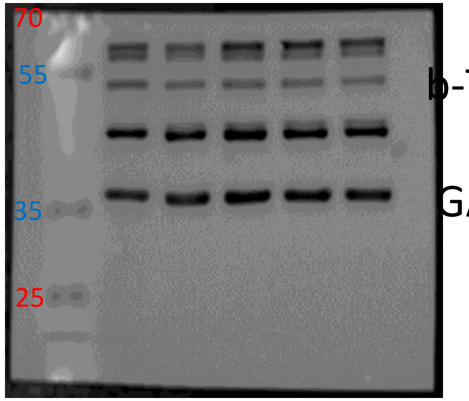

b-Tubulin

GAPDH

Round 5

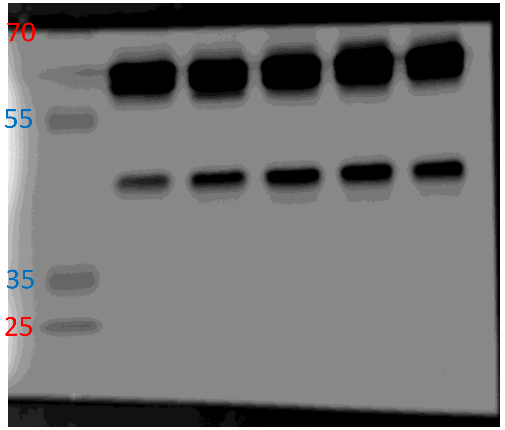

AKT

MEK

HKE:

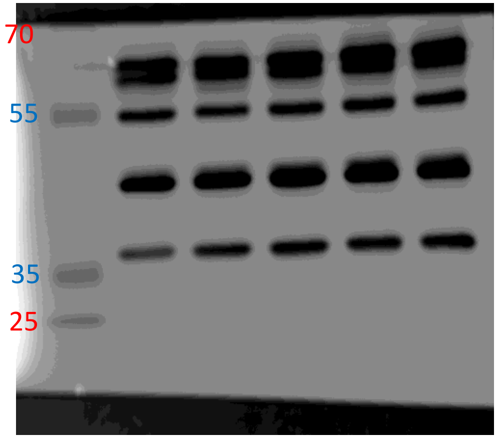

AKT

b-Tubulin

MEK

GAPDH

Round 6

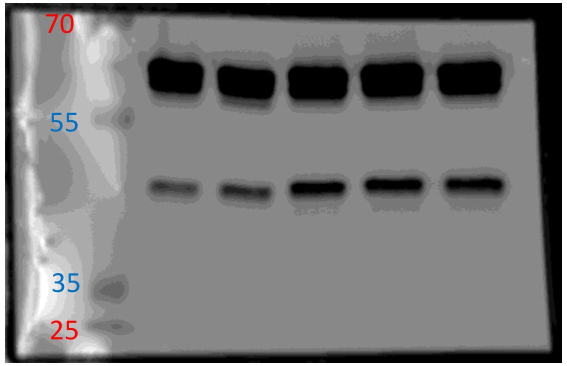

AKT

MEK

HKE:

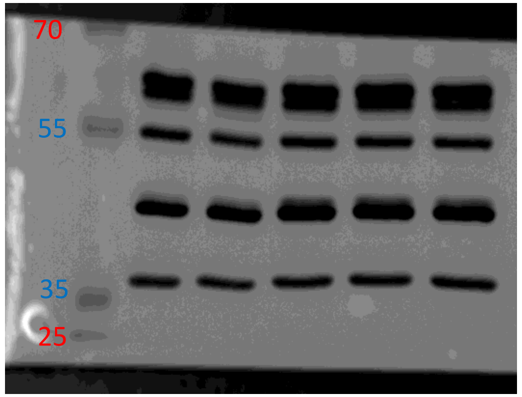

AKT

b-Tubulin

MEK

GAPDH

pMEK 1/2 (45kDa) // GAPDH (37kDa)

Round 1

Round 2

Round 3

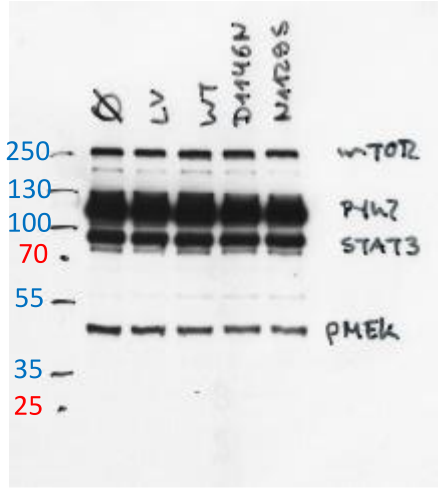

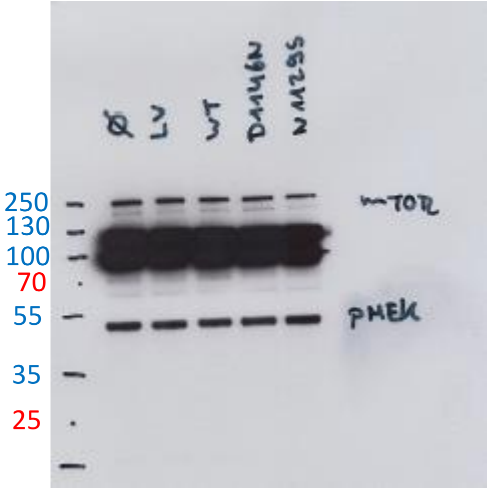

HKE:

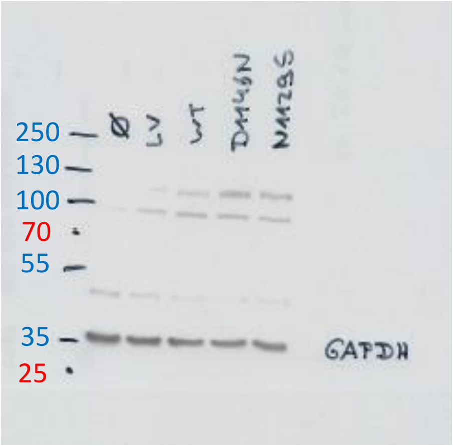

HKE:

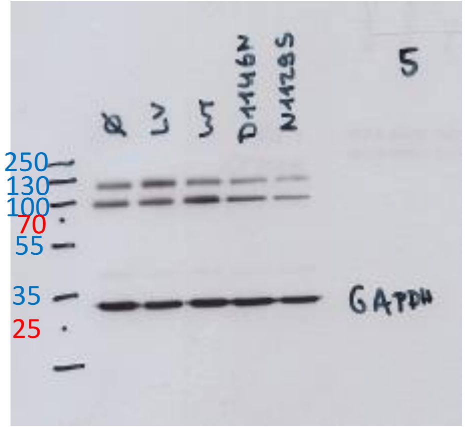

pMEK 1/2 (45kDa) // GAPDH (37kDa)

Round 4

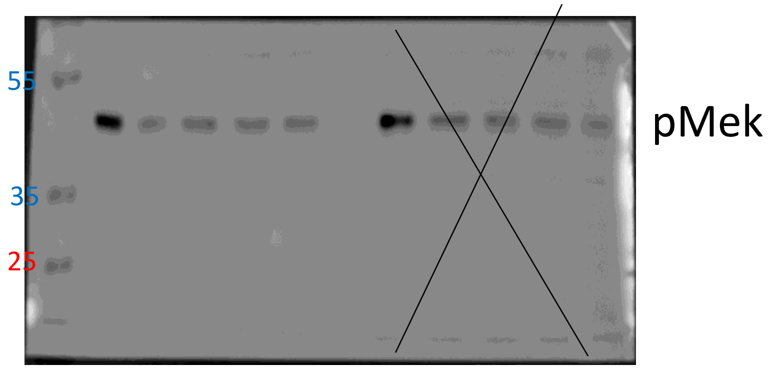

HKE:

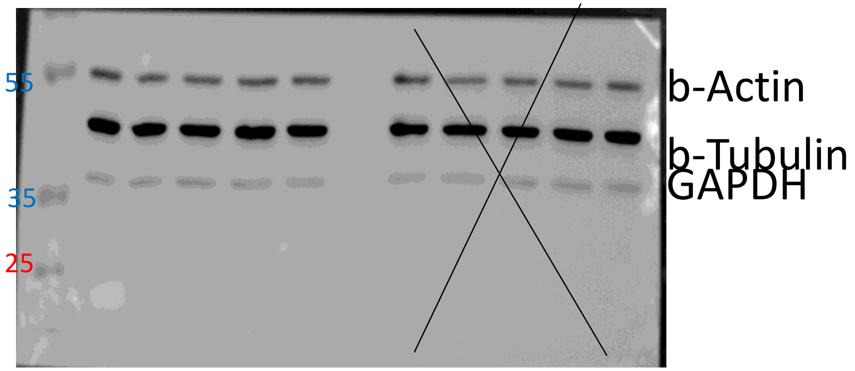

Round 5

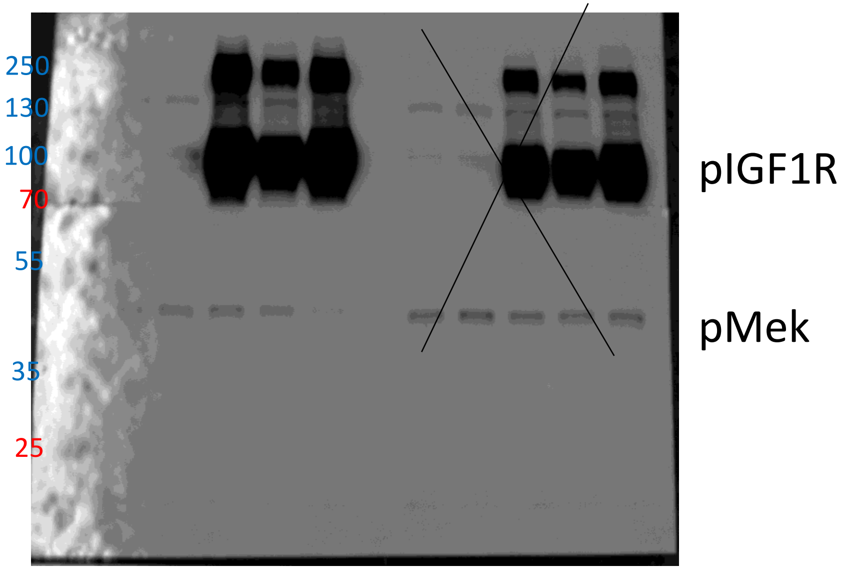

HKE:

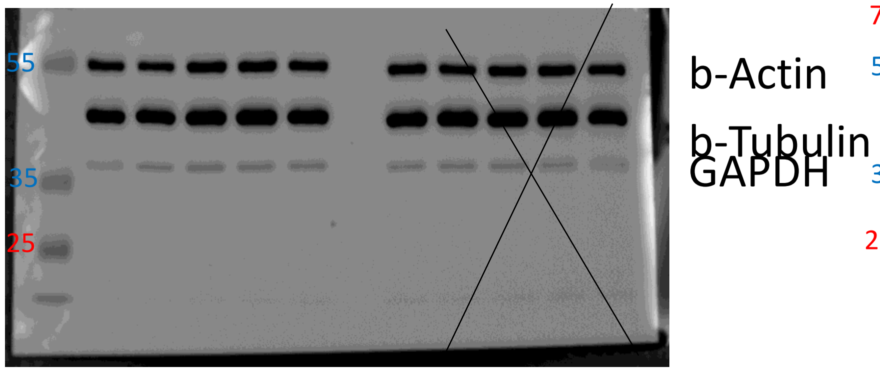

Round 6

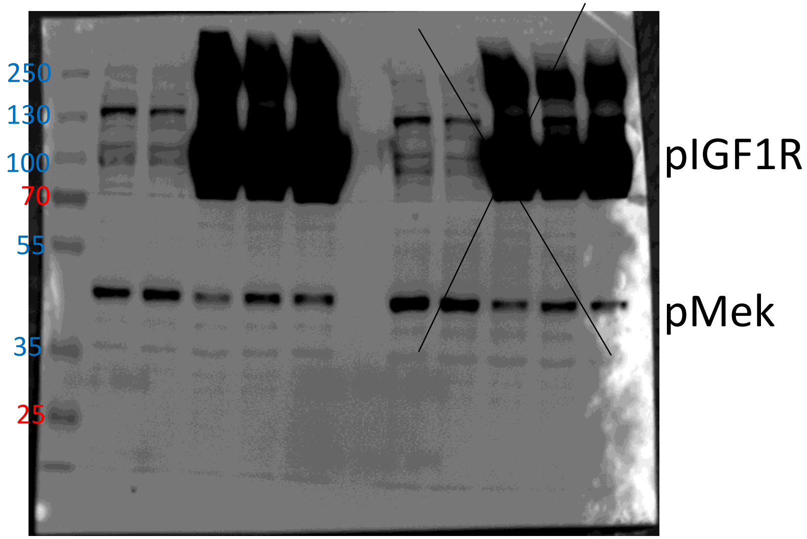

HKE:

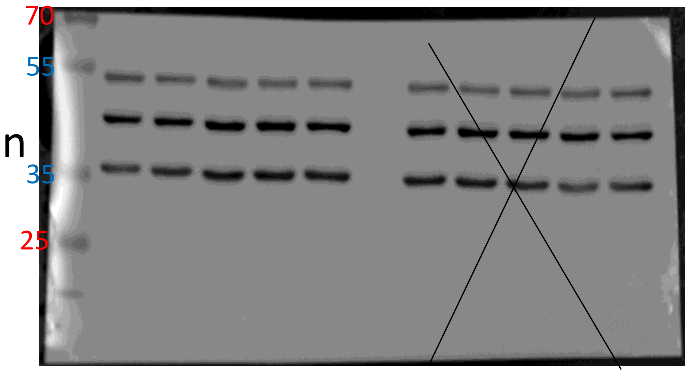

only concerns figure 4

ERK 1/2 (45kDa) // GAPDH (37kDa)

Round 1

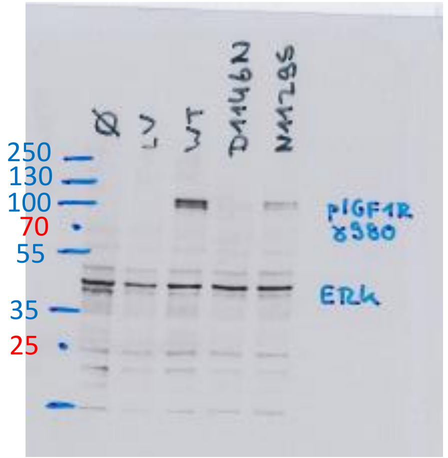

HKE:

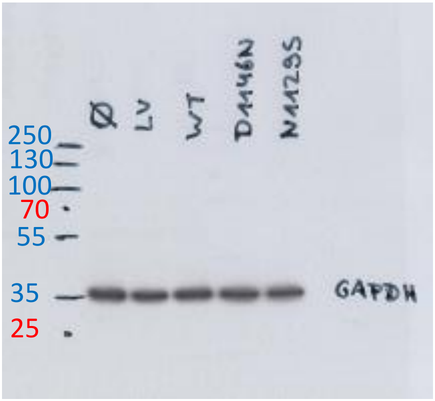

Round 2

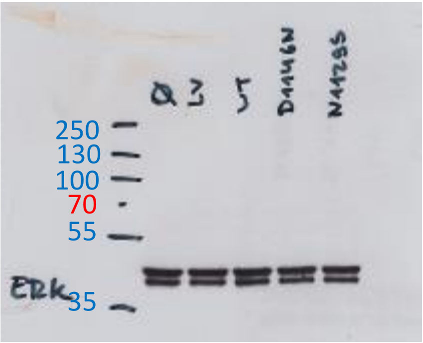

HKE:

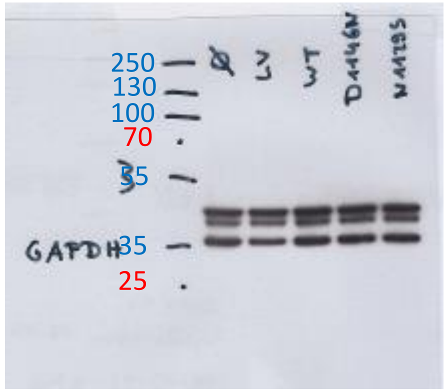

Round 3

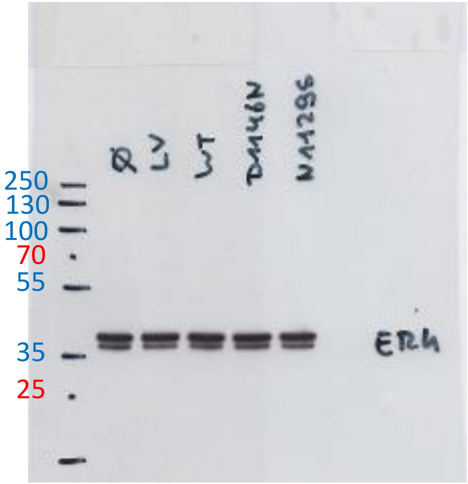

HKE:

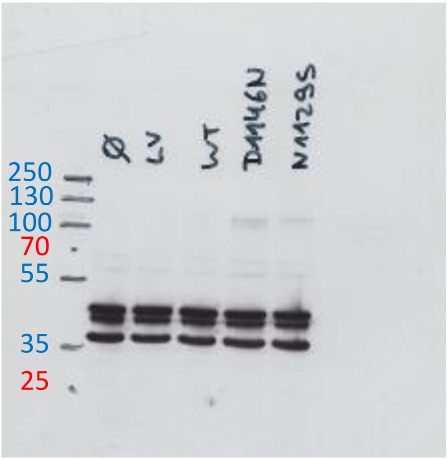

ERK 1/2 (45kDa) // GAPDH (37kDa)

Round 4

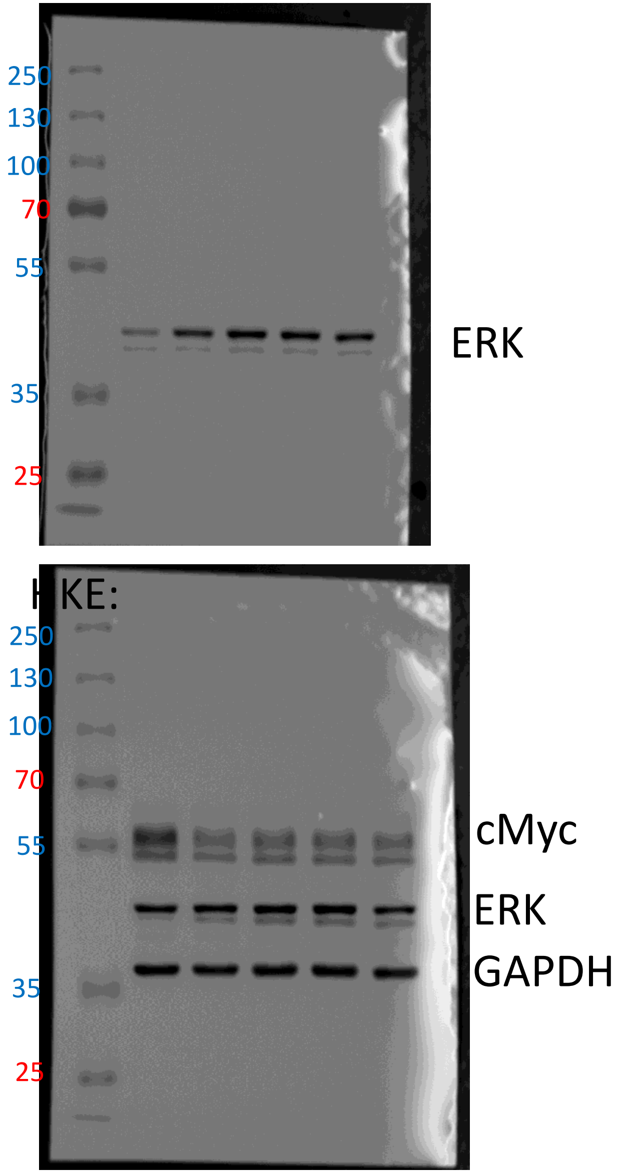

Round 5

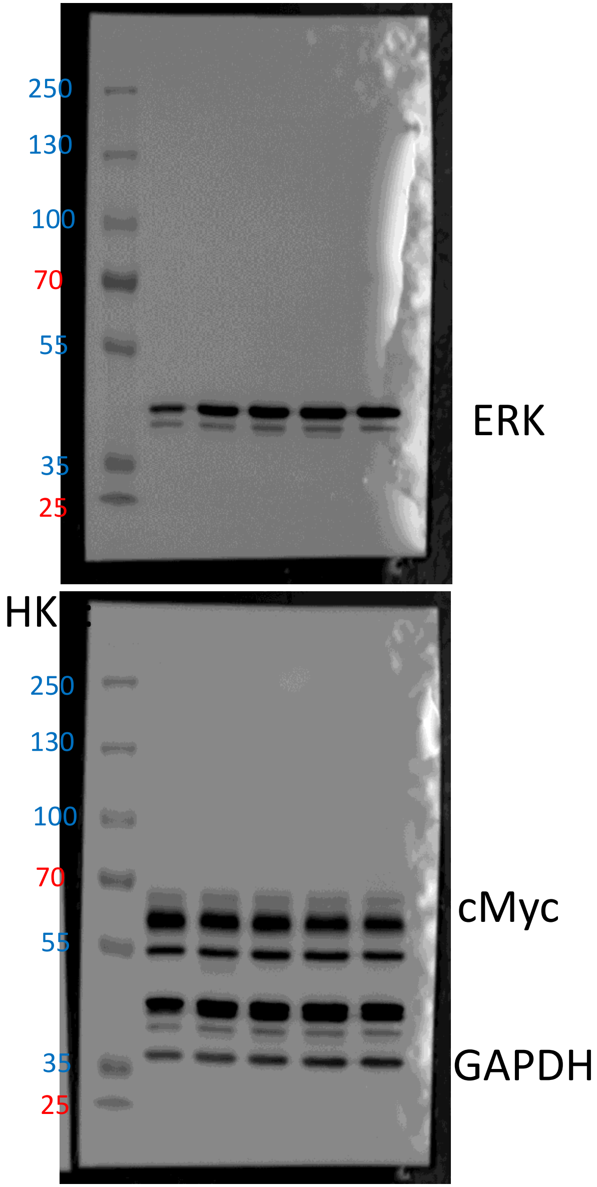

Round 6

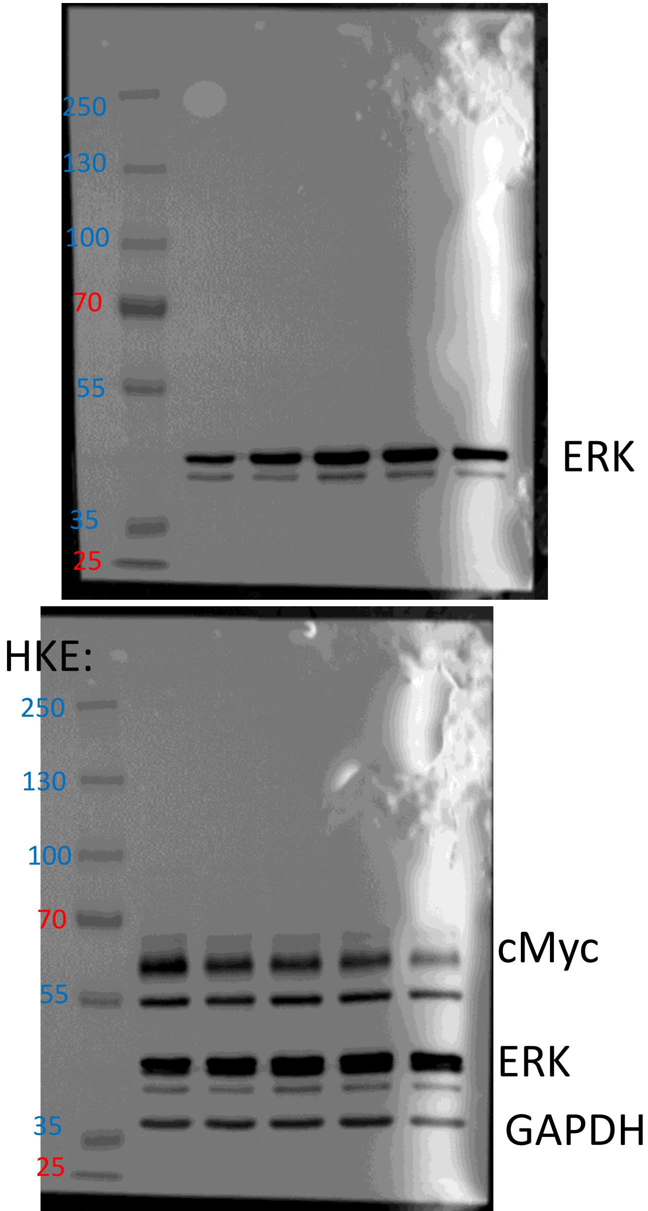

pERK 1/2 (45kDa) // GAPDH (37kDa)

Round 1

Round 2

Round 3

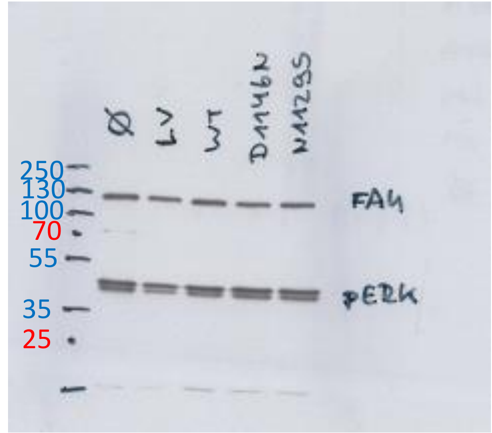

HKE:

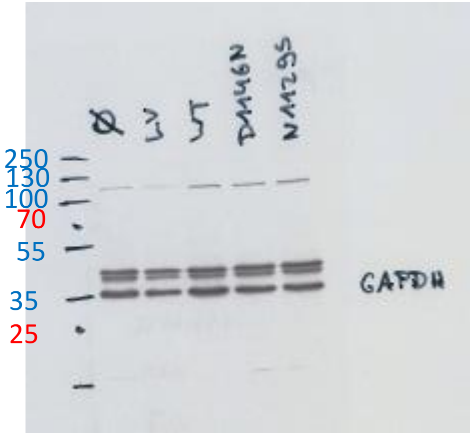

pERK 1/2 (45kDa) // GAPDH (37kDa)

Round 4

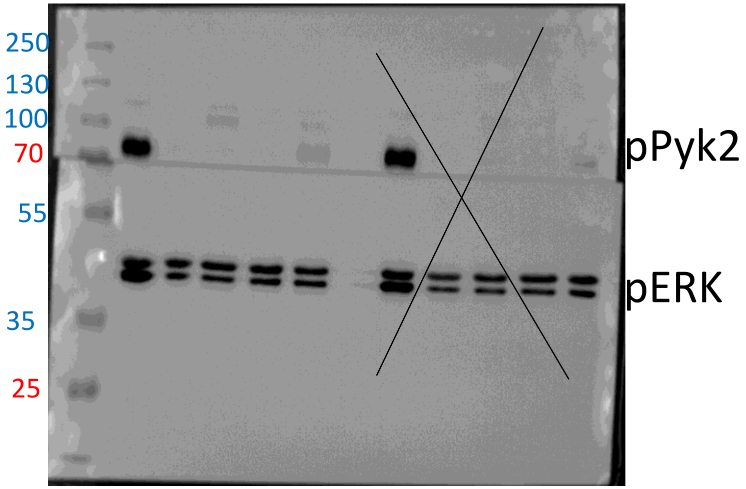

HKE:

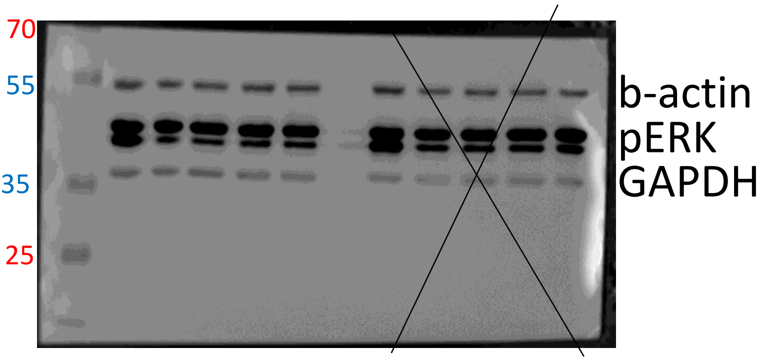

Round 5

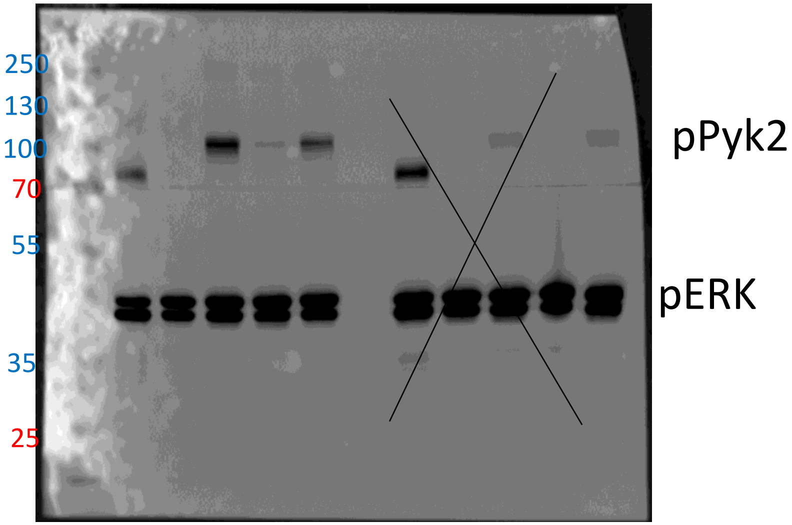

HKE:

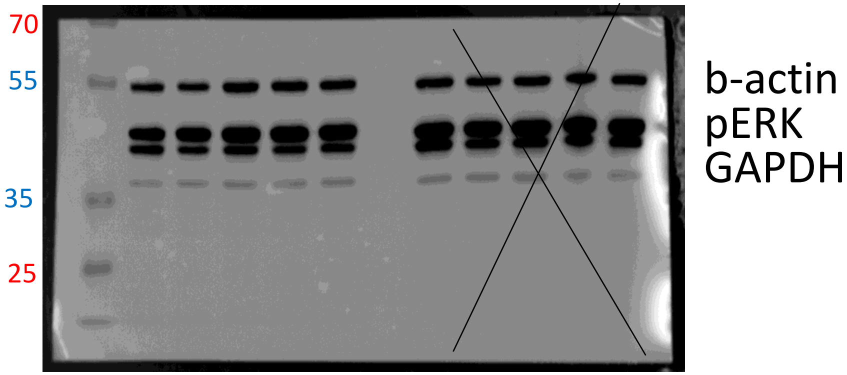

Round 6

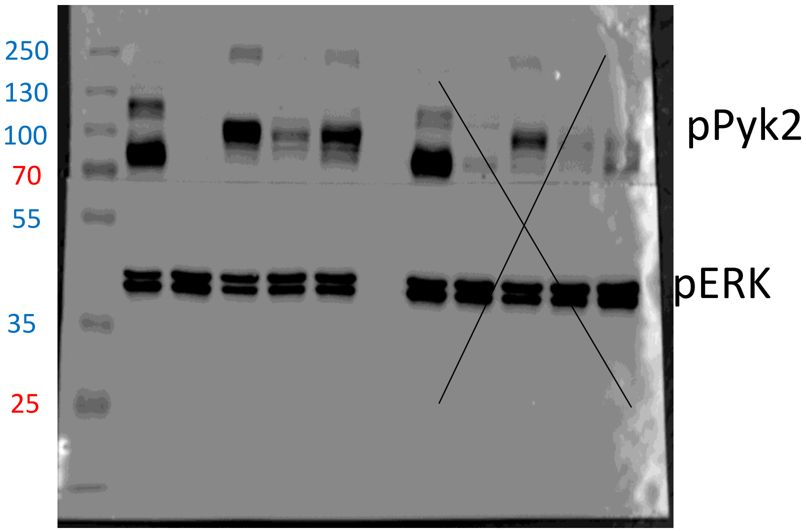

HKE:

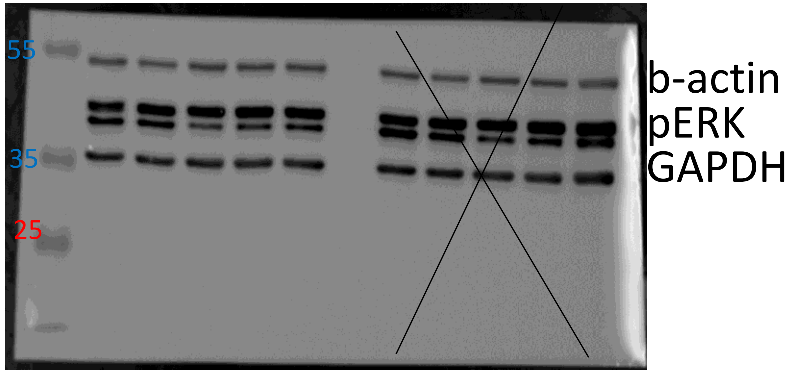

only concerns figure 4

Figure 4

Figure 4 (L-363-B4)

Ladder

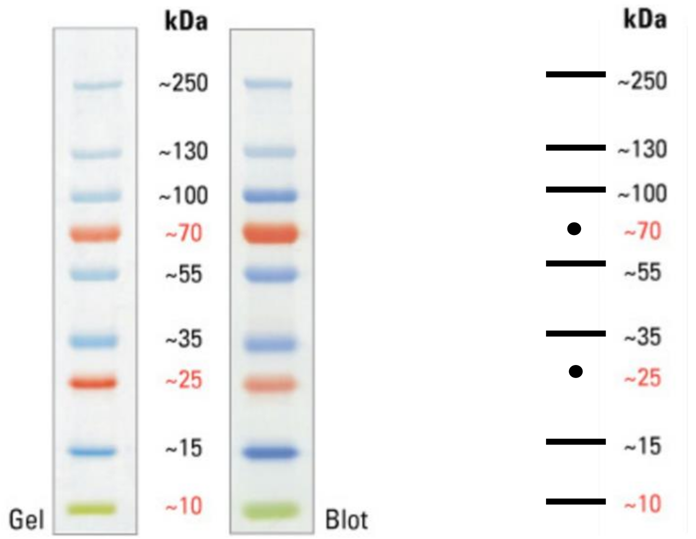

PageRuler™ Plus
Prestained Protein
Ladder, 10 to 250 kDa
Katalognummer: #26
619 Thermo Fischer

Markers:

IGF1R
pIGF1R(Y1135)
pAKT
pMEK1/2
pERK1/2

House keepers (HKEs):
GAPDH
b-actin
b-tubulin

Round depicted
in WB figure

Round not depicted
in WB figure
(used for bar graphs)

Figure 4 (L-363-B4)

Round 1-5:

Loading Ladder | L-363 | L-363-B4 | EV | IGF1R^{WT} | IGF1R^{D1146N} | IGF1R^{N1129S}

	L-363		L-363-B4		EV		IGF1R^{WT}		IGF1R^{D1146N}		IGF1R^{N1129S}	
Loading Ladder	-	+	-	+	-	+	-	+	-	+	-	+

-: serum starved (unstimulated)

+: stimulated with IGF1

only serum starved (-) bands were included in the analysis.

Round 6,7:

Loading: L-363-B4 | EV | IGF1R^{WT} | IGF1R^{D1146N} | IGF1R^{N1129S} — culturing medium, concerns only figure 2

L-363-B4 | EV | IGF1R^{WT} | IGF1R^{D1146N} | IGF1R^{N1129S} — starving medium

IGF1R (95kDa) // GAPDH (37kDa)

Round 1

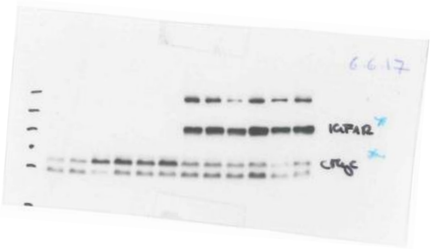

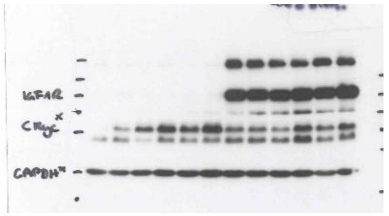

Round 2

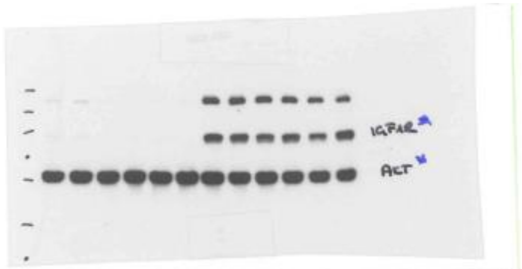

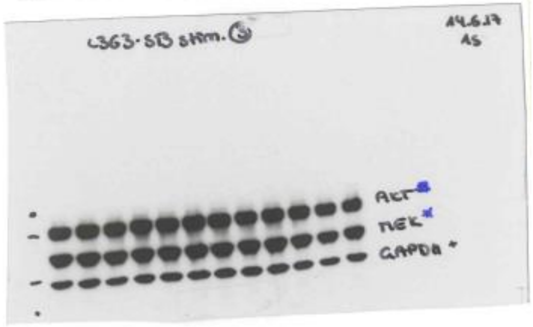

Round 3

Round 4

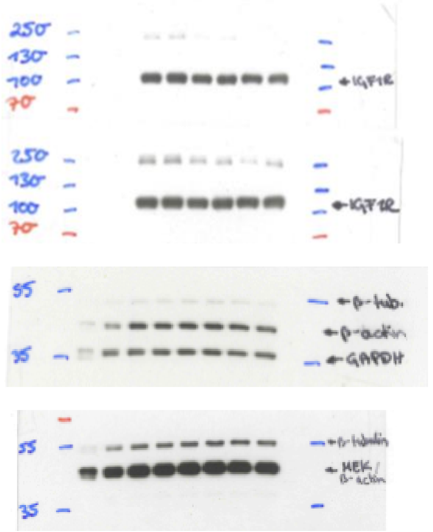

Round 5

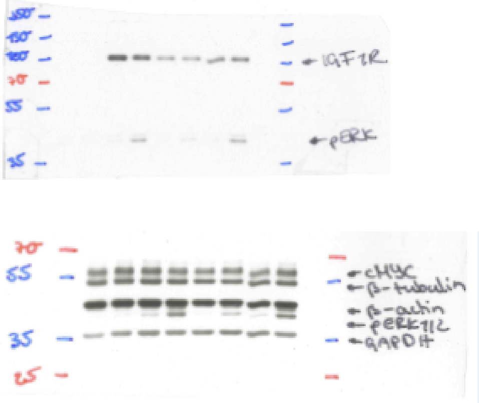

IGF1R (95kDa) // GAPDH (37kDa)

Round 6

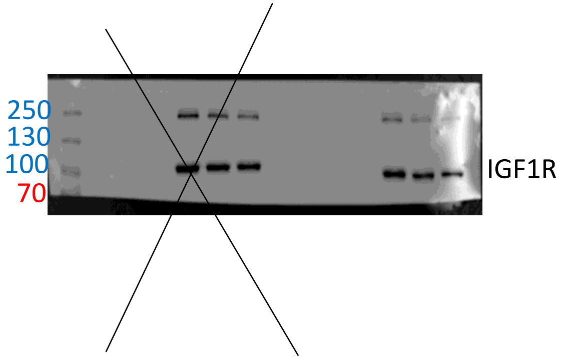

HKE:

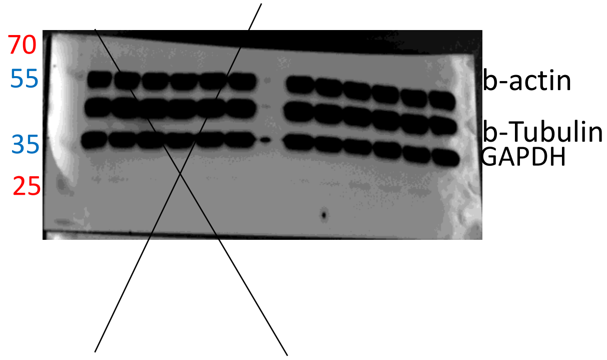

Round 7

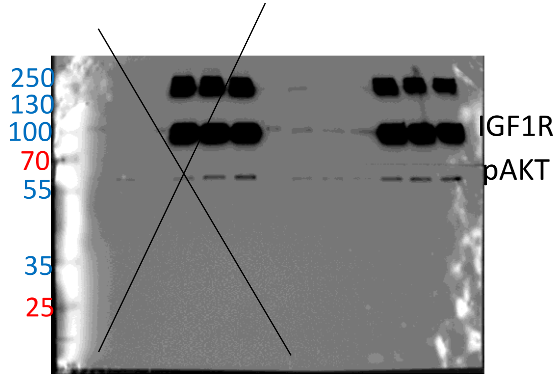

HKE:

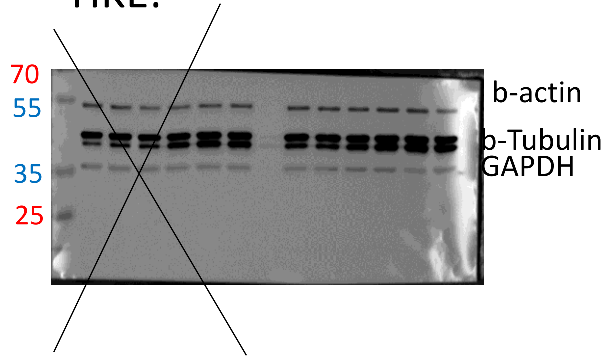

X only concerns figure 2

pIGF1R (Y1135) (95kDa) // GAPDH (37kDa)

Round 1

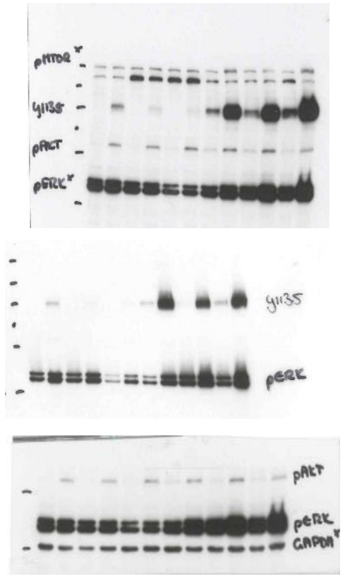

Round 2

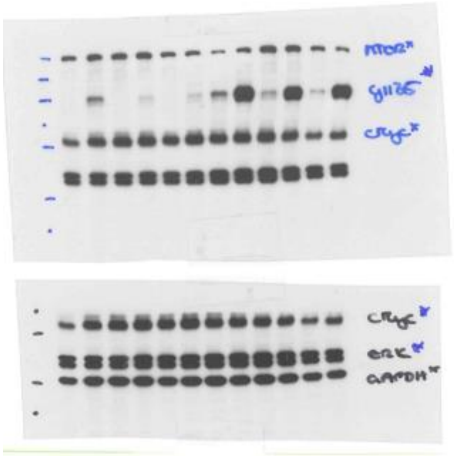

Round 3

Round 4

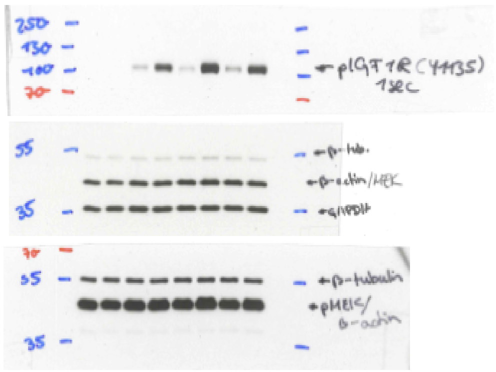

Round 5

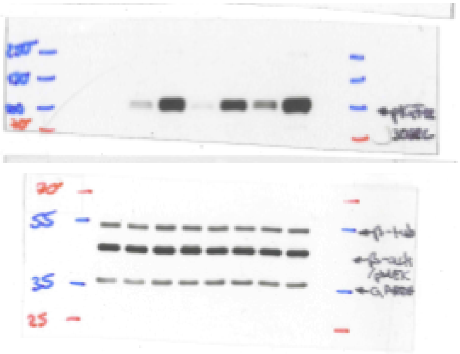

pIGF1R (Y1135) (95kDa) // GAPDH (37kDa)

Round 6

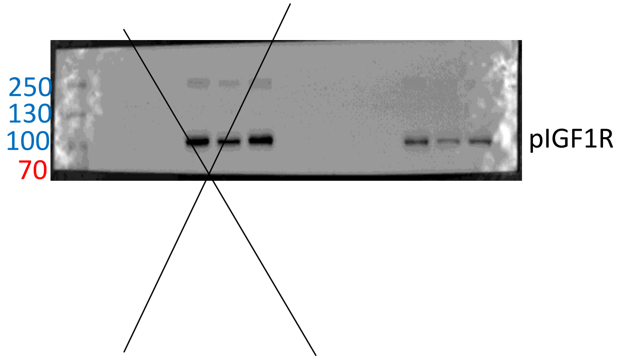

HKE:

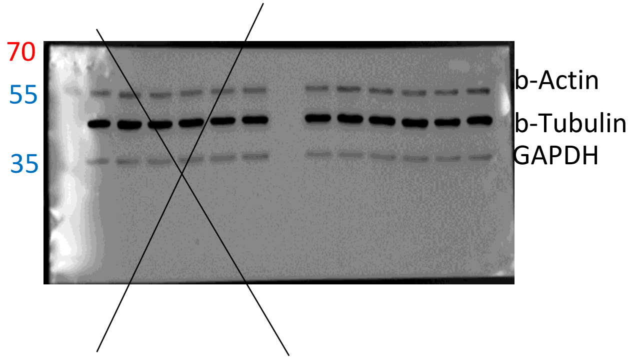

Round 7

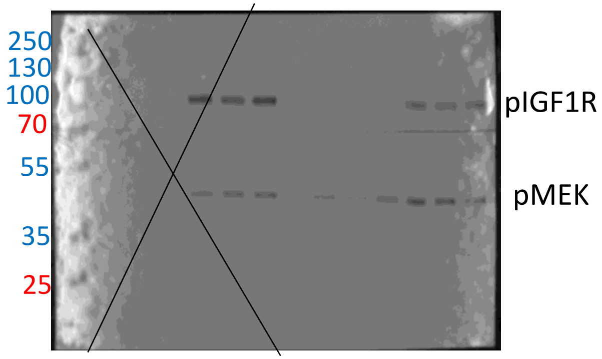

HKE:

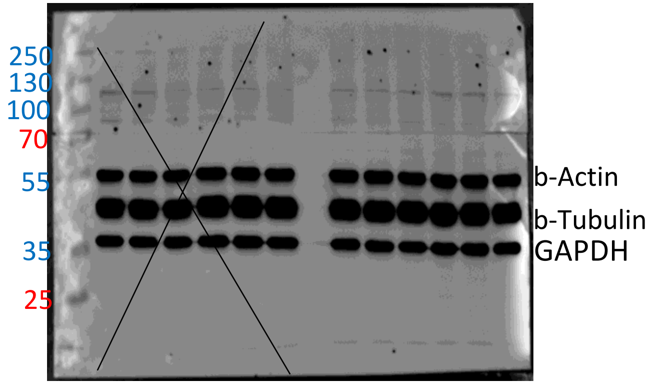

only concerns figure 2

pAKT (60kDa) // GAPDH (37kDa)

Round 1

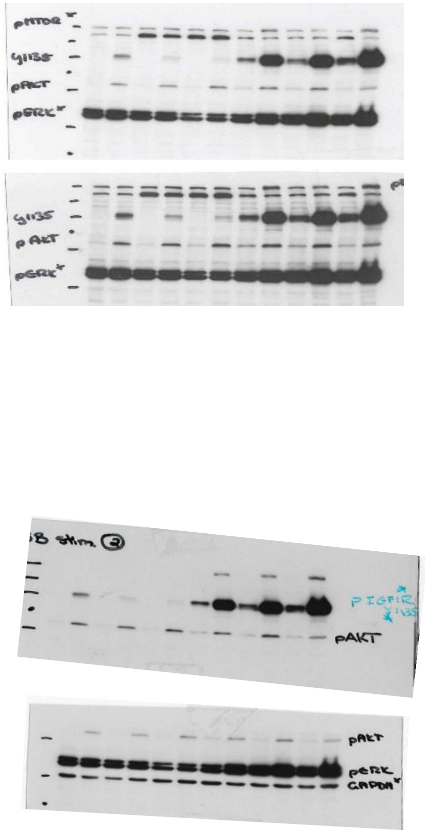

Round 2

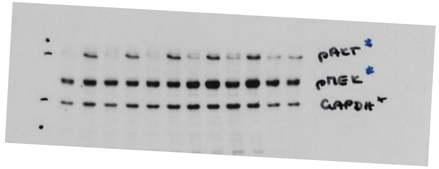

Round 3

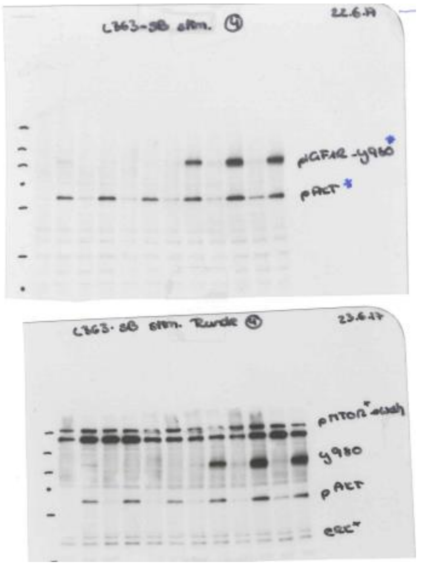

Round 4

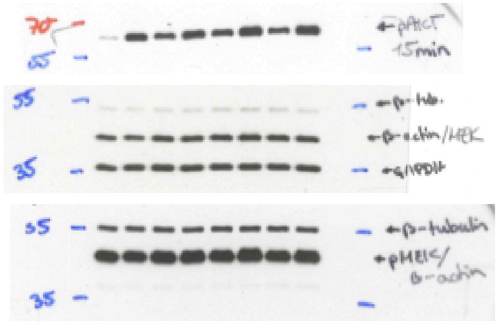

Round 5

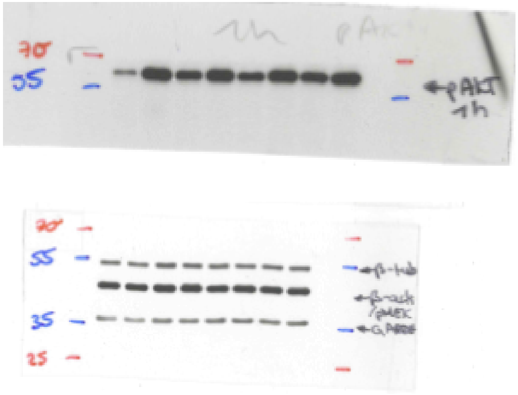

pAKT (60kDa) // GAPDH (37kDa)

Round 6

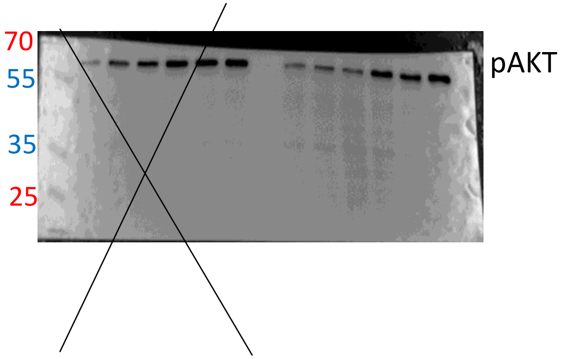

HKE:

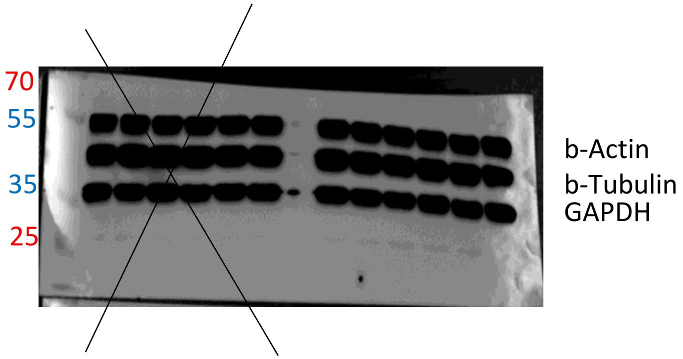

Round 7

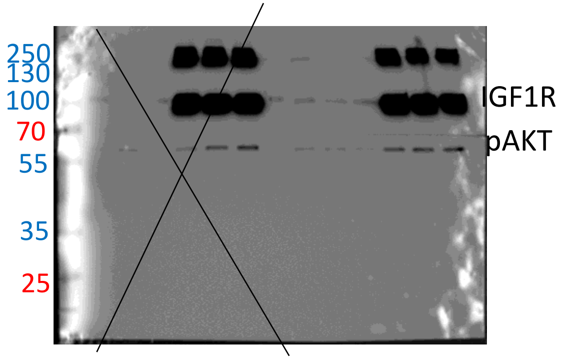

HKE:

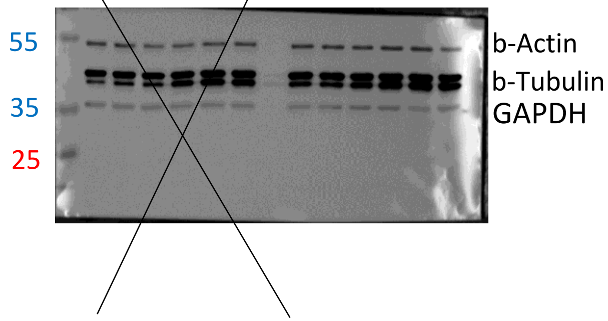

X only concerns figure 2

pMEK 1/2 (45kDa) // GAPDH (37kDa)

Round 1

Round 2

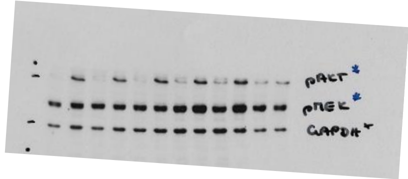

Round 3

Round 4

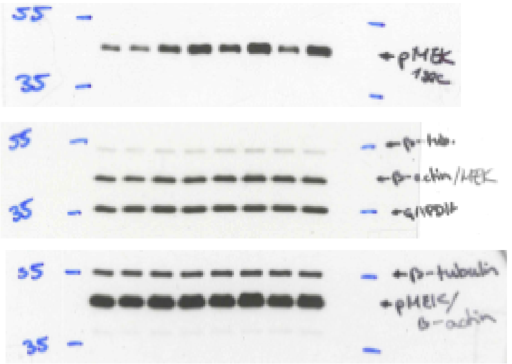

Round 5

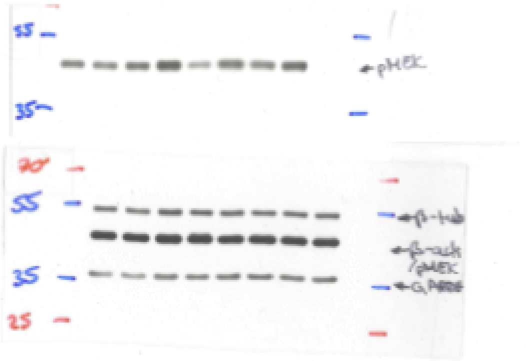

pMEK 1/2 (45kDa) // GAPDH (37kDa)

Round 6

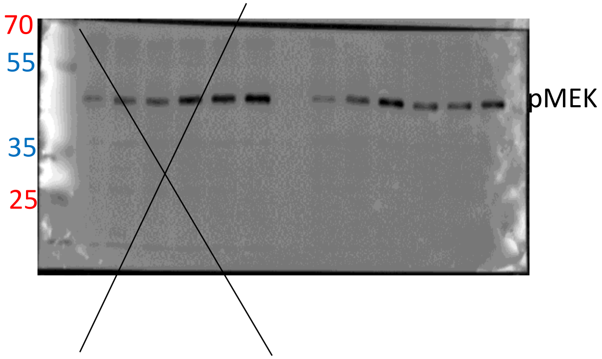

HKE:

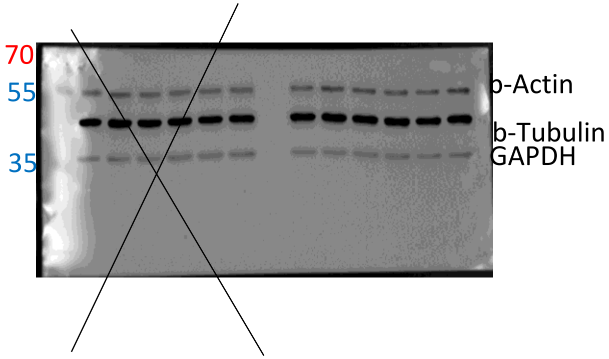

Round 7

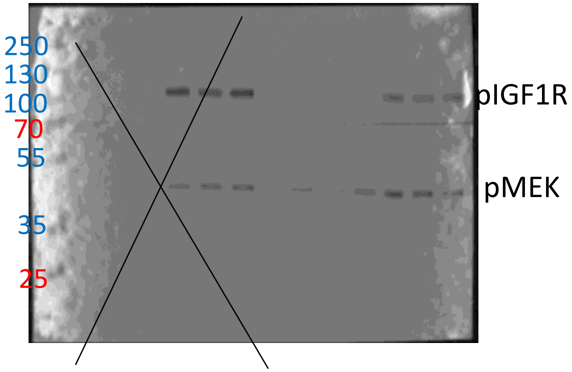

HKE:

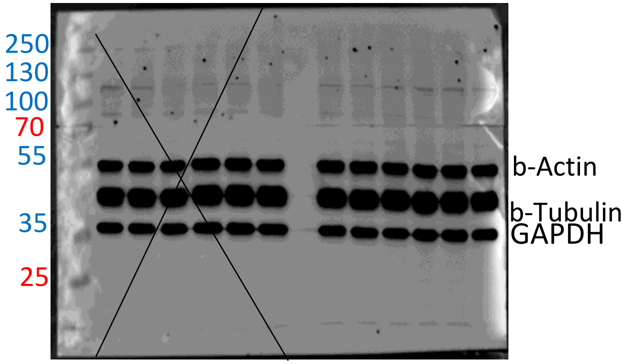

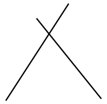
only concerns figure 2

pERK 1/2 (45kDa) // GAPDH (37kDa)

Round 1

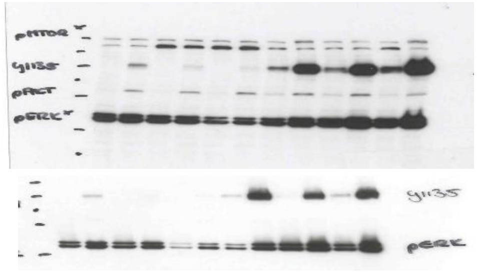

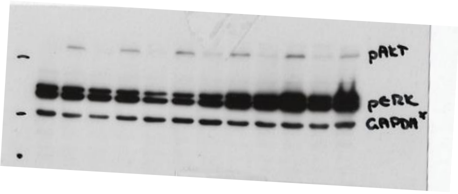

Round 2

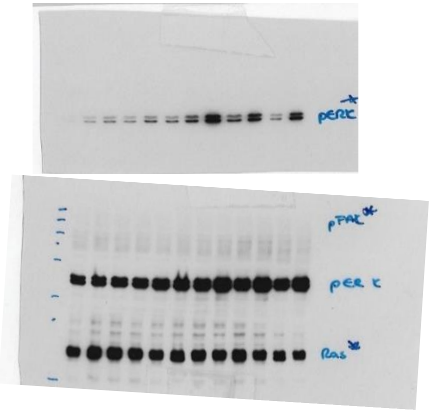

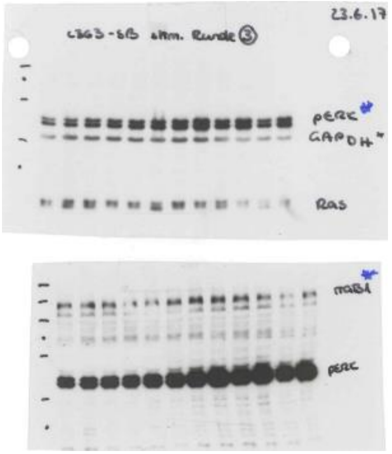

Round 3

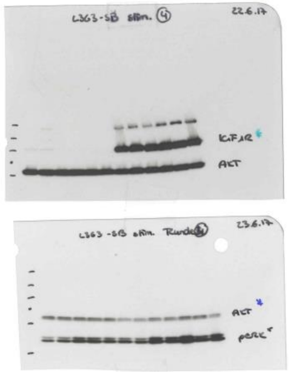

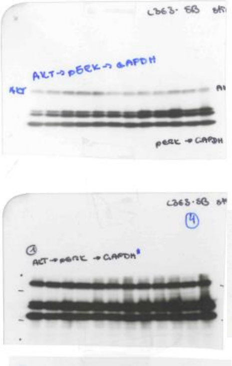

Round 4

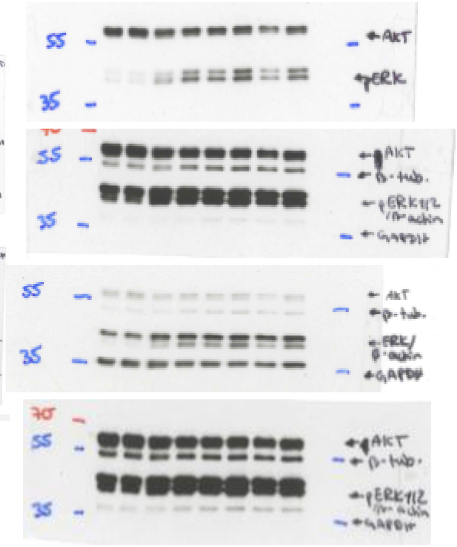

Round 5

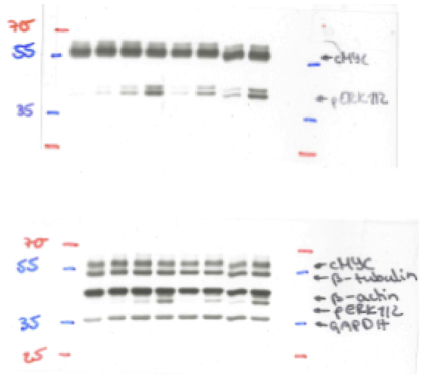

pERK 1/2 (45kDa) // GAPDH (37kDa)

Round 6

Round 7

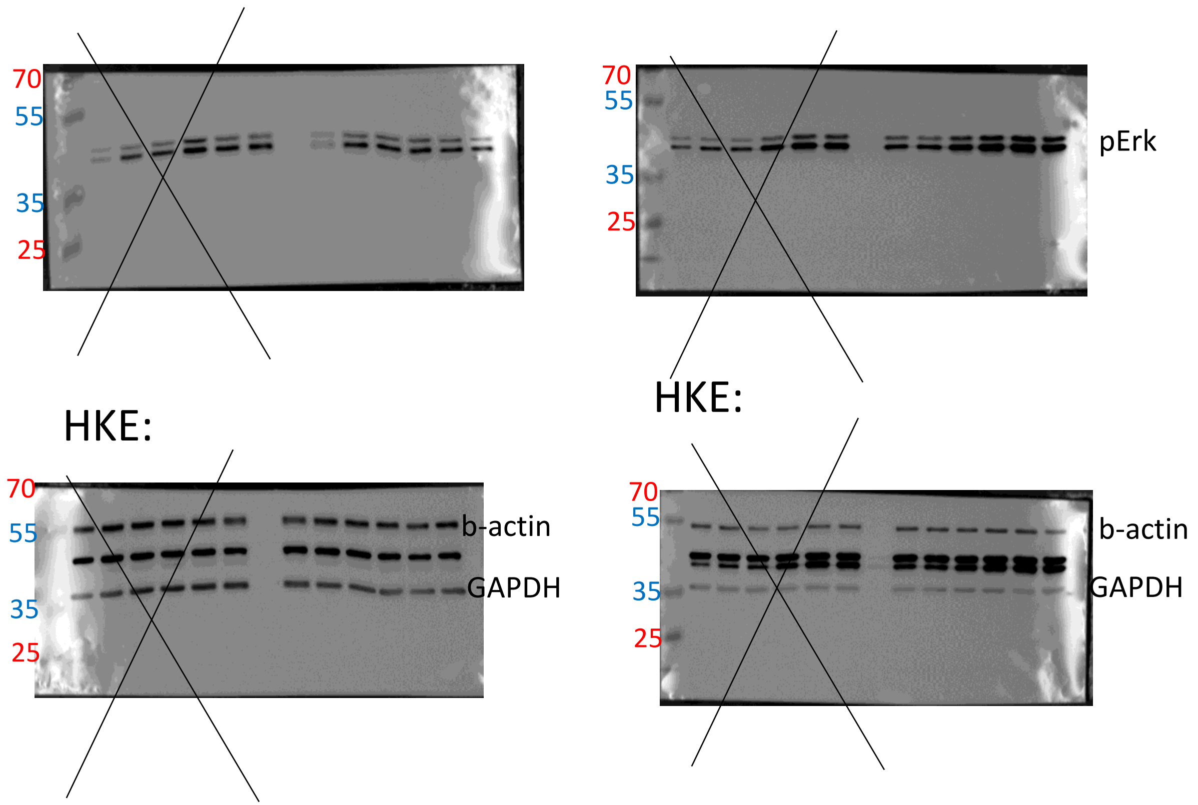

only concerns
figure 2

Figure 4 (AMO-1)

Round 1,2,4-8:

Loading Ladder	AMO-1	EV	IGF1R^{WT}	IGF1R^{D1146N}	IGF1R^{N1129S}
	- +	- +	- +	- +	- +

Round 3:

Loading Ladder	AMO-1	EV	IGF1R^{WT}	IGF1R^{D1146N}	IGF1R^{N1129S}
	+ -	+ -	+ -	+ -	+ -

-: serum starved (unstimulated), only these bands were used in the analysis

+: stimulated with IGF1

Round 9,10:

Loading: ~~AMO-1 EV IGF1R^{WT} IGF1R^{D1146N} IGF1R^{N1129S}~~ AMO-1 EV IGF1R^{WT} IGF1R^{D1146N} IGF1R^{N1129S}

~~culturing medium, only concerns Figure 2~~ | starving medium

IGF1R (95kDa) // GAPDH (37kDa)

Round 1

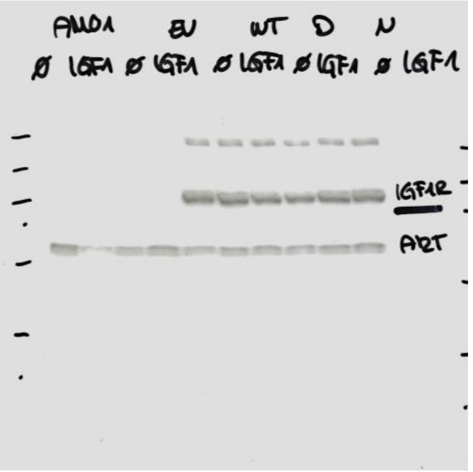

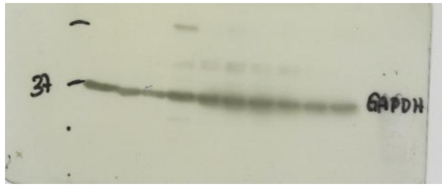

Round 2

Round 3

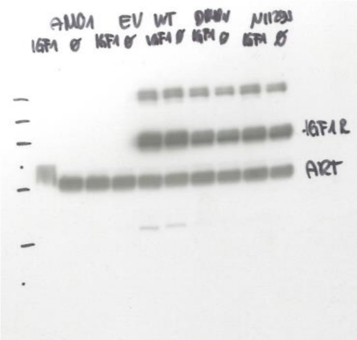

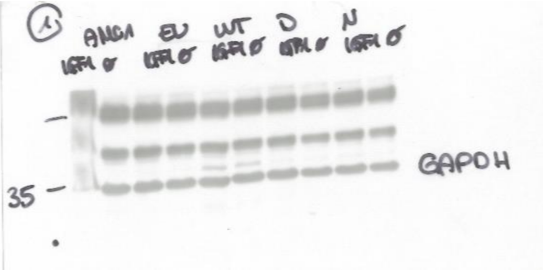

Round 4

IGF1R (95kDa) // GAPDH (37kDa)

Round 5

Round 6

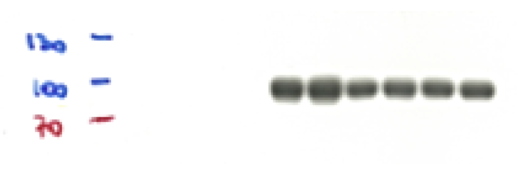

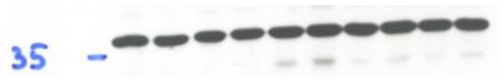

Round 7

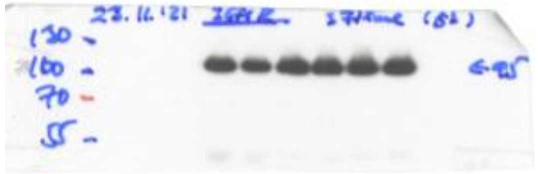

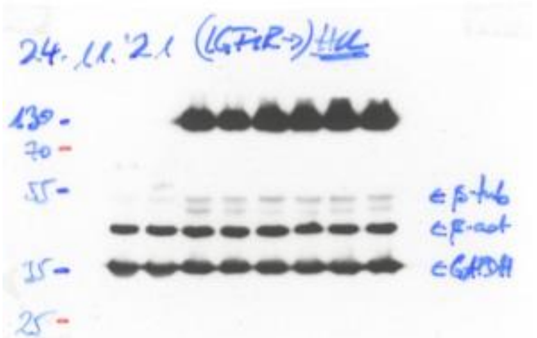

Round 8

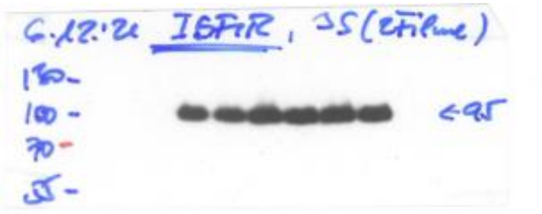

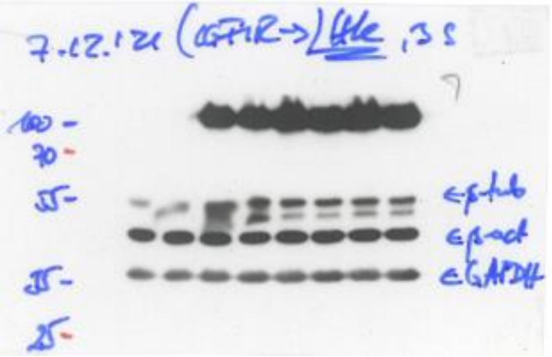

IGF1R (95kDa) // GAPDH (37kDa)

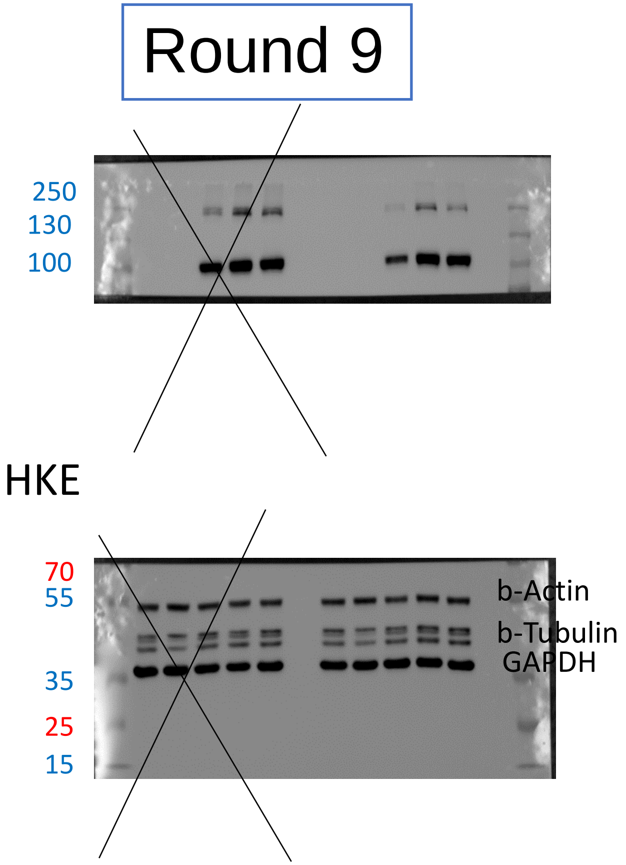

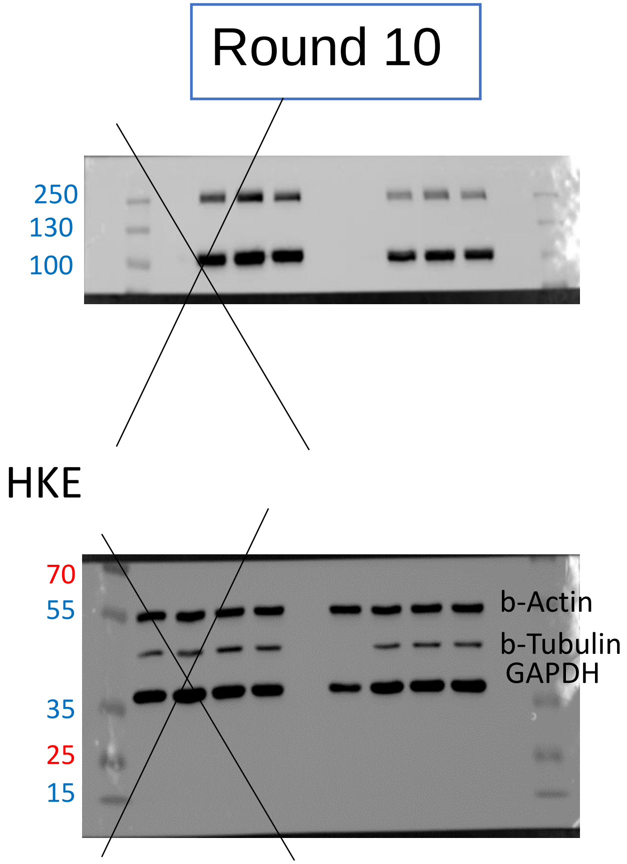

X only concerns figure 2

pIGF1R (Y1135) (95kDa) // GAPDH (37kDa)

Round 1

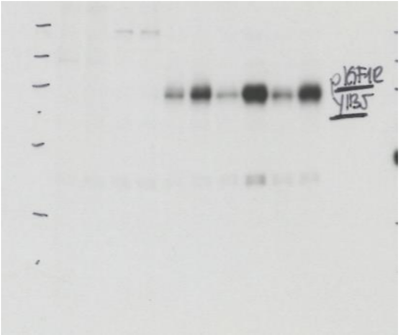

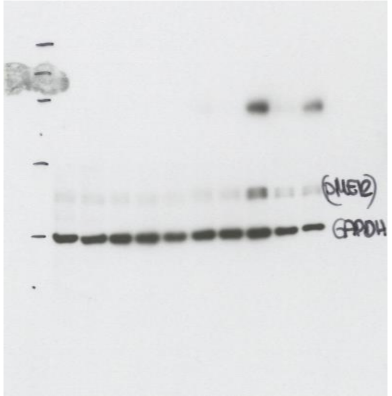

Round 2

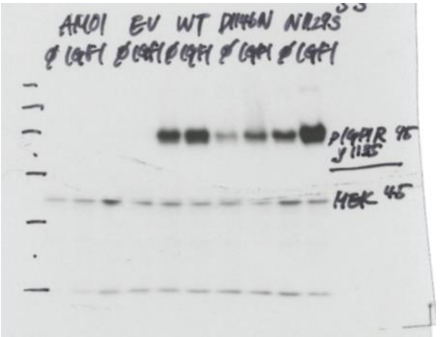

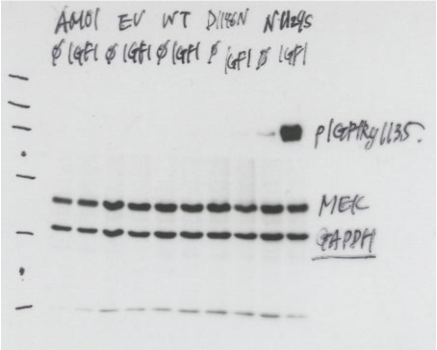

Round 3

Round 4

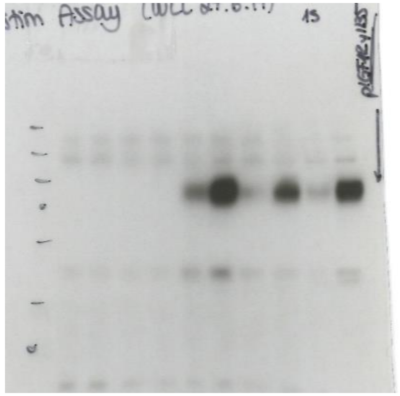

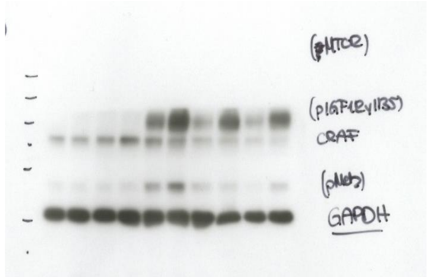

pIGF1R (Y1135) (95kDa) // GAPDH (37kDa)

Round 5

Round 6

Round 7

Round 8

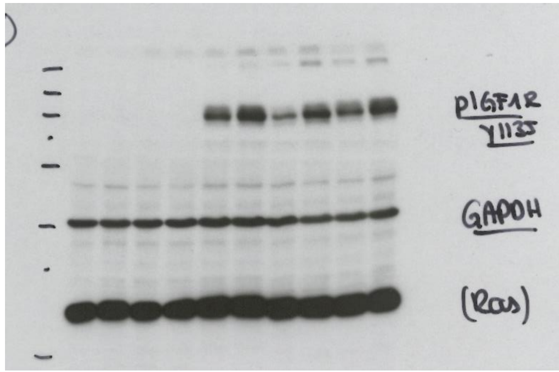

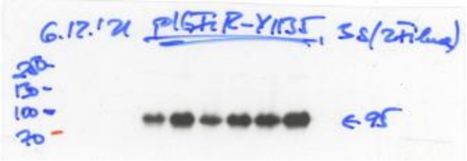

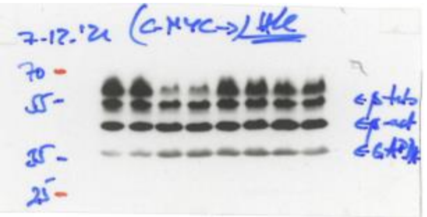

pIGF1R (Y1135) (95kDa) // GAPDH (37kDa)

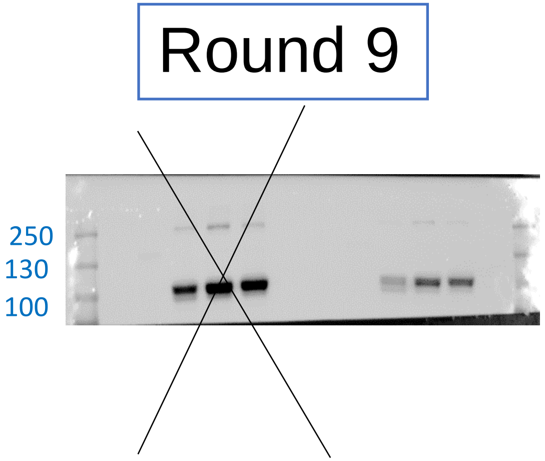

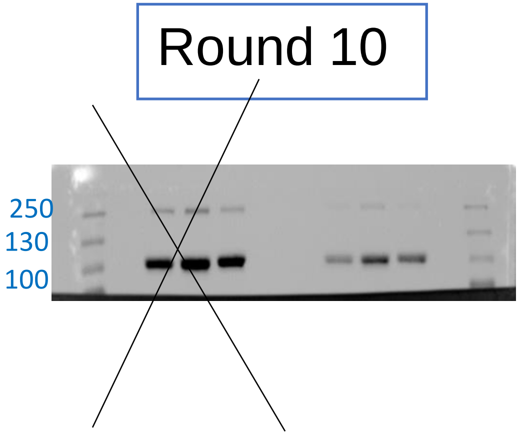

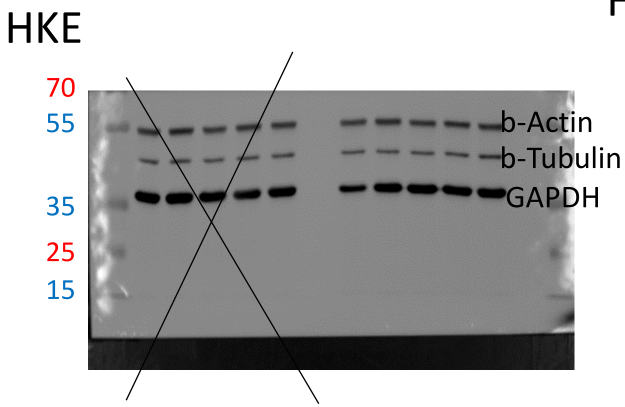

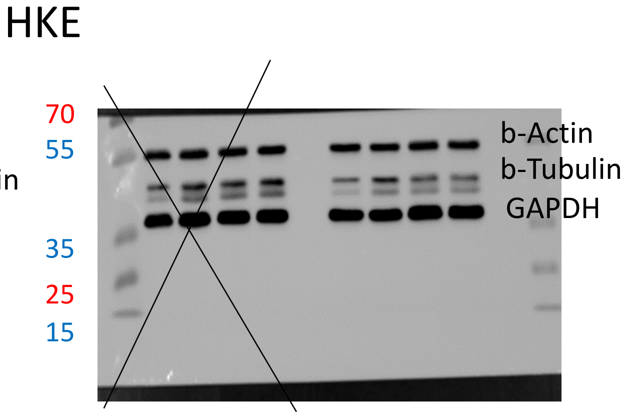

only concerns figure 2

pAKT (60kDa) // GAPDH (37kDa)

Round 1

Round 2

Round 3

Round 4

Round 5

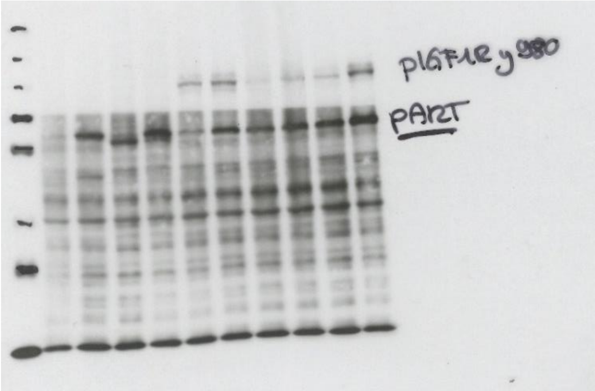

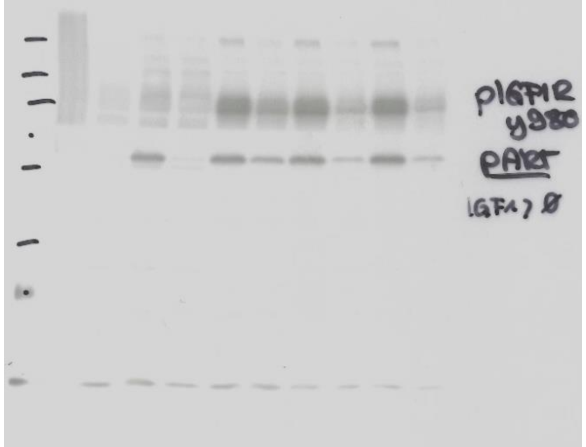

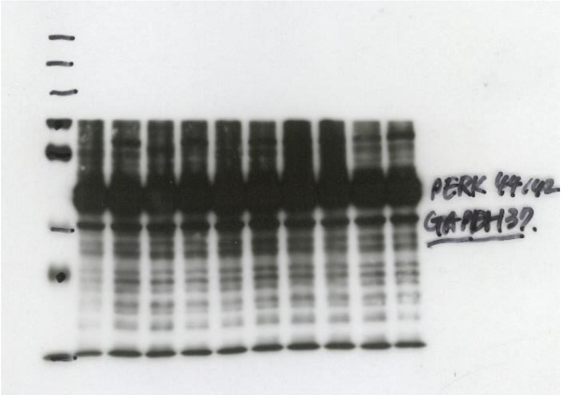

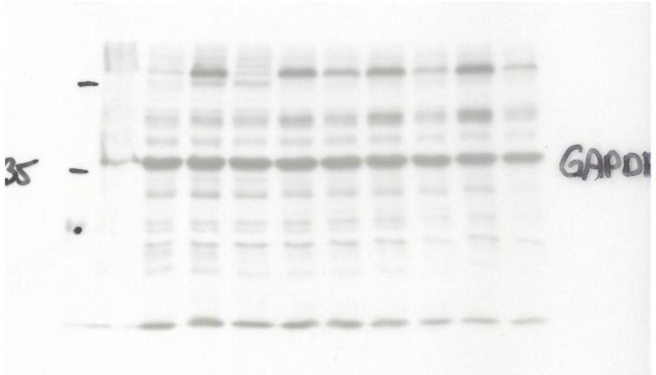

pAKT (60kDa) // GAPDH (37kDa)

Round 6

Round 7

Round 8

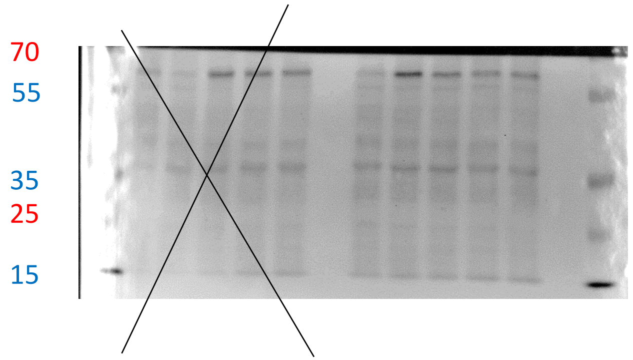

HKE:

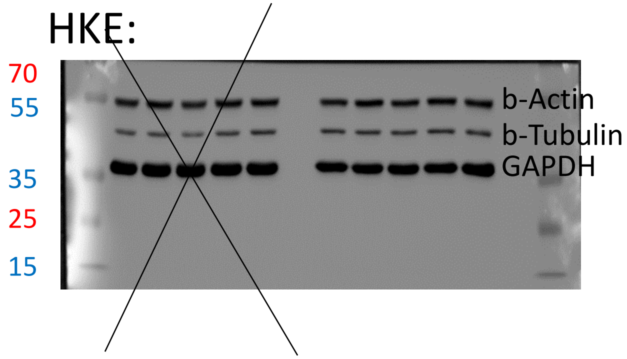

Round 9

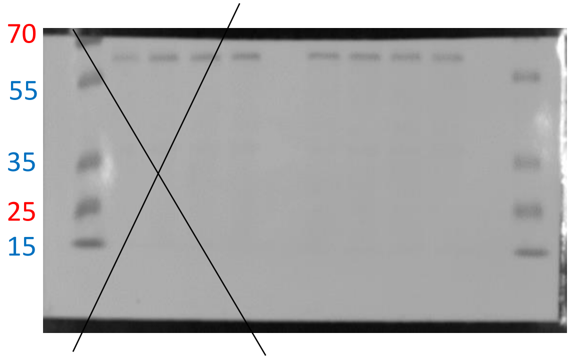

HKE:

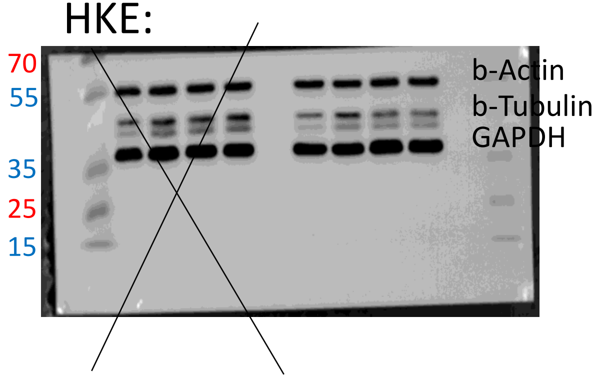

only concerns figure 2

pMEK 1/2 (45kDa) // GAPDH (37kDa)

Round 1

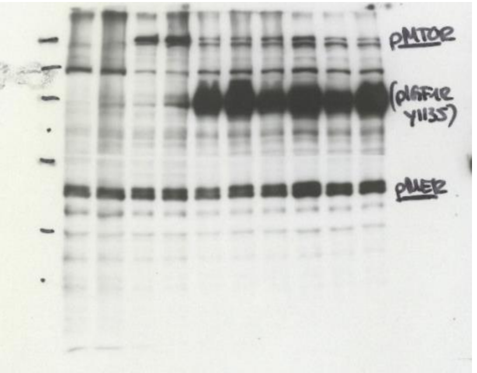

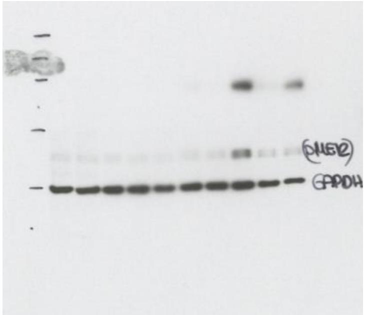

Round 2

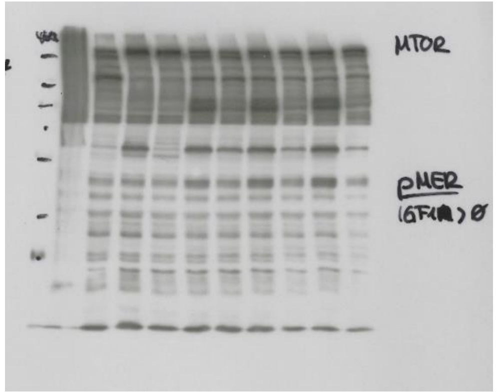

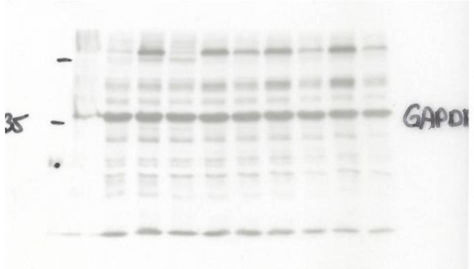

Round 3

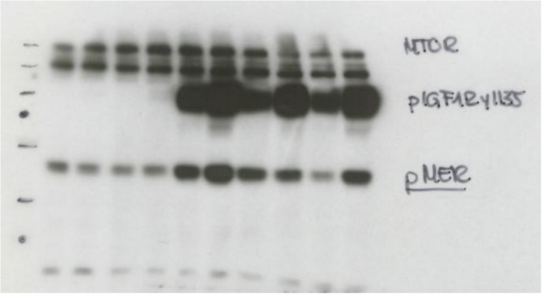

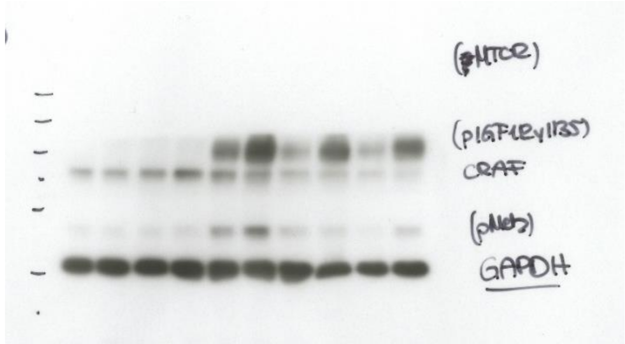

Round 4

Round 5

pMEK 1/2 (45kDa) // GAPDH (37kDa)

Round 6

Round 7

Round 8

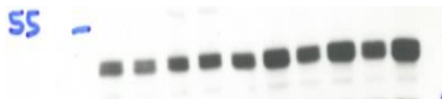

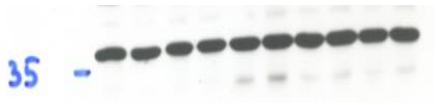

Round 9

Round 10

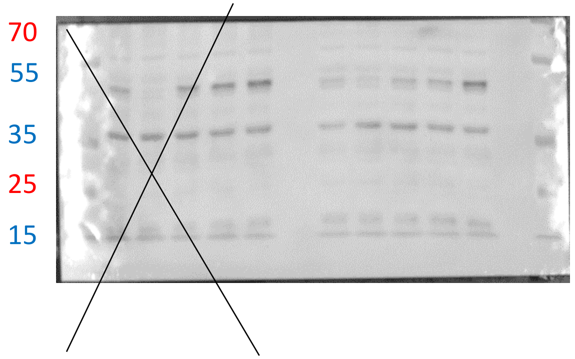

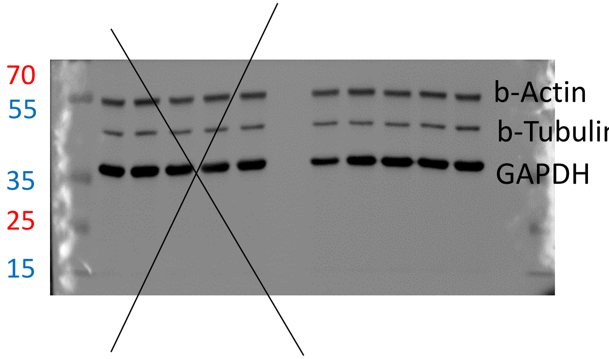

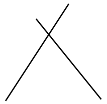

only concerns figure 2

pERK 1/2 (45kDa) // GAPDH (37kDa)

Round 1

Round 2

Round 3

Round 4

Round 5

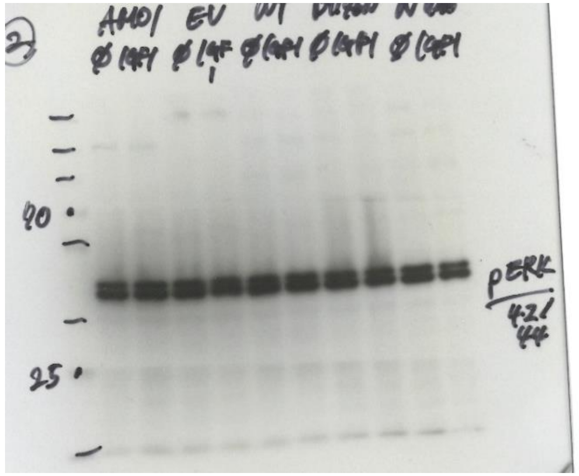

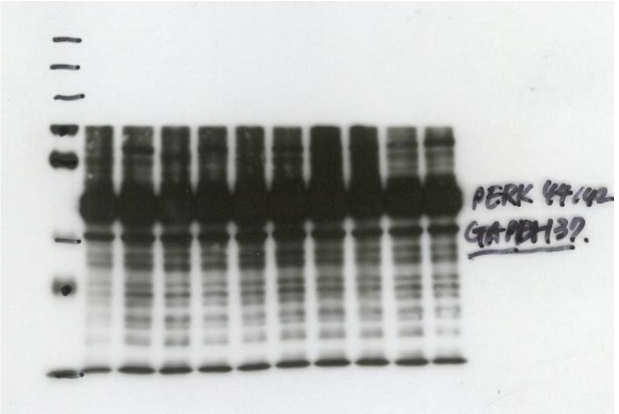

pERK 1/2 (45kDa) // GAPDH (37kDa)

Round 6

Round 7

Round 8

Round 9

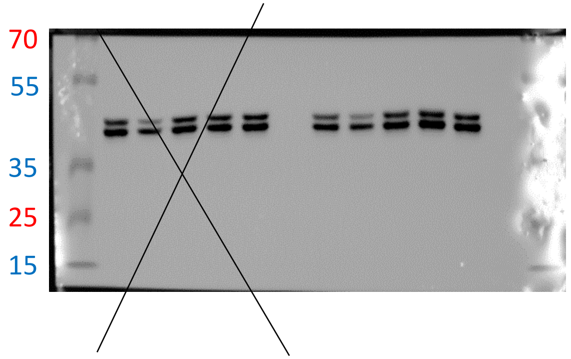

HKE:

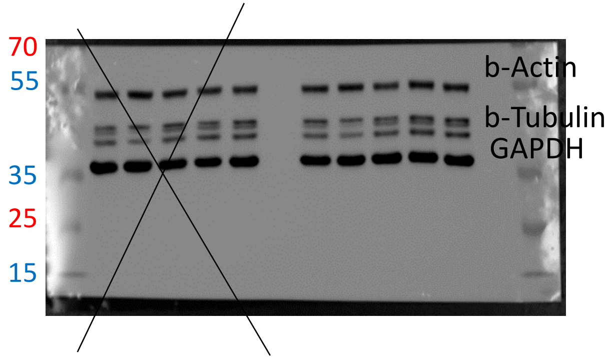

Round 10

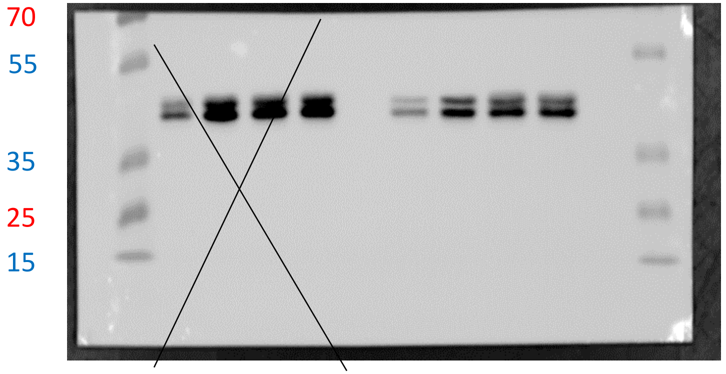

HKE:

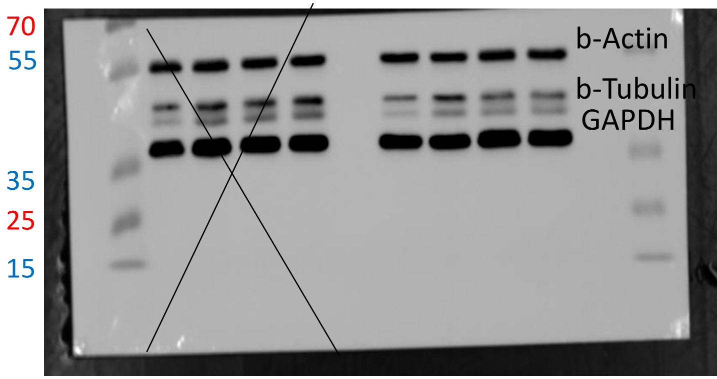

only concerns figure 2

Figure 4 (AMO-1) uncropped blots of representative blot: Protein from round 2 loaded in correct order

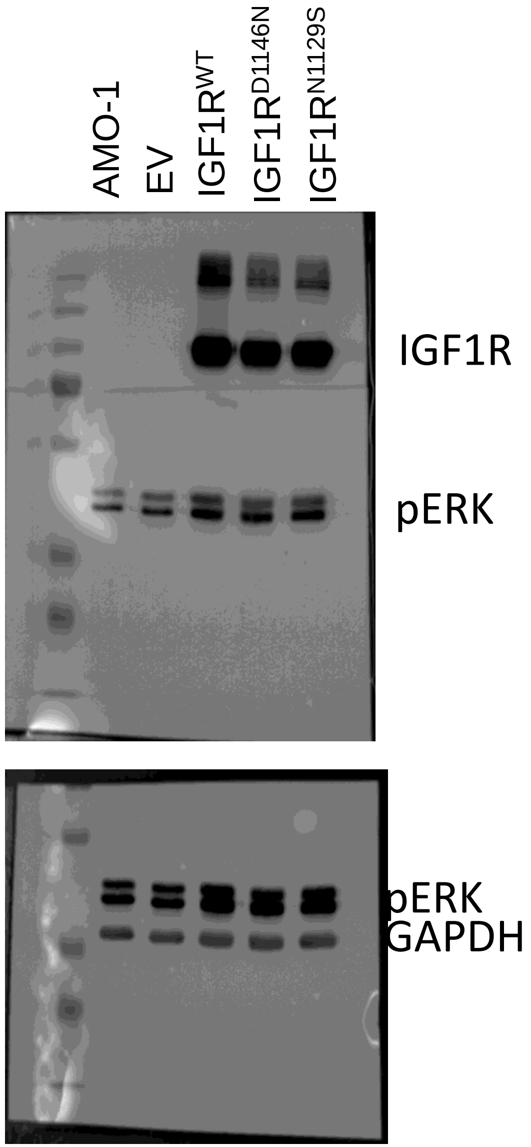

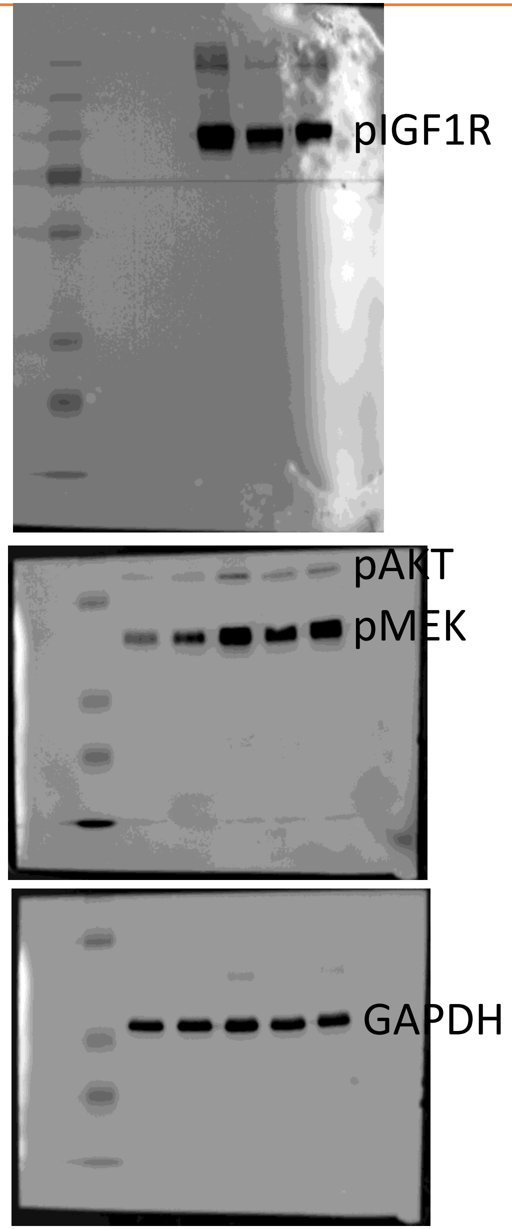

Figure 4 (U-266)

Round 1-4:

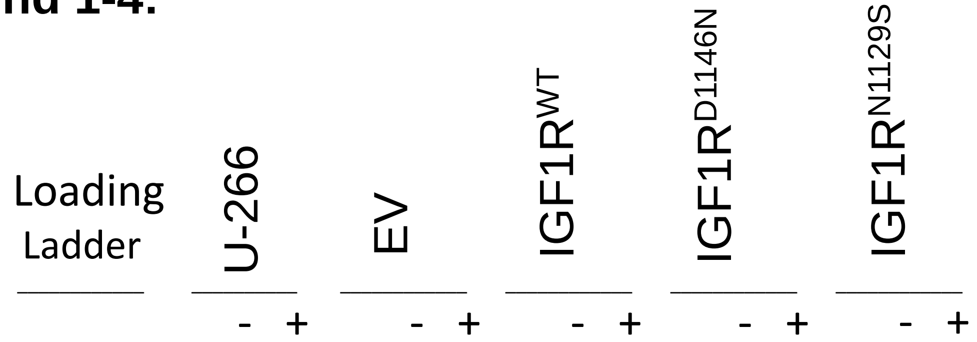

-: serum starved (unstimulated), only these bands used for analysis

+: stimulated with IGF1

Round 5,6:

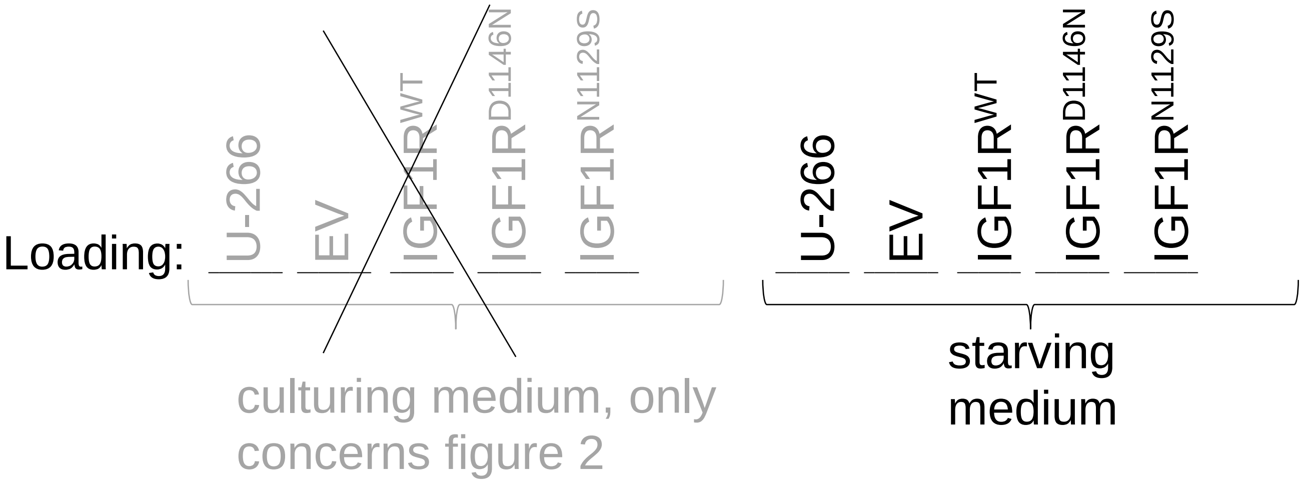

IGF1R (95kDa) // GAPDH (37kDa)

Round 1

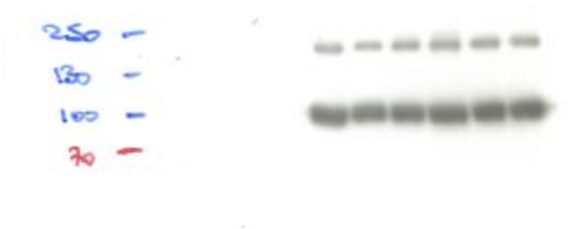

HKE

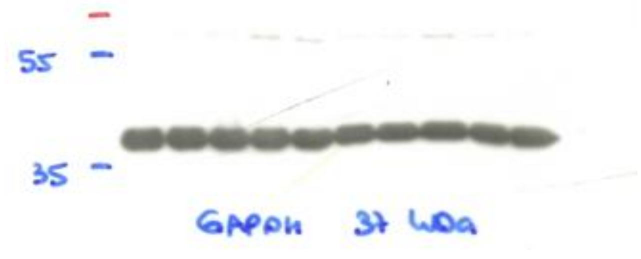

Round 2

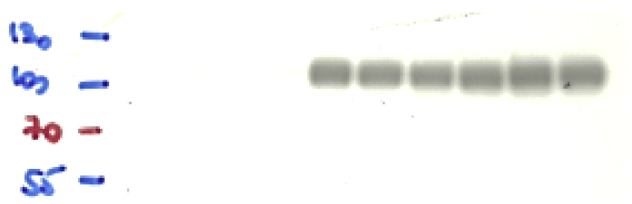

HKE

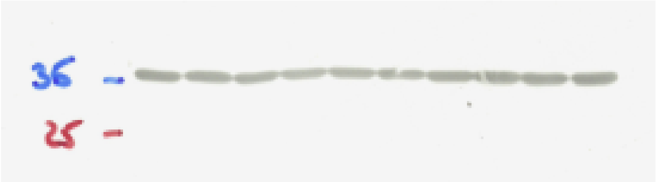

Round 3

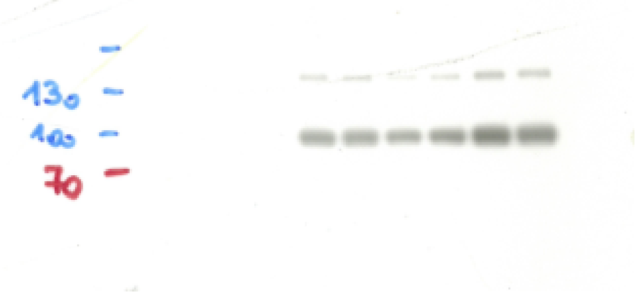

HKE

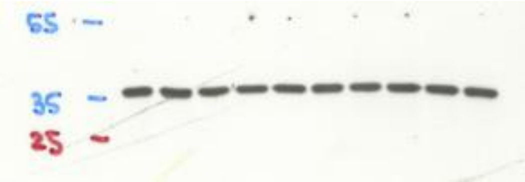

IGF1R (95kDa) // GAPDH (37kDa)

Round 4

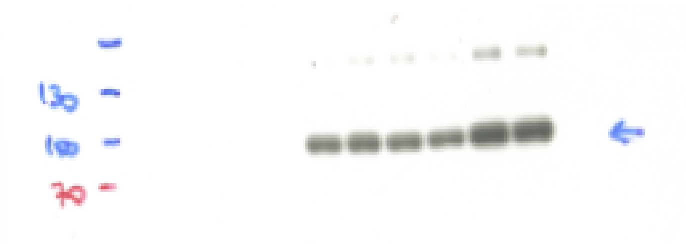

HKE

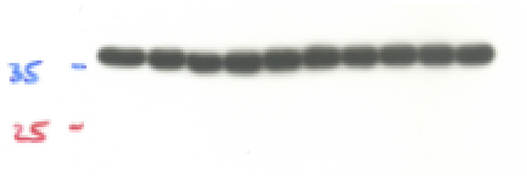

Round 5

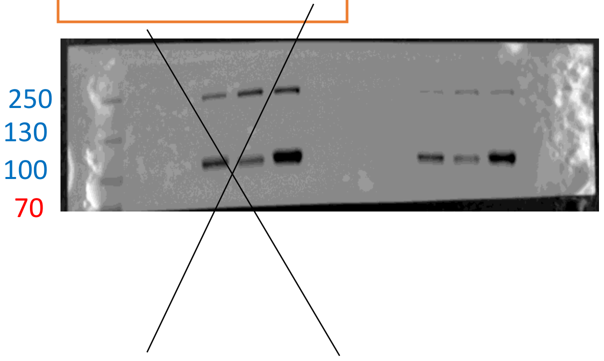

HKE

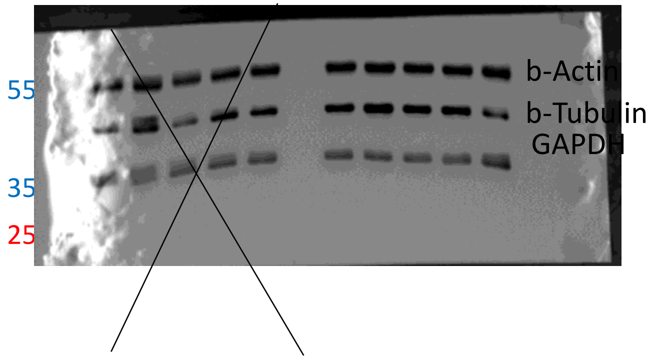

Round 6

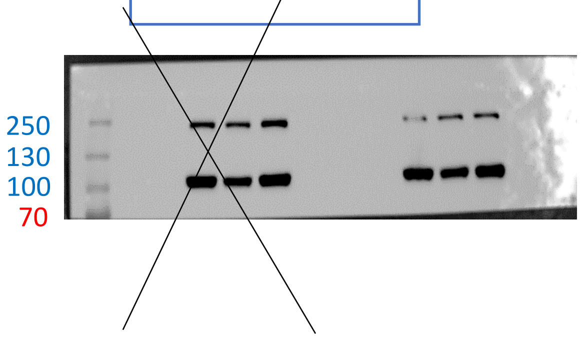

HKE

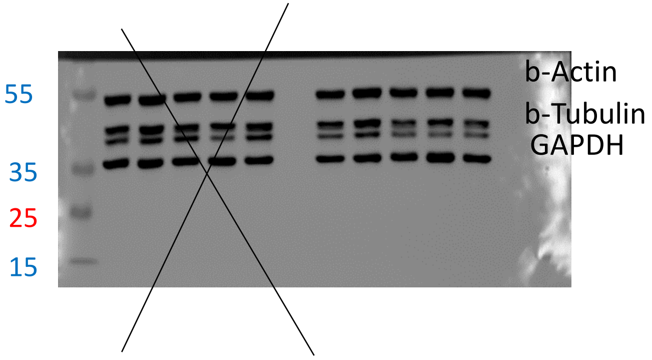

X only concerns figure 2

pIGF1R (Y1135) (95kDa) // GAPDH (37kDa)

Round 1

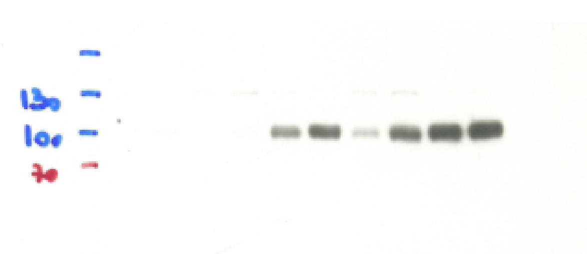

HKE

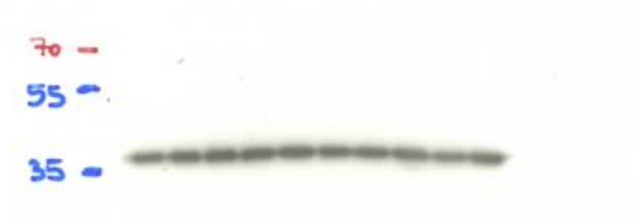

Round 2

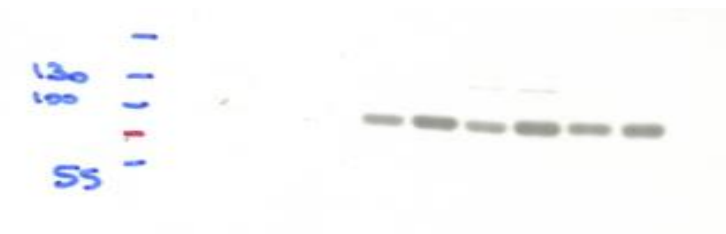

HKE

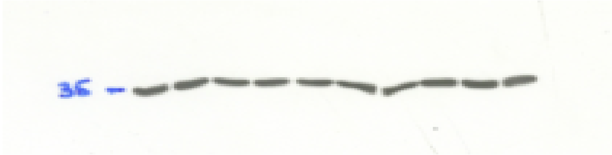

Round 3

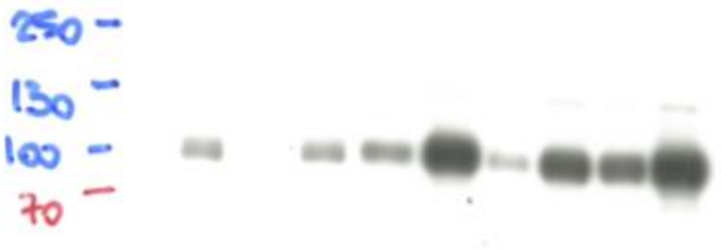

HKE

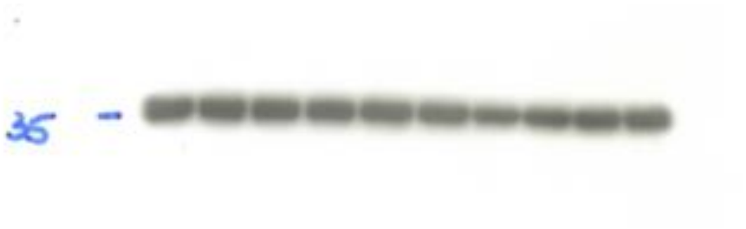

pIGF1R (Y1135) (95kDa) // GAPDH (37kDa)

Round 4

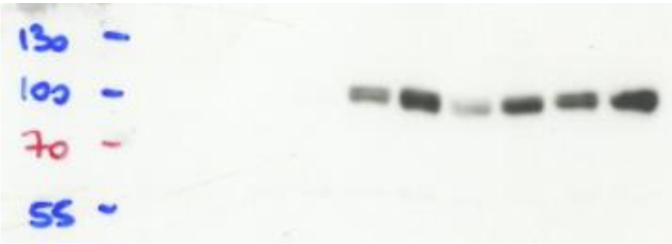

HKE

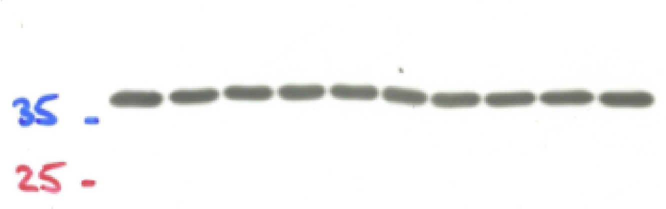

Round 5

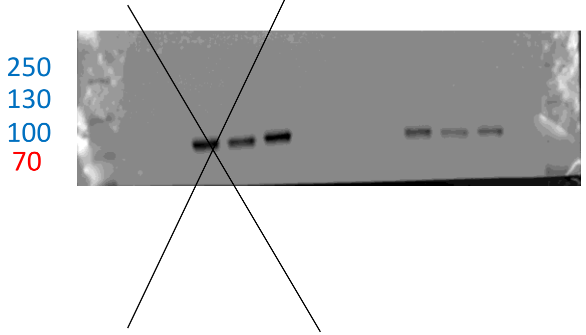

HKE

HKE:

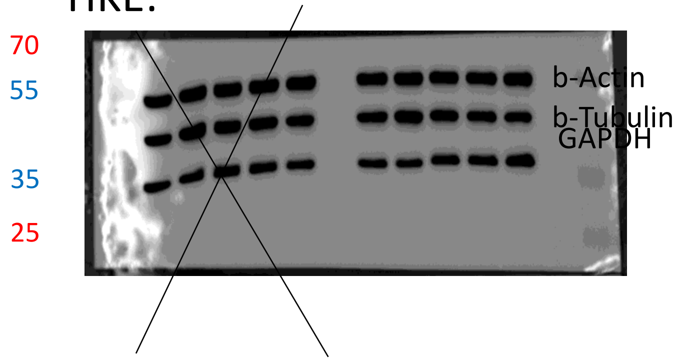

Round 6

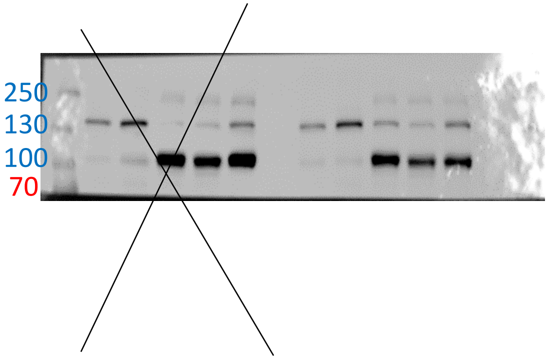

HKE

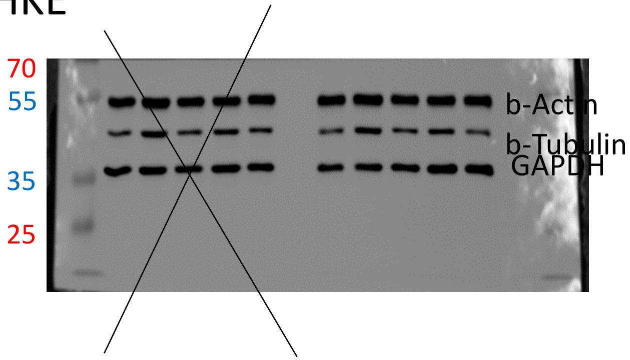

X only concerns figure 2

pAKT (60kDa) // GAPDH (37kDa)

Round 1

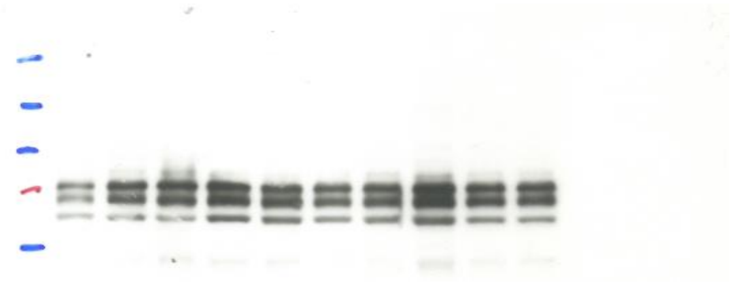

HKE

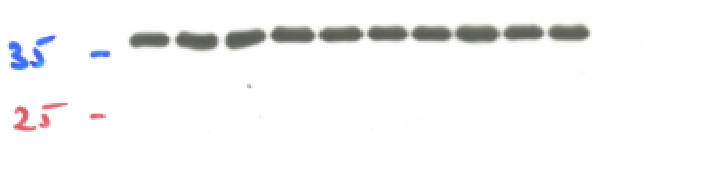

Round 2

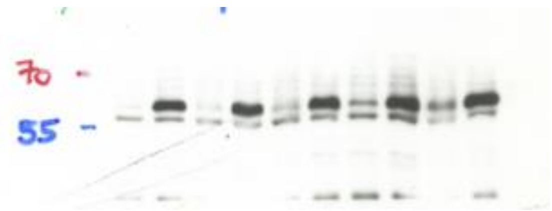

HKE

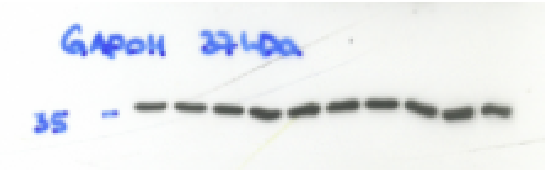

Round 3

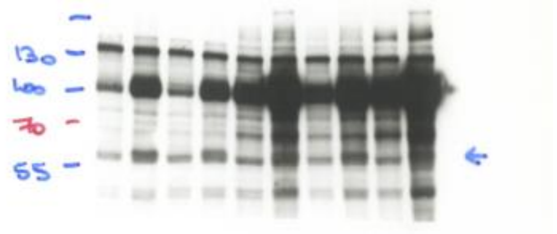

HKE

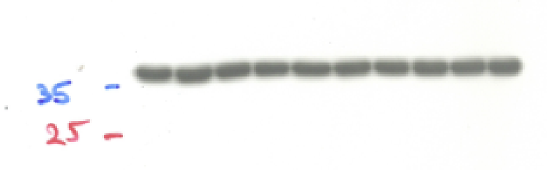

pAKT (60kDa) // GAPDH (37kDa)

Round4

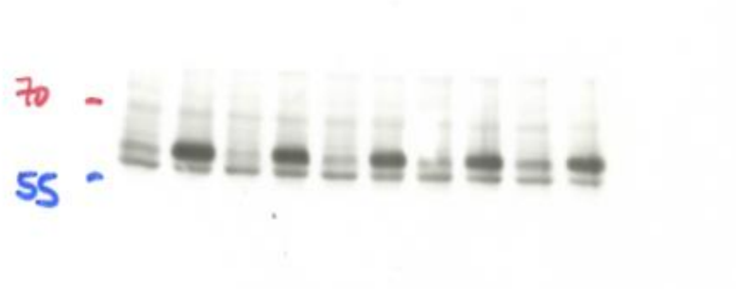

HKE:

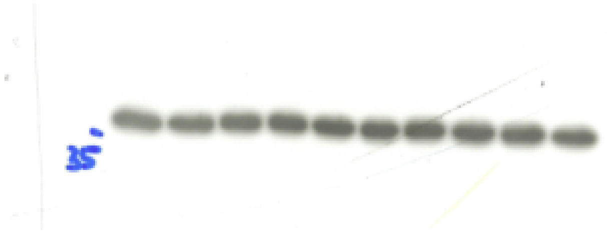

Round 5

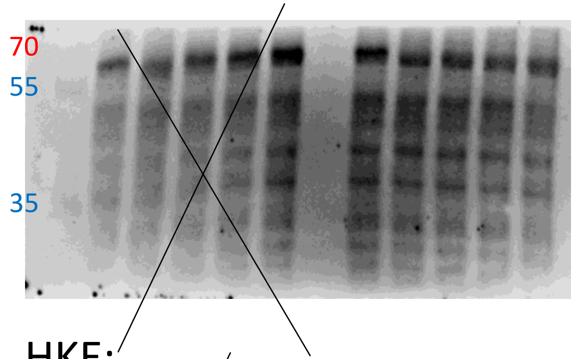

HKE:

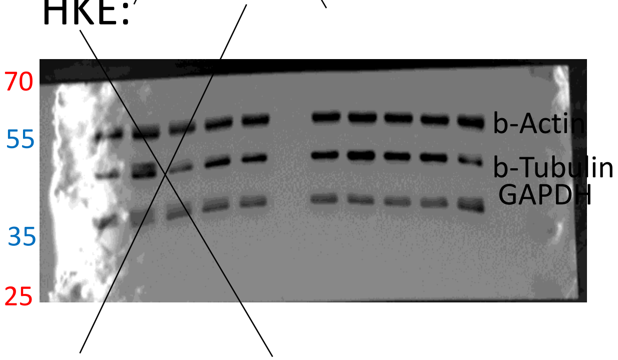

Round 6

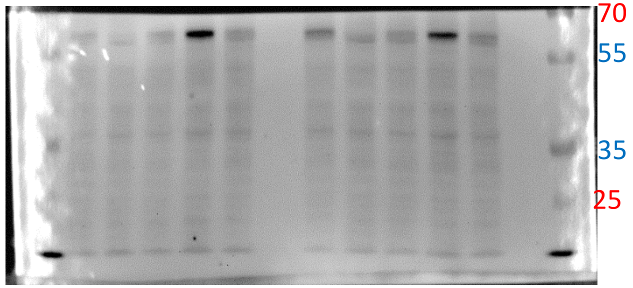

HKE:

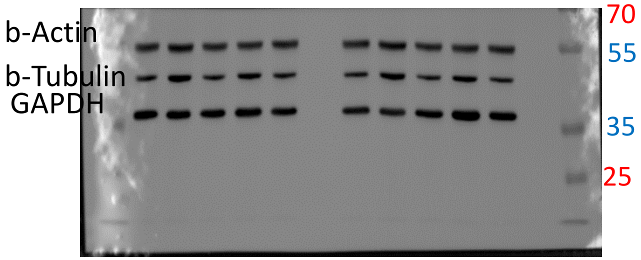

X only concerns figure 2

pMEK 1/2 (45kDa) // GAPDH (37kDa)

Round 1

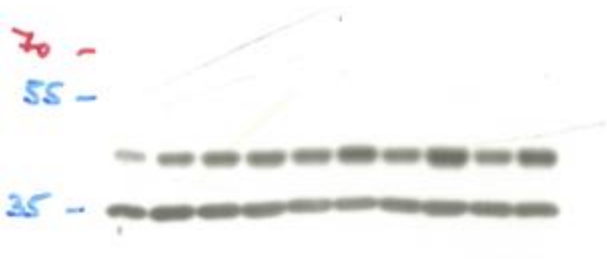

HKE

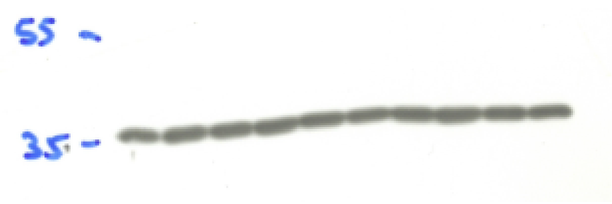

Round 2

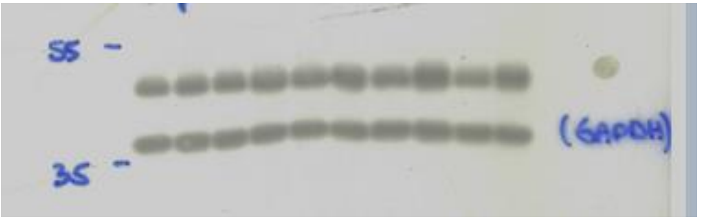

HKE

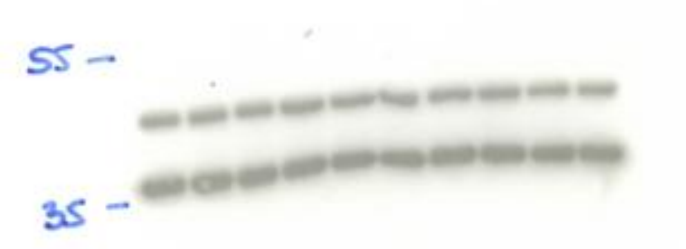

Round 3

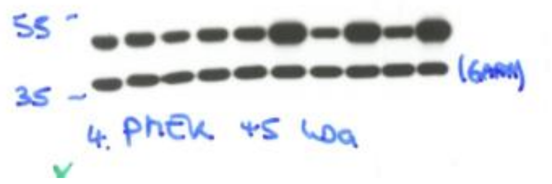

HKE

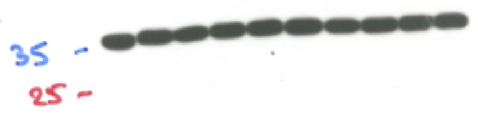

pMEK 1/2 (45kDa) // GAPDH (37kDa)

Round 4

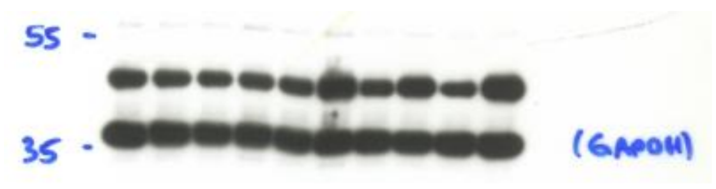

HKE

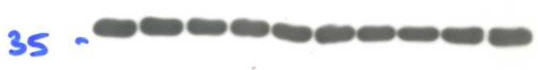

Round 5

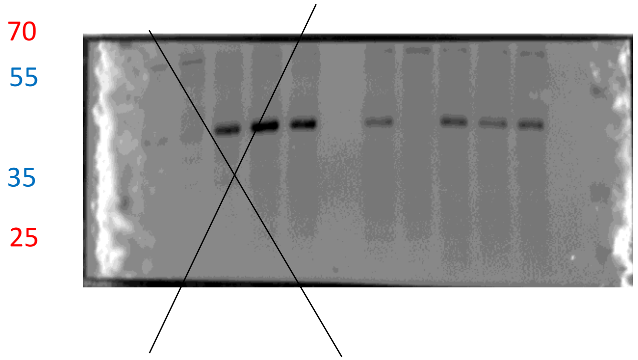

HKE

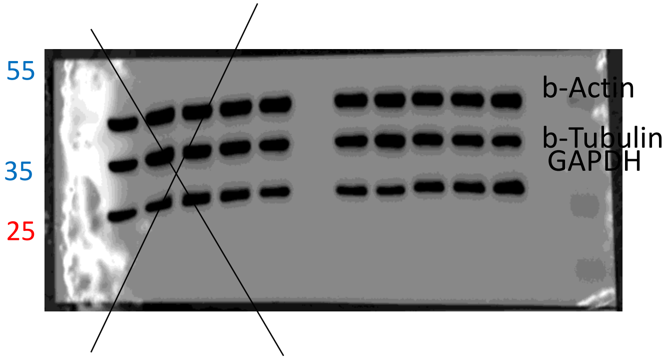

Round 6

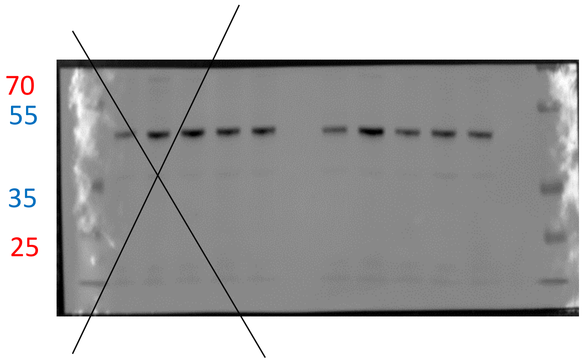

HKE

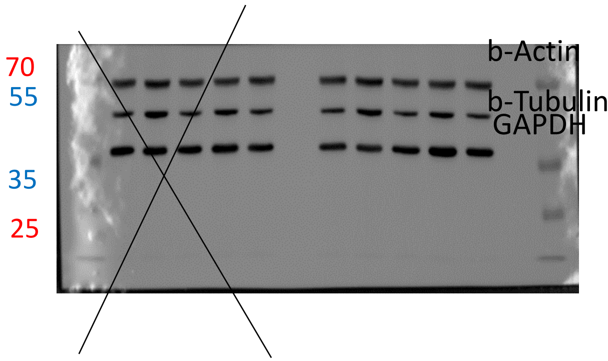

X only concerns figure 2

pERK 1/2 (45kDa) // GAPDH (37kDa)

Round 1

Round 2

Round 3

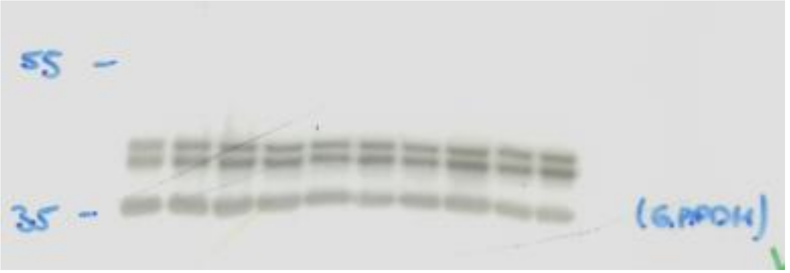

HKE

HKE

HKE

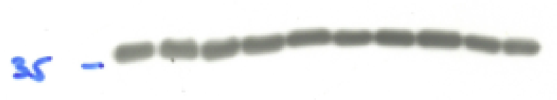

pERK 1/2 (45kDa) // GAPDH (37kDa)

Round 4

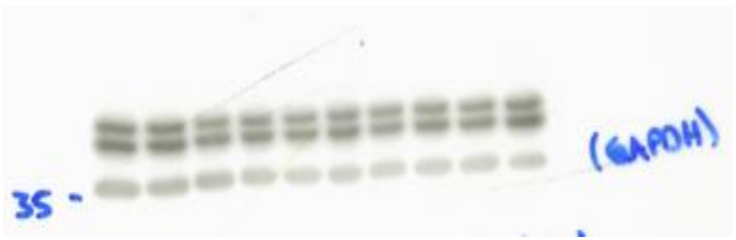

HKE:

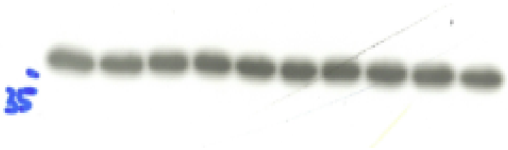

Round 5

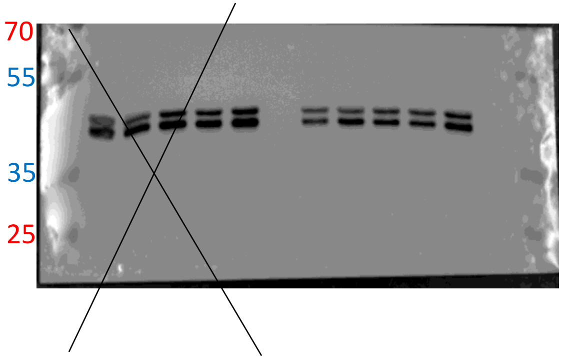

HKE:

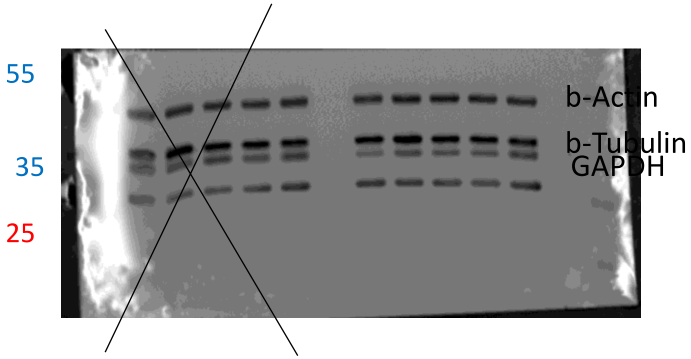

Round 6

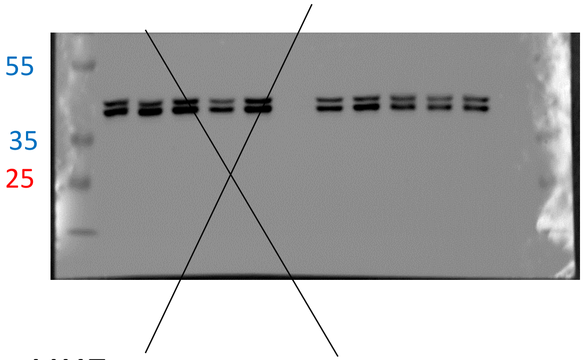

HKE:

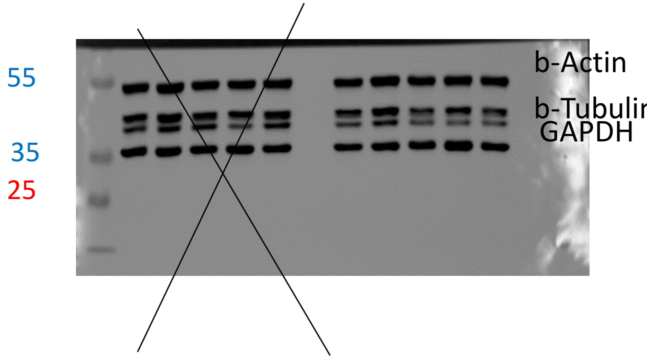

only concerns figure 2

Figure 4 (JJN-3)

Round 1-3: D1146N and N1129S loaded on different blots.
0.5% FBS is starved, only these bands used for analysis

Ø			LV			WT			Mut (D1146N / N1129S)		
0.5%	10%	IGF1	0.5%	10%	IGF1	0.5%	10%	IGF1	0.5%	10%	IGF1

Round 4:

Ladder	JJN-3		EV		$IGF1R^{WT}$		$IGF1R^{D1146N}$		$IGF1R^{N1129S}$	
	-	+	-	+	-	+	-	+	-	+

-: serum starved (unstimulated), only these bands used for analysis
+: stimulated with IGF1

Round 5-7:

Loading:

JJN-3	EV	$IGF1R^{WT}$	$IGF1R^{D1146N}$	$IGF1R^{N1129S}$

culturing medium, only concerns Figure 2

JJN-3	EV	$IGF1R^{WT}$	$IGF1R^{D1146N}$	$IGF1R^{N1129S}$

starving medium

IGF1R (95kDa) // GAPDH (37kDa)

Round 1

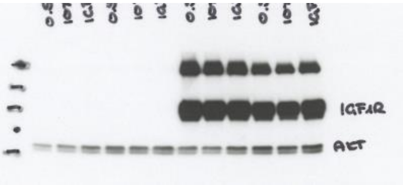

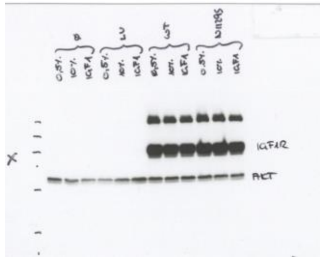

HKE:

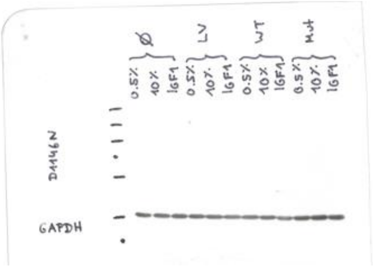

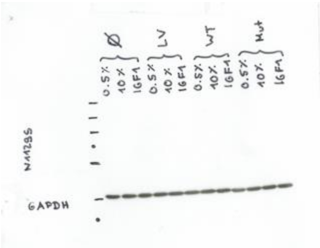

Round 2

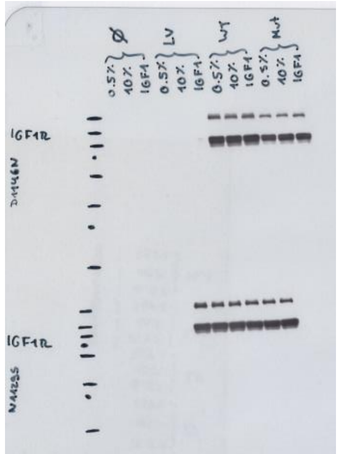

HKE:

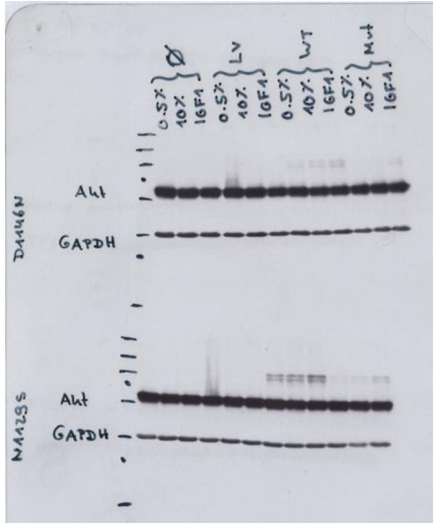

Round 3

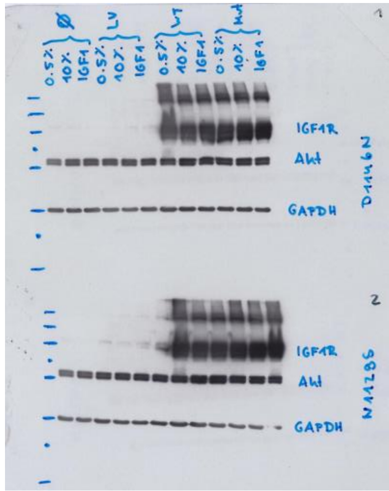

HKE:

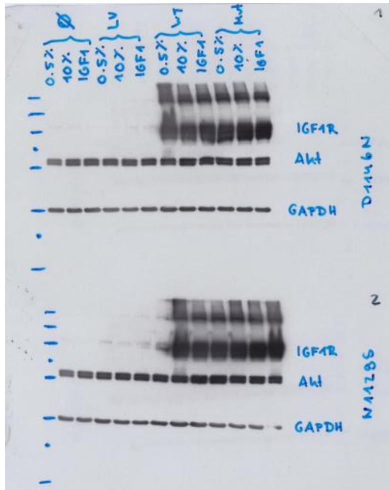

Round 4

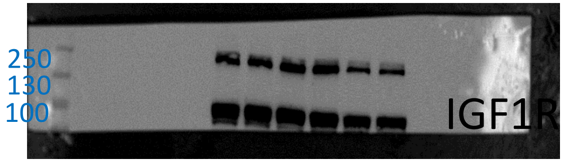

HKE:

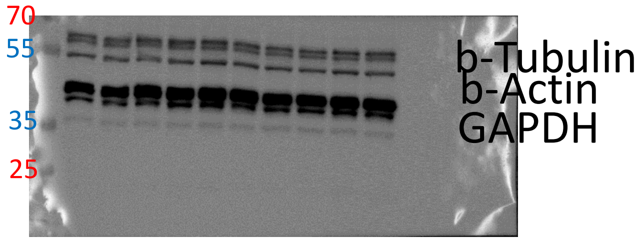

IGF1R (95kDa) // GAPDH (37kDa)

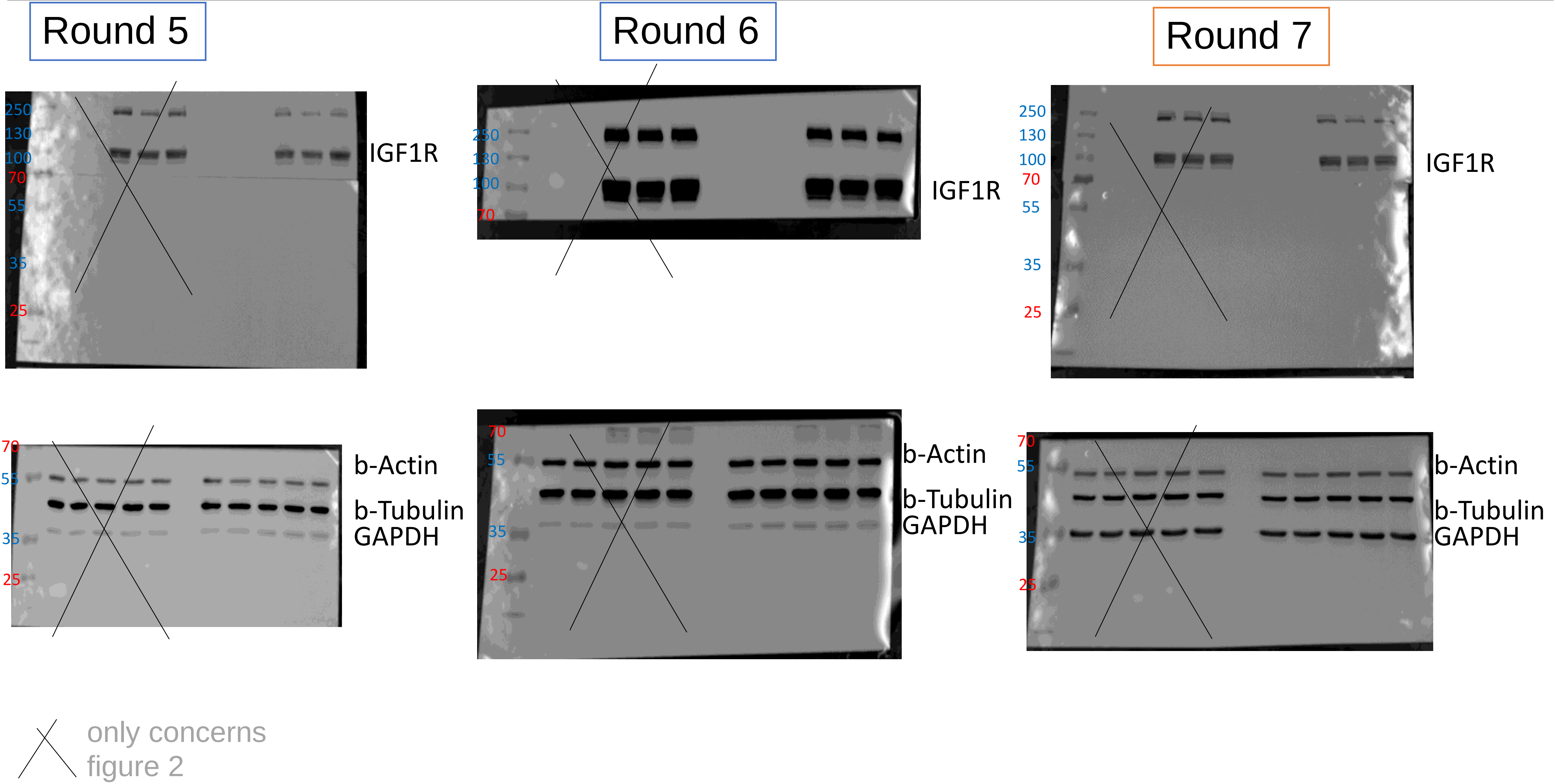

pIGF1R (Y1135) (95kDa) // GAPDH (37kDa)

Round 1

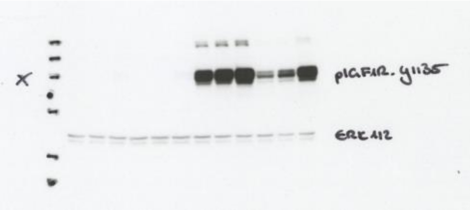
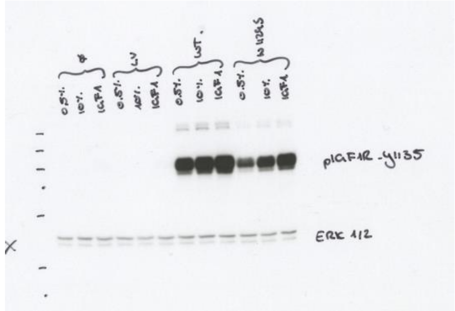

HKE:

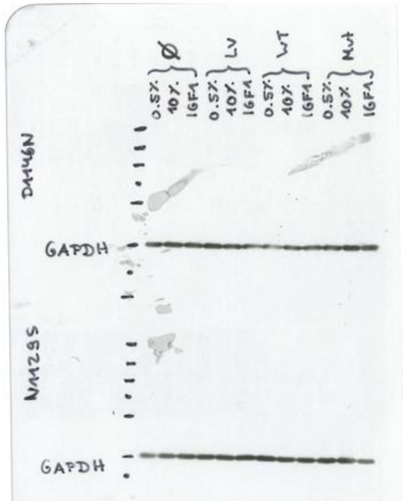

Round 2

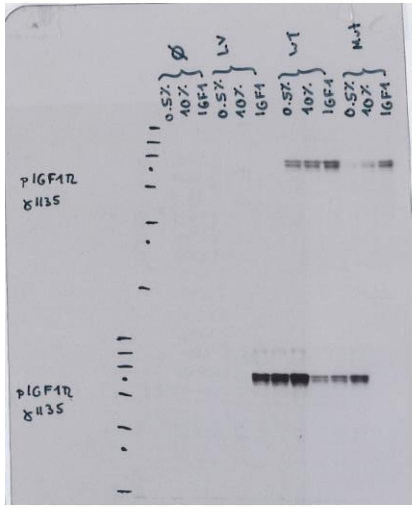

HKE:

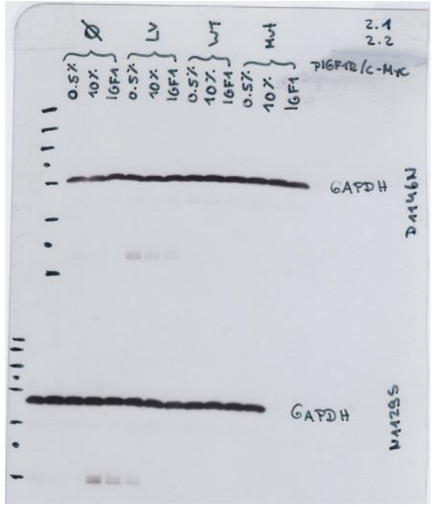

Round 3

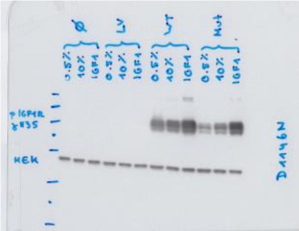
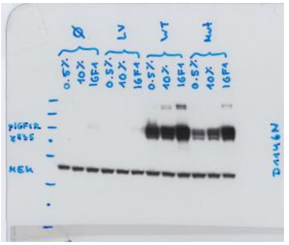

HKE:

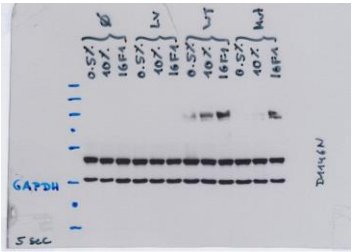
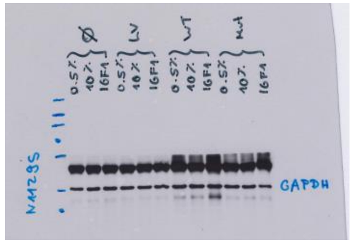

Round 4

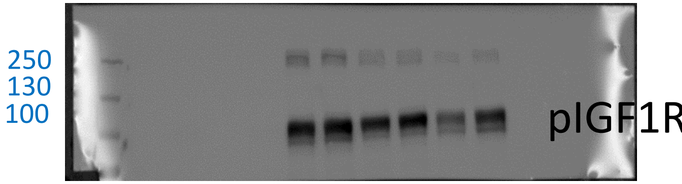

HKE:

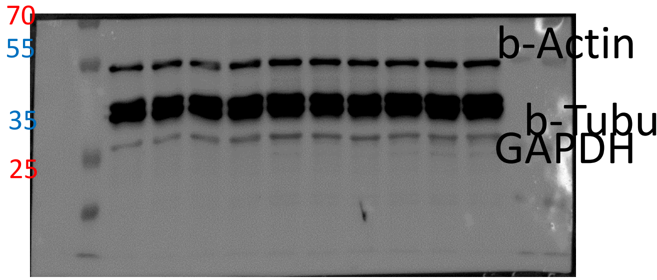

pIGF1R (Y1135) (95kDa) // GAPDH (37kDa)

Round 5

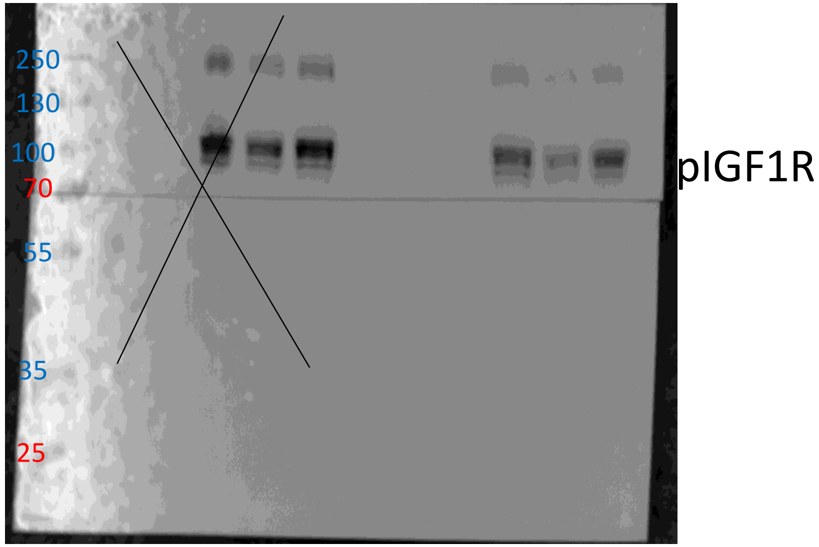

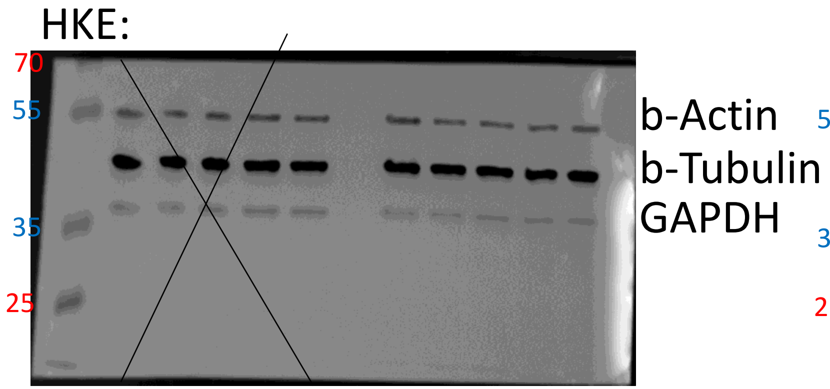

Round 6

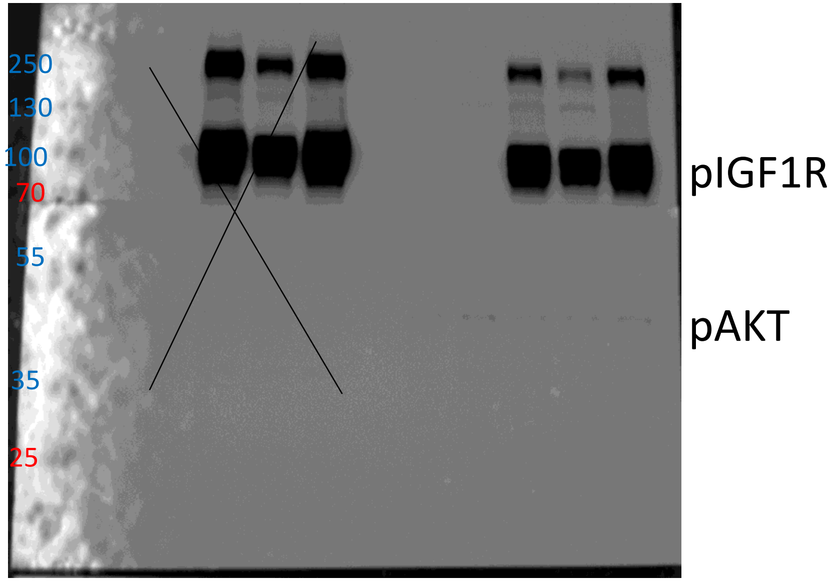

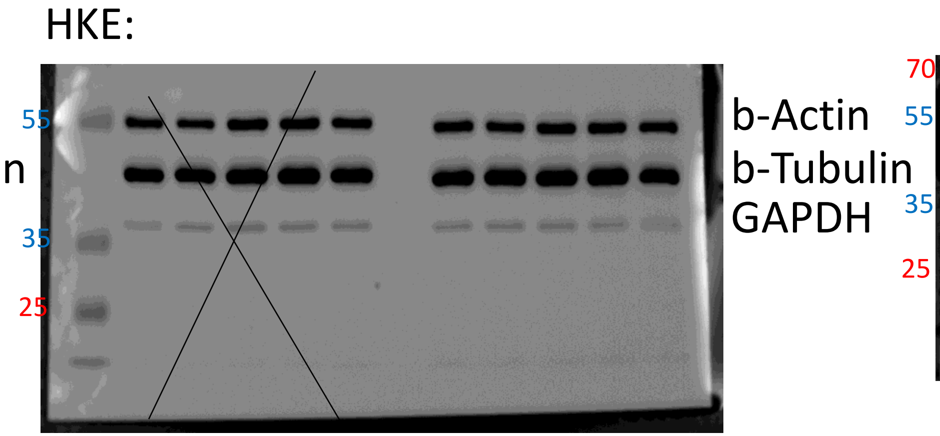

Round 7

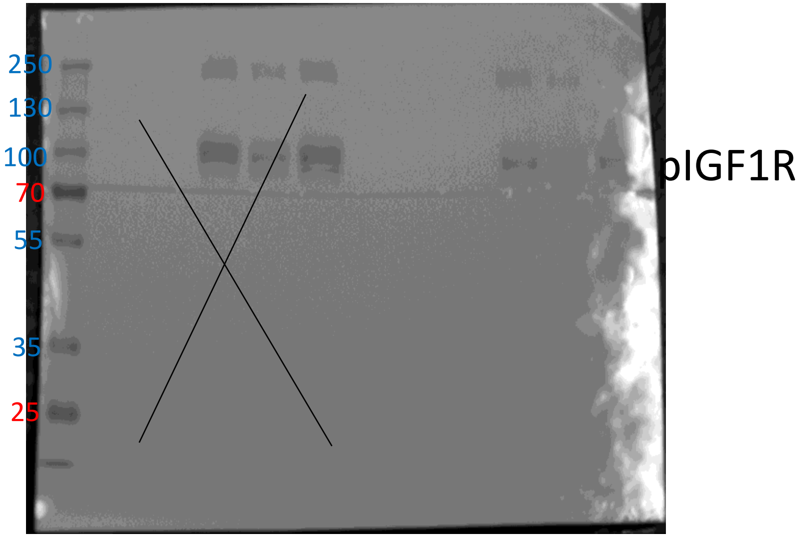

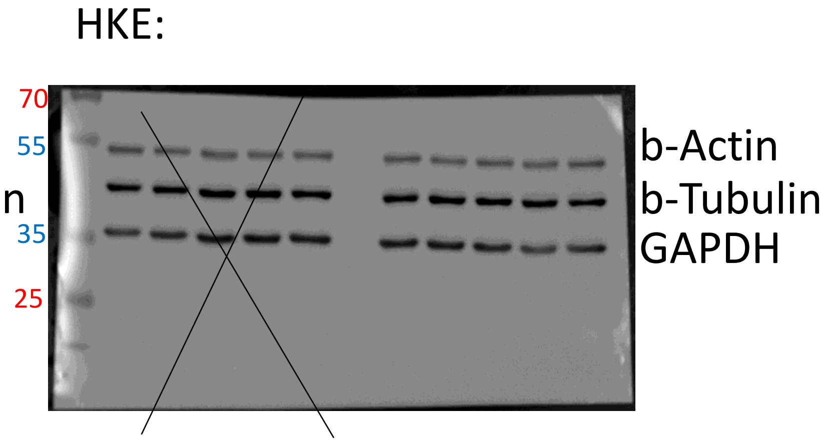

X only concerns figure 2

pAKT (60kDa) // GAPDH (37kDa)

Round 1

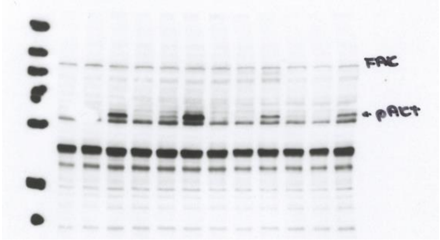

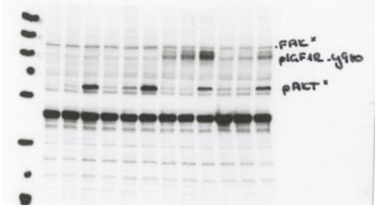

HKE:

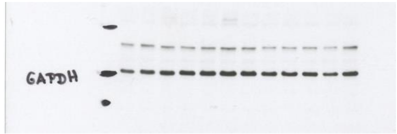

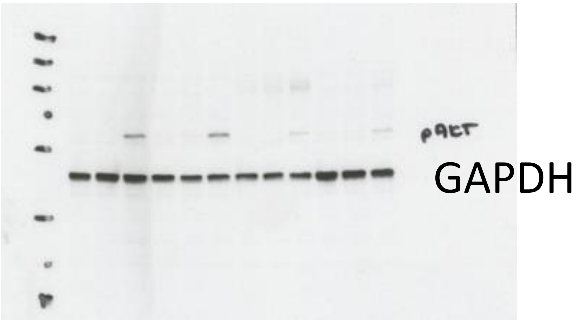

GAPDH

Round 2

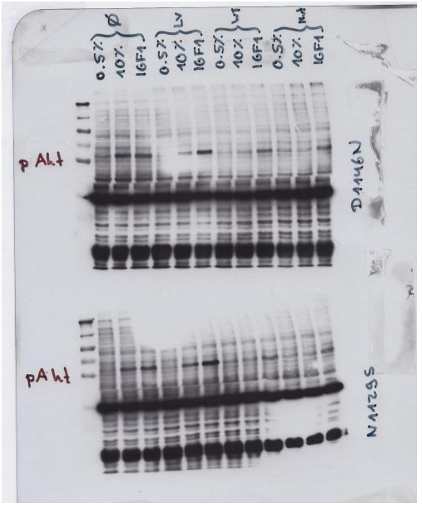

HKE:

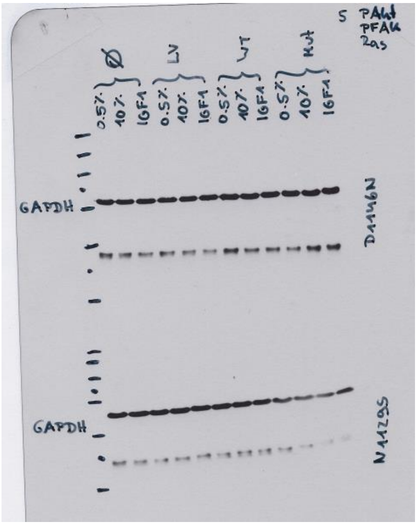

Round 3

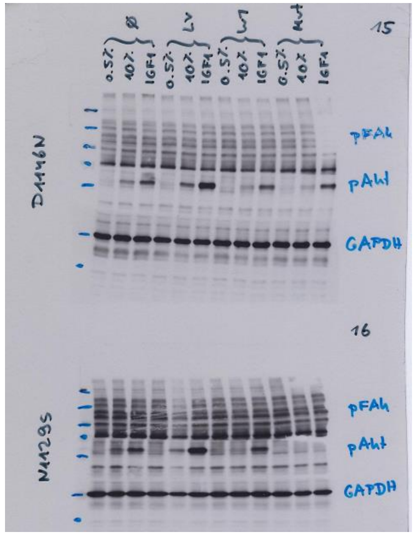

HKE:

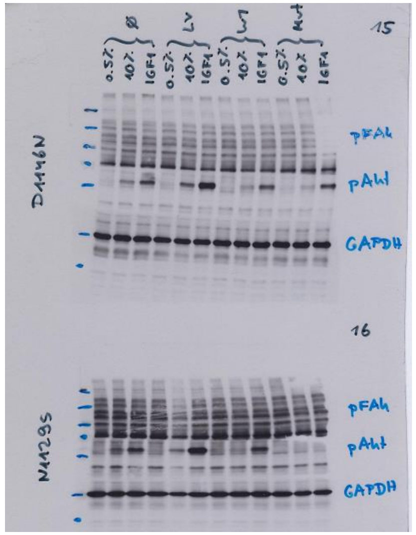

Round 4

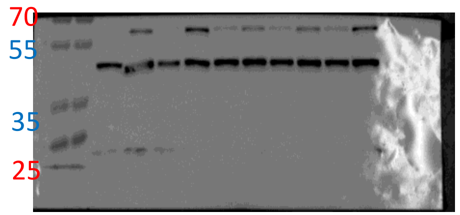

pAKT

HKE:

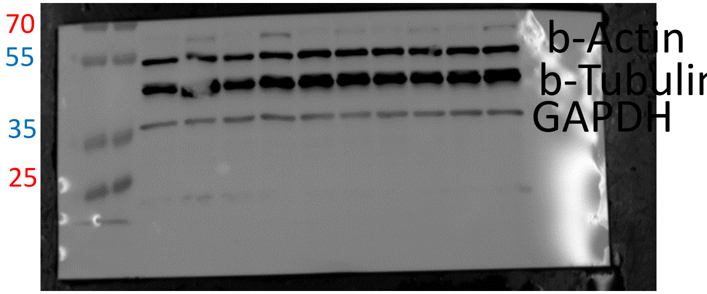

pAKT (60kDa) // GAPDH (37kDa)

Round 5

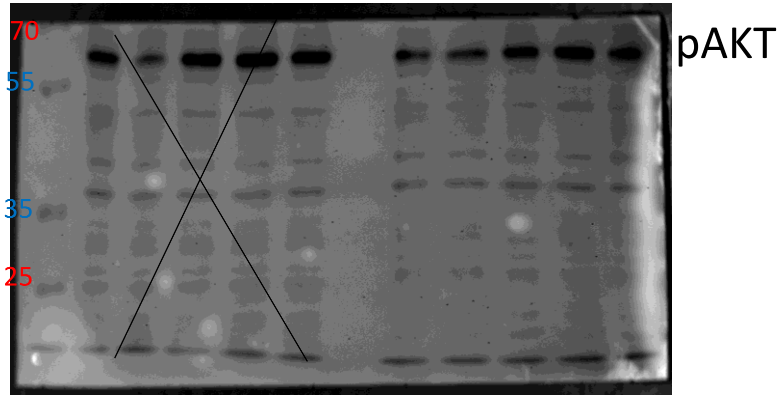

HKE:

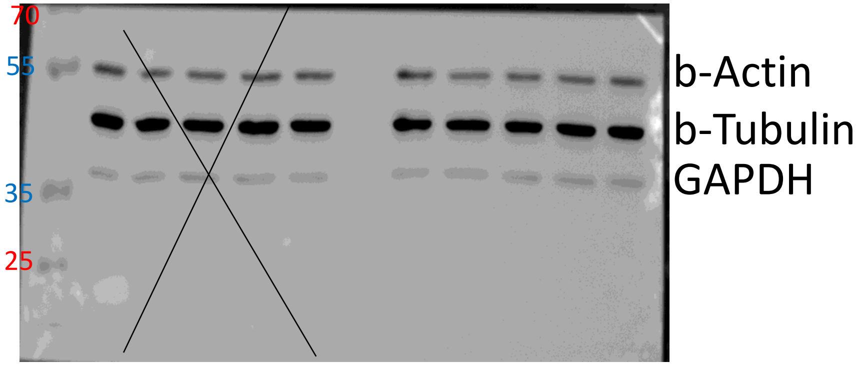

Round 6

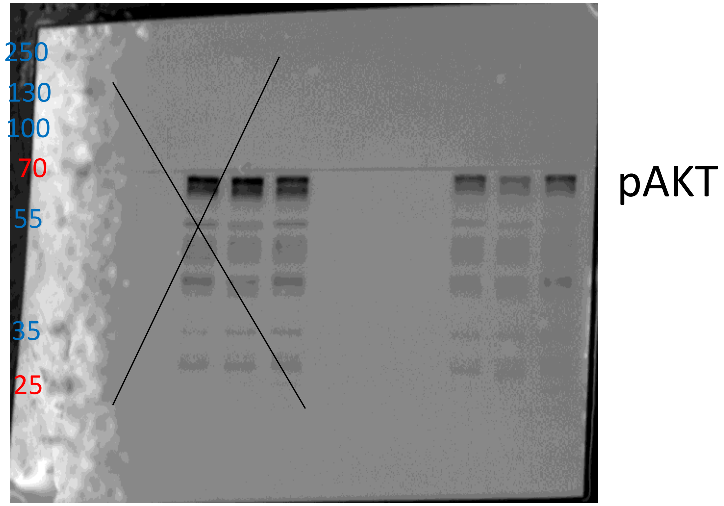

HKE:

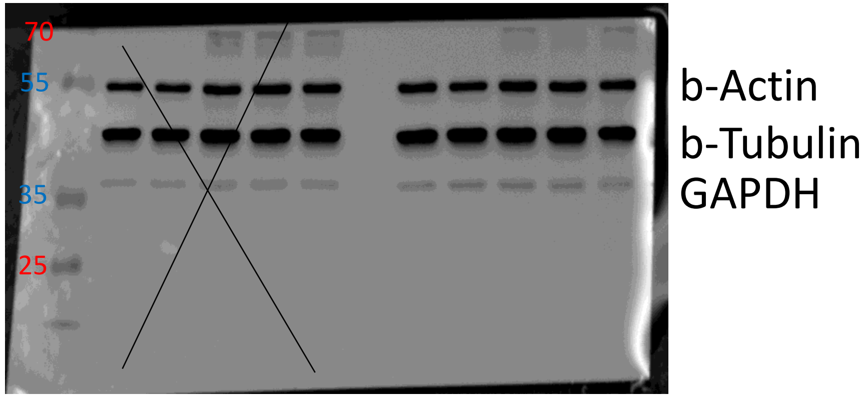

Round 7

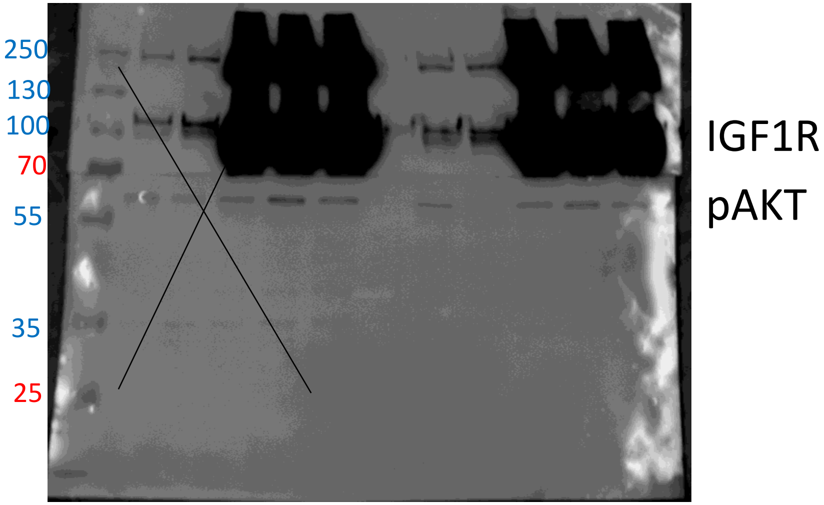

HKE:

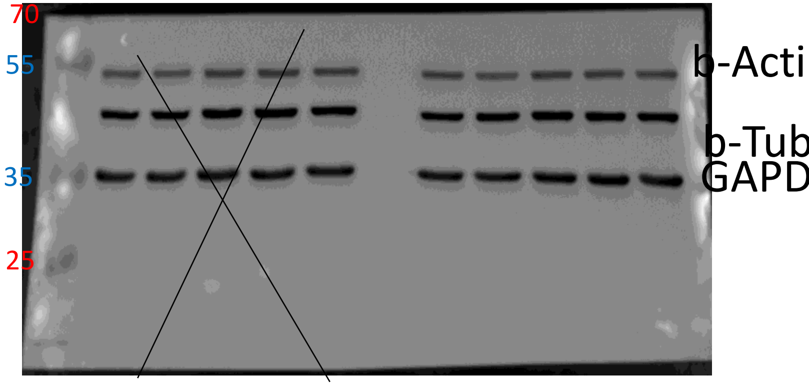

X only concerns figure 2

pMEK 1/2 (45kDa) // GAPDH (37kDa)

Round 1

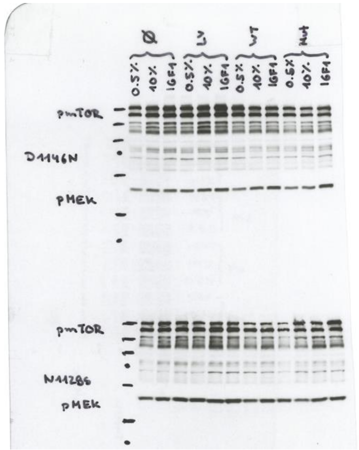

HKE:

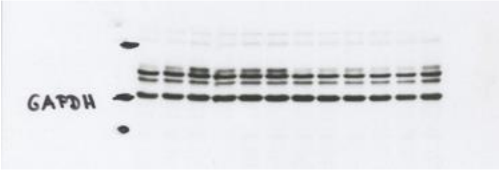

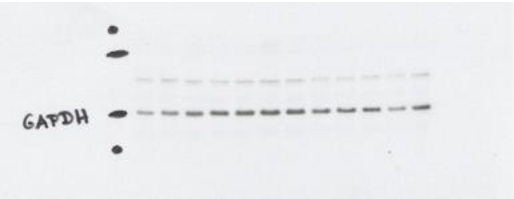

Round 2

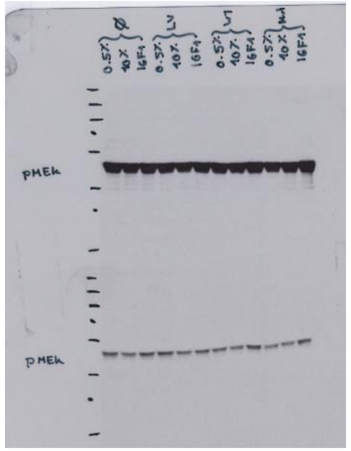

HKE:

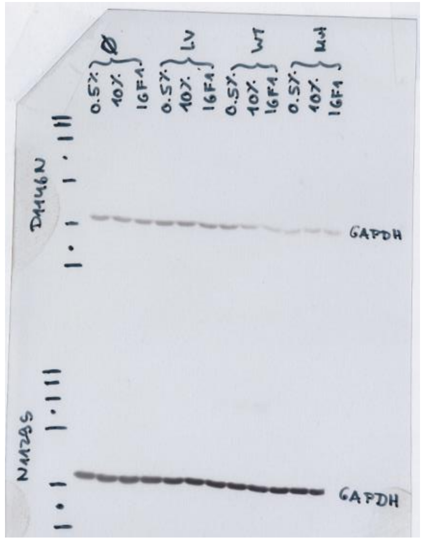

Round 3

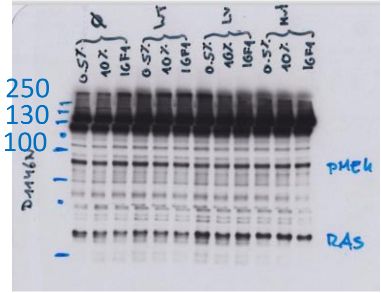

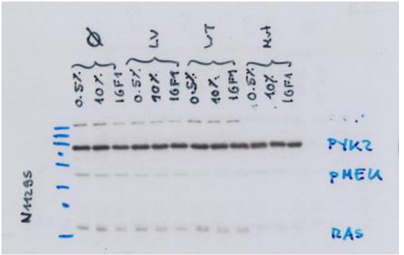

HKE:

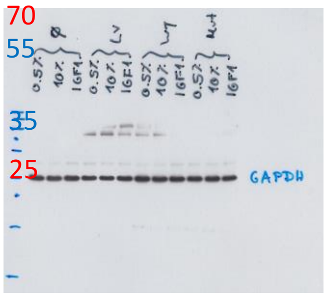

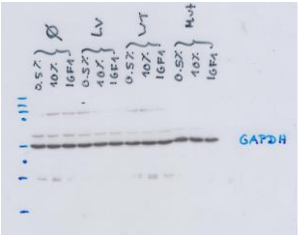

Round 4

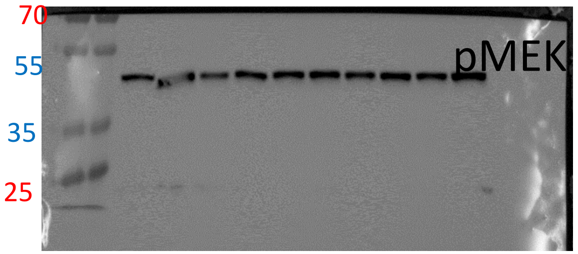

HKE:

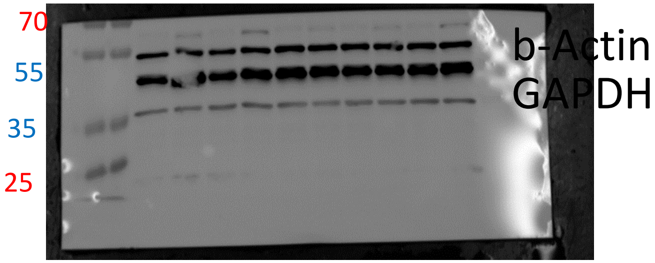

pMEK 1/2 (45kDa) // GAPDH (37kDa)

Round 5

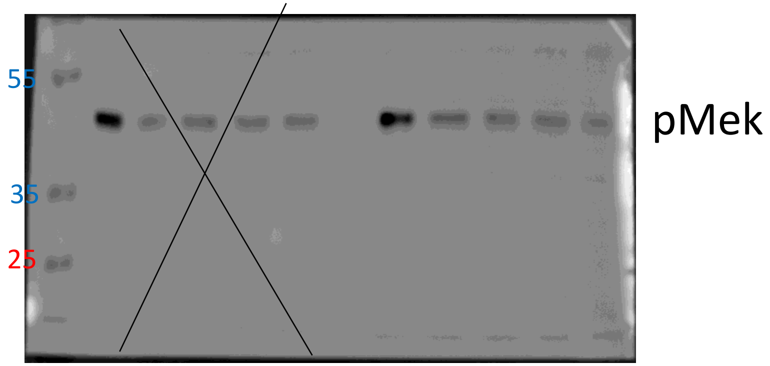

HKE:

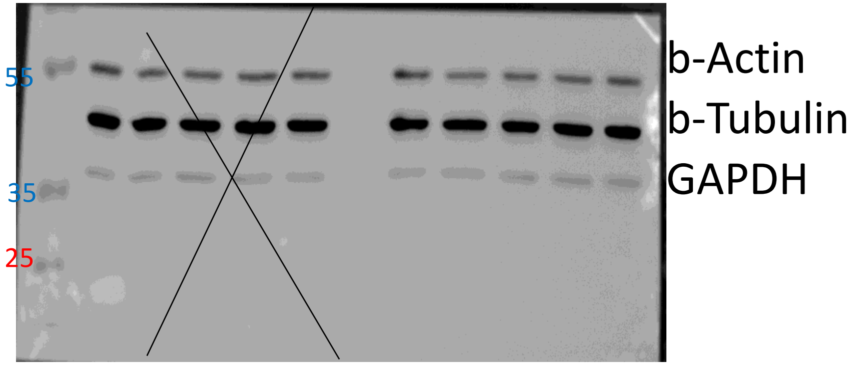

Round 6

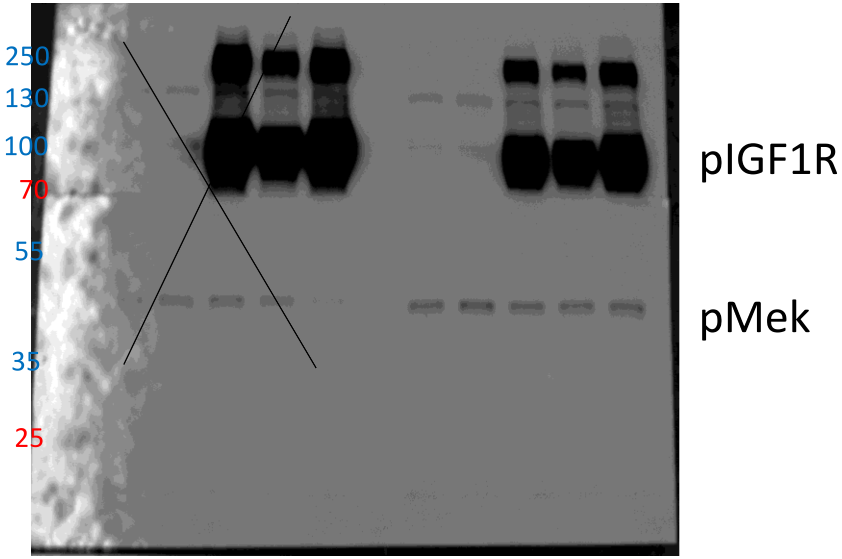

HKE:

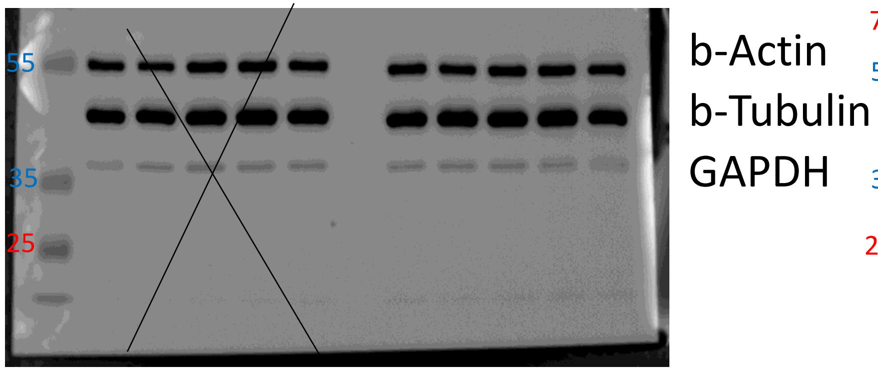

Round 7

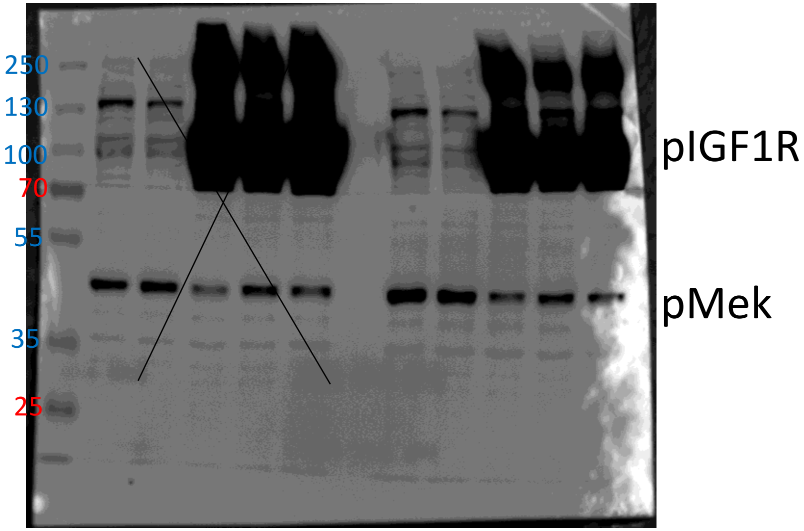

HKE:

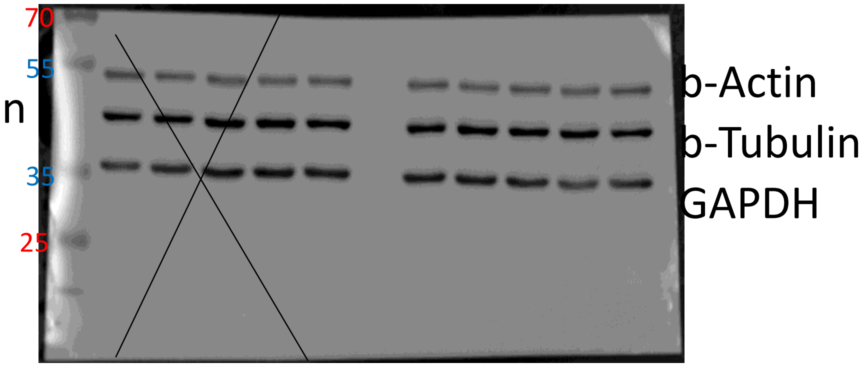

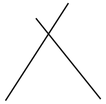

only concerns figure 2

pERK 1/2 (45kDa) // GAPDH (37kDa)

Round 1

Round 2

Round 3

Round 4

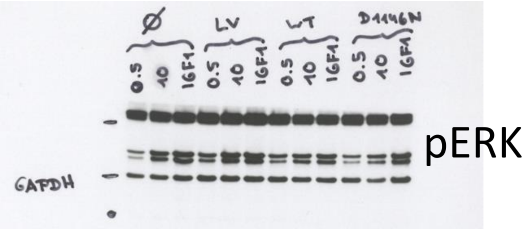

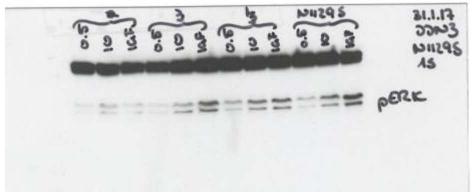

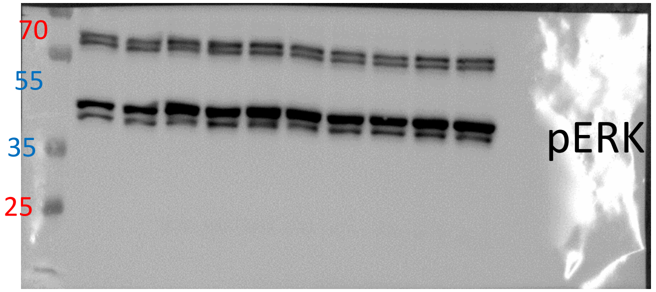

HKE:

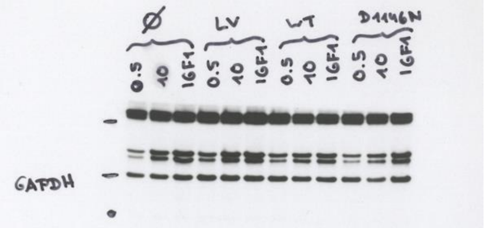

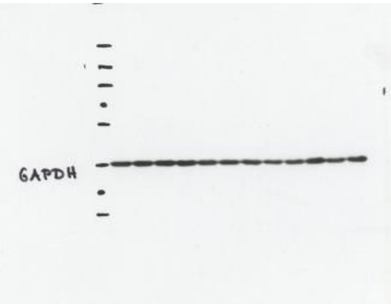

HKE:

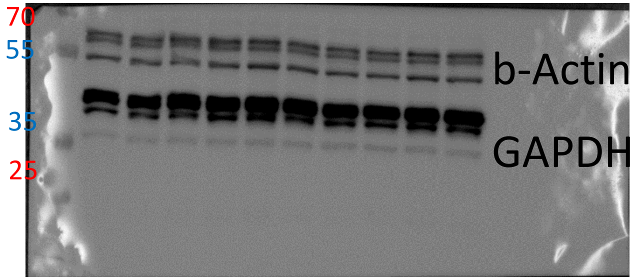

pERK 1/2 (45kDa) // GAPDH (37kDa)

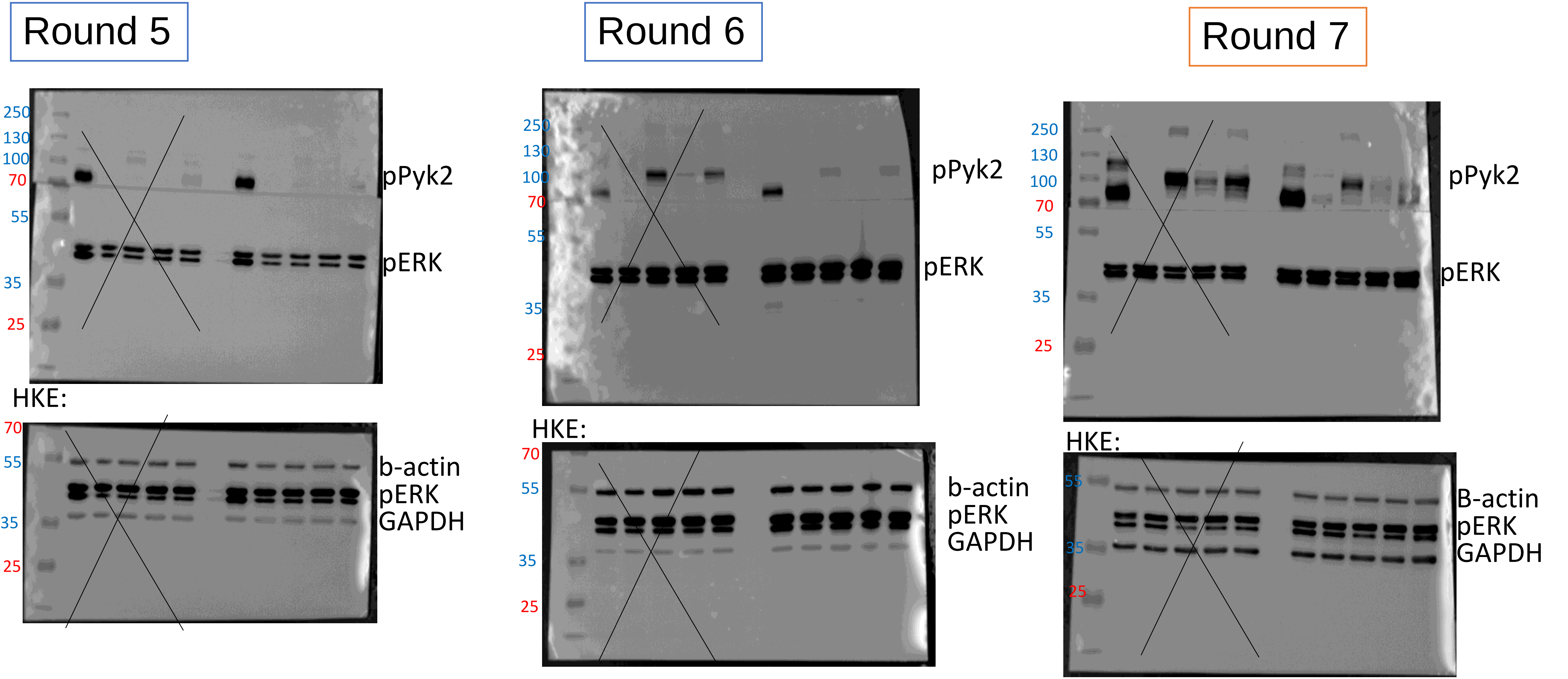

only concerns figure 2

Figure 5

Ladder

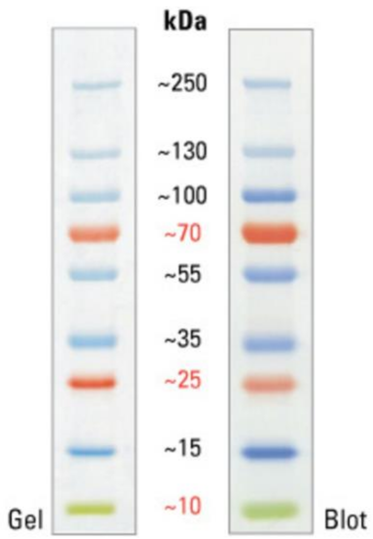

PageRuler™ Plus
Prestained Protein
Ladder, 10 to 250 kDa
Katalognummer: #26
619 Thermo Fischer

Markers (Figure 5B):

IGF1R
pIGF1R(Y1135)
pAKT
PARP1
Caspase 9

House keepers (HKEs):
b-actin
b-tubulin
GAPDH

Round depicted
in WB figure

Figure 5B

Loading:	KMS-12BM	AMO-1	MM.1s	KMS-11	L-363	JJN-3	U-266
	- +	- +	- +	- +	- +	- +	- +

-: DMSO

+: 0.4µM linisitinib (24h)

IGF1R (95kDa) /HKES

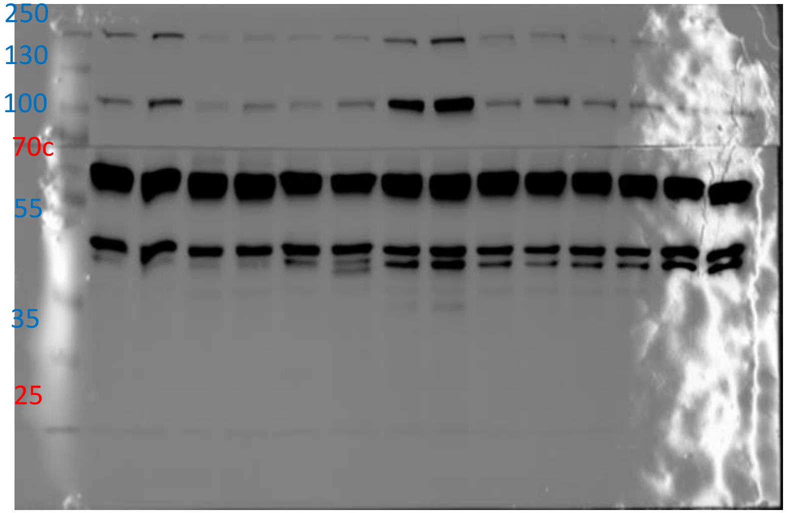

HKE:

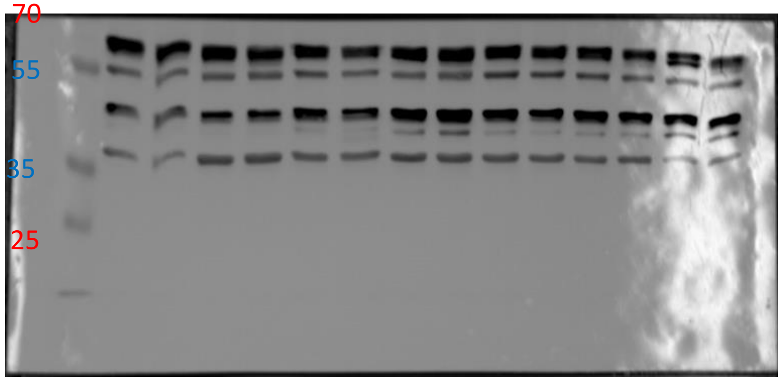

pIGF1R (Y1135) (95kDa) /HKES

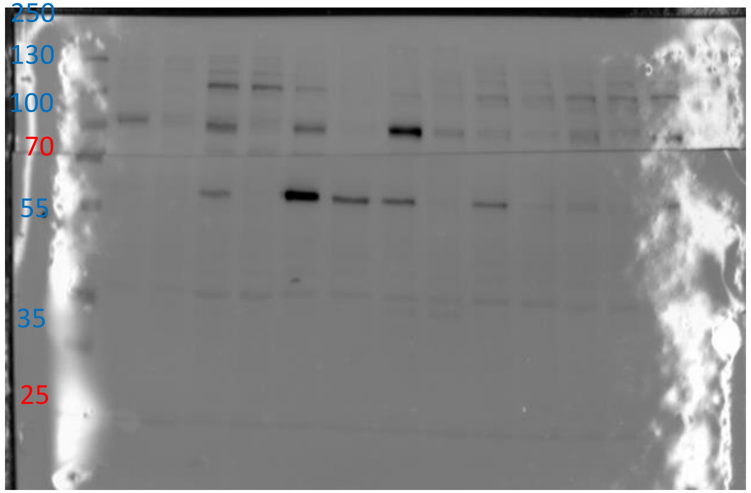

HKE:

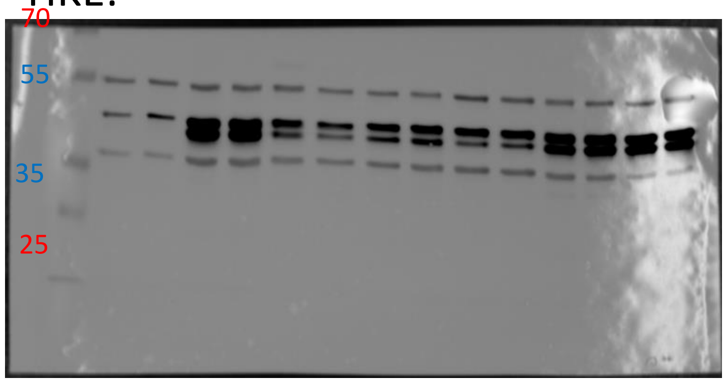

pAKT(60kDa) /HKES

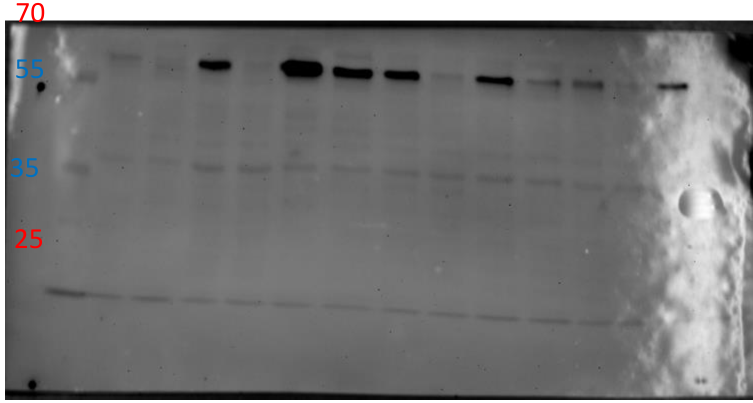

HKE:

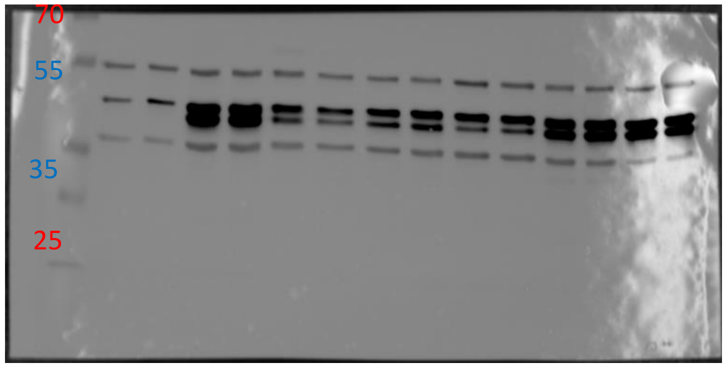

PARP (116, 89 kDa) /HKES

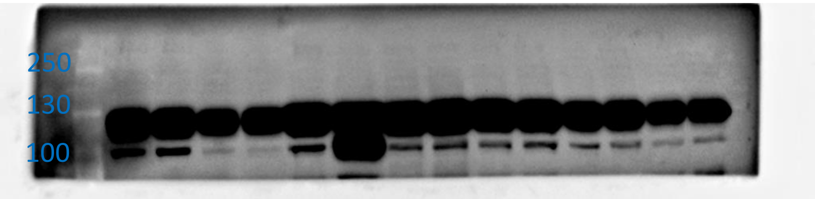

HKE:

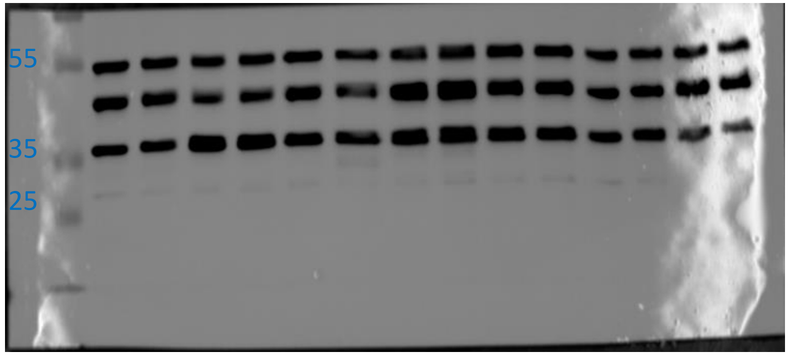

Caspase 9 (47, 37,35kDa) /HKES

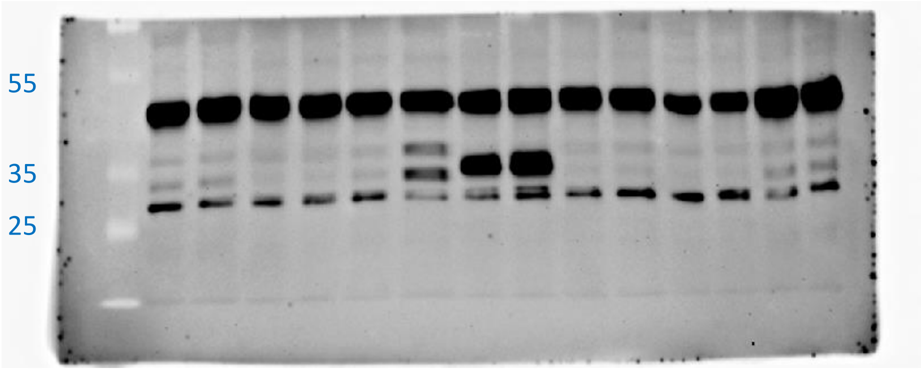

HKE:

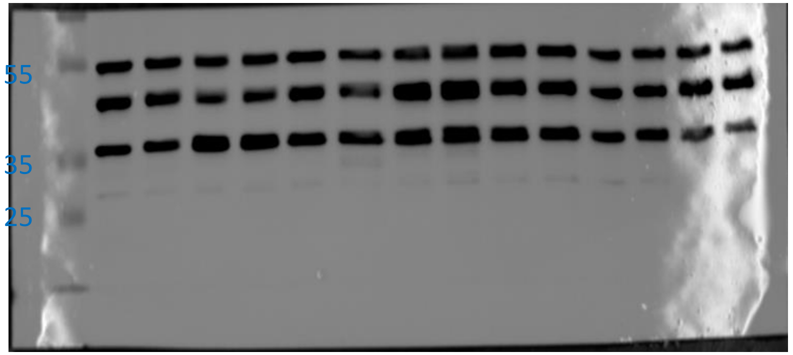

Ladder

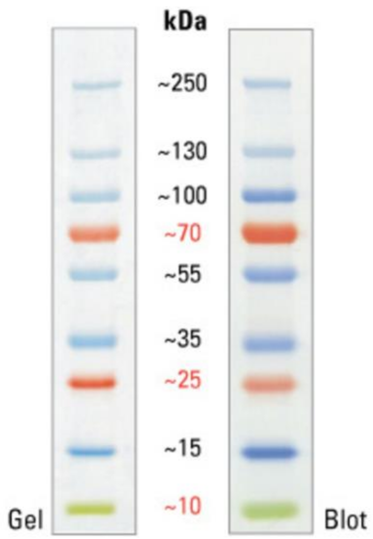

PageRuler™ Plus
Prestained Protein
Ladder, 10 to 250 kDa
Katalognummer: #26
619 Thermo Fischer

Markers (Figure 5C):

IGF1R
pIGF1R(Y1135)
PARP1
Caspase 9

House keepers (HKEs):
b-actin
b-tubulin
GAPDH

Round depicted

in WB figure

PARP (116, 89 kDa) /HKES

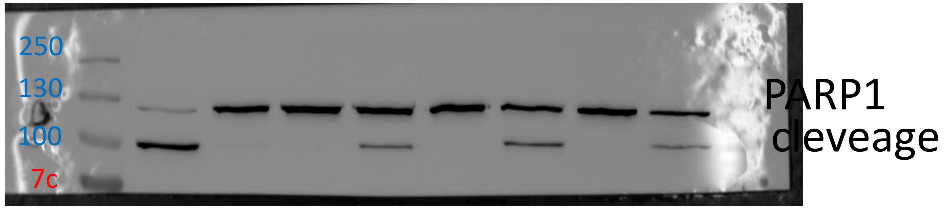

HKE:

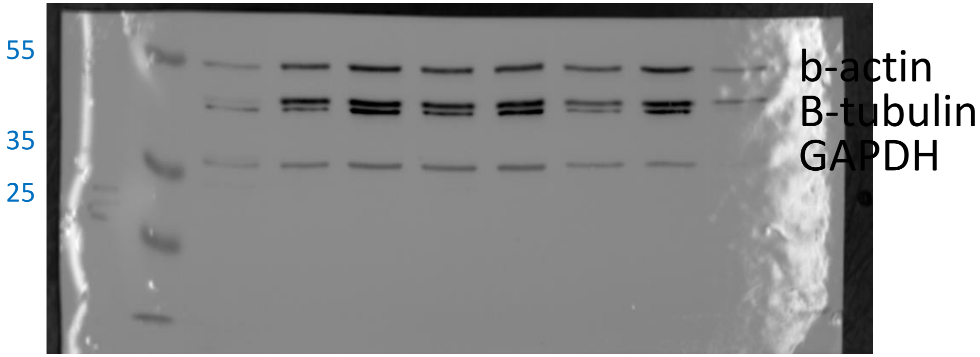

Caspase 9 (47, 37,35kDa) /HKES

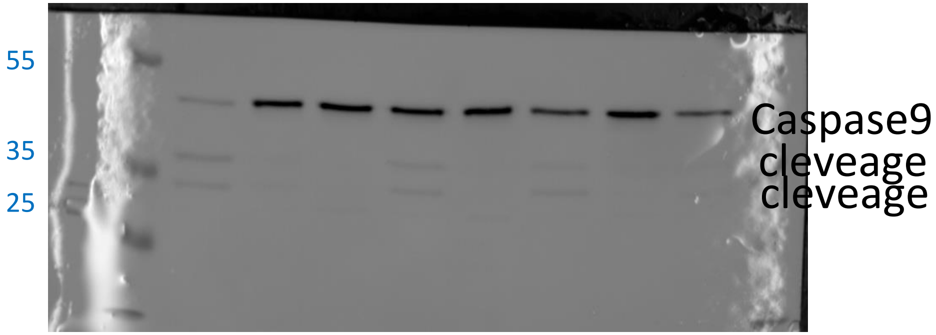

HKE:

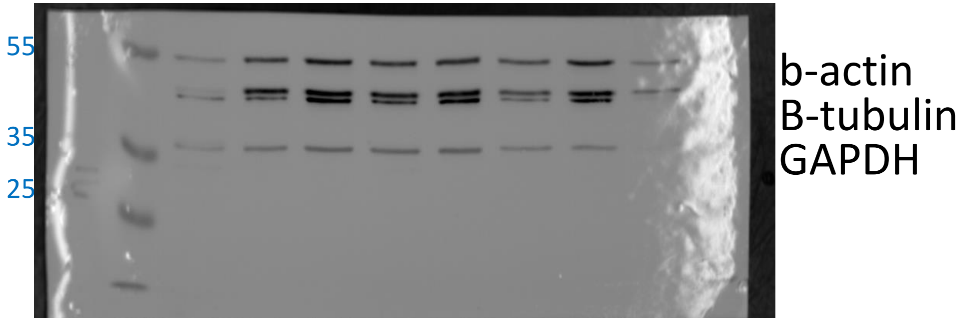

Supplement: Supplementary file 1 [file cancers-16-02139-s001.zip › Supplementary methods_Uncropped_Western_blots.pdf]
