# Supplementary material for: Functional Investigation of IGF1R Mutations in Multiple Myeloma
Source: Cancers (Basel). 2024 Jun 4;16(11):2139. doi: 10.3390/cancers16112139 (PMC11171363; doi:10.3390/cancers16112139)
Supplement: Supplementary file 1 [file cancers-16-02139-s001.zip › Supplementary Tables S2-S4.pdf]

Supplemental Tables

Table S2: Primers used for mutagenesis PCR

Primer		Sequence	°C T_M	% GC
IGF1R_L363_Mut_G/A	F	GCATGGTAGCCGAAAATTTCACAGTC	59	46
	R	GACTGTGAAATTTTCGGCTACCATGC	59	46
IGF1R_MM882_Mut_A/G	F	CATACCTCAACGCCAGTAAGTTCG	58	50
	R	CGAACTTACTGGCGTTGAGGTATG	58	50

Table S3: Primers used for cloning of expression and donor vectors

Primer		Sequence	°C T_M	% GC
IGF1R_NotI	F	ACGGCGGCCGCACC ATGAAGTCTG	67.9	67
IGF1R_EcoRI_Stop	R	ACGCGAATTCTCAGCAGGTCGAA	60.9	52
IGF1R_NheI	F	ACGGCTAGCACCATGAAGTCTG	61.9	55
IGF1R_NotI	R	ACGCGCGGCCGCTCAGCAGGTCGAA	72.4	72

Table S4: Primer used for Sanger sequencing of ligated plasmids

Primer		Sequence	°C T_M	% GC
IGF1R_seq_1	F	CTGACAACGACACGGCCT	57.9	61
IGF1R_seq_1	R	CAAACCTGTAGGTGTTGGGC	56.3	55
IGF1R_seq_2	F	ATCATCATAACCTGGCACCG	54.9	50
IGF1R_seq_2	R	GCATCCTGCCCATCATACTC	55.6	55
IGF1R_seq_3	F	CCTTTCTTTGAGAGCAGAGTGG	55.5	50
IGF1R_seq_3	R	TGGATATCGATGCGGTACAA	53.5	45
IGF1R_seq_4	F	CATGCGTGAAAGGATTGAGTT	53.6	43
IGF1R_seq_4	R	GACACCACACCCAGCAATC	56.4	58
IGF1R_seq_5	F	CTCCTCGTCCTCCCTGCC	60.1	72
IGF1R_seq_5	R	CTCTCGTCGAAGCTGGCG	58.5	67
M13_fw	F	GTAAAACGACGGCCAGT	52.6	53
M13_rv	R	AACAGCTATGACCATG	45.4	44
CMV_fw	F	CGCAAATGGGCGGTAGGCGTG	63.9	67
